# Supplementary material for: PKM2 functions as a histidine kinase to phosphorylate PGAM1 and increase glycolysis shunts in cancer
Source: EMBO J. 2024 May 15;43(12):5. doi: 10.1038/s44318-024-00110-8 (PMC11183095; doi:10.1038/s44318-024-00110-8)
Supplement: Supplementary file 1 — Appendix [file 44318_2024_110_MOESM1_ESM.pdf]

APPENDIX

PKM2 functions as a histidine kinase to phosphorylate PGAM1 and increase glycolysis shunts in cancer

Yang Wang, Hengyao Shu, Yanzhao Qu, Xin Jin, Jia Liu, Wanting Peng, Lihua Wang, Miao Hao, Mingjie Xia, Zhexuan Zhao, Kejian Dong, Yao Di, Miaomiao Tian, Fengqi Hao, Chaoyi Xia, Wenxia Zhang, Xueqing Ba, Yunpeng Feng, Min Wei

TABLE OF CONTENTS

Appendix Figure S1. PKM2 and ENO1 physically interacts with PGAM1 .... page 2

Appendix Figure S2. Assay for PGAM1 activity and pyruvate generation .... page 3

Appendix Figure S3. Analysis of PGAM1 tyrosine phosphorylation by mass spectrometry .... page 4

Appendix Figure S4. PGAM1, PKM2 and Src are highly expressed in tumor tissues .... page 5

Appendix Figure S5. The impact of PEP levels on PGAM1 H11 phosphorylation .... page 6

Appendix Figure S1

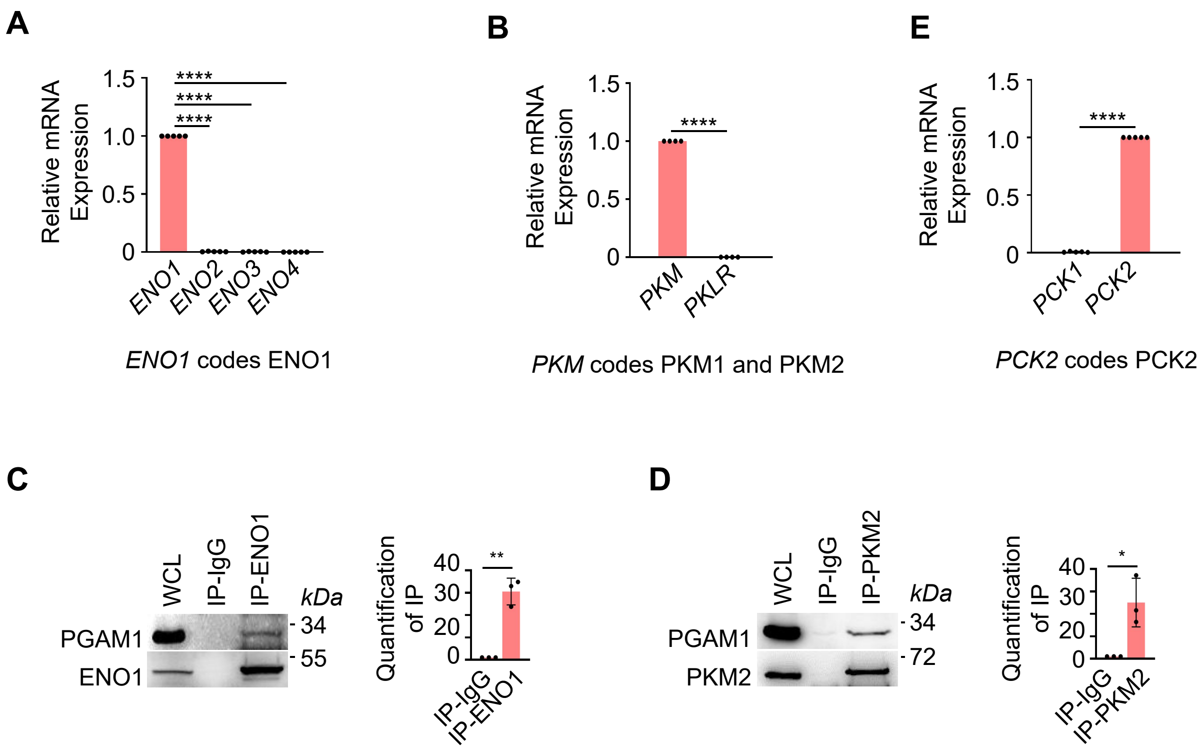

Appendix Figure S1. PKM2 and ENO1 physically interacts with PGAM1.

(A, B) *PKM* and *ENO1* are the predomiantly transcribed genes of PKs and ENOs families. Quantitative real-time polymerase chain reaction (qPCR) was performed with specific primers for mRNA of ENOs (*ENO1, ENO2, ENO3, ENO4*) and PKs (*PKM, PKLR*).

(C, D) PKM2 and ENO1 exist in the PGAM1-associated protein complex. *(left)* PKM2- or ENO1-associated proteins in A549 cells were immunoprecipitated and analyzed by WB. IgG served as a negative control. One representative experiment out of three was shown. (*right*) Quantification of IP. Relative levels of PGAM1 in IP were normalized to that in WCL for each group.

(E) *PCK2* is the predomiantly transcribed genes of PCKs families. qPCR was performed with specific primers for mRNA of PCKs (*PCK1, PCK2*).

Data information: data represent mean ± SD of four (A, B, E) or three (C, D) independent experiments, with significance determined by one-way ANOVA test (A, B, E) or student's *t*-test (C, D); ****P<0.0001, **P<0.01, *P<0.05.

Appendix Figure S2

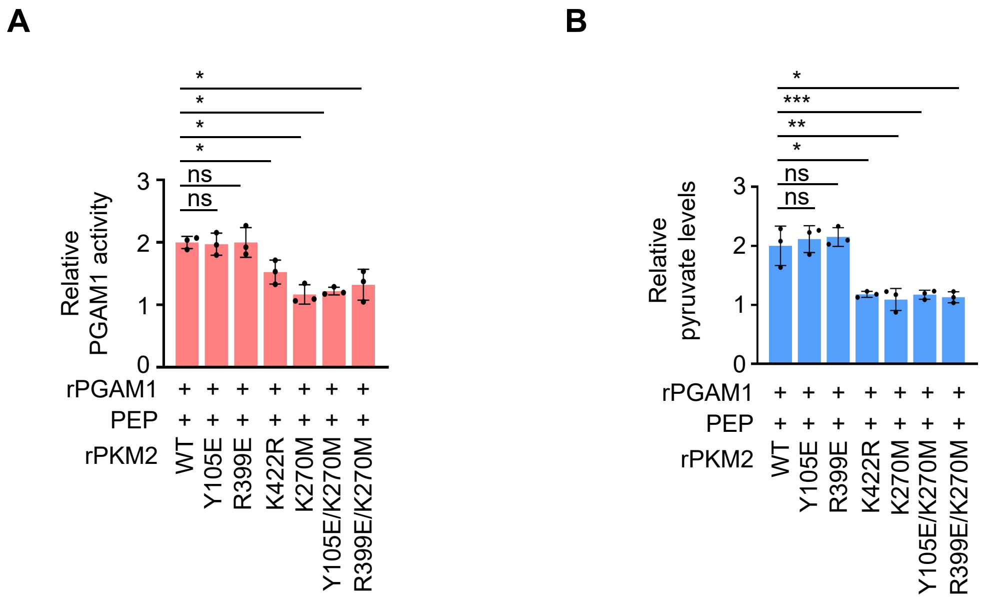

Appendix Figure S2. Assay for PGAM1 activity and pyruvate generation.

(A, B) PKM2 Y105E and R399E mutations are favorable for PGAM1 activity than K422R mutation. The PGAM1 activity (A) and pyruvate level (B) from the *in vitro* kinase assay in Fig. 2C were respectively detected by commercially available kits. Data represent mean ± SD of three (A, B) independent experiments with significance determined by one-way ANOVA test; ***P<0.001, **P<0.01, *P<0.05, ns, nonsignificant.

Appendix Figure S3

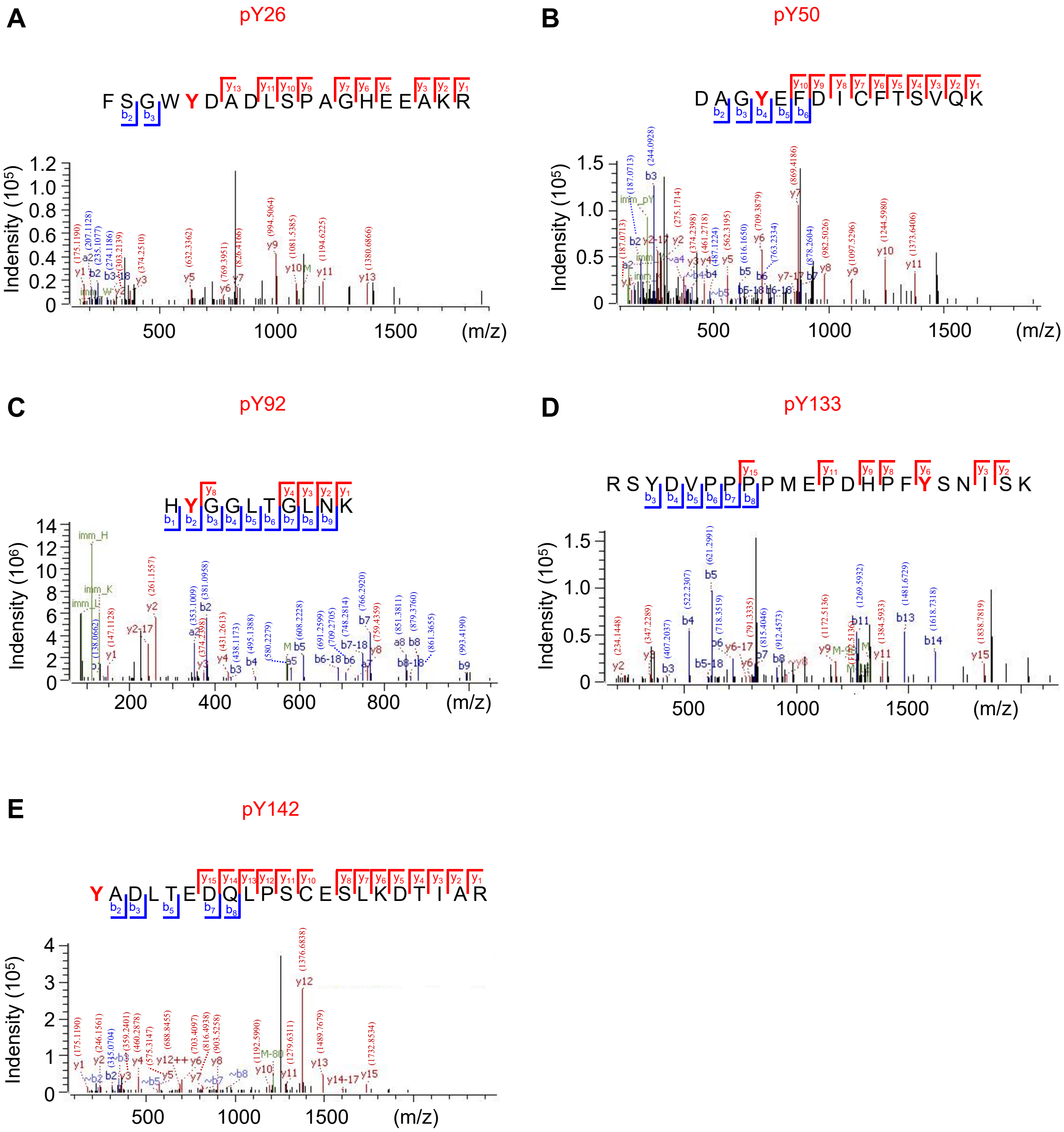

Appendix Figure S3. Analysis of PGAM1 tyrosine phosphorylation by mass spectrometry.

(A-E) The PGAM1 tyrosine-phosphorylated sites are analyzed by MS. PGAM1 protein immunoprecipitated from A549 cells was trypsinized and then analyzed by MS. MS data were processed with the Byonic engine and identified the peptide including Y26, Y50, Y92, Y133 and Y142 phosphorylation sites respectively. The y and b fragmentations were used to map the phosphorylation sites to the Tyr indicated in red.

Appendix Figure S4

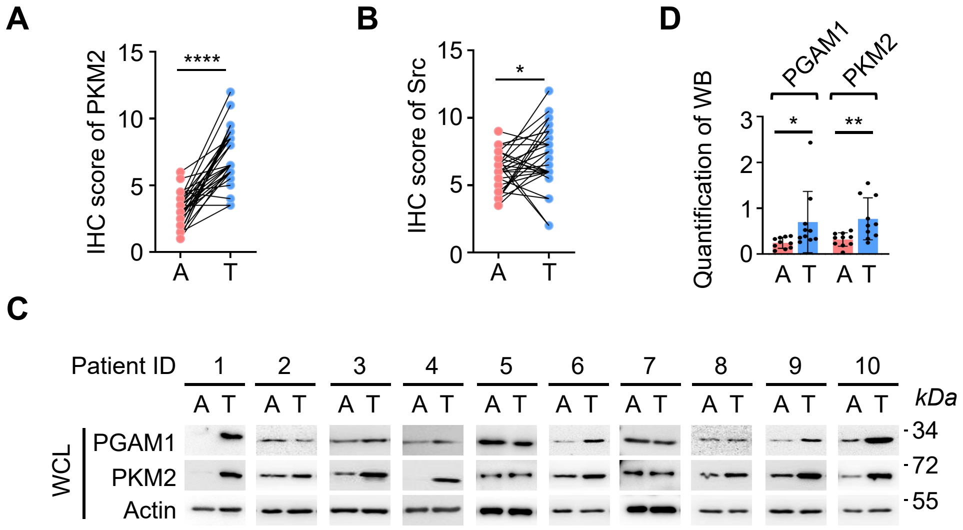

Appendix Figure S4. PGAM1, PKM2 and Src are highly expressed in tumor tissues.

(A, B) PKM2 and Src levels in tumor tissues are higher than that in adjacent normal tissues. The levels of PKM2 and Src from Fig. 6A were scored and subjected to statistical analysis (n = 30 pairs of adjacent and tumor tissues).

(C, D) PKM2 and PGAM1 levels in tumor tissues are higher than that in adjacent normal tissues. (C) The whole cell lysates from tumor tissues and adjacent normal tissues were analyzed by WB. (D) Quantification of WB. Relative levels of PGAM1 or PKM2 were normalized to that of Actin for each group (n = 10 pairs of adjacent and tumor tissues).

Data information: data represents mean ± SD (n ≥ 10) with significance determined by student's *t*-test (A, B, D); ****P<0.0001, **P<0.01, *P<0.05.

Appendix Figure S5

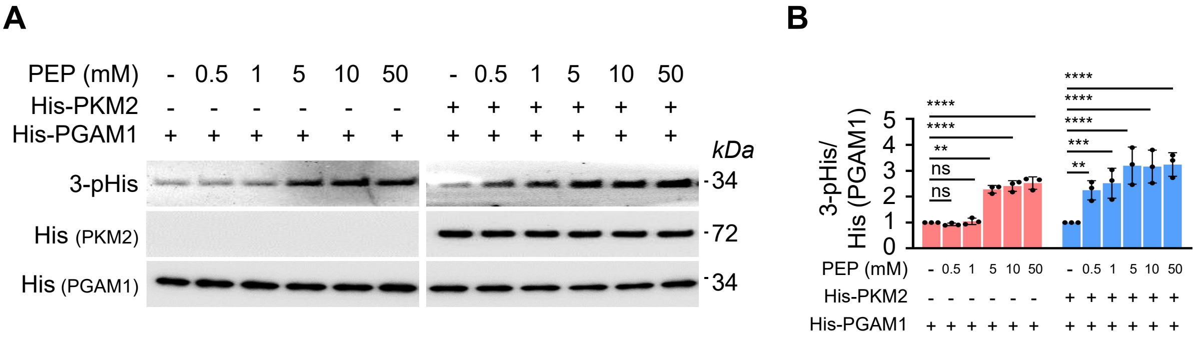

Appendix Figure S5. The impact of PEP levels on PGAM1 H11 phosphorylation.

(A, B) *In vitro* kinase assay was carried out with recombinant GST-PKM2 and His-PGAM1 in the different levels of PEP. (A) PGAM1 H11 phosphorylation was detected by WB. One representative experiment out of three was shown. (B) Quantification of WB. Relative levels of 3-pHis were normalized to that of His (PGAM1) for each group. Data represents mean ± SD (n = 3) with significance determined by one-way ANOVA test; ****P<0.0001, ***P<0.001. **P<0.01.
