# Supplementary figures and images for: PKM2 functions as a histidine kinase to phosphorylate PGAM1 and increase glycolysis shunts in cancer (part 1 of 3)
Source: EMBO J. 2024 May 15;43(12):5. doi: 10.1038/s44318-024-00110-8 (PMC11183095; doi:10.1038/s44318-024-00110-8)

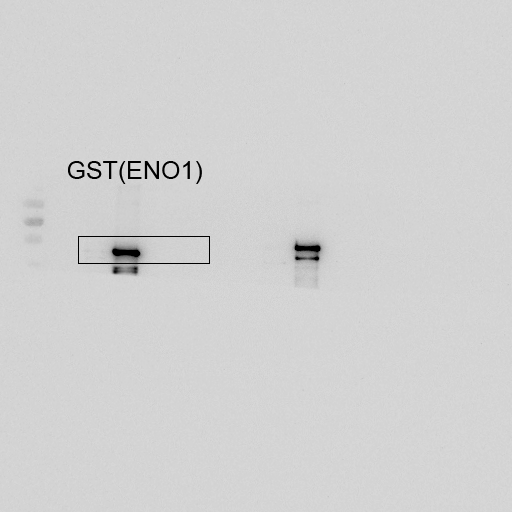

Supplement: Supplementary file 3 — Source data Fig. 1 [file 44318_2024_110_MOESM3_ESM.zip › Figure 1/1B-D/1C-GST (ENO1).tif]

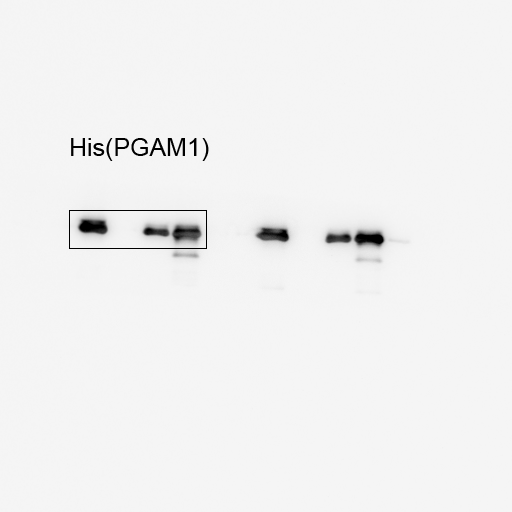

Supplement: Supplementary file 3 — Source data Fig. 1 [file 44318_2024_110_MOESM3_ESM.zip › Figure 1/1B-D/1C-His (PGAM1).tif]

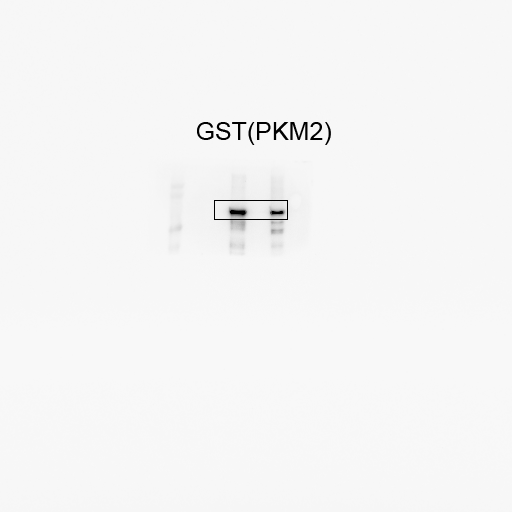

Supplement: Supplementary file 3 — Source data Fig. 1 [file 44318_2024_110_MOESM3_ESM.zip › Figure 1/1B-D/1B-GST (PKM2).tif]

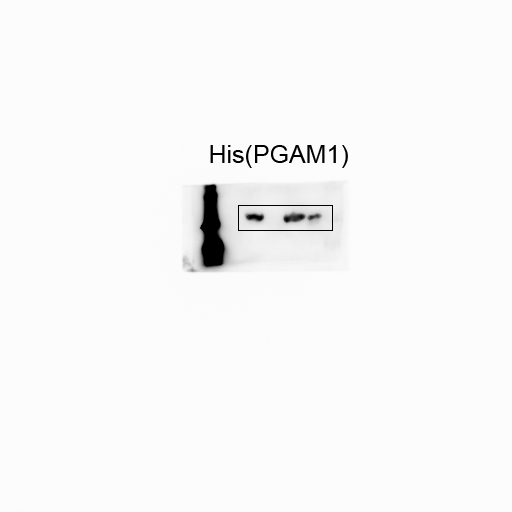

Supplement: Supplementary file 3 — Source data Fig. 1 [file 44318_2024_110_MOESM3_ESM.zip › Figure 1/1B-D/1B-His (PGAM1).tif]

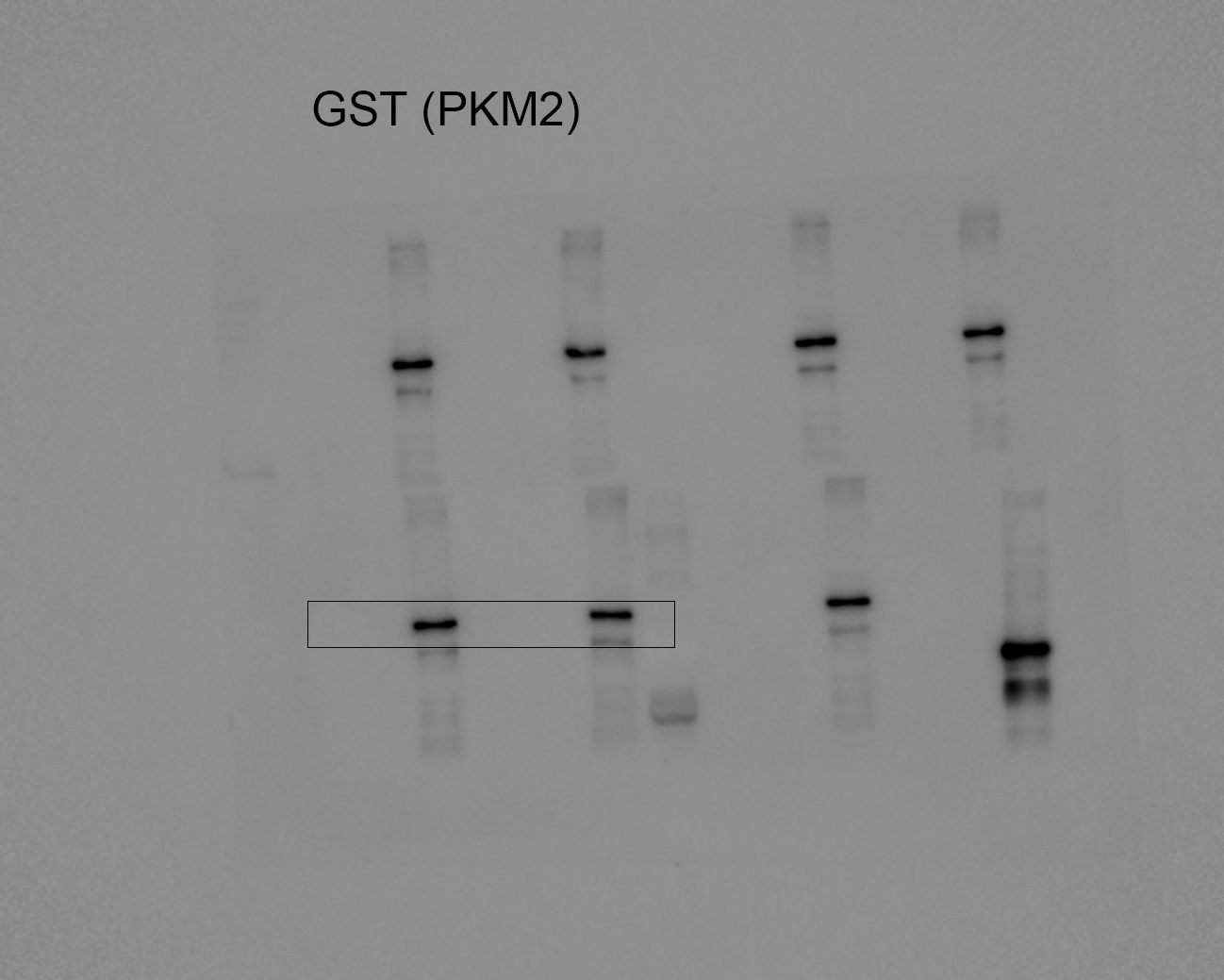

Supplement: Supplementary file 3 — Source data Fig. 1 [file 44318_2024_110_MOESM3_ESM.zip › Figure 1/1H/5-GST (PKM2).tif]

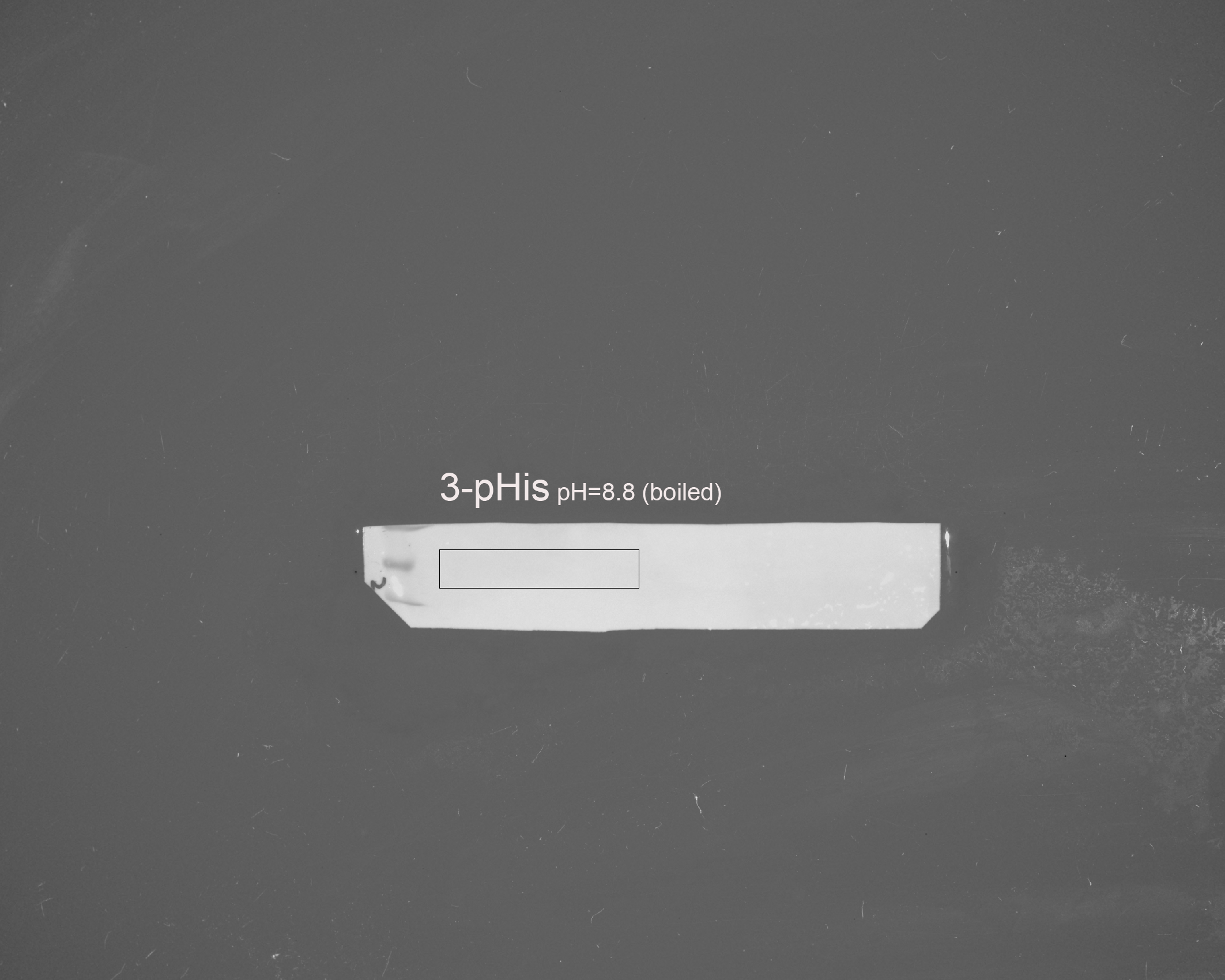

Supplement: Supplementary file 3 — Source data Fig. 1 [file 44318_2024_110_MOESM3_ESM.zip › Figure 1/1H/2-3-pHis pH = 8.8 (boiled).tif]

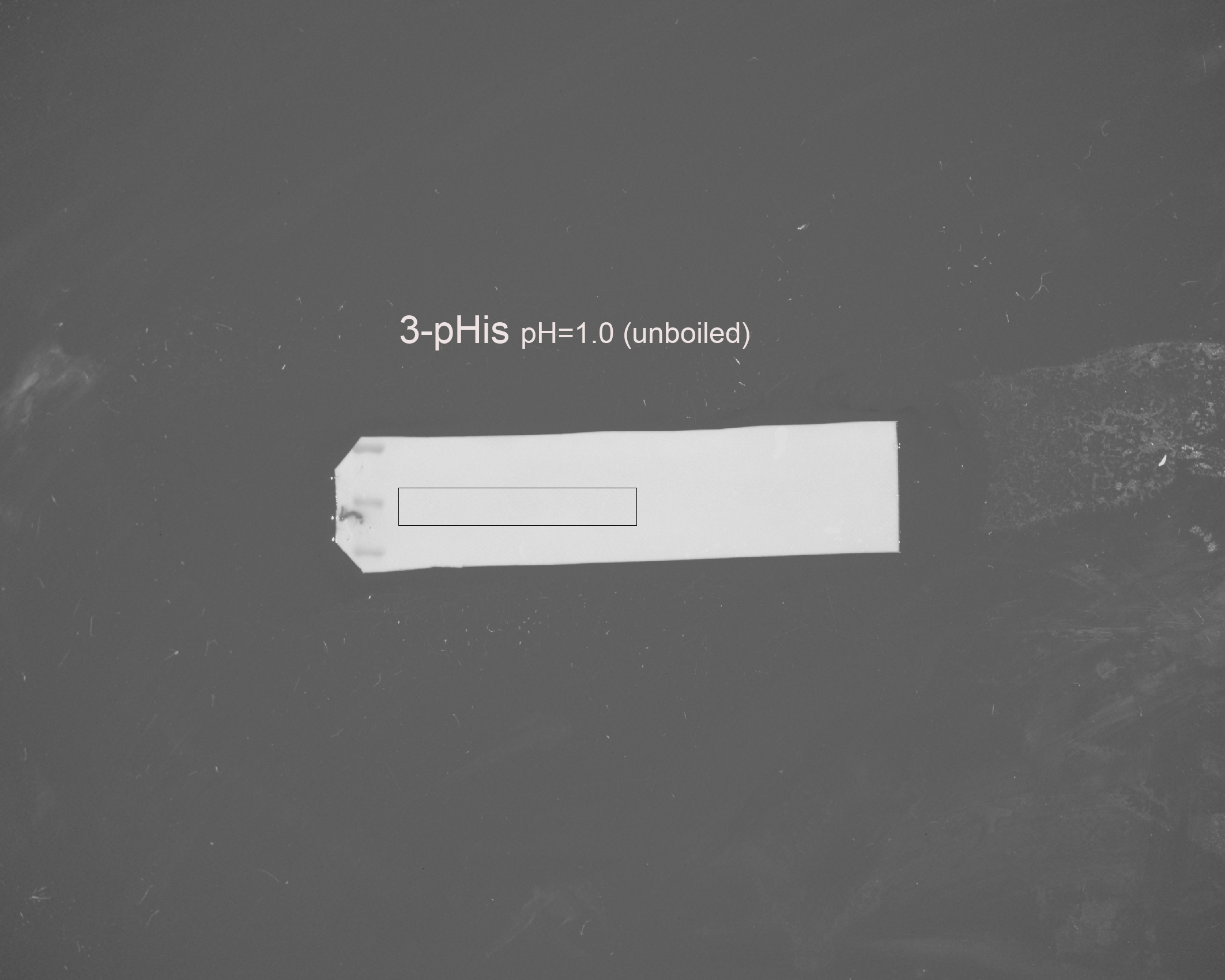

Supplement: Supplementary file 3 — Source data Fig. 1 [file 44318_2024_110_MOESM3_ESM.zip › Figure 1/1H/3-3-pHis pH = 1.0 (unboiled).tif]

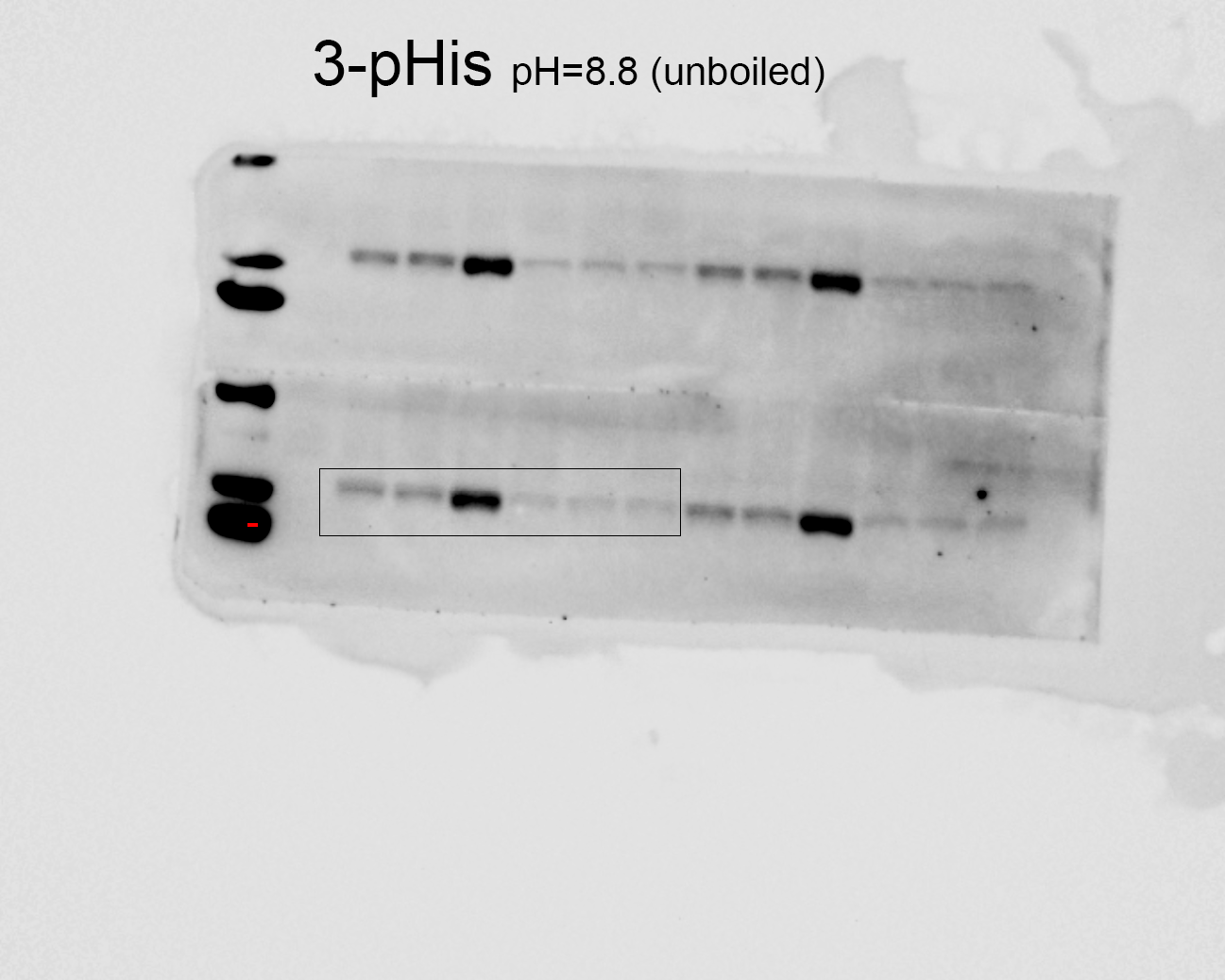

Supplement: Supplementary file 3 — Source data Fig. 1 [file 44318_2024_110_MOESM3_ESM.zip › Figure 1/1H/1-3-pHis pH = 8.8 (unboiled).tif]

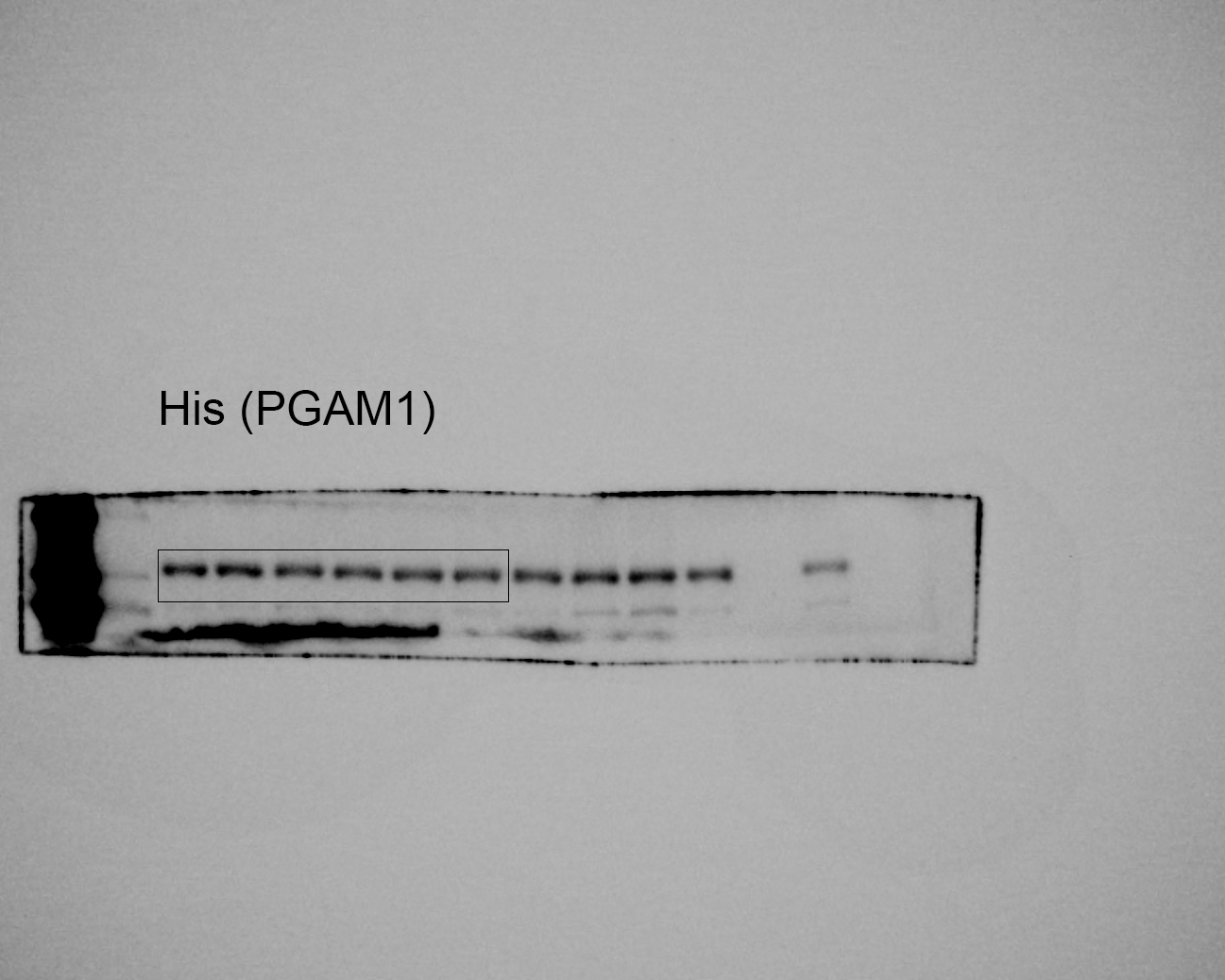

Supplement: Supplementary file 3 — Source data Fig. 1 [file 44318_2024_110_MOESM3_ESM.zip › Figure 1/1H/4-His (PGAM1).tif]

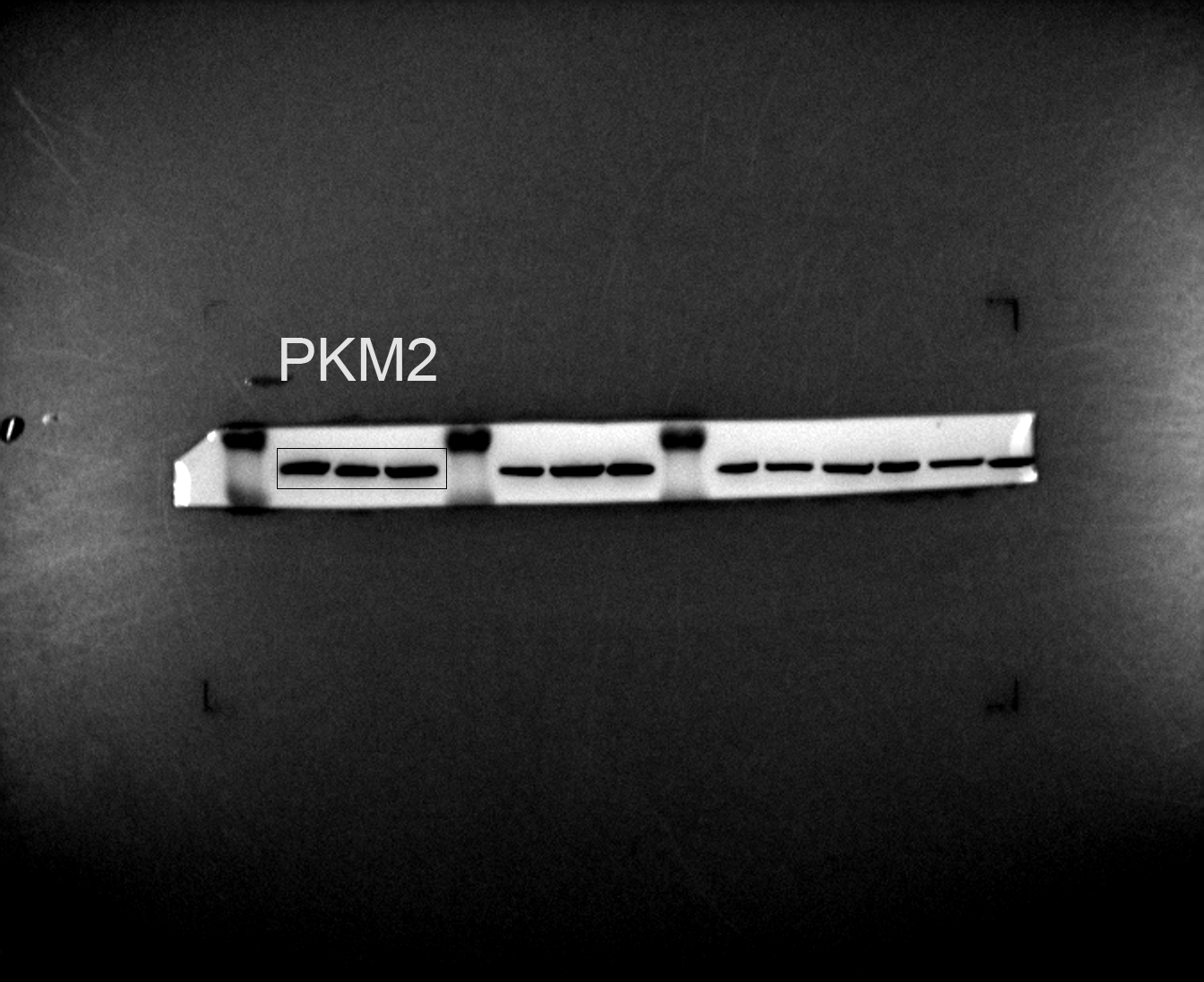

Supplement: Supplementary file 3 — Source data Fig. 1 [file 44318_2024_110_MOESM3_ESM.zip › Figure 1/1F/4-PKM2.Tif]

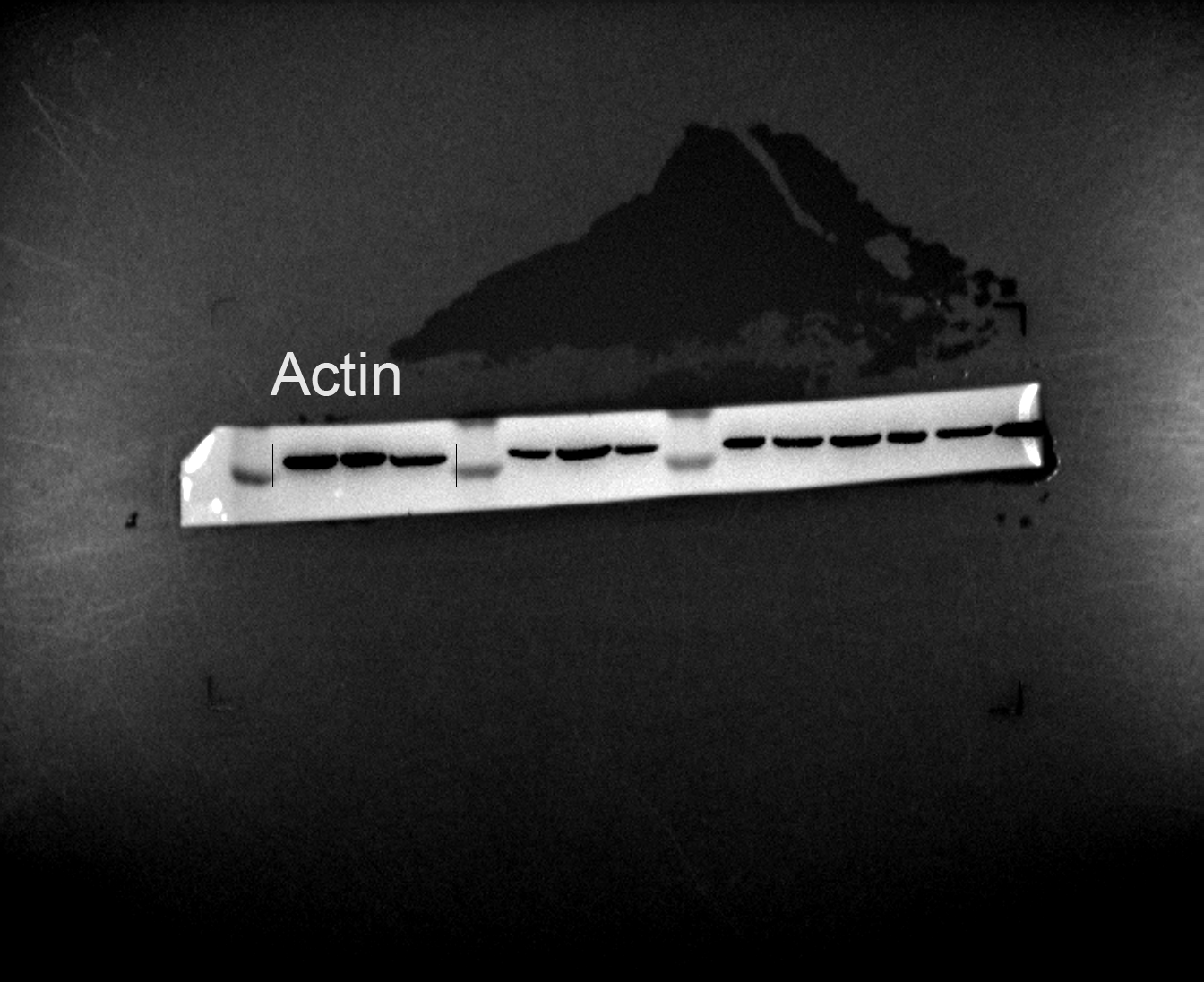

Supplement: Supplementary file 3 — Source data Fig. 1 [file 44318_2024_110_MOESM3_ESM.zip › Figure 1/1F/6-Actin.Tif]

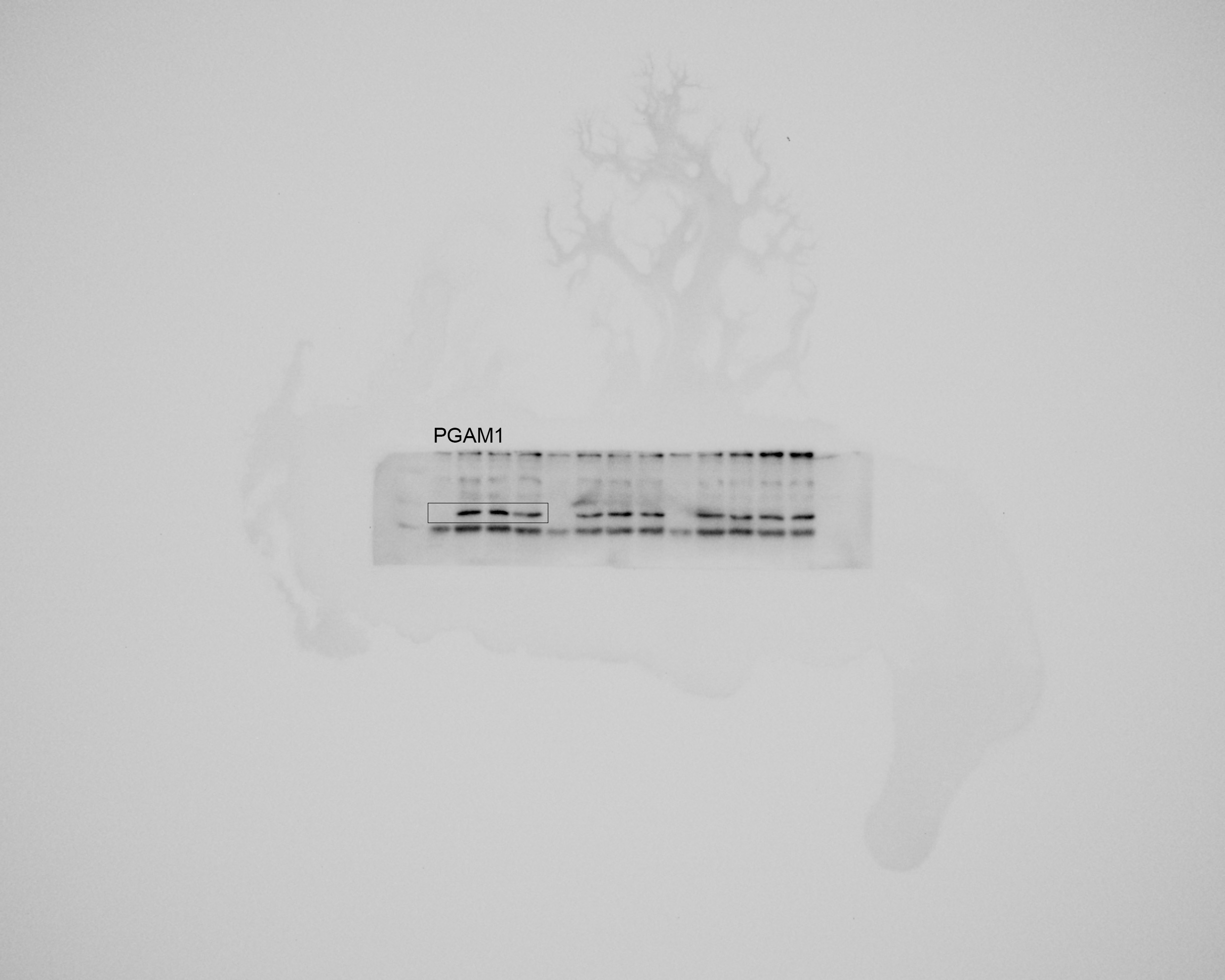

Supplement: Supplementary file 3 — Source data Fig. 1 [file 44318_2024_110_MOESM3_ESM.zip › Figure 1/1F/3-PGAM1.tif]

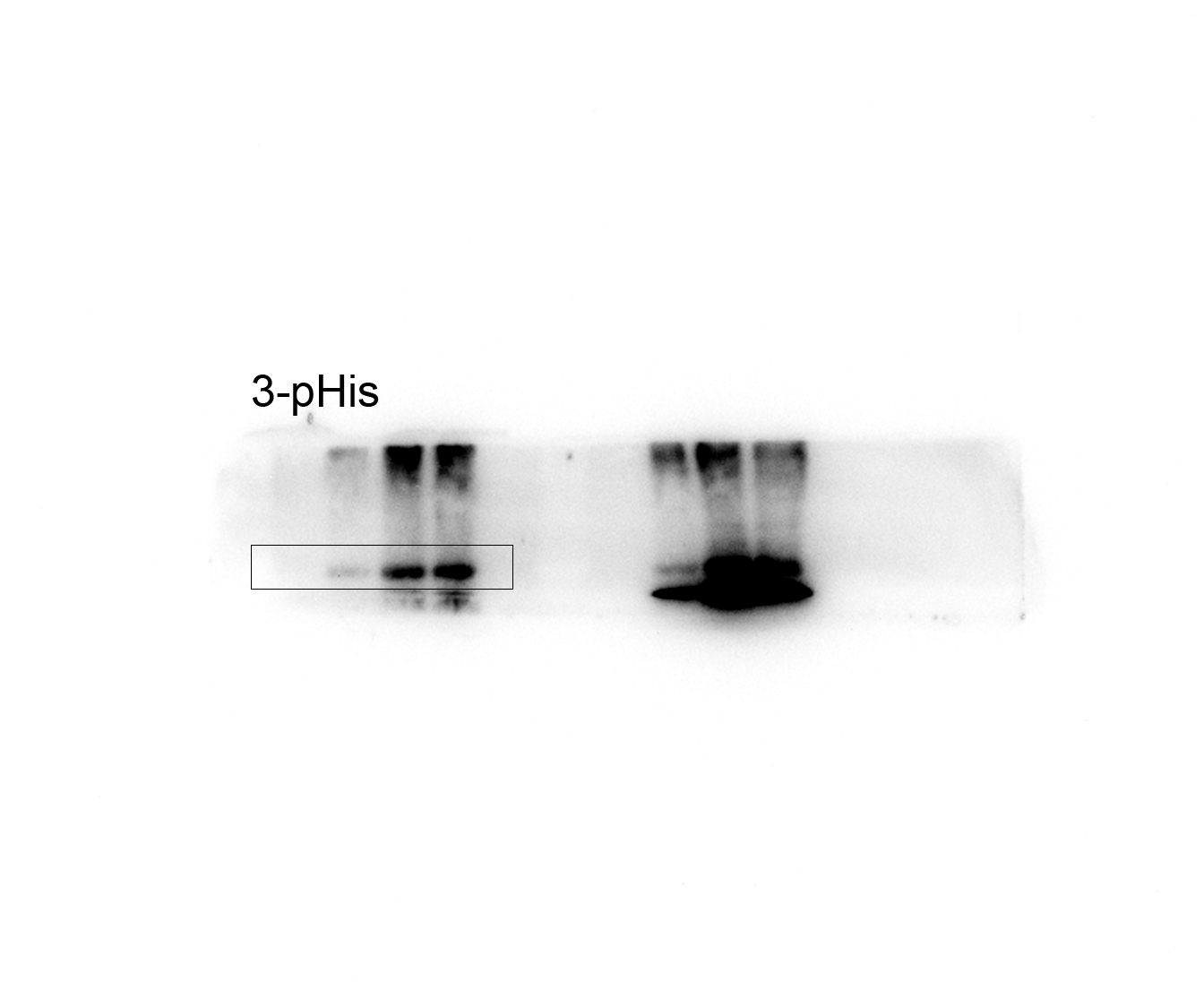

Supplement: Supplementary file 3 — Source data Fig. 1 [file 44318_2024_110_MOESM3_ESM.zip › Figure 1/1F/2-3-pHis.Tif]

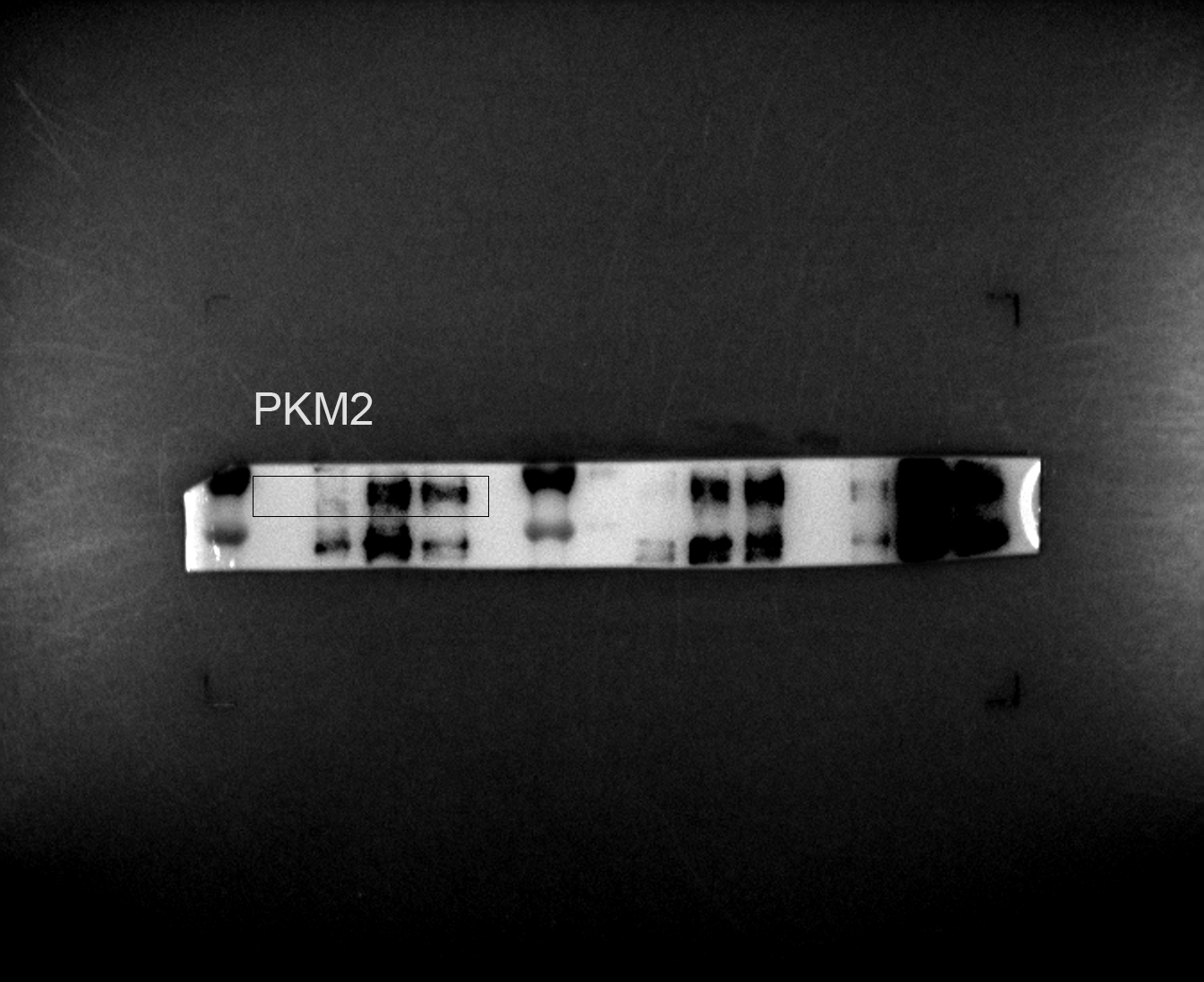

Supplement: Supplementary file 3 — Source data Fig. 1 [file 44318_2024_110_MOESM3_ESM.zip › Figure 1/1F/1-PKM2.Tif]

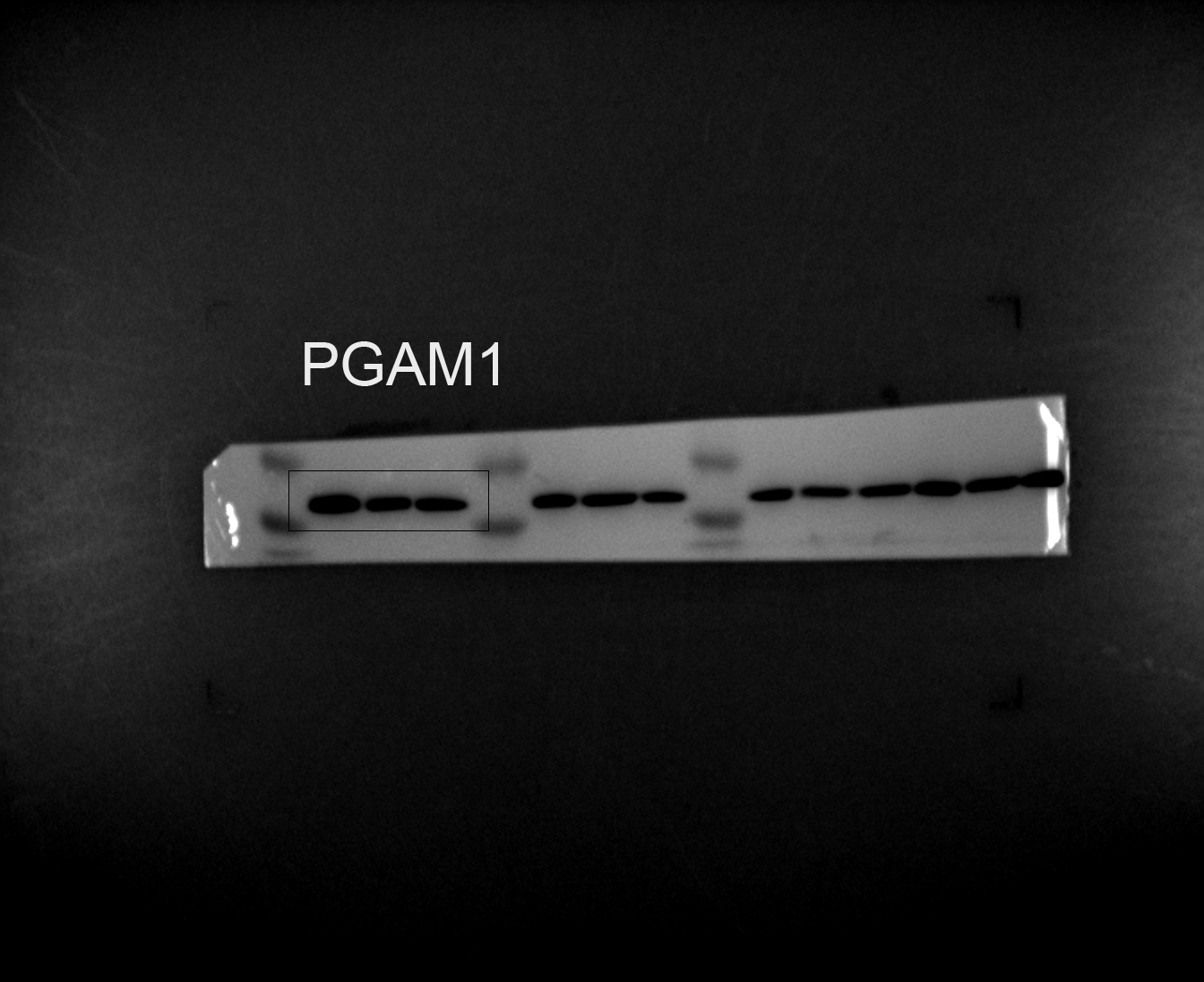

Supplement: Supplementary file 3 — Source data Fig. 1 [file 44318_2024_110_MOESM3_ESM.zip › Figure 1/1F/5-PGAM1.Tif]

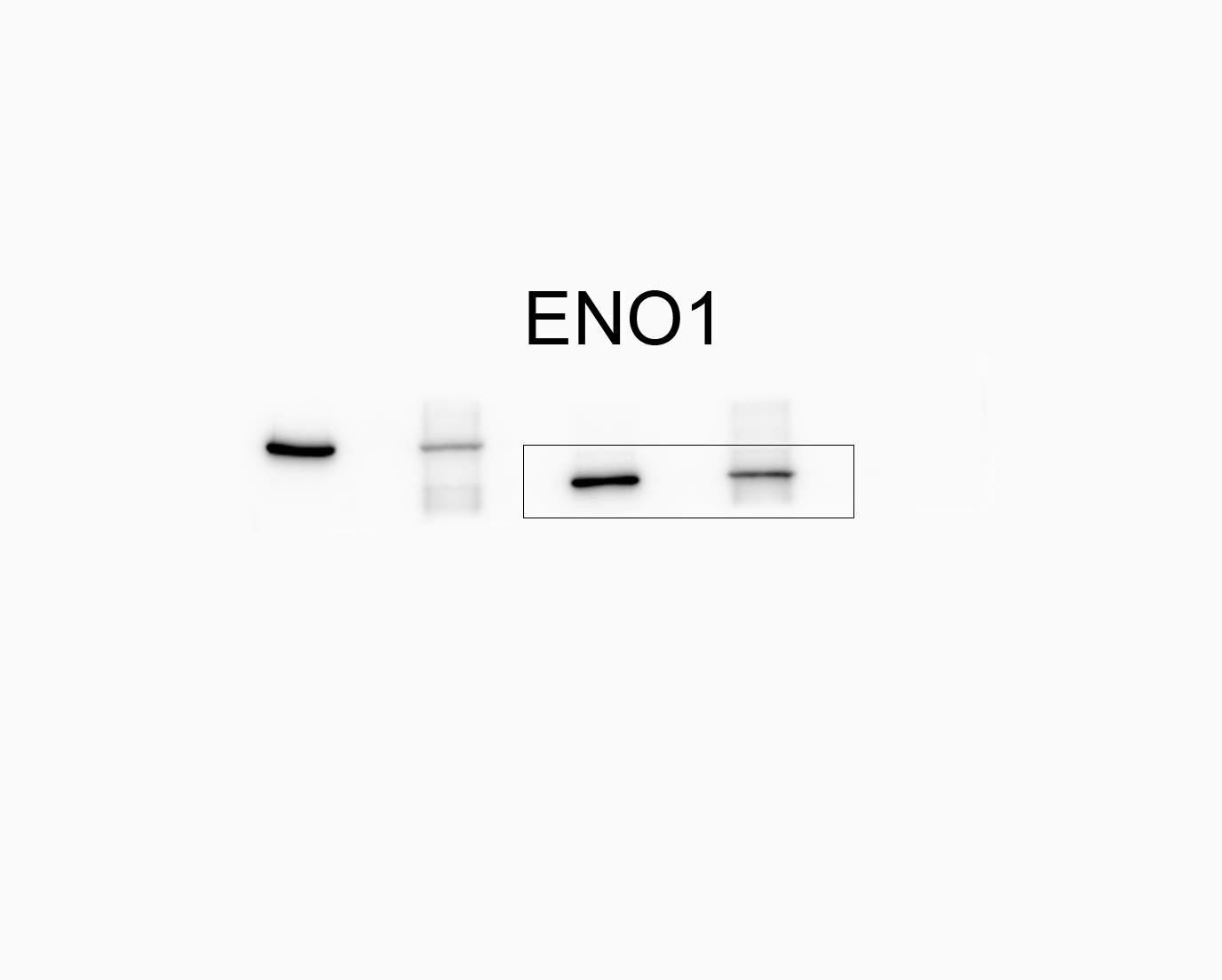

Supplement: Supplementary file 3 — Source data Fig. 1 [file 44318_2024_110_MOESM3_ESM.zip › Figure 1/1A/2-ENO1.tif]

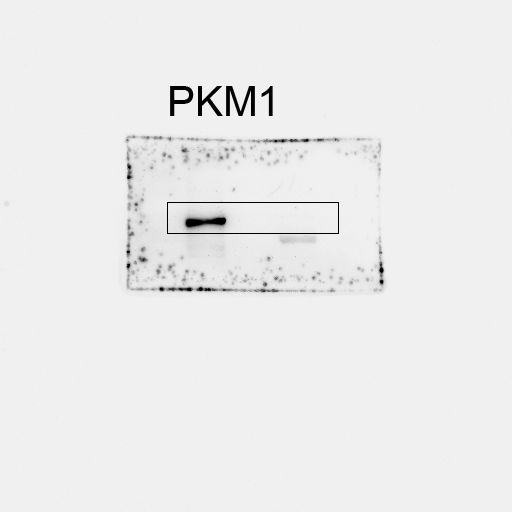

Supplement: Supplementary file 3 — Source data Fig. 1 [file 44318_2024_110_MOESM3_ESM.zip › Figure 1/1A/3-PKM1.tif]

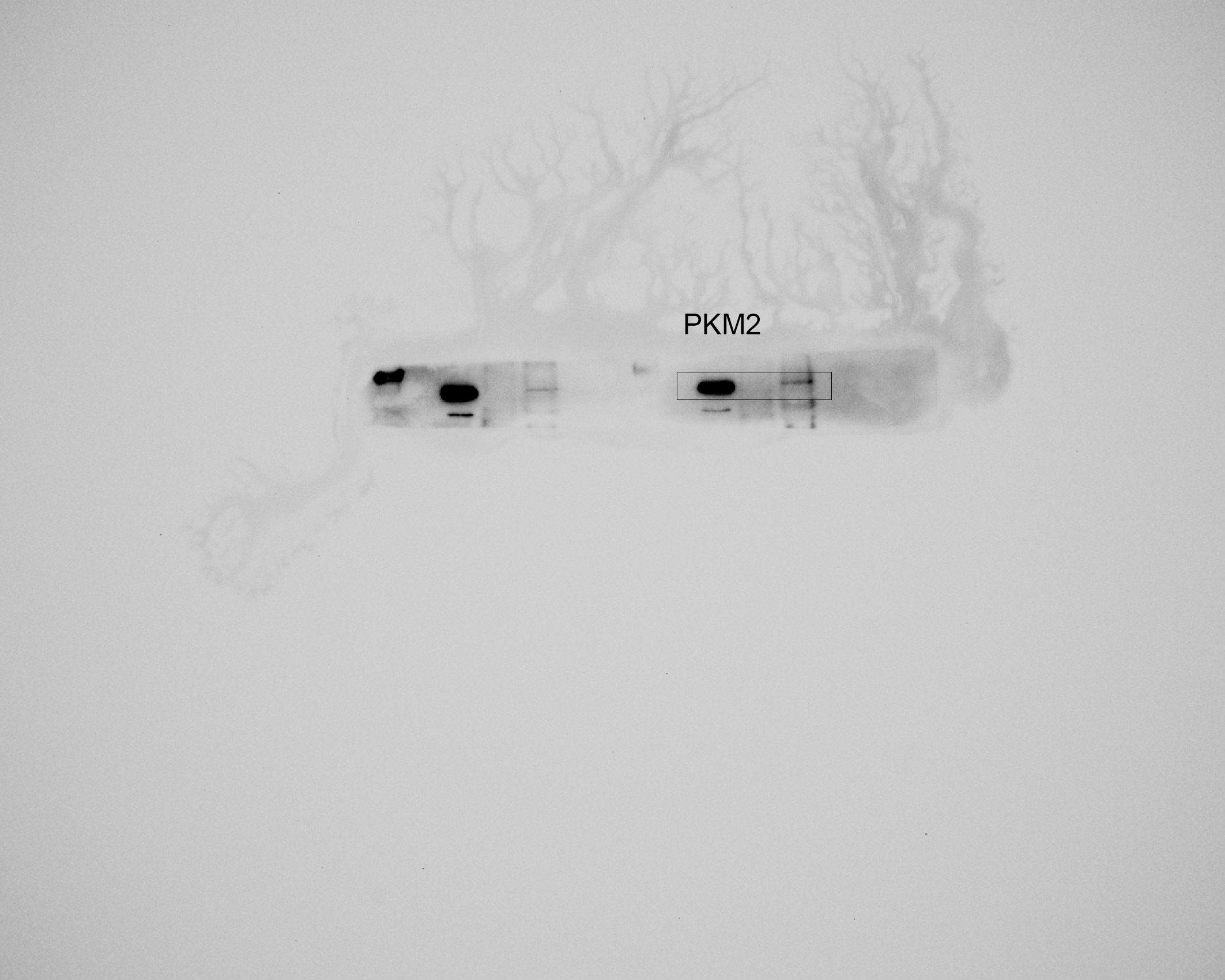

Supplement: Supplementary file 3 — Source data Fig. 1 [file 44318_2024_110_MOESM3_ESM.zip › Figure 1/1A/1-PKM2.tif]

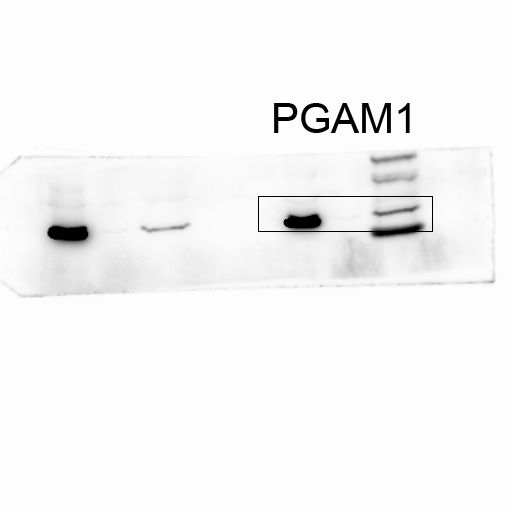

Supplement: Supplementary file 3 — Source data Fig. 1 [file 44318_2024_110_MOESM3_ESM.zip › Figure 1/1A/5-PGAM1.tif]

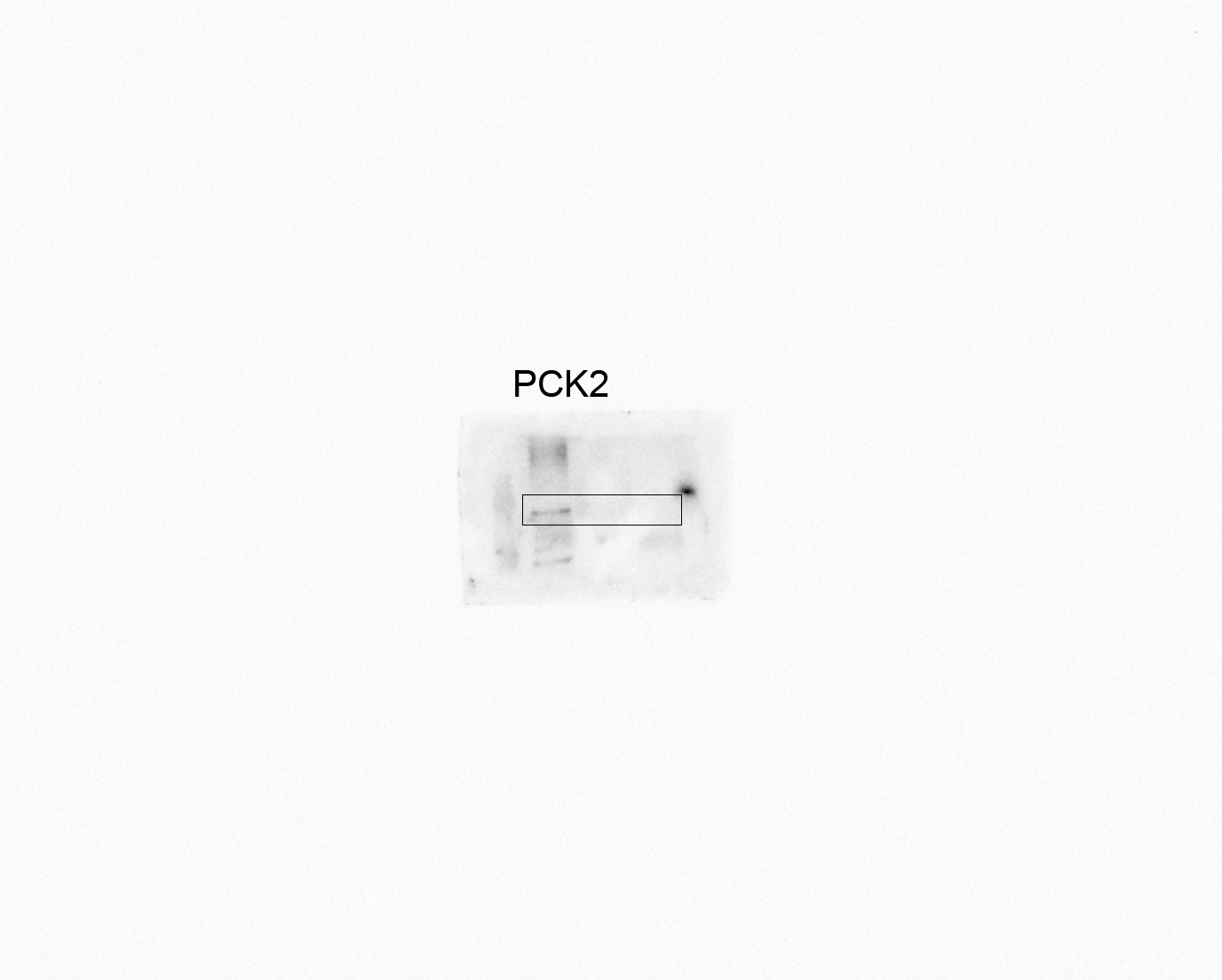

Supplement: Supplementary file 3 — Source data Fig. 1 [file 44318_2024_110_MOESM3_ESM.zip › Figure 1/1A/4-PCK2.Tif]

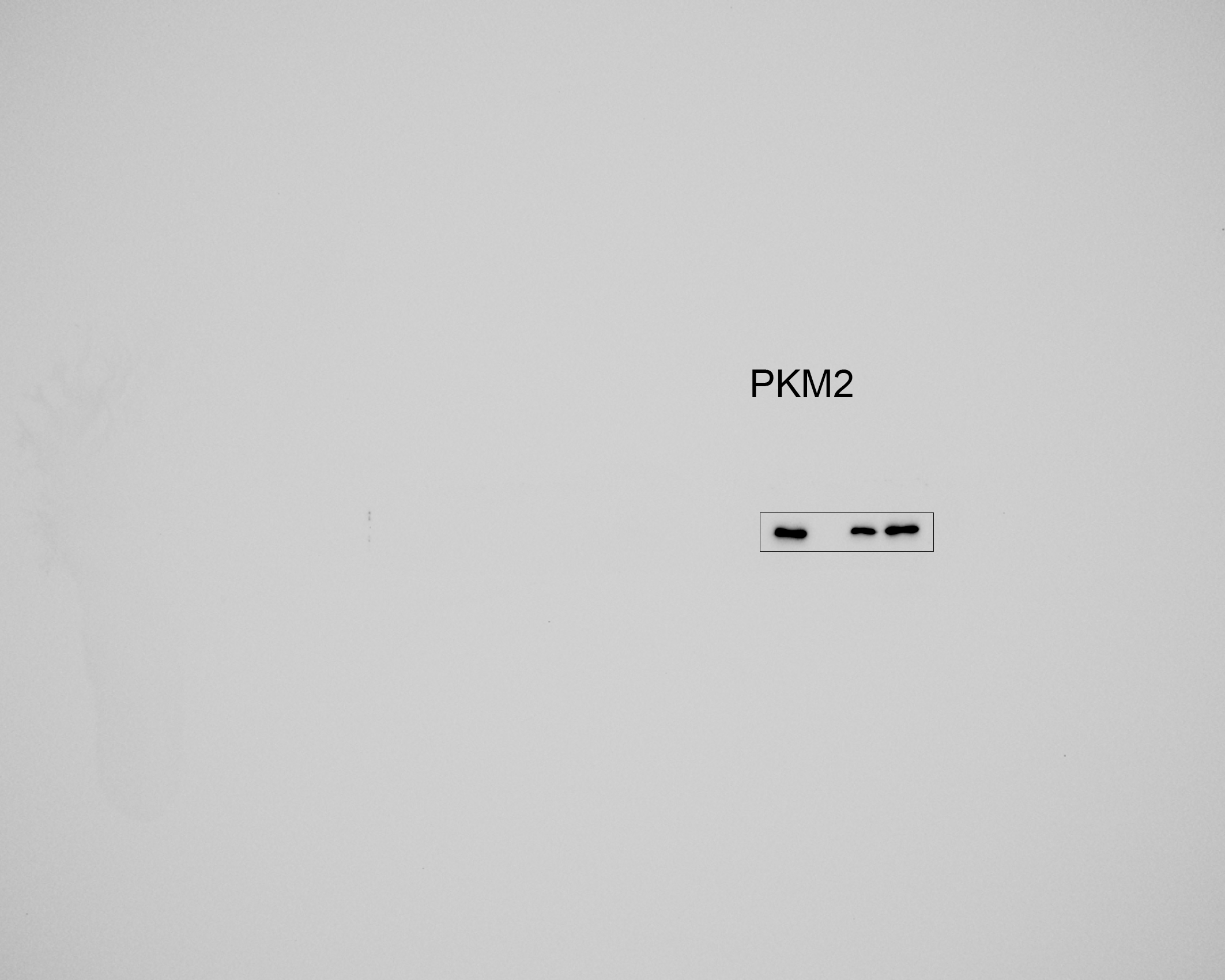

Supplement: Supplementary file 3 — Source data Fig. 1 [file 44318_2024_110_MOESM3_ESM.zip › Figure 1/1G/4-PKM2.tif]

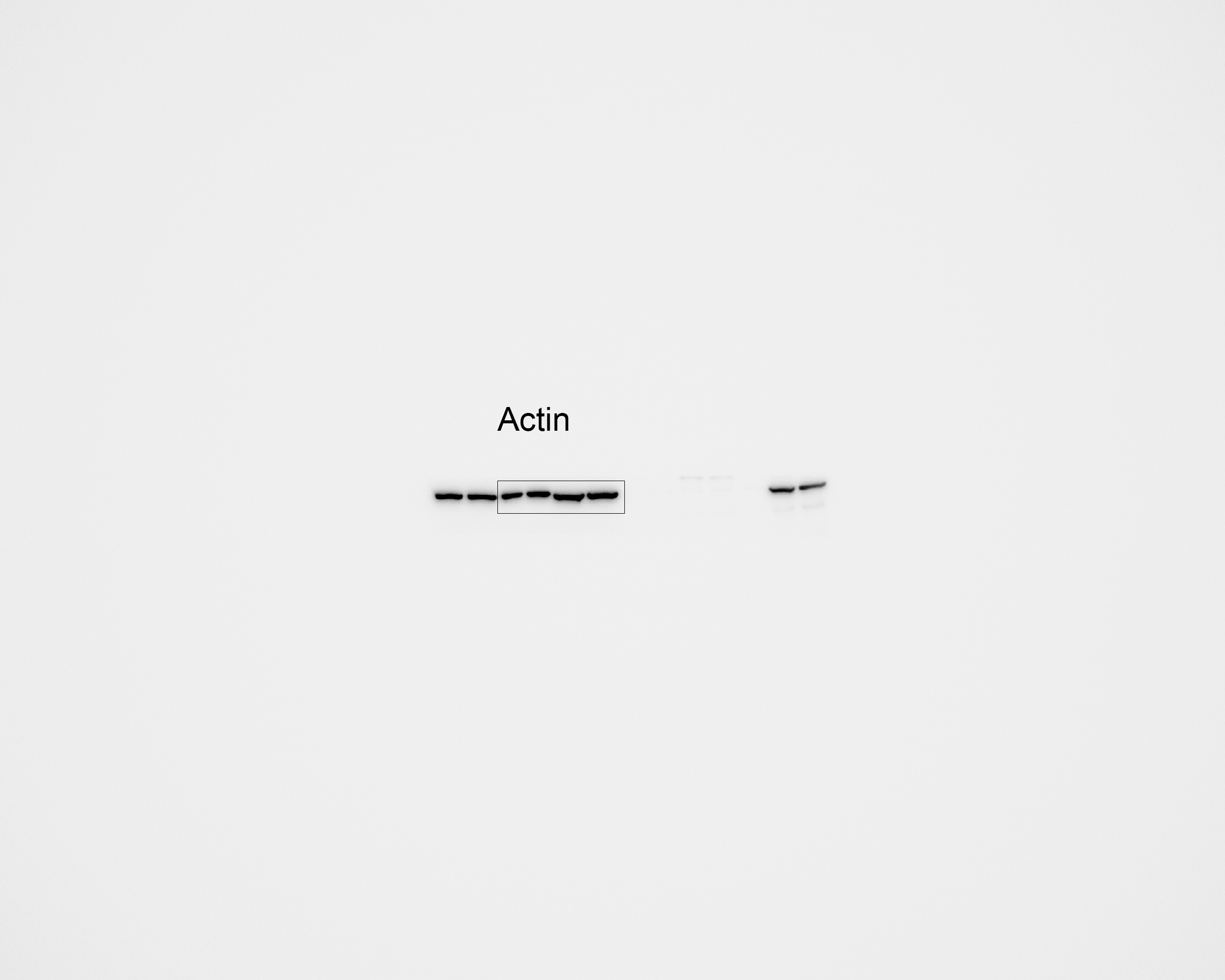

Supplement: Supplementary file 3 — Source data Fig. 1 [file 44318_2024_110_MOESM3_ESM.zip › Figure 1/1G/6-Actin.tif]

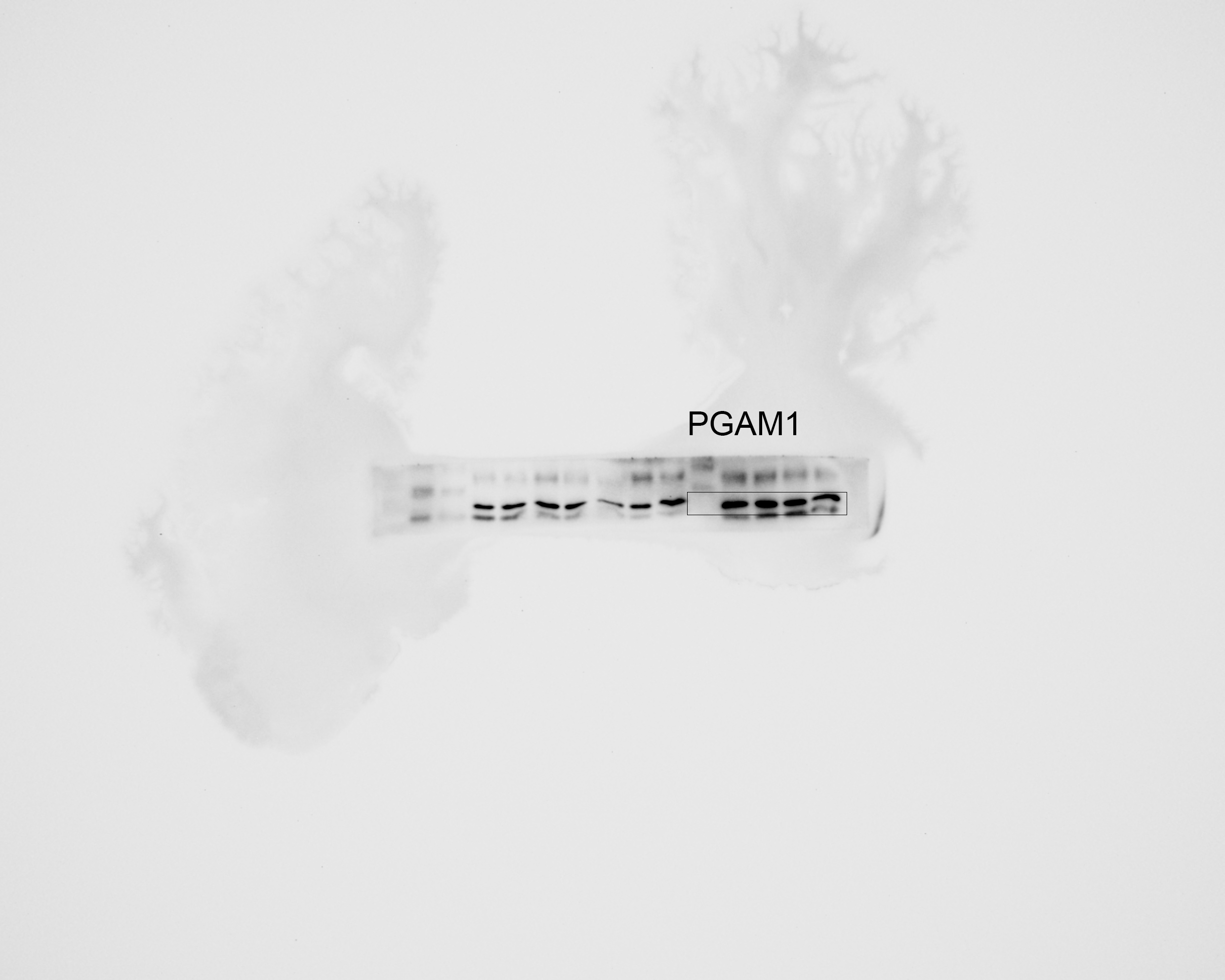

Supplement: Supplementary file 3 — Source data Fig. 1 [file 44318_2024_110_MOESM3_ESM.zip › Figure 1/1G/3-PGAM1.tif]

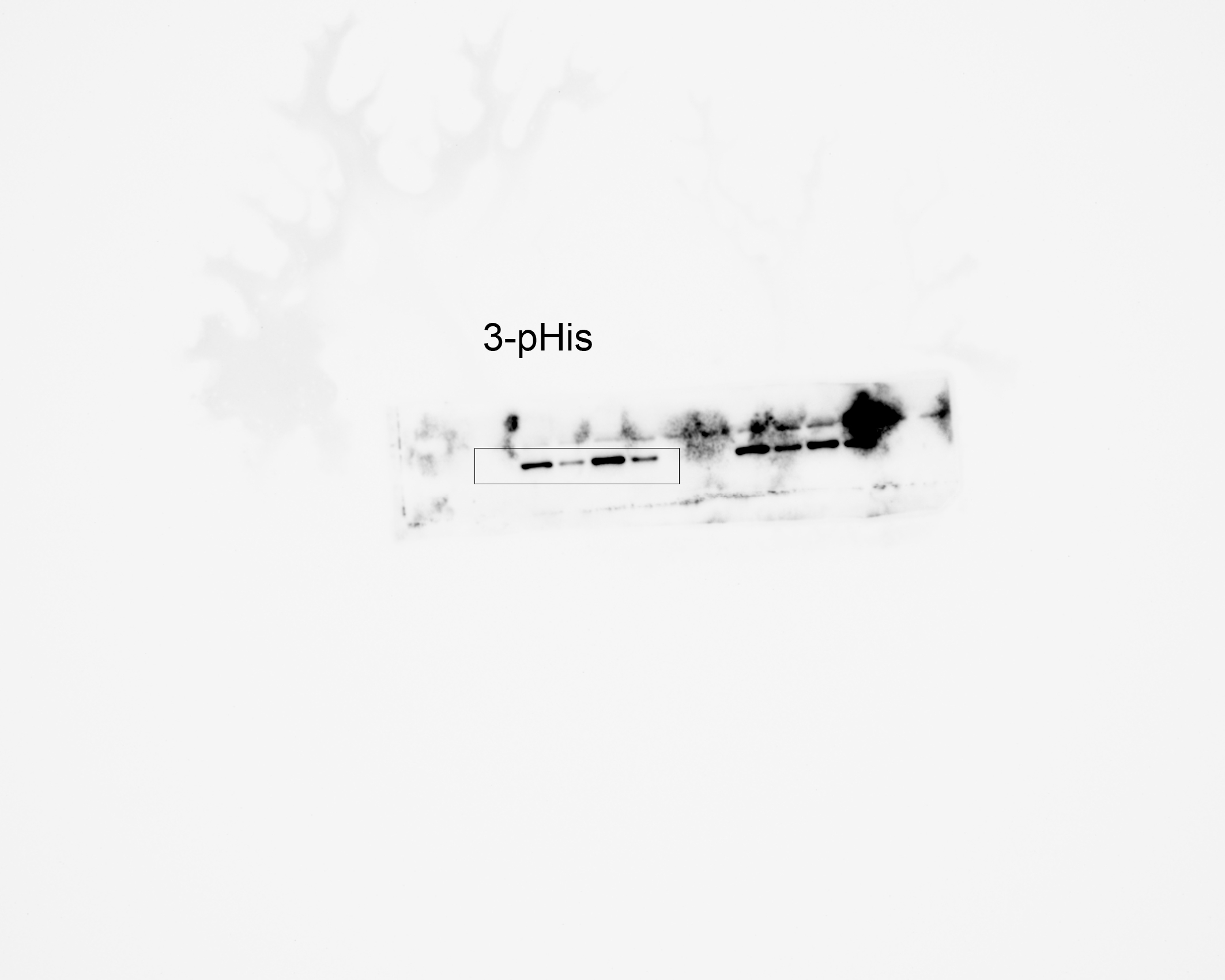

Supplement: Supplementary file 3 — Source data Fig. 1 [file 44318_2024_110_MOESM3_ESM.zip › Figure 1/1G/2-3-pHis.tif]

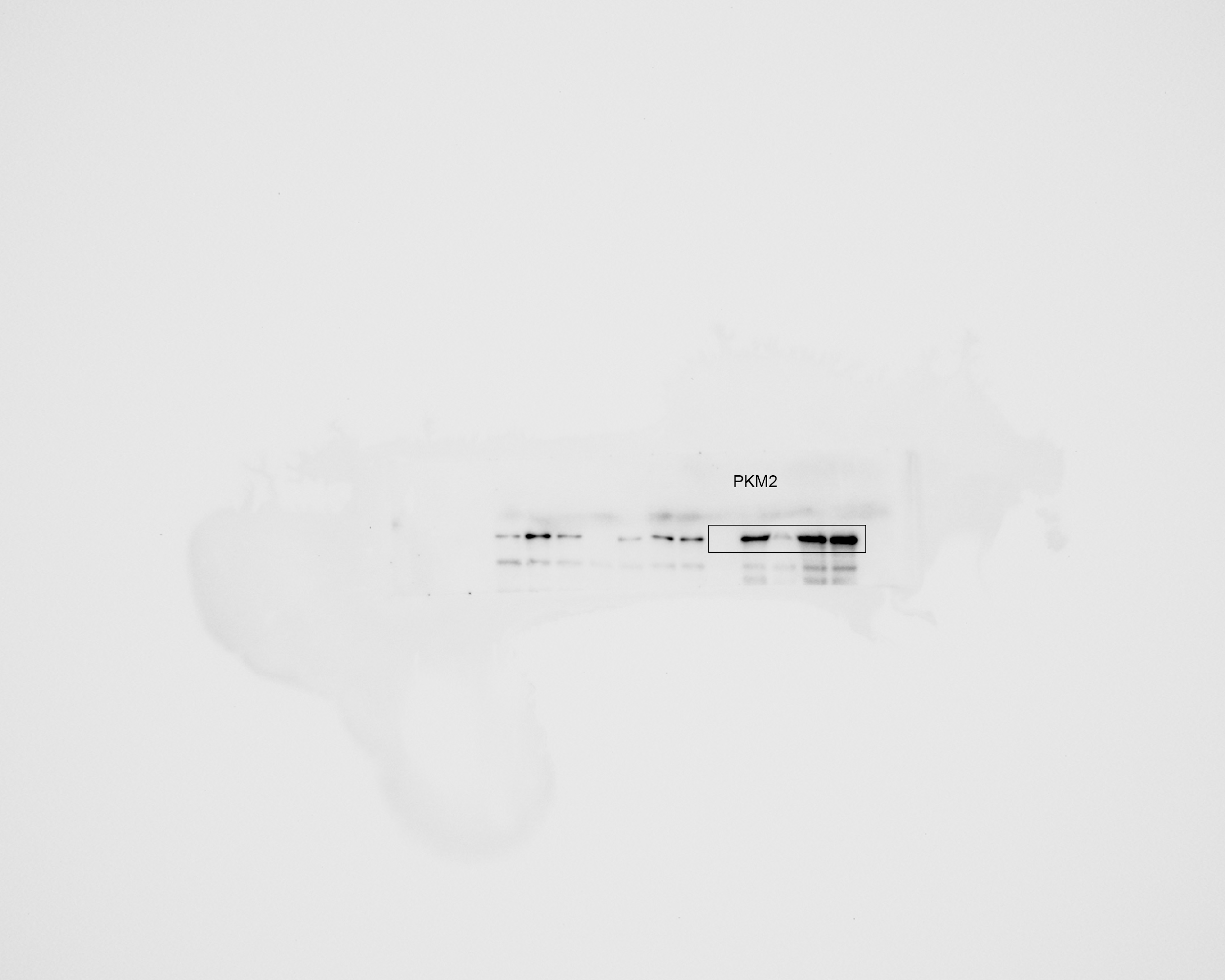

Supplement: Supplementary file 3 — Source data Fig. 1 [file 44318_2024_110_MOESM3_ESM.zip › Figure 1/1G/1-PKM2.tif]

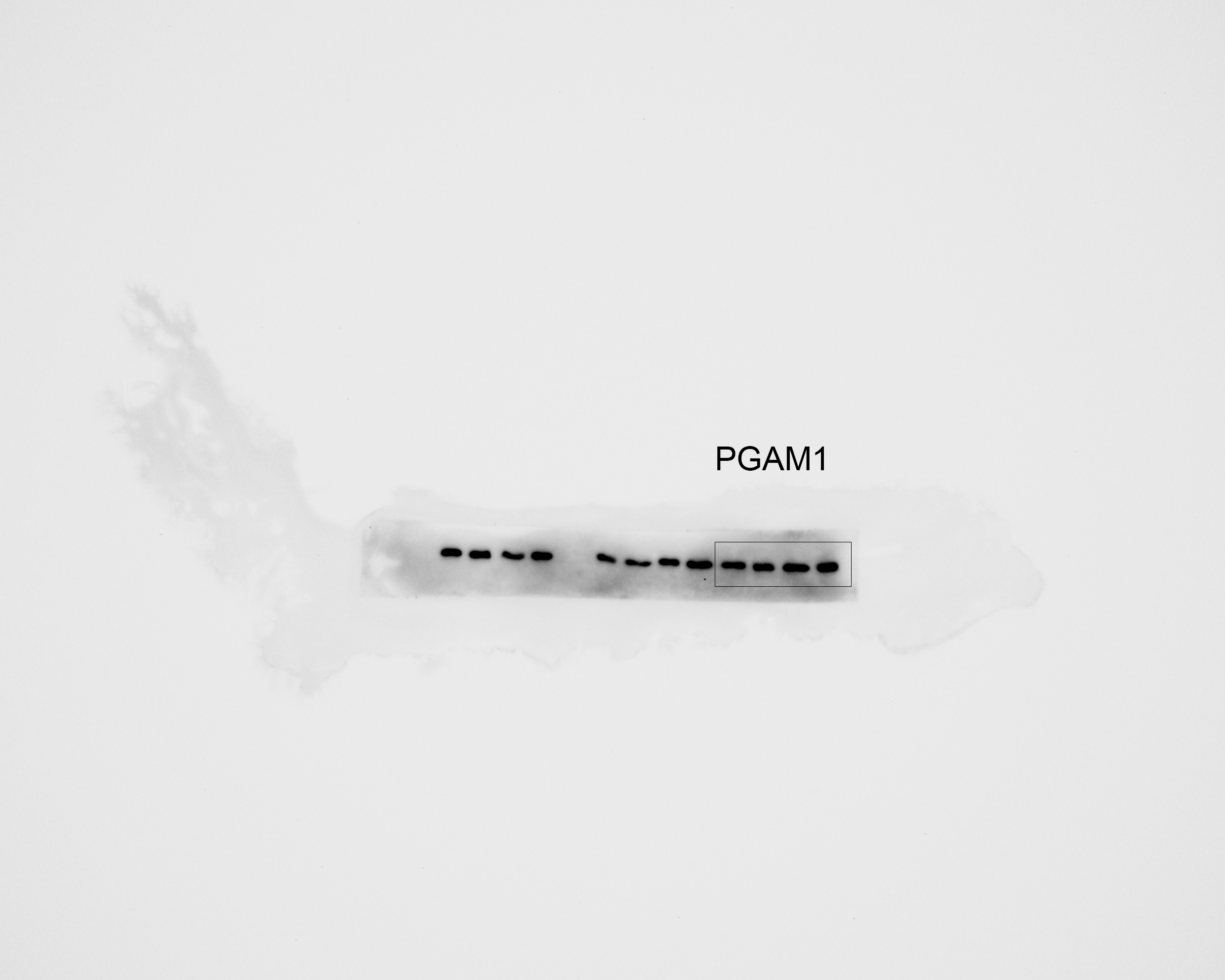

Supplement: Supplementary file 3 — Source data Fig. 1 [file 44318_2024_110_MOESM3_ESM.zip › Figure 1/1G/5-PGAM1.tif]

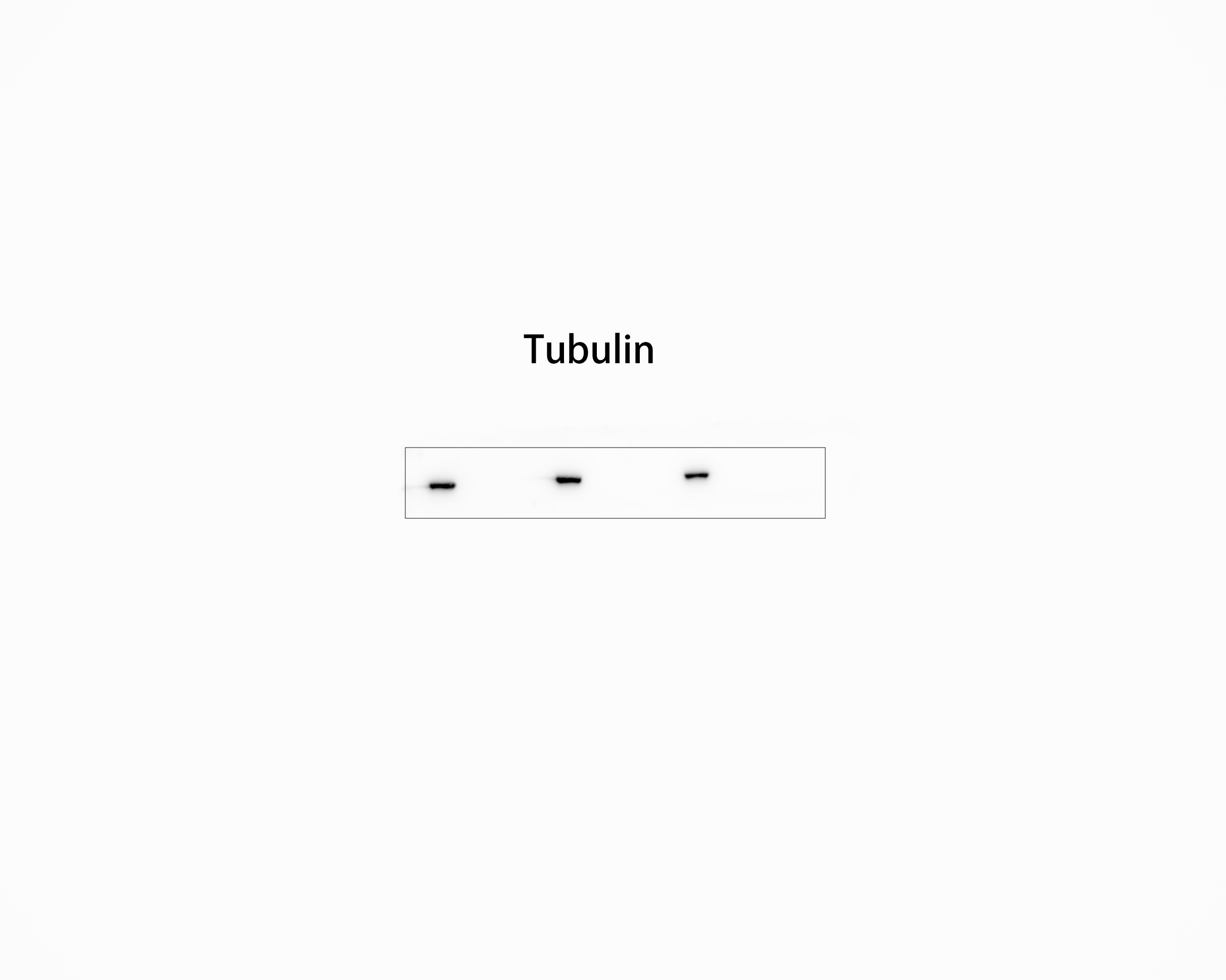

Supplement: Supplementary file 3 — Source data Fig. 1 [file 44318_2024_110_MOESM3_ESM.zip › Figure 1/1L/2-Tubulin.tif]

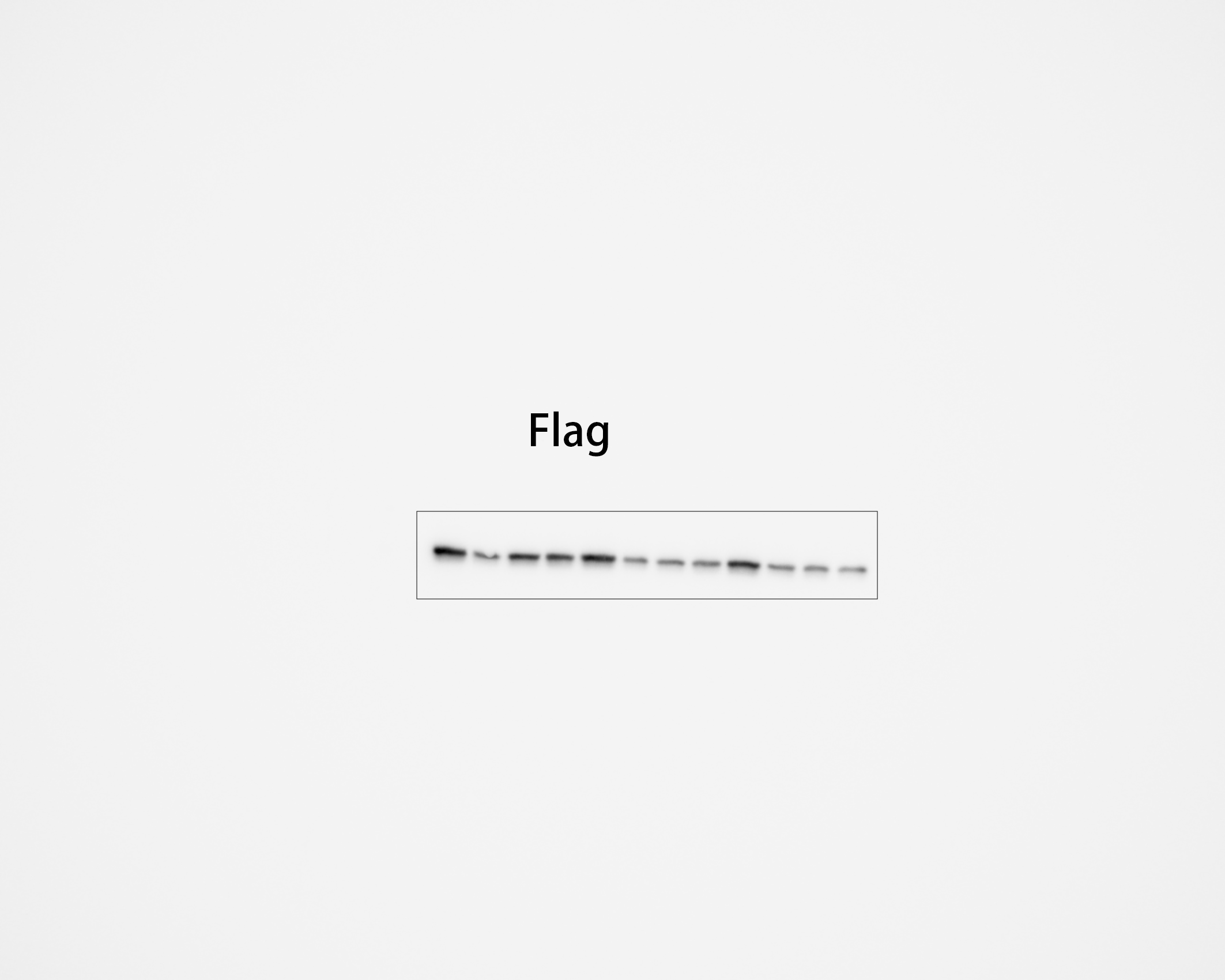

Supplement: Supplementary file 3 — Source data Fig. 1 [file 44318_2024_110_MOESM3_ESM.zip › Figure 1/1L/1-Flag.tif]

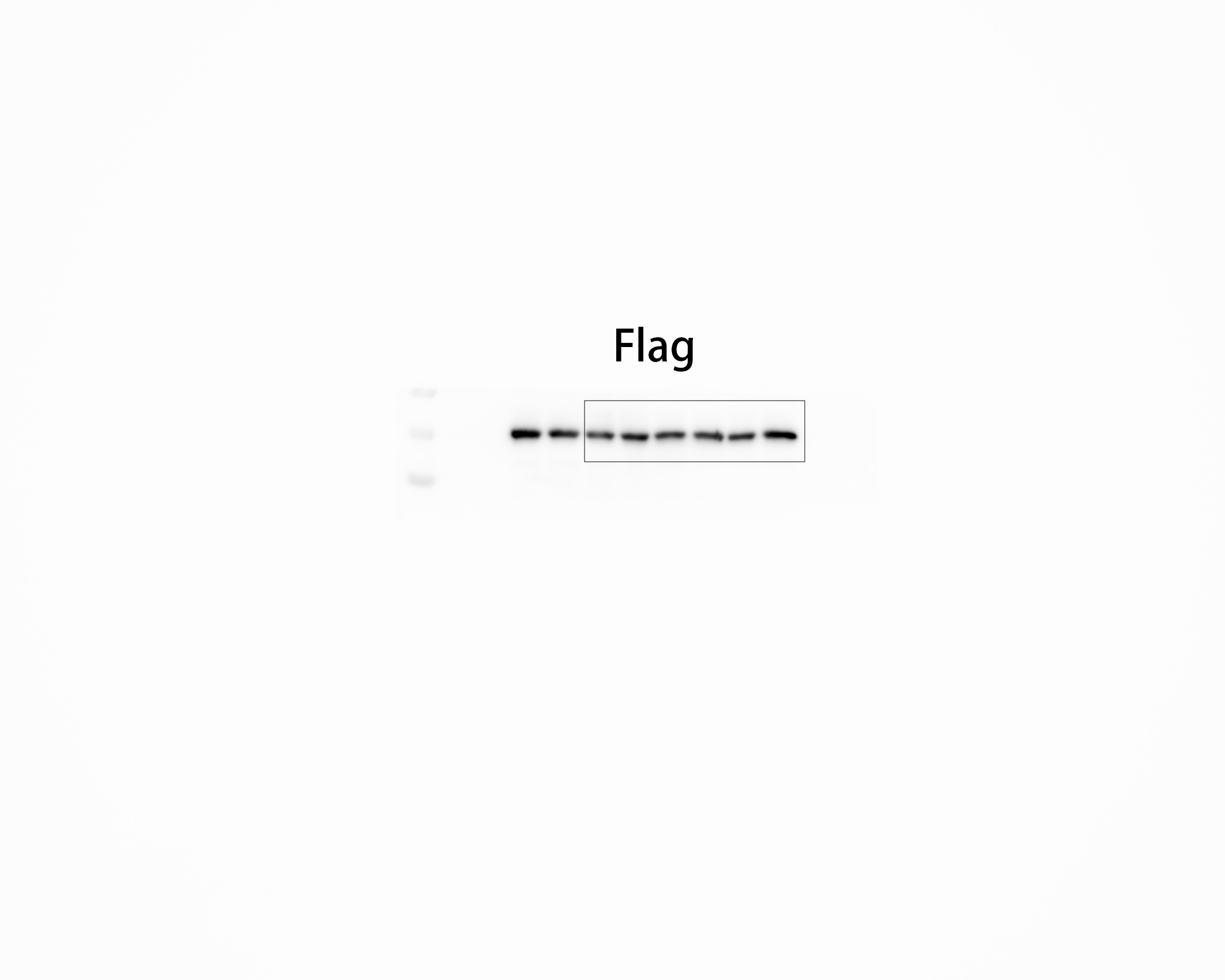

Supplement: Supplementary file 3 — Source data Fig. 1 [file 44318_2024_110_MOESM3_ESM.zip › Figure 1/1M/5-Flag.tif]

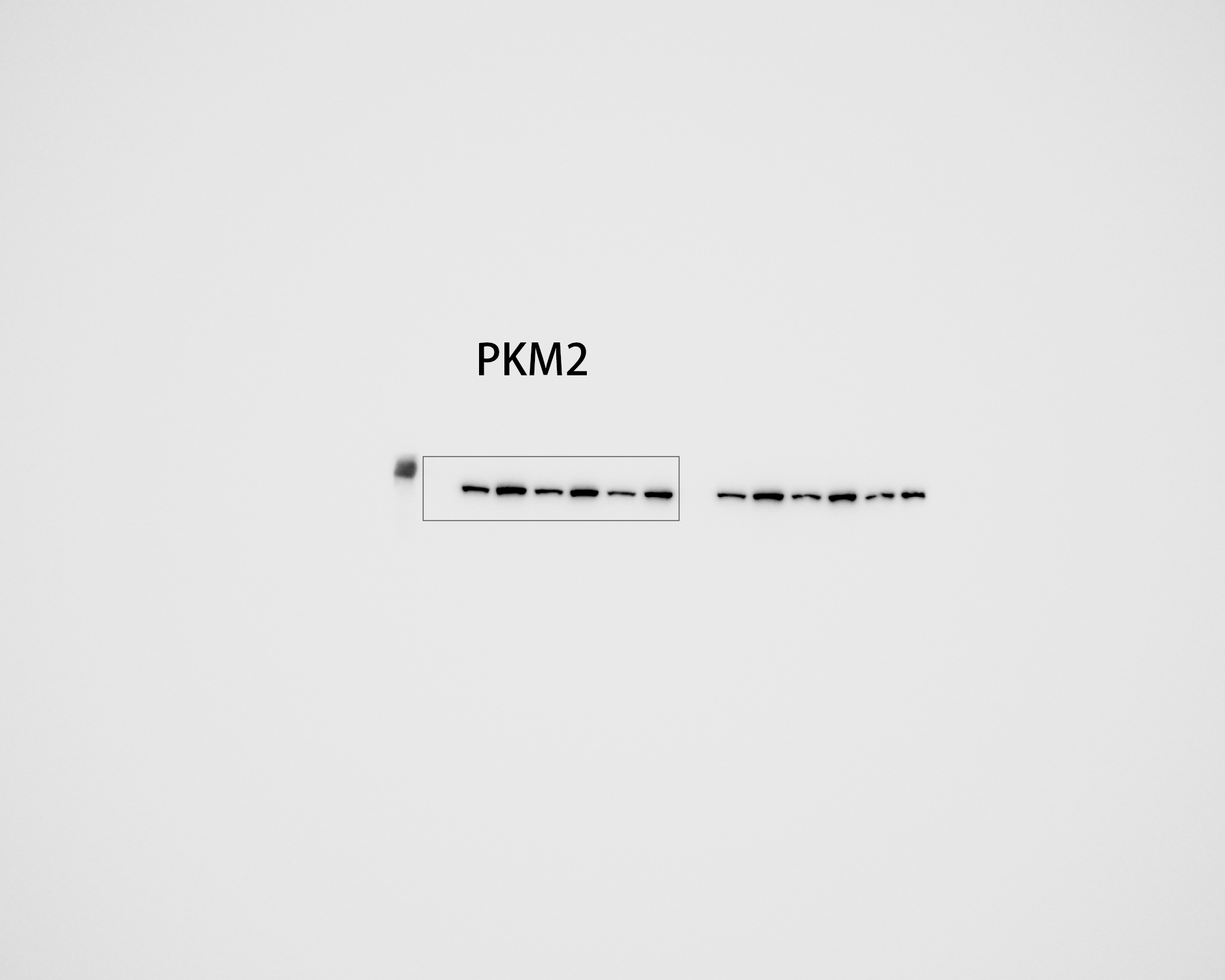

Supplement: Supplementary file 3 — Source data Fig. 1 [file 44318_2024_110_MOESM3_ESM.zip › Figure 1/1M/2-PKM2.tif]

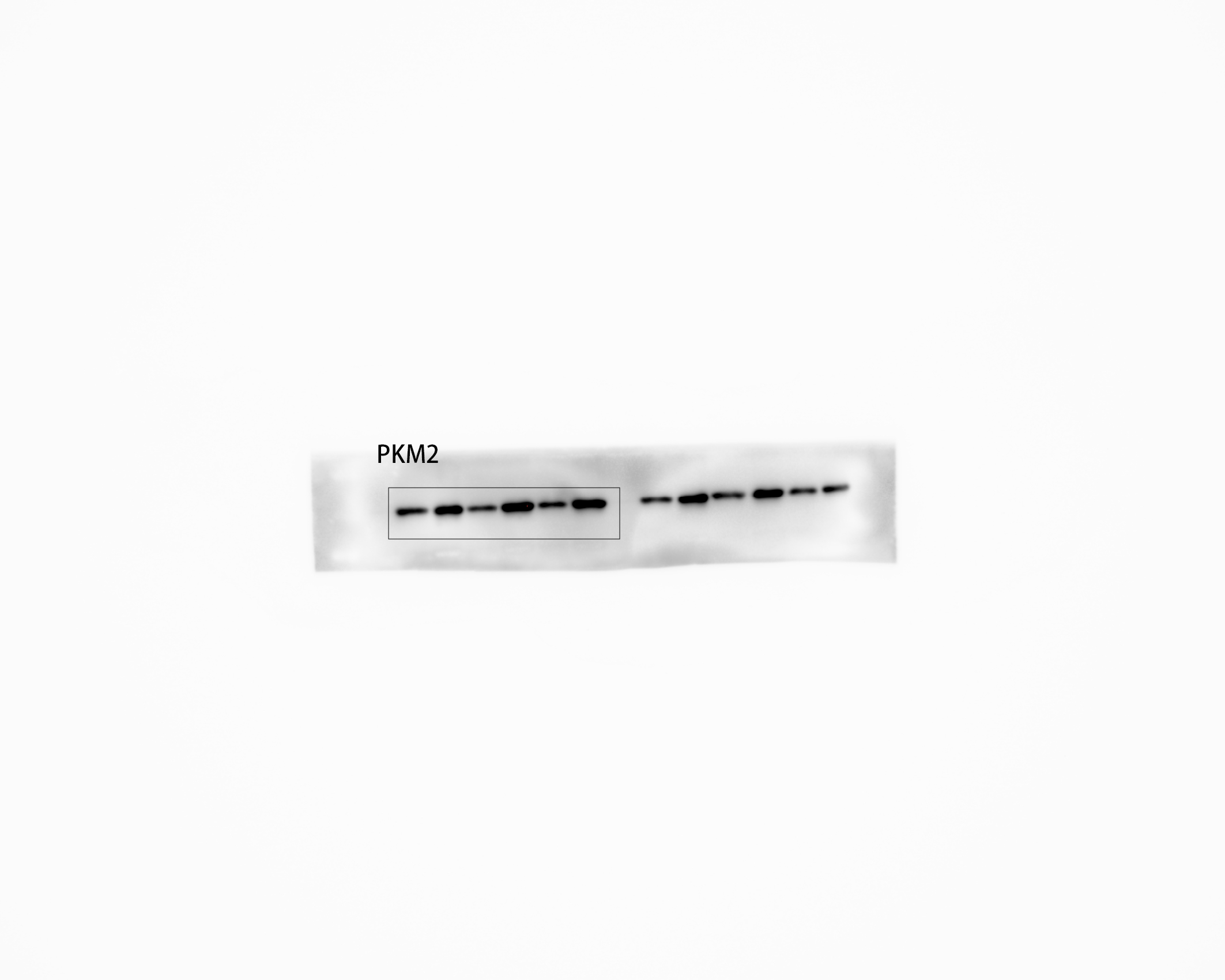

Supplement: Supplementary file 3 — Source data Fig. 1 [file 44318_2024_110_MOESM3_ESM.zip › Figure 1/1M/4-PKM2.tif]

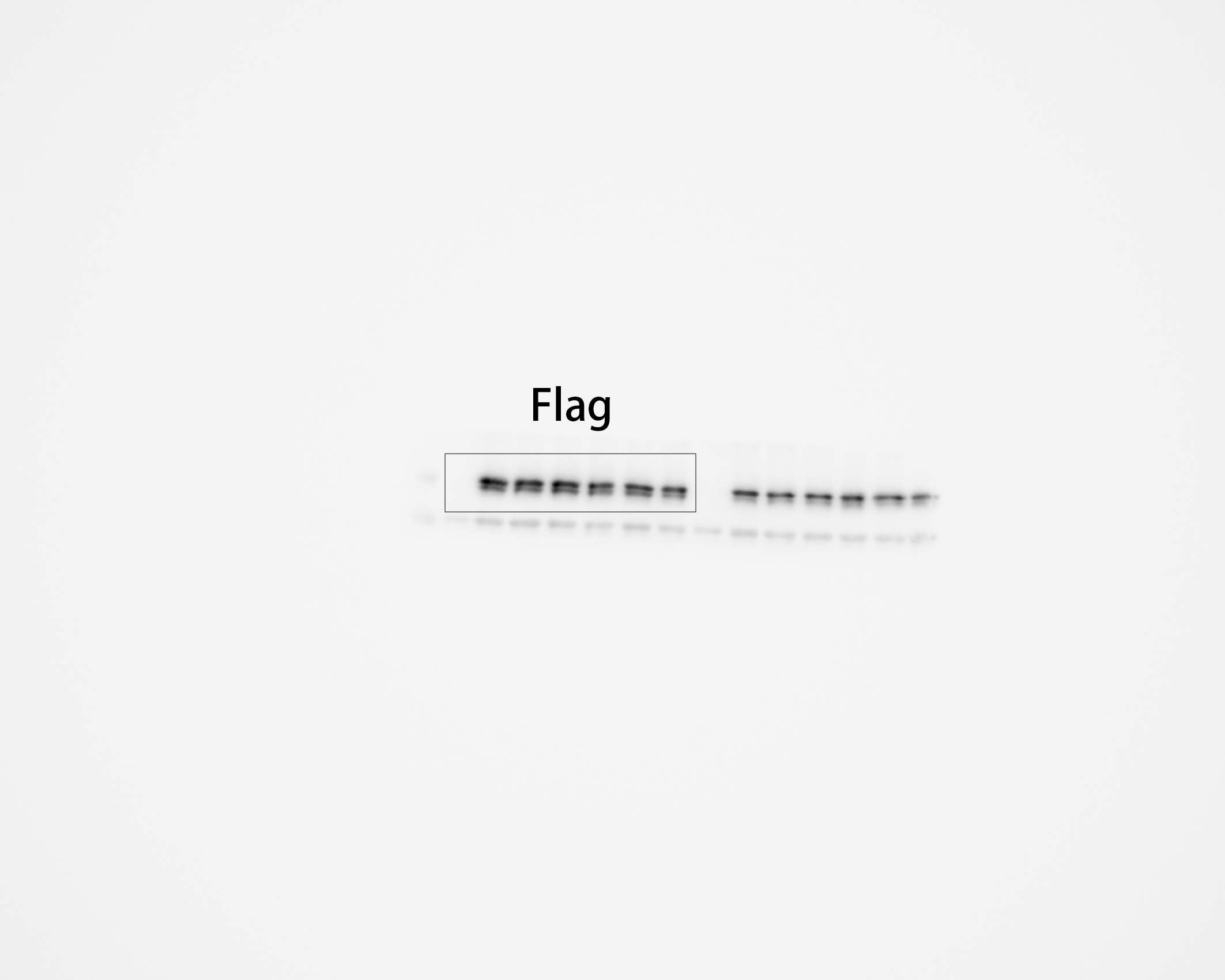

Supplement: Supplementary file 3 — Source data Fig. 1 [file 44318_2024_110_MOESM3_ESM.zip › Figure 1/1M/3-Flag.tif]

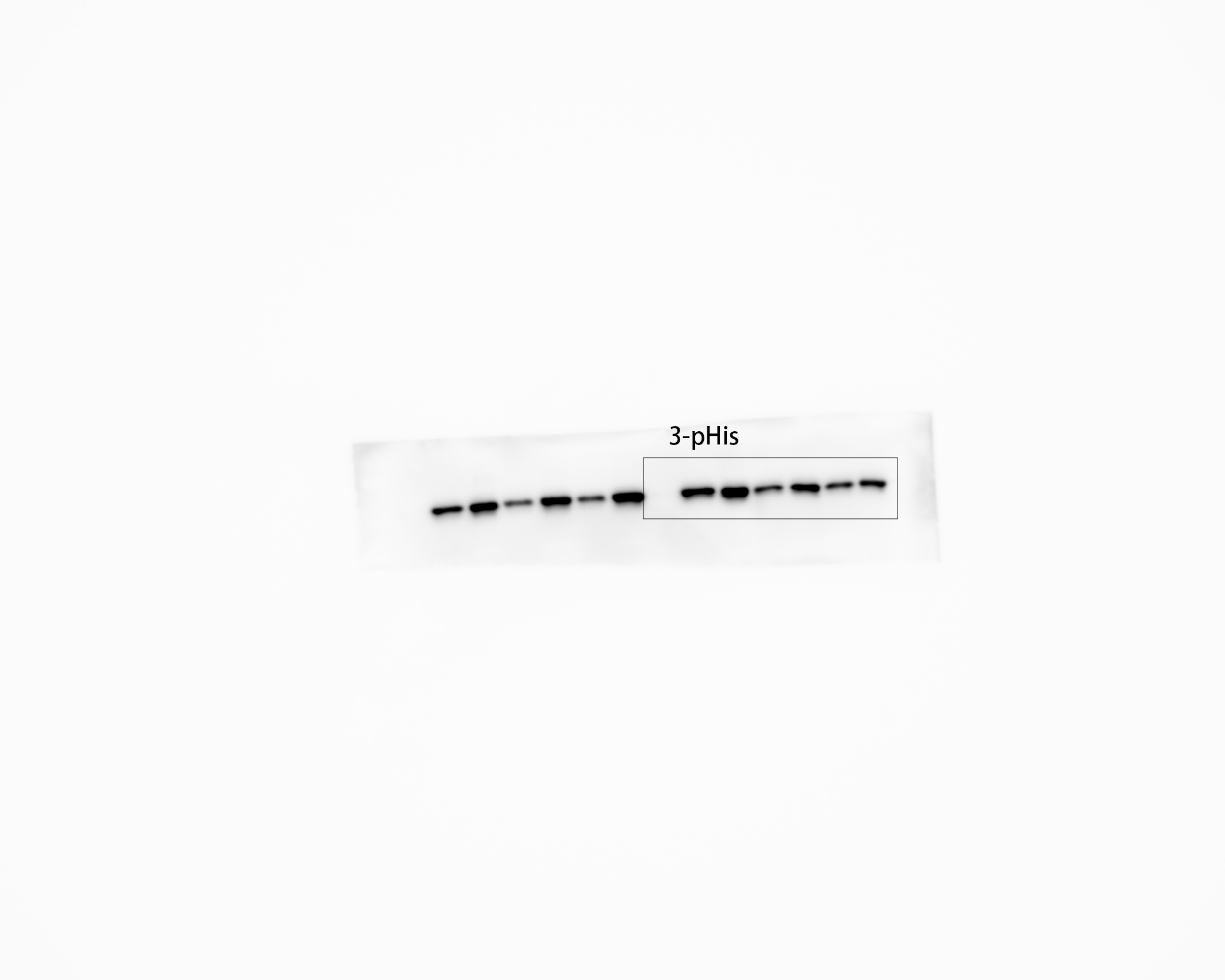

Supplement: Supplementary file 3 — Source data Fig. 1 [file 44318_2024_110_MOESM3_ESM.zip › Figure 1/1M/1-3-pHis.tif]

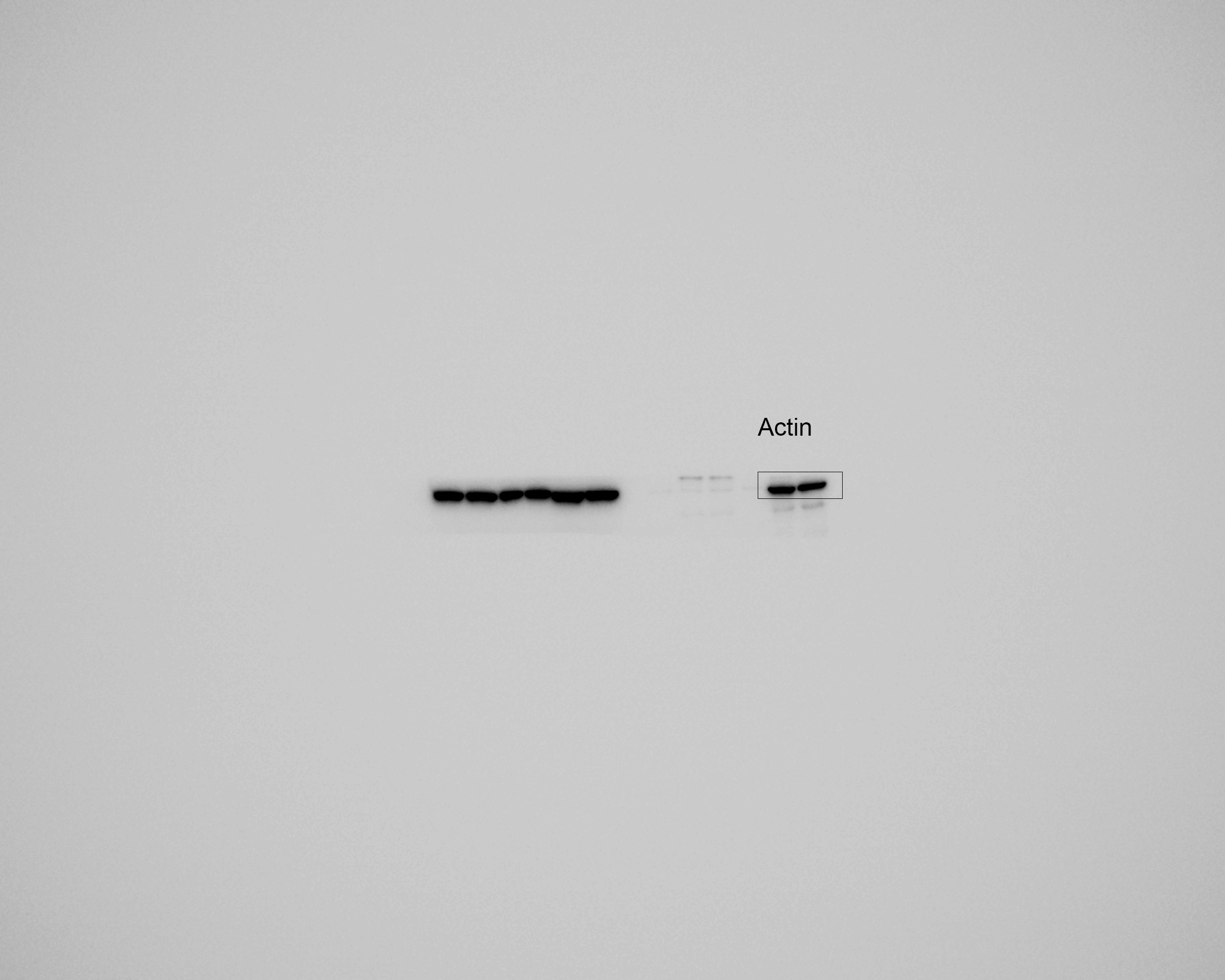

Supplement: Supplementary file 4 — Source data Fig. 2 [file 44318_2024_110_MOESM4_ESM.zip › Figure 2/2A/7-Actin.tif]

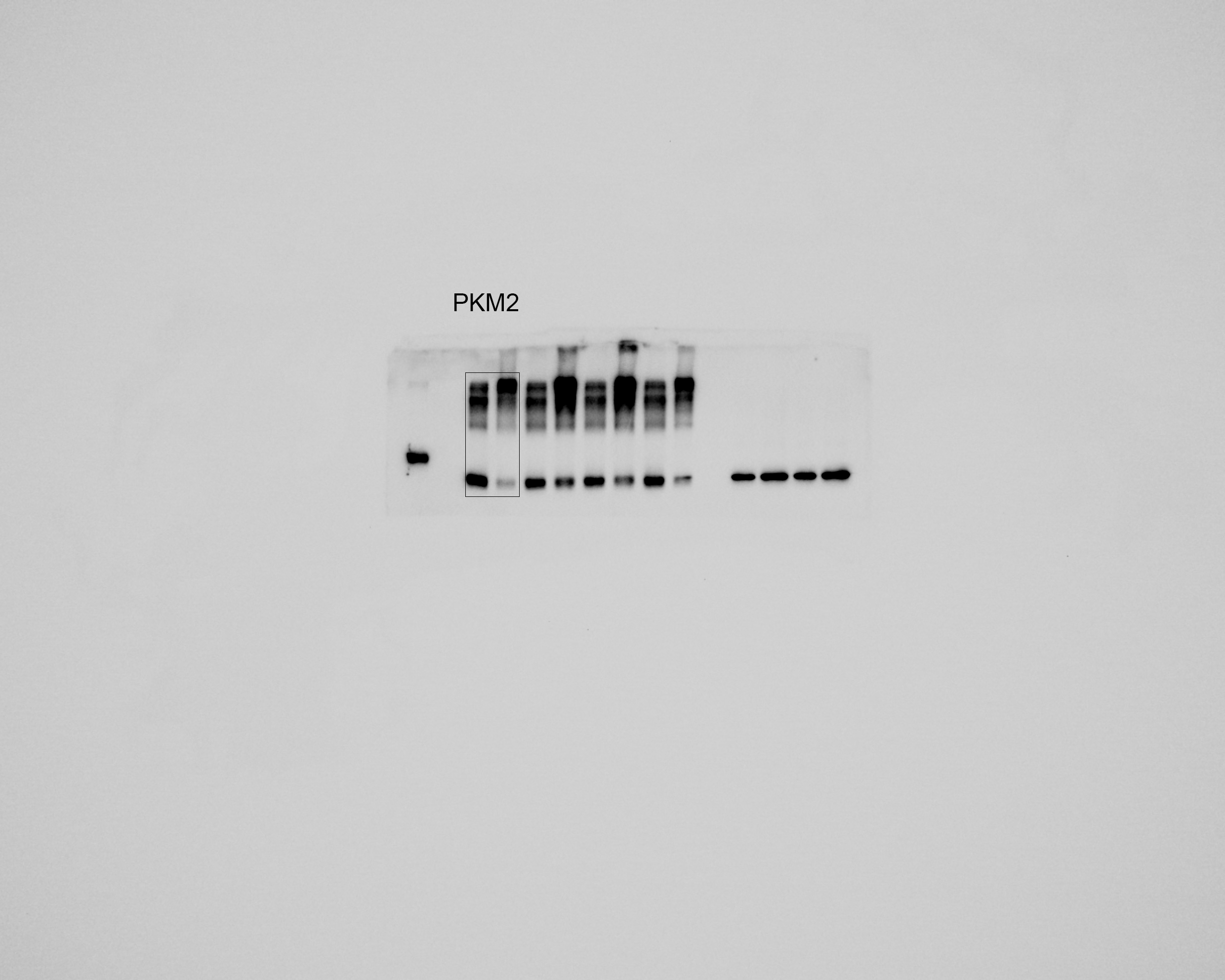

Supplement: Supplementary file 4 — Source data Fig. 2 [file 44318_2024_110_MOESM4_ESM.zip › Figure 2/2A/4-PKM2.tif]

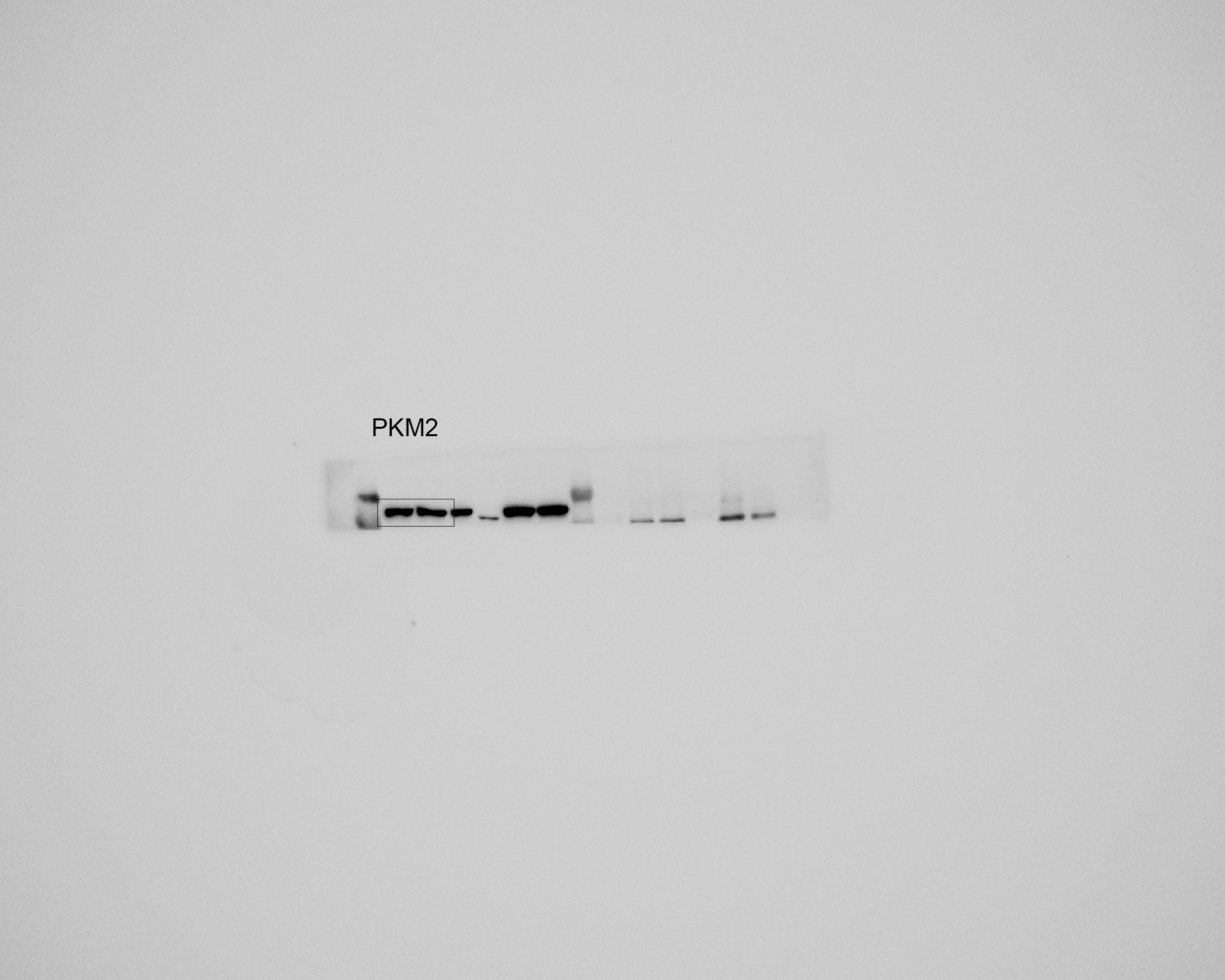

Supplement: Supplementary file 4 — Source data Fig. 2 [file 44318_2024_110_MOESM4_ESM.zip › Figure 2/2A/5-PKM2.tif]

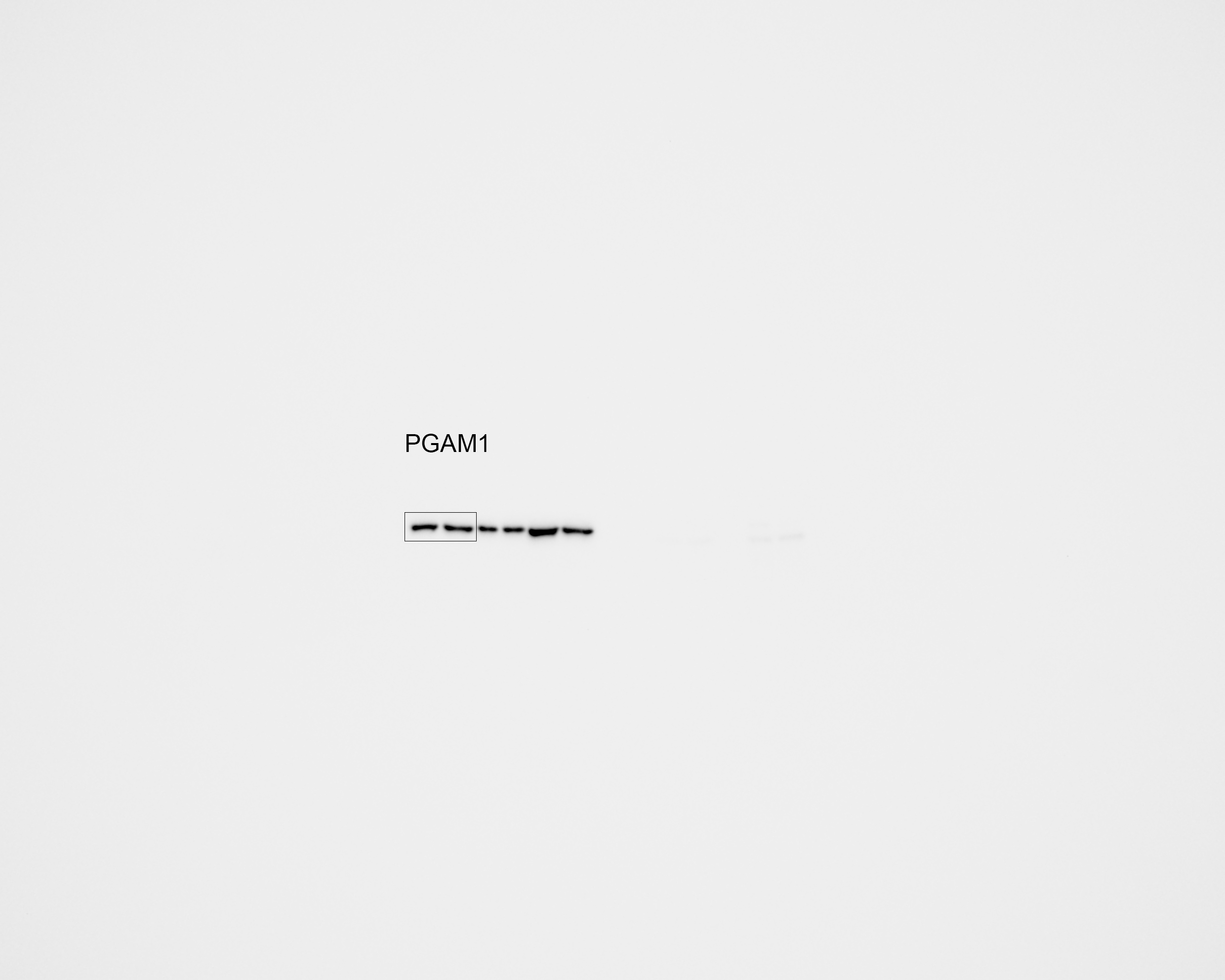

Supplement: Supplementary file 4 — Source data Fig. 2 [file 44318_2024_110_MOESM4_ESM.zip › Figure 2/2A/6-PGAM1.tif]

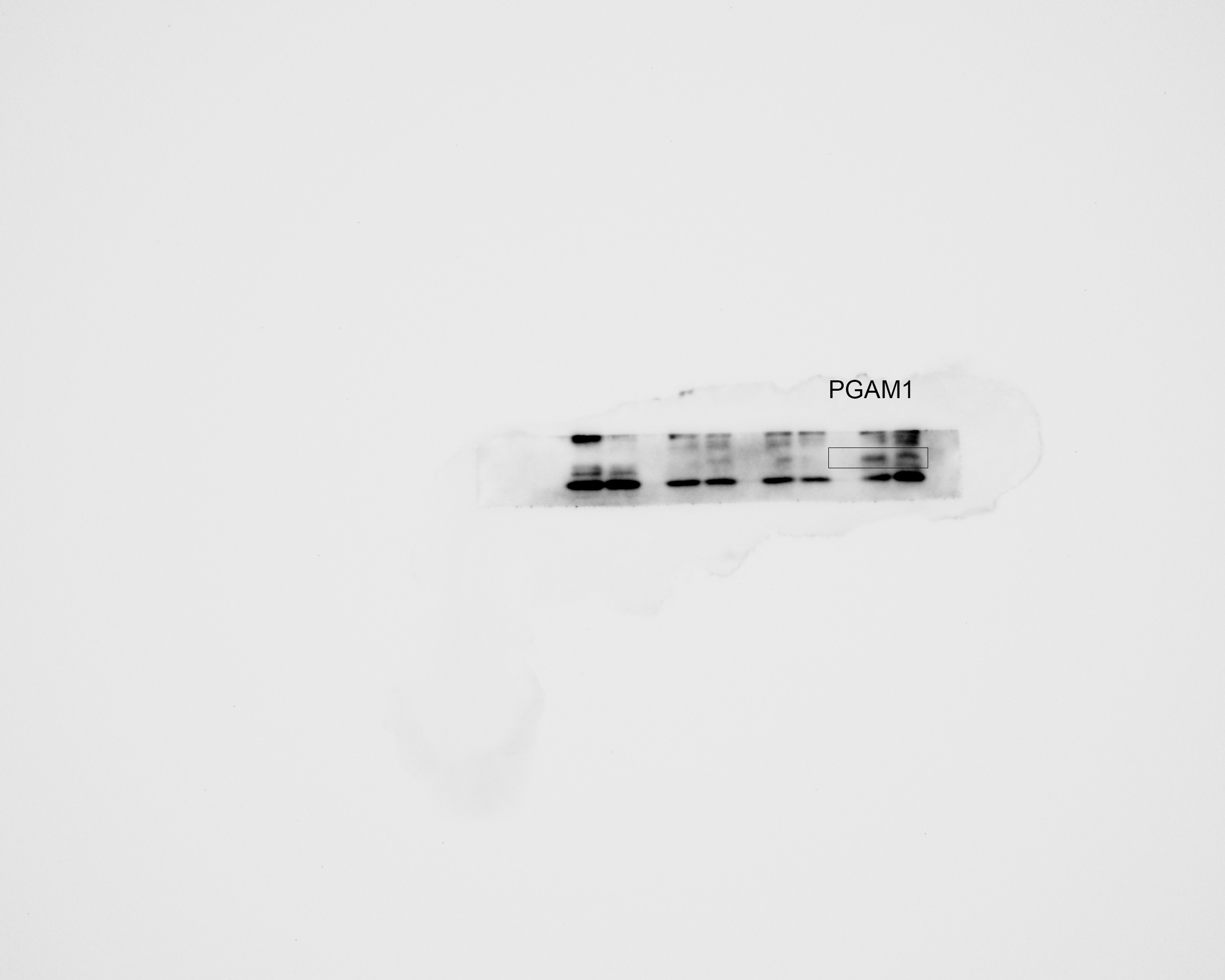

Supplement: Supplementary file 4 — Source data Fig. 2 [file 44318_2024_110_MOESM4_ESM.zip › Figure 2/2A/3-PGAM1.tif]

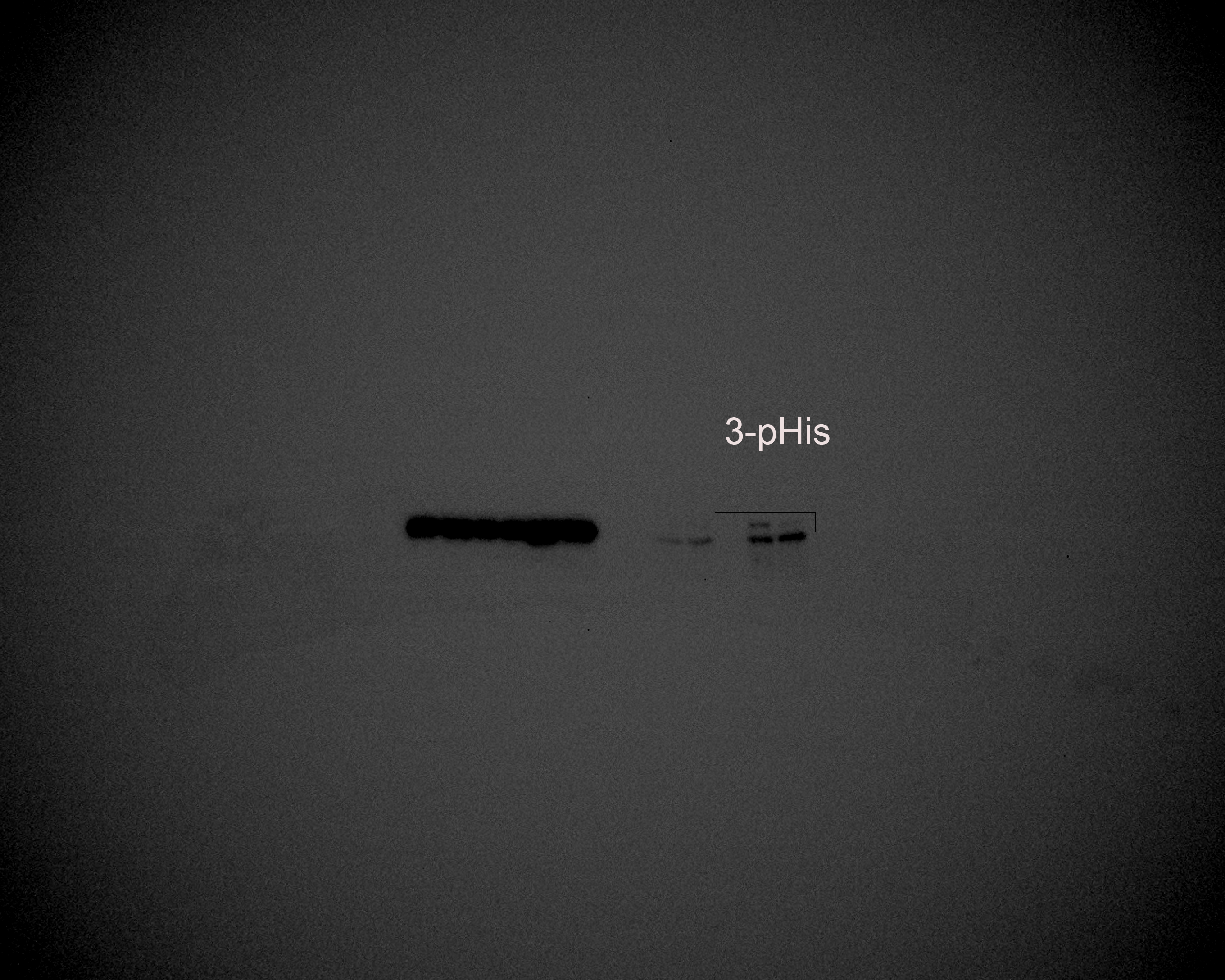

Supplement: Supplementary file 4 — Source data Fig. 2 [file 44318_2024_110_MOESM4_ESM.zip › Figure 2/2A/2-3-pHis.tif]

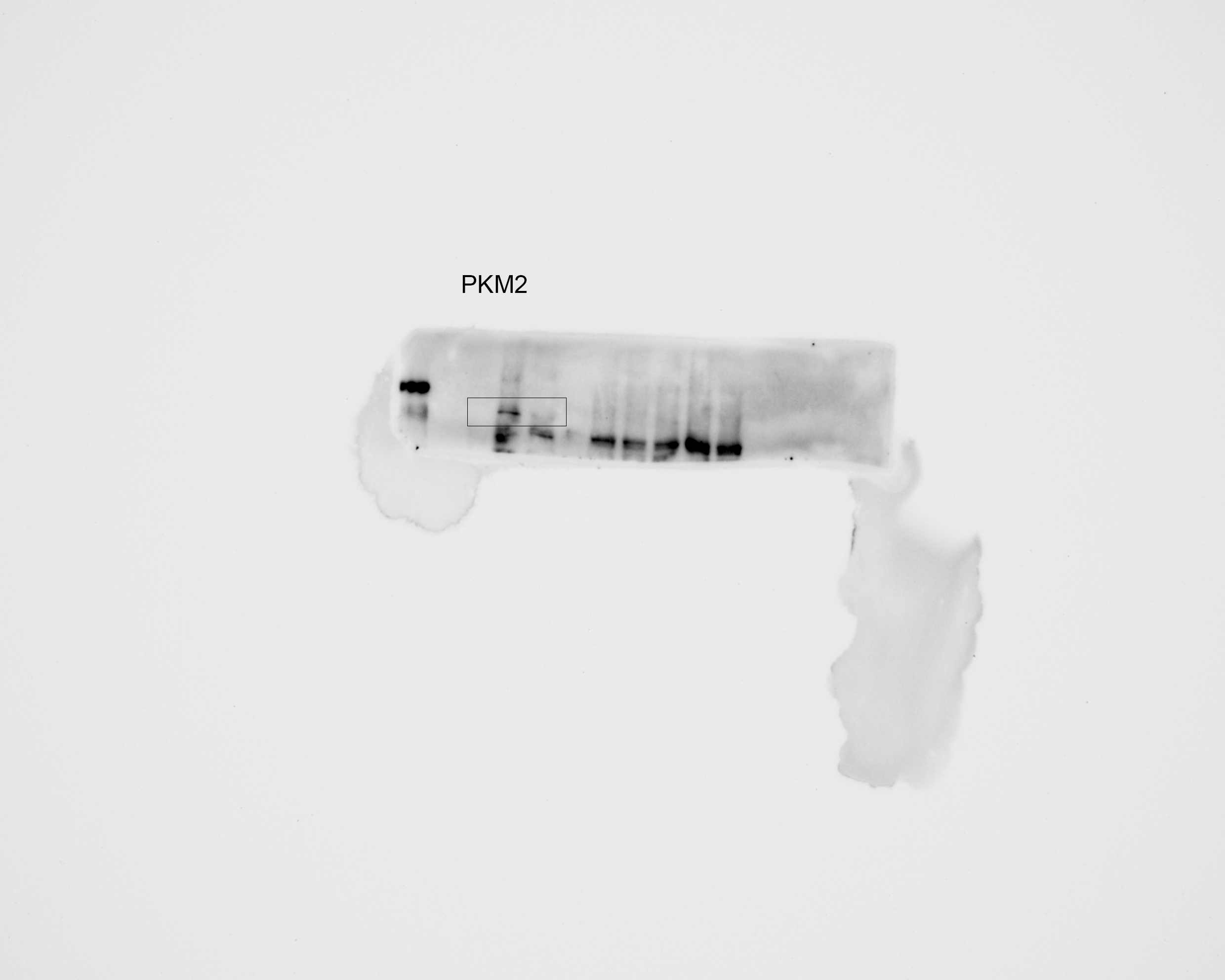

Supplement: Supplementary file 4 — Source data Fig. 2 [file 44318_2024_110_MOESM4_ESM.zip › Figure 2/2A/1-PKM2.tif]

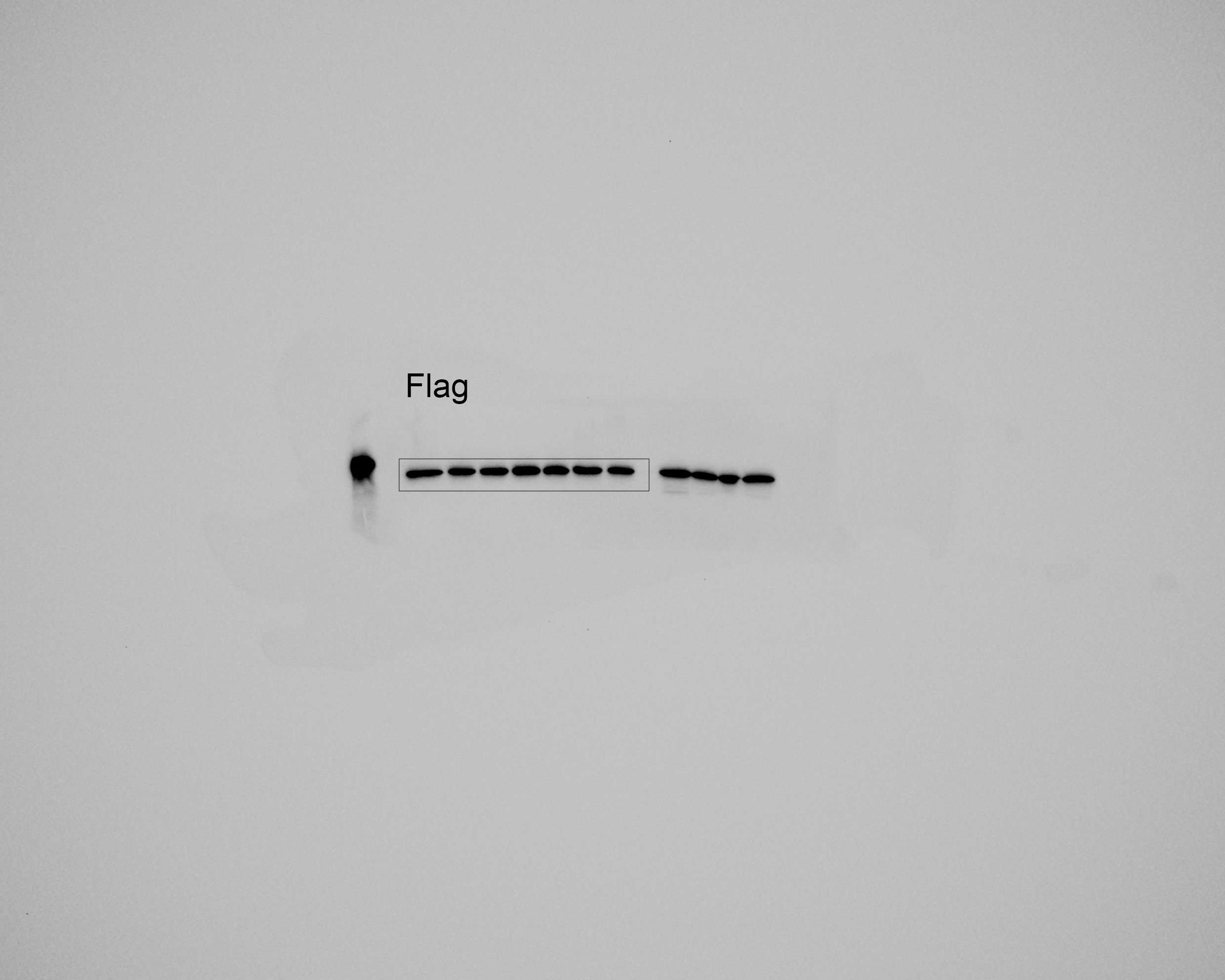

Supplement: Supplementary file 4 — Source data Fig. 2 [file 44318_2024_110_MOESM4_ESM.zip › Figure 2/2C/5-Flag.tif]

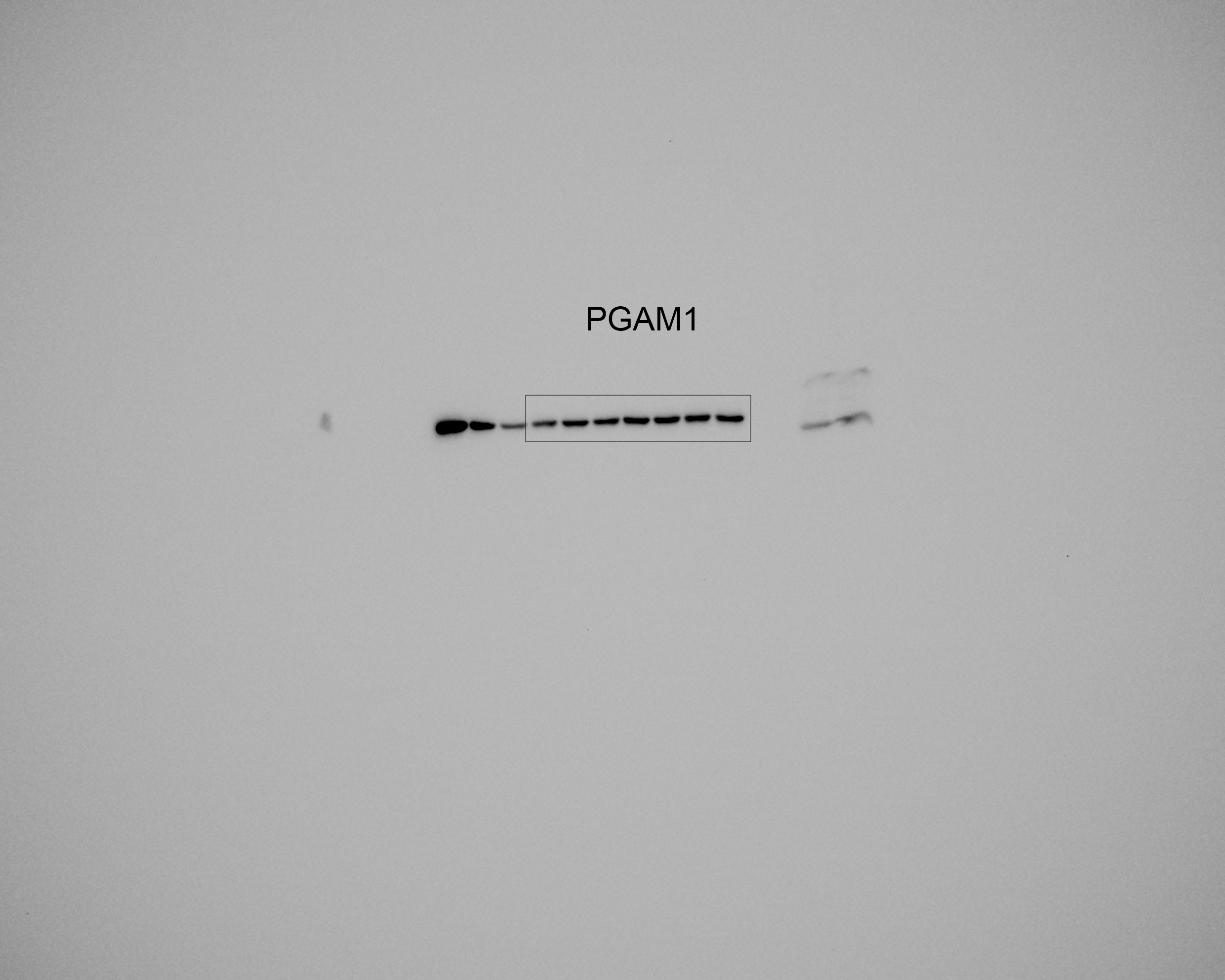

Supplement: Supplementary file 4 — Source data Fig. 2 [file 44318_2024_110_MOESM4_ESM.zip › Figure 2/2C/6-PGAM1.tif]

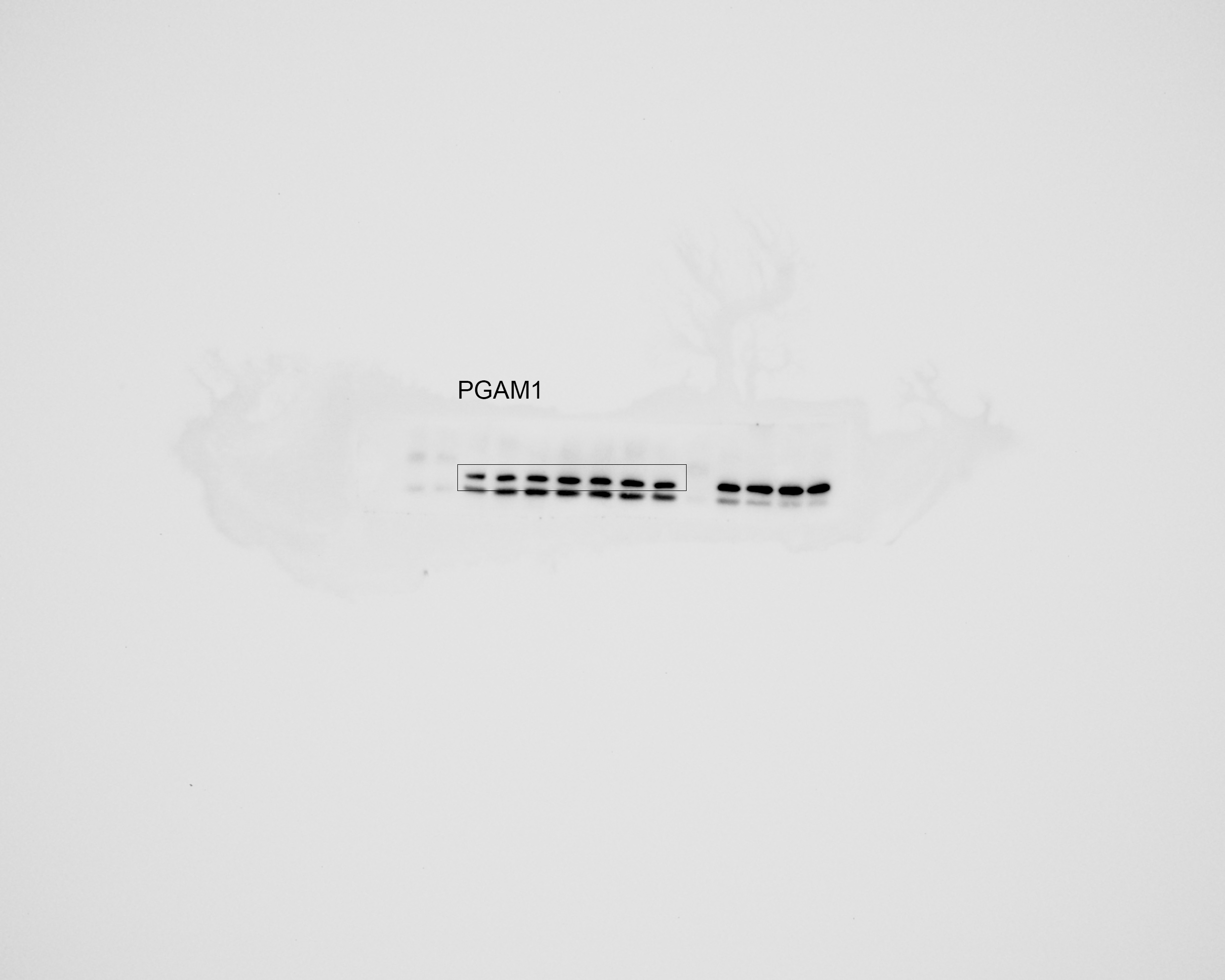

Supplement: Supplementary file 4 — Source data Fig. 2 [file 44318_2024_110_MOESM4_ESM.zip › Figure 2/2C/3-PGAM1.tif]

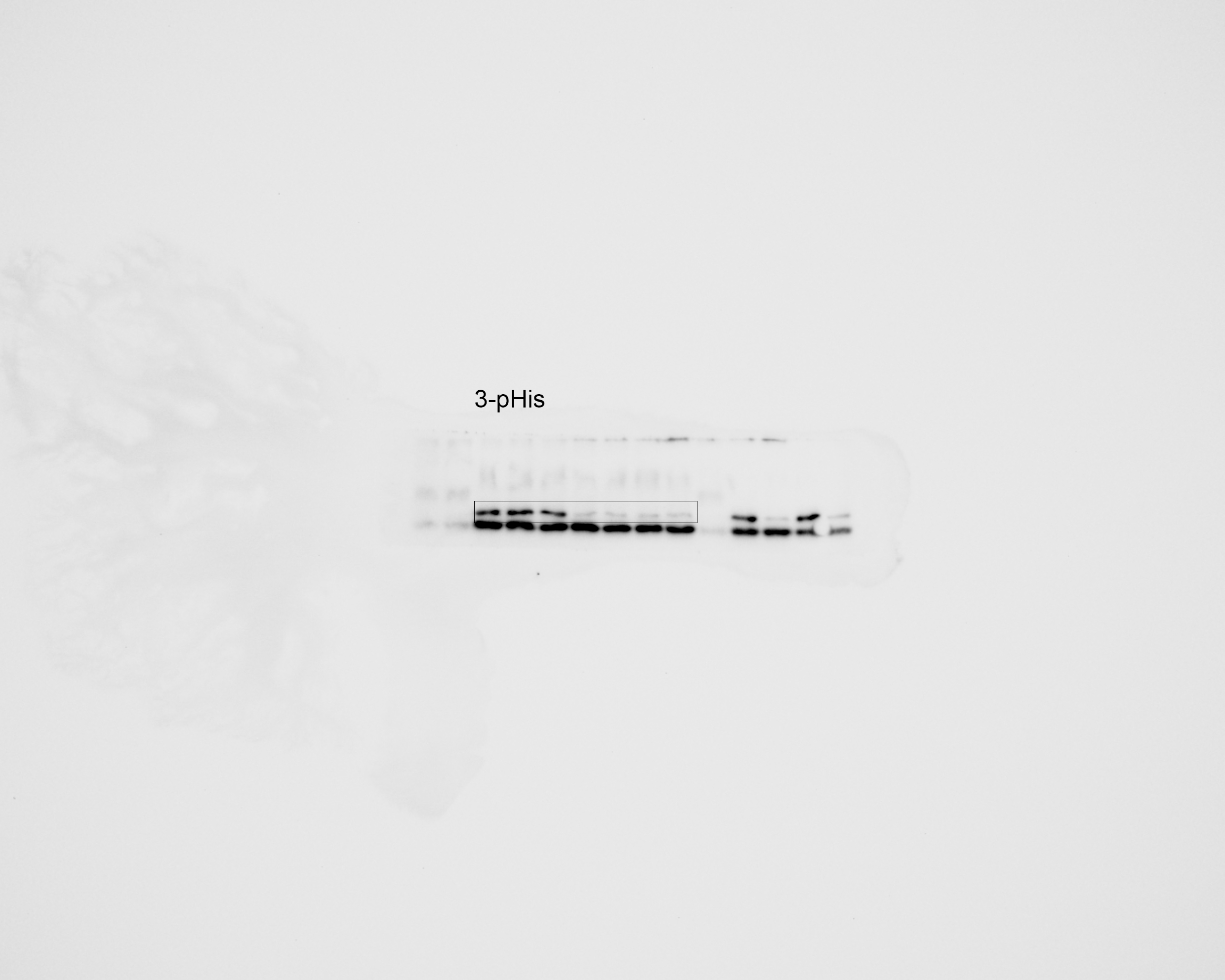

Supplement: Supplementary file 4 — Source data Fig. 2 [file 44318_2024_110_MOESM4_ESM.zip › Figure 2/2C/2-3-pHis.tif]

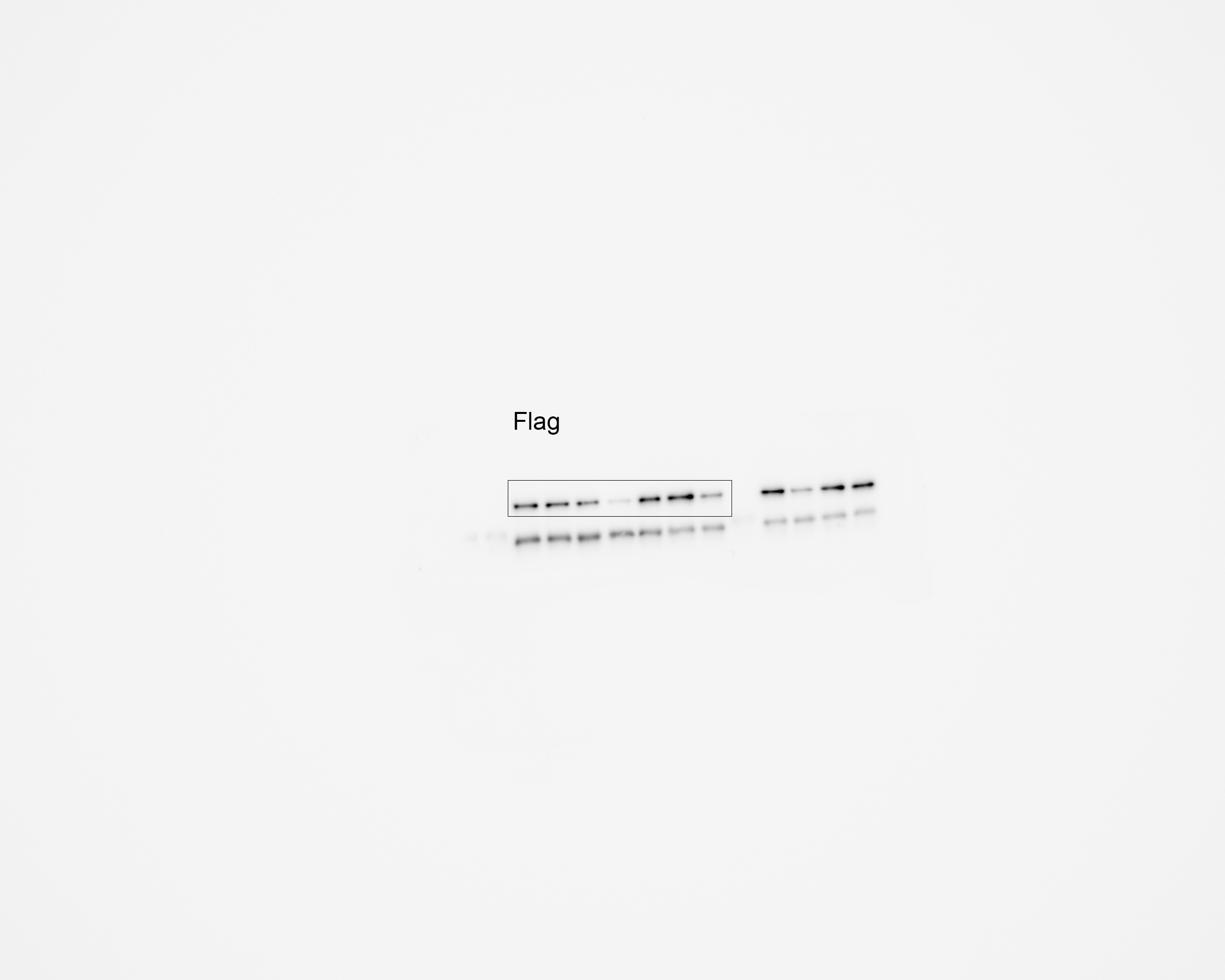

Supplement: Supplementary file 4 — Source data Fig. 2 [file 44318_2024_110_MOESM4_ESM.zip › Figure 2/2C/1-Flag.tif]

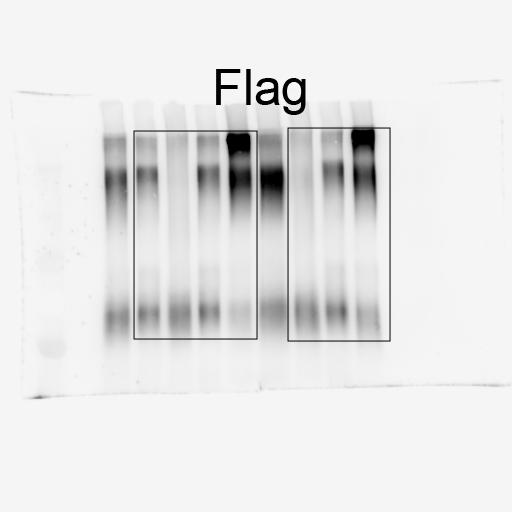

Supplement: Supplementary file 4 — Source data Fig. 2 [file 44318_2024_110_MOESM4_ESM.zip › Figure 2/2C/4-Flag (PKM2).tif]

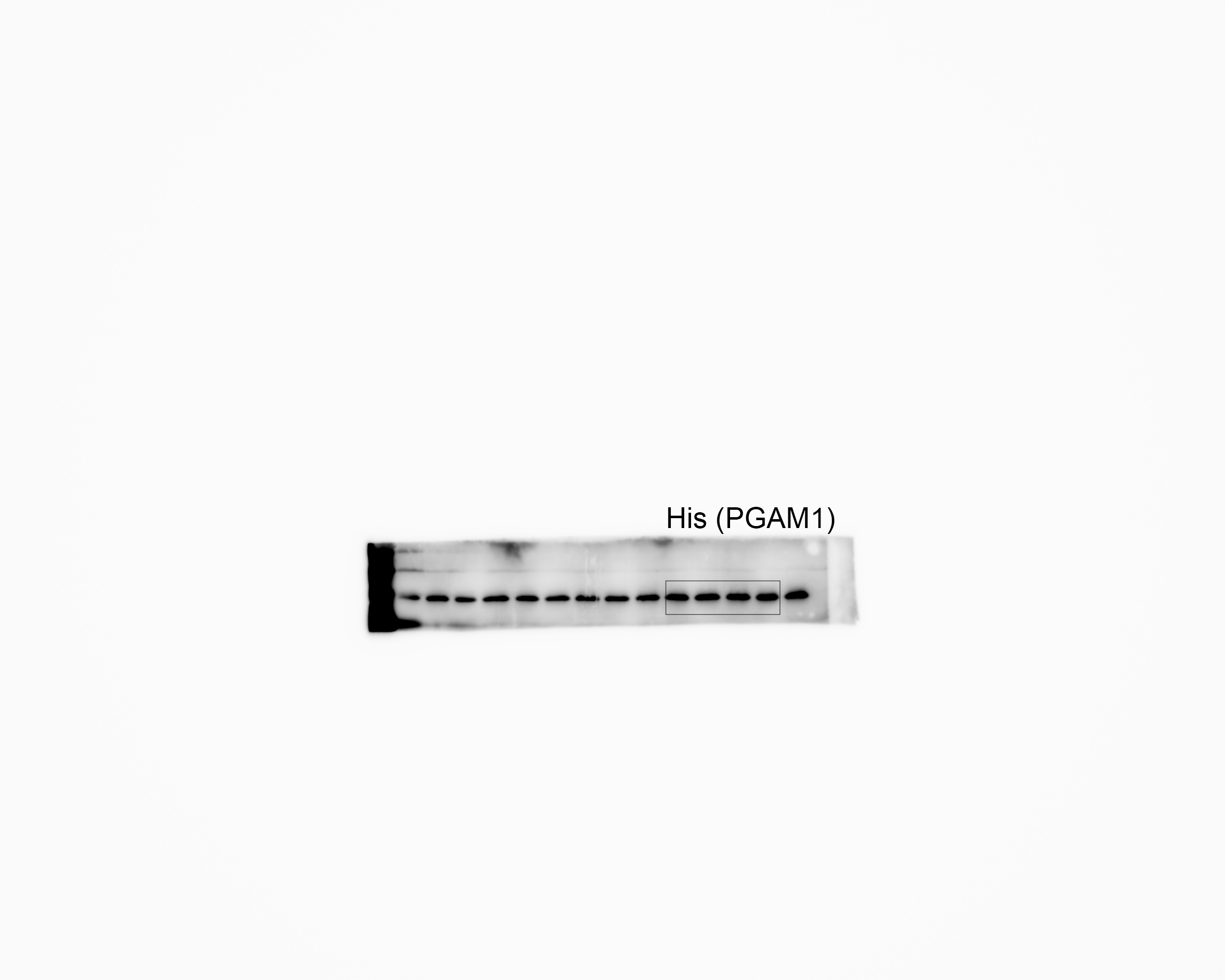

Supplement: Supplementary file 4 — Source data Fig. 2 [file 44318_2024_110_MOESM4_ESM.zip › Figure 2/2D/6-His (PGAM1).tif]

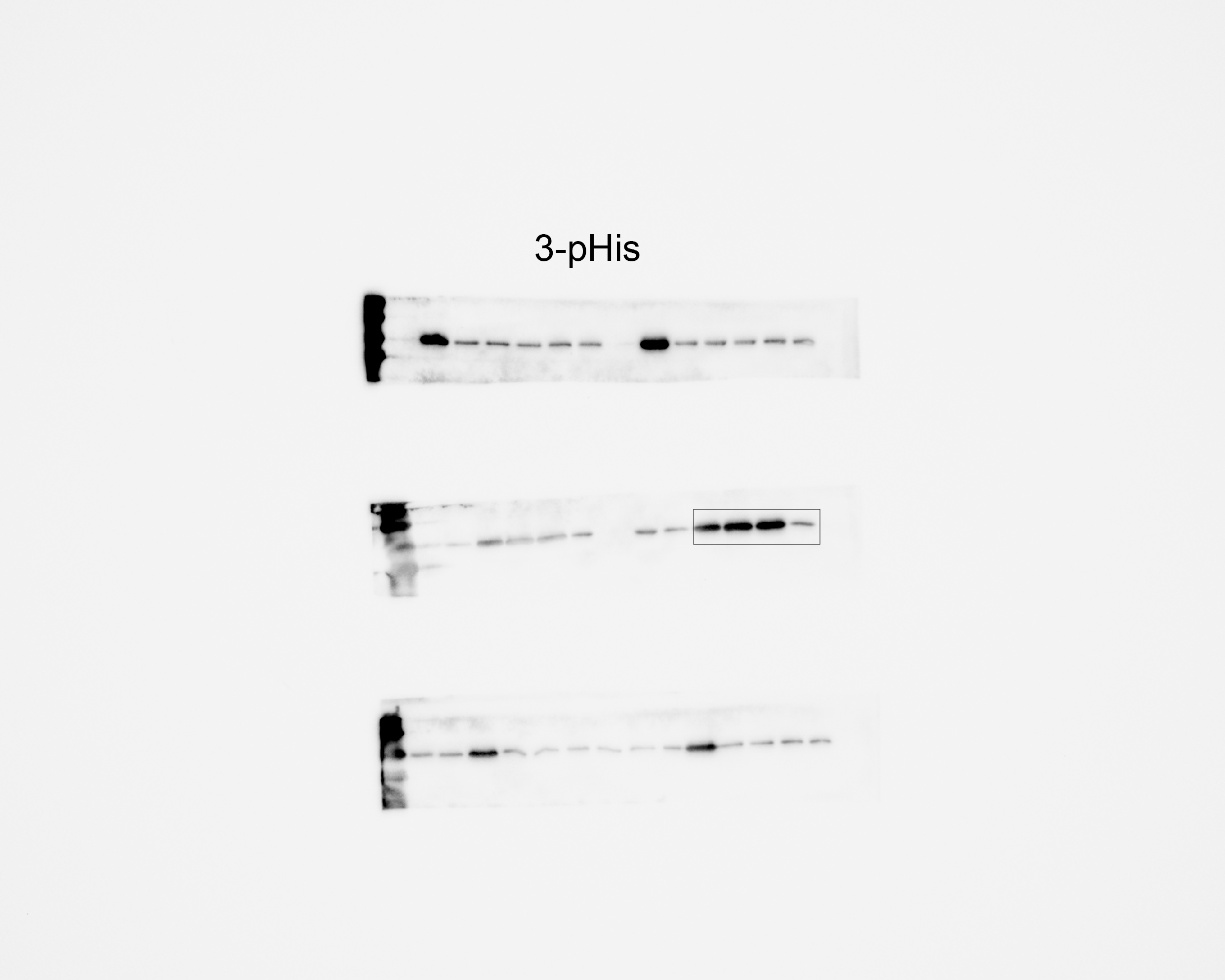

Supplement: Supplementary file 4 — Source data Fig. 2 [file 44318_2024_110_MOESM4_ESM.zip › Figure 2/2D/1-3-pHis.tif]

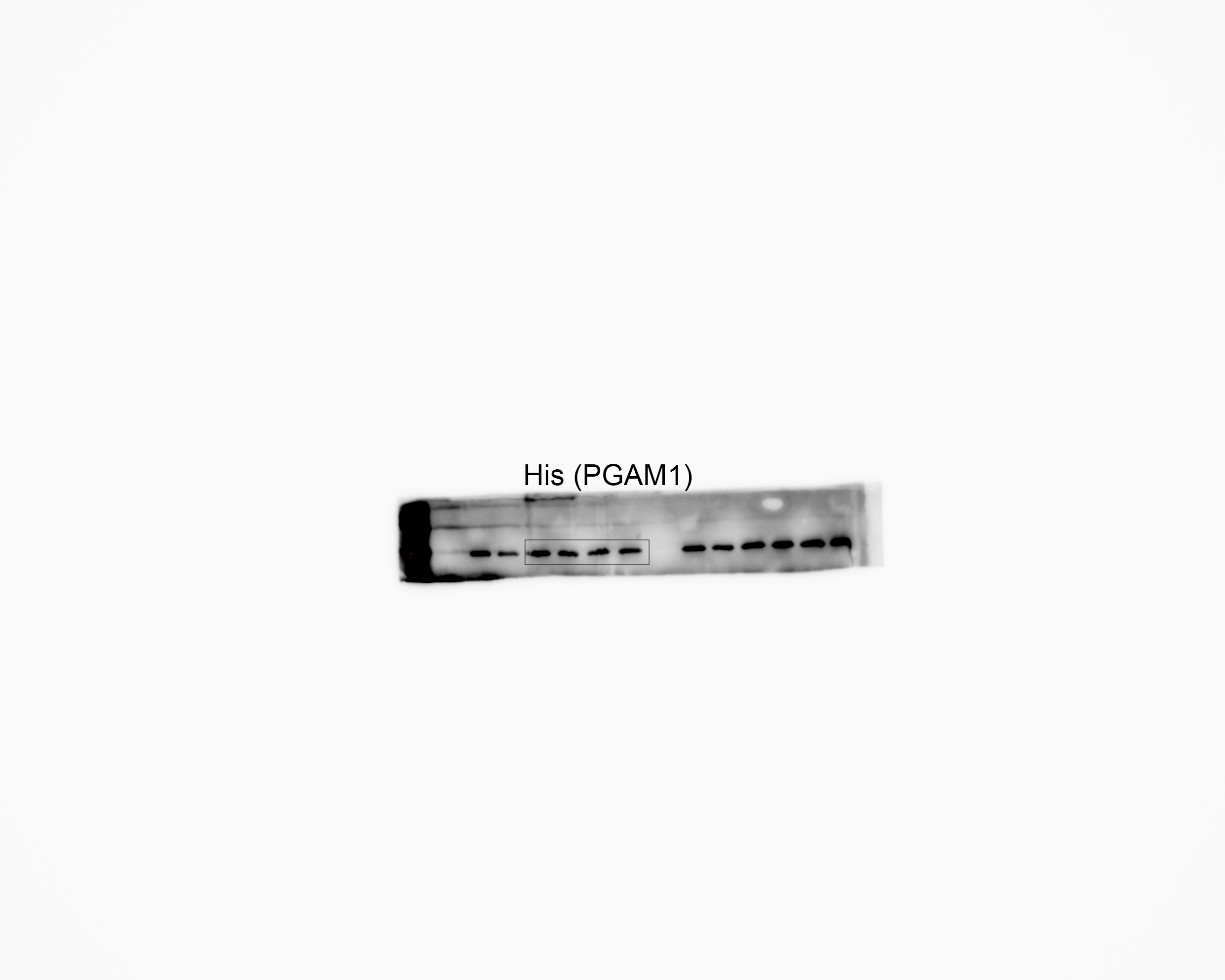

Supplement: Supplementary file 4 — Source data Fig. 2 [file 44318_2024_110_MOESM4_ESM.zip › Figure 2/2D/5-His (PGAM1).tif]

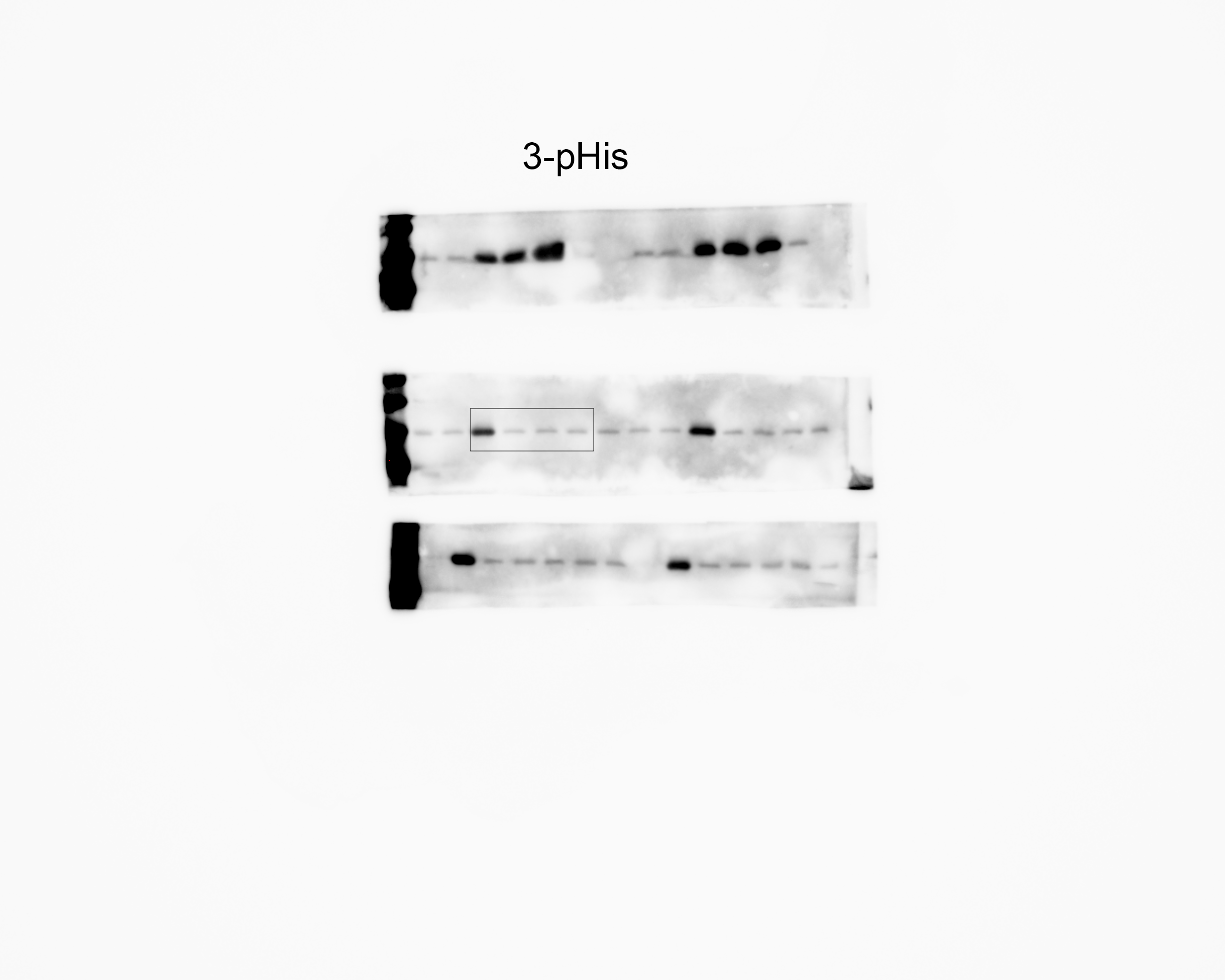

Supplement: Supplementary file 4 — Source data Fig. 2 [file 44318_2024_110_MOESM4_ESM.zip › Figure 2/2D/2-3-pHis.tif]

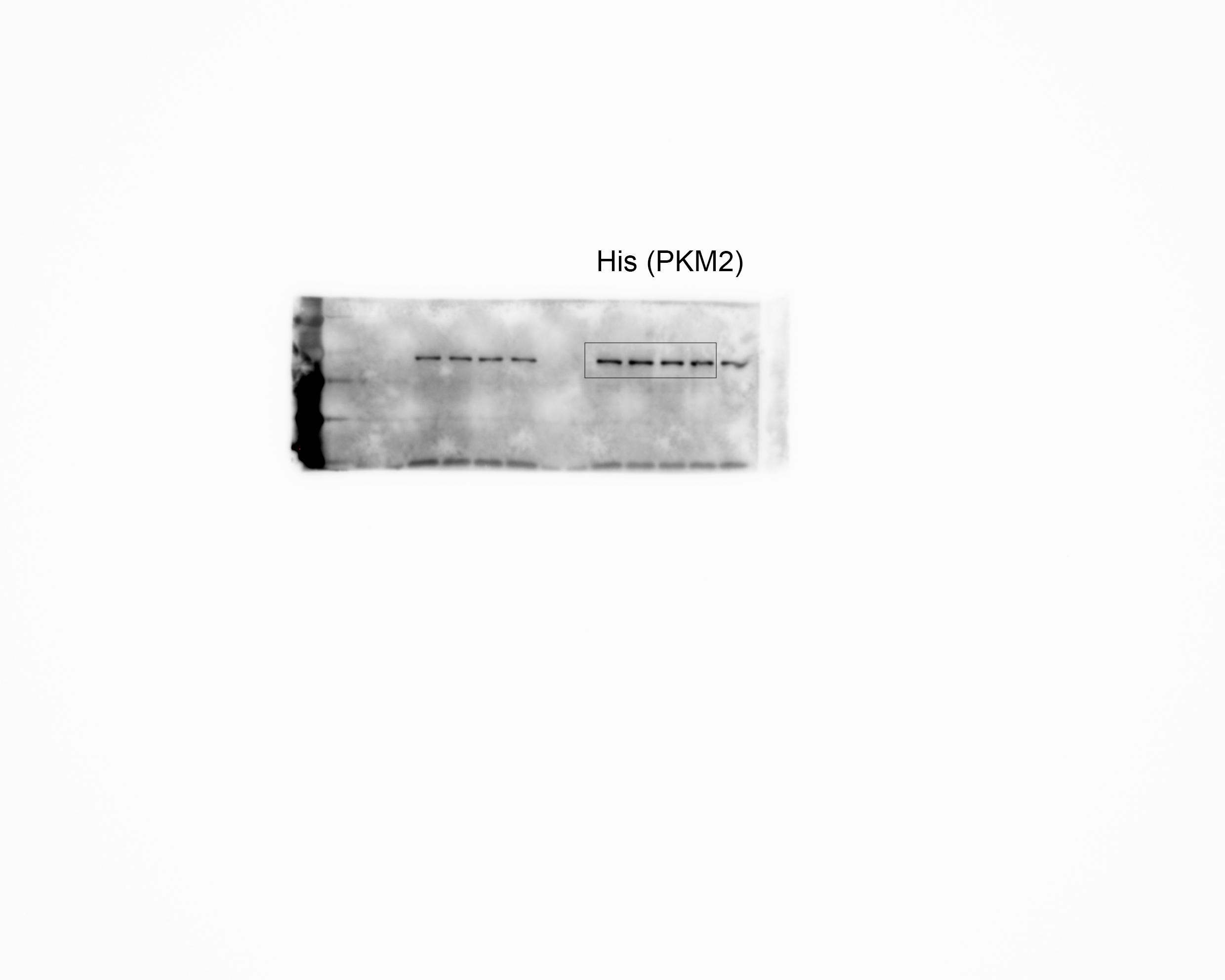

Supplement: Supplementary file 4 — Source data Fig. 2 [file 44318_2024_110_MOESM4_ESM.zip › Figure 2/2D/4-His (PKM2).tif]

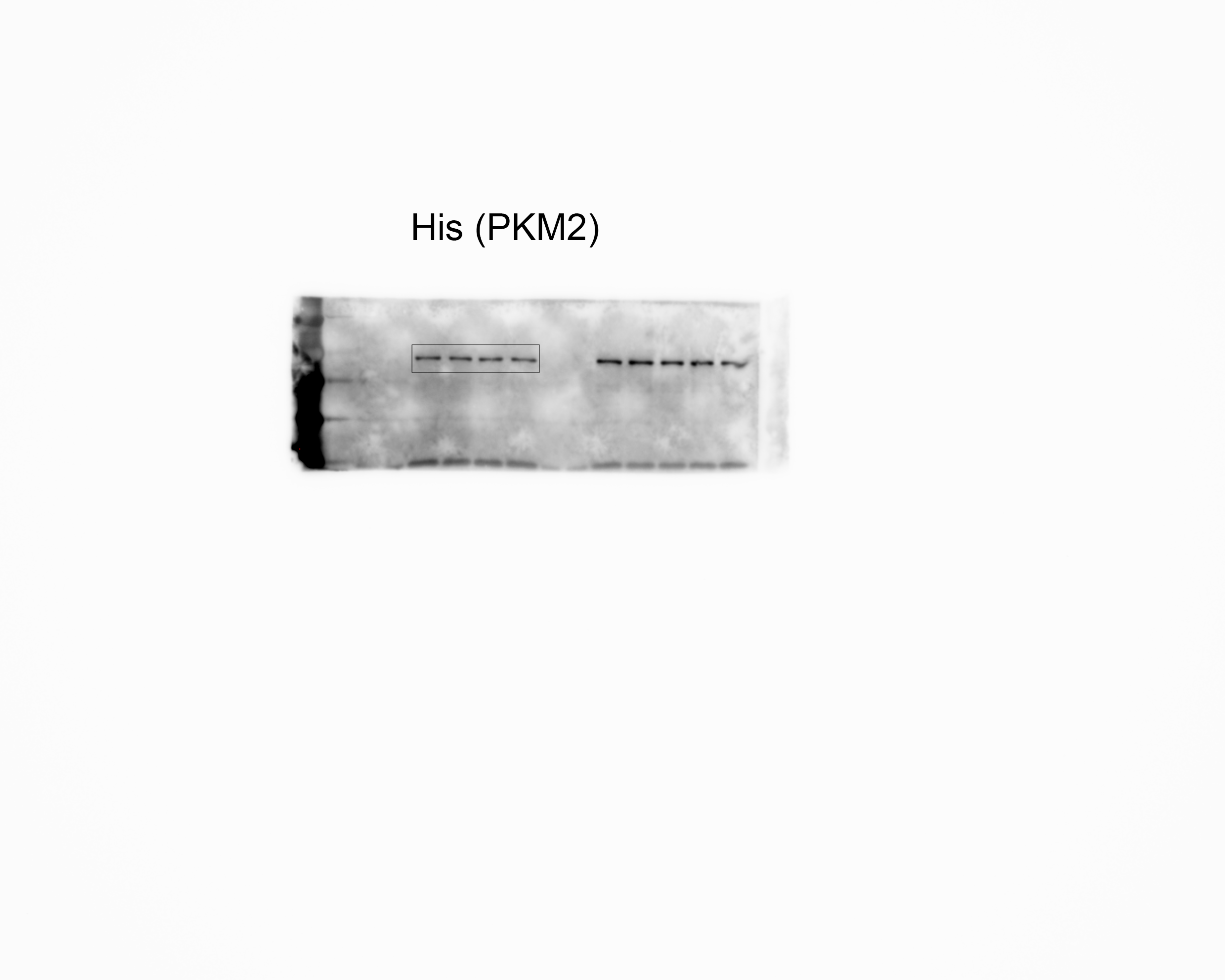

Supplement: Supplementary file 4 — Source data Fig. 2 [file 44318_2024_110_MOESM4_ESM.zip › Figure 2/2D/3-His (PKM2).tif]

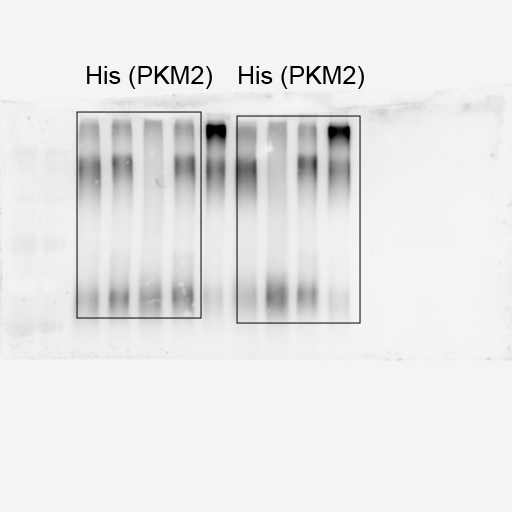

Supplement: Supplementary file 4 — Source data Fig. 2 [file 44318_2024_110_MOESM4_ESM.zip › Figure 2/2D/7-His (PKM2).tif]

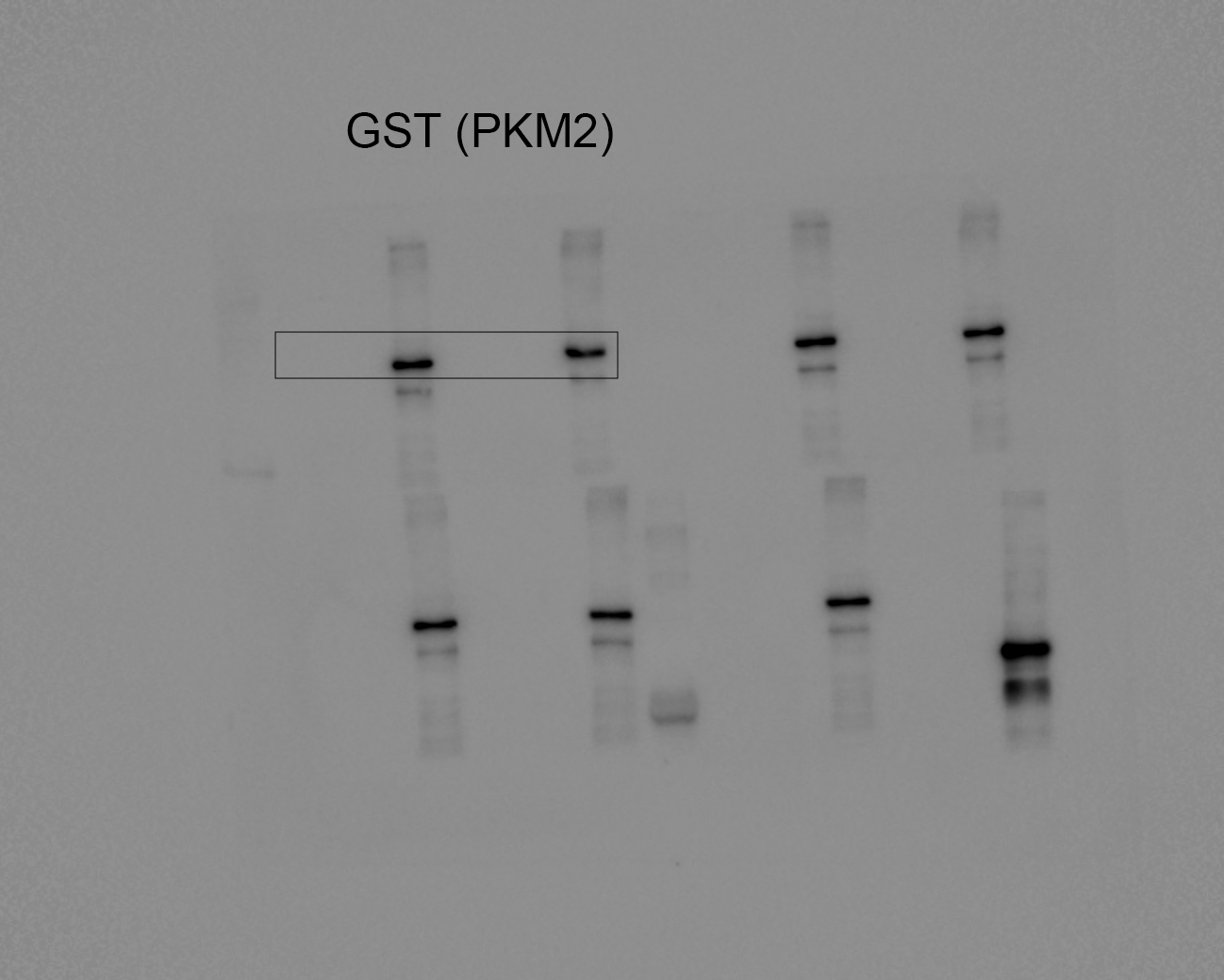

Supplement: Supplementary file 5 — Source data Fig. 3 [file 44318_2024_110_MOESM5_ESM.zip › Figure 3/3E/2-GST (PKM2).tif]

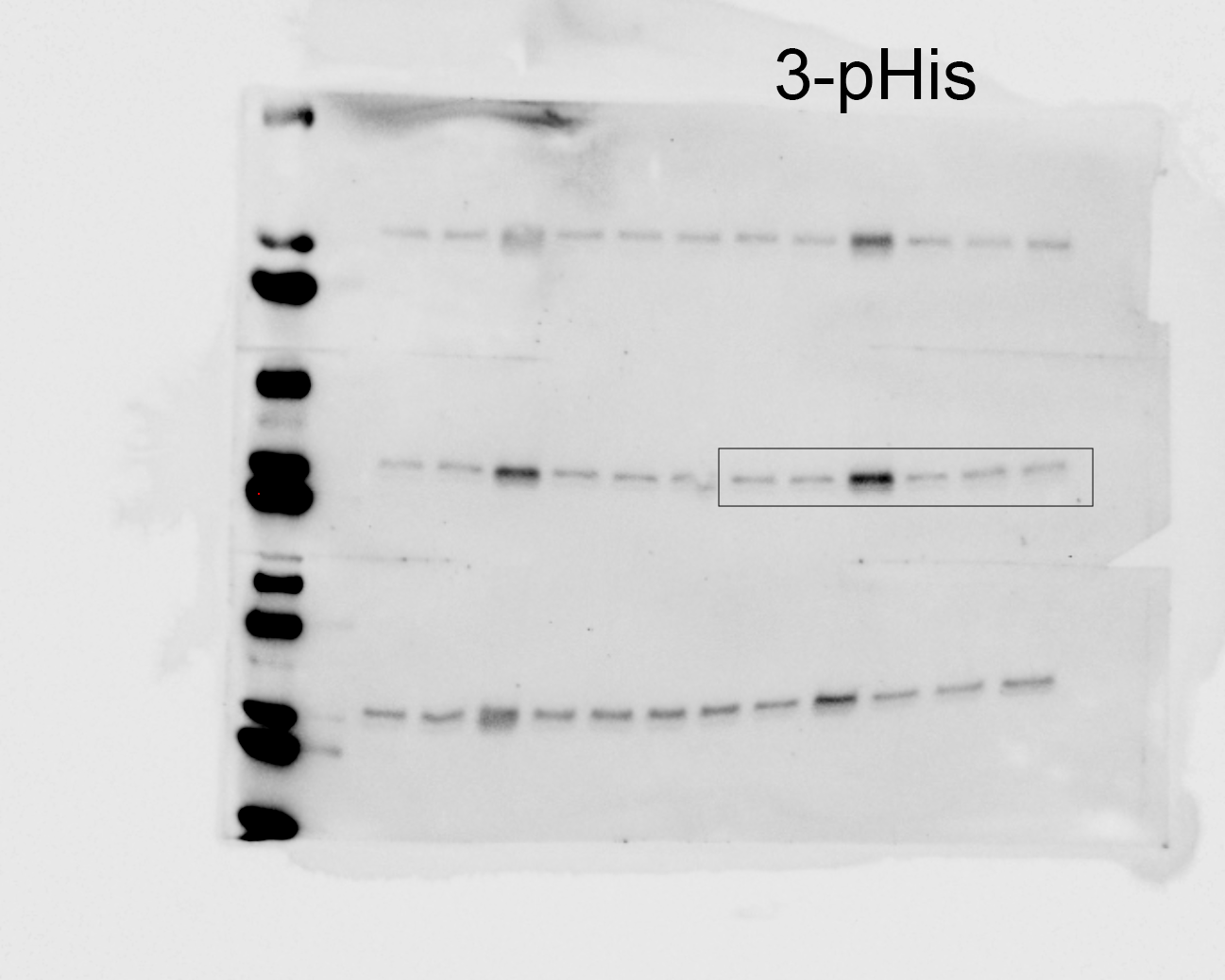

Supplement: Supplementary file 5 — Source data Fig. 3 [file 44318_2024_110_MOESM5_ESM.zip › Figure 3/3E/1-3-pHis.tif]

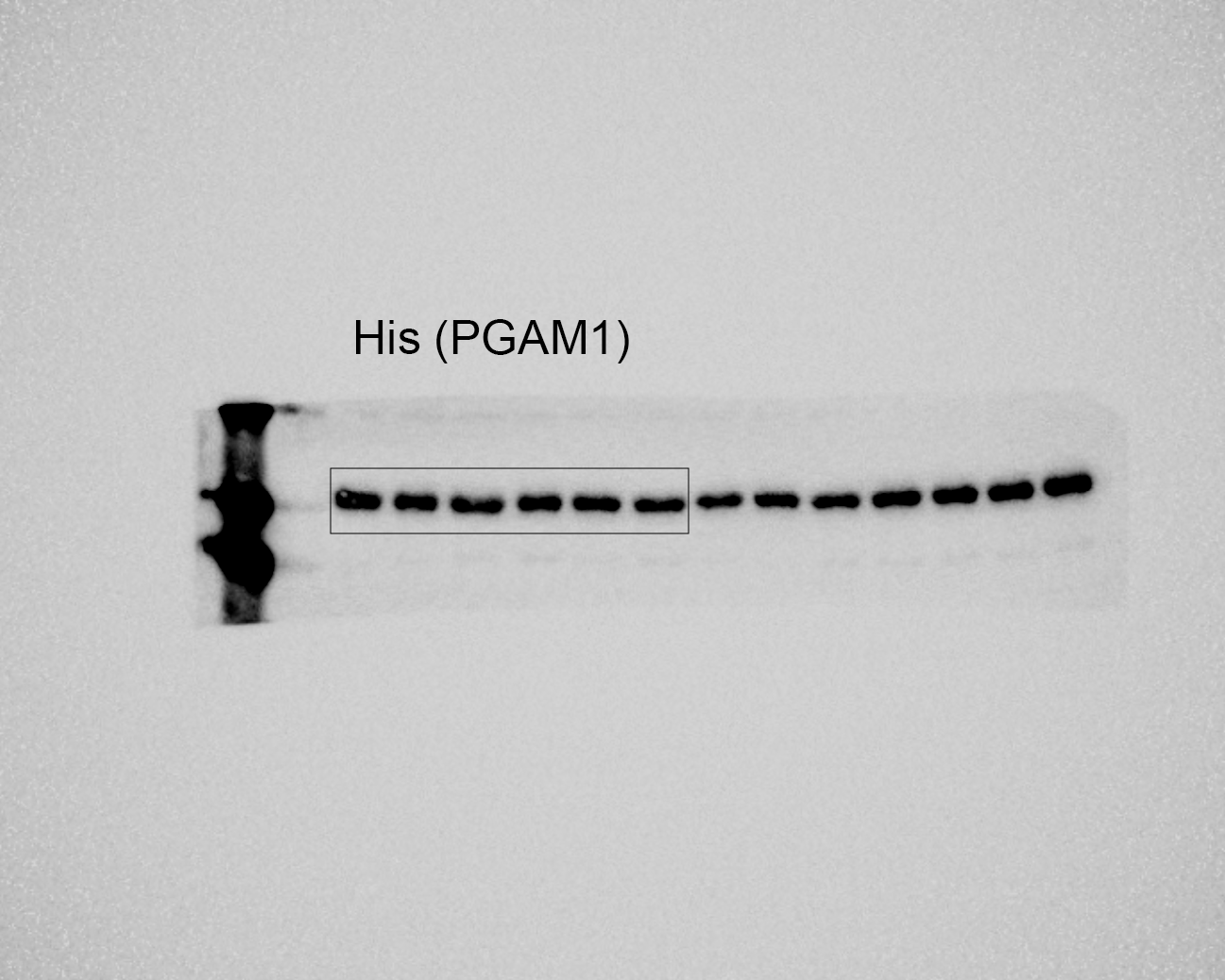

Supplement: Supplementary file 5 — Source data Fig. 3 [file 44318_2024_110_MOESM5_ESM.zip › Figure 3/3E/3-His (PGAM1).tif]

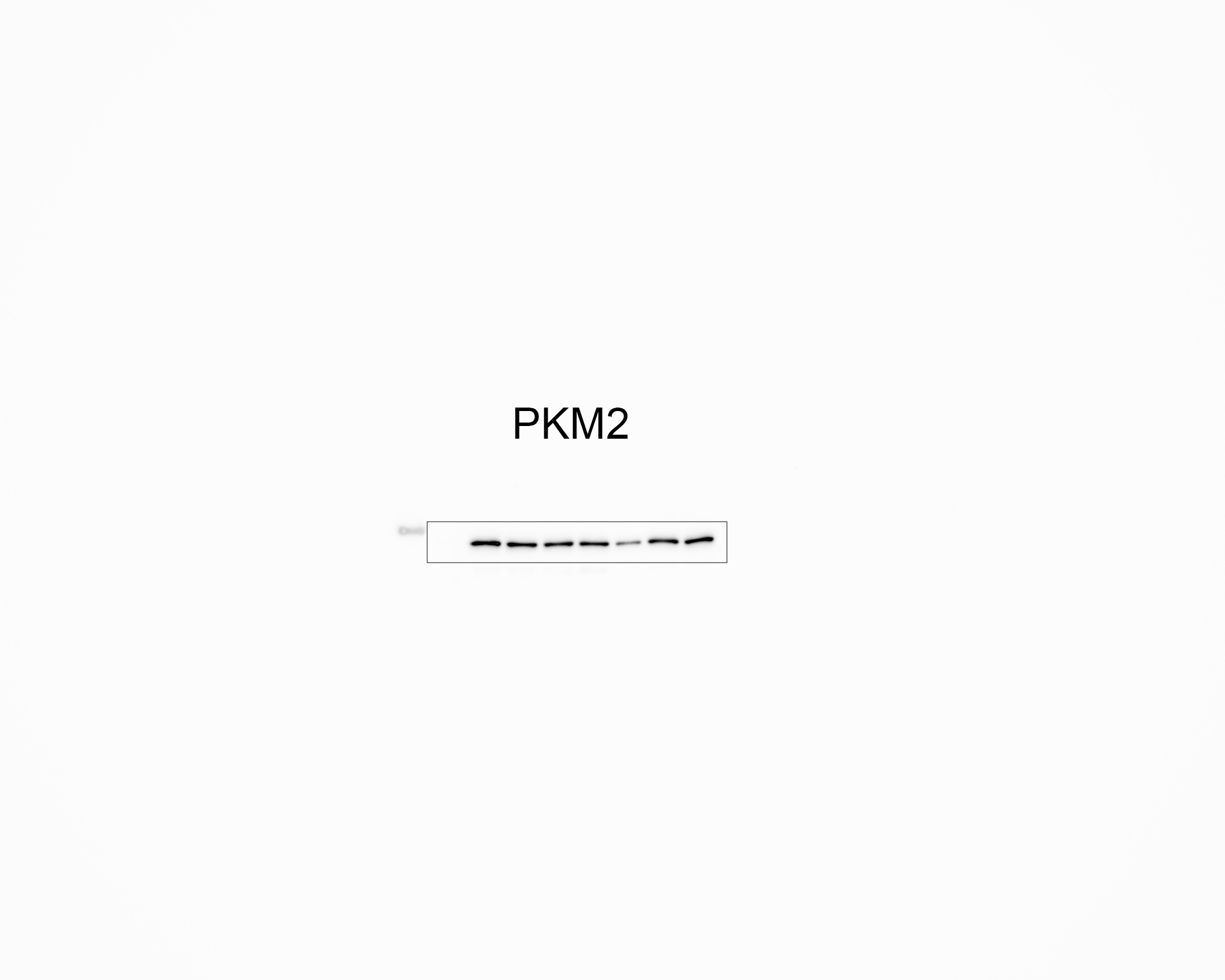

Supplement: Supplementary file 5 — Source data Fig. 3 [file 44318_2024_110_MOESM5_ESM.zip › Figure 3/3C/2-PKM2.tif]

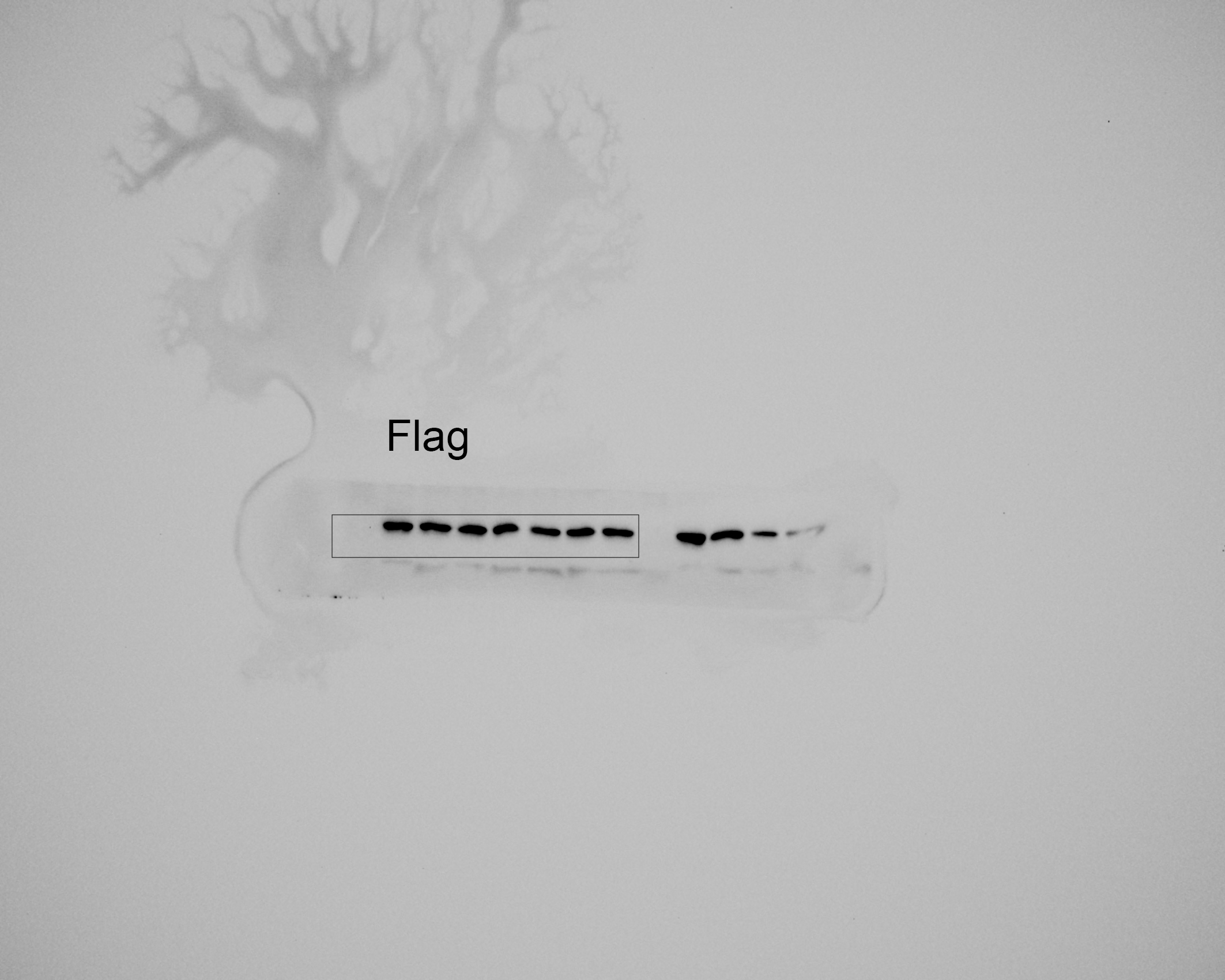

Supplement: Supplementary file 5 — Source data Fig. 3 [file 44318_2024_110_MOESM5_ESM.zip › Figure 3/3C/3-Flag.tif]

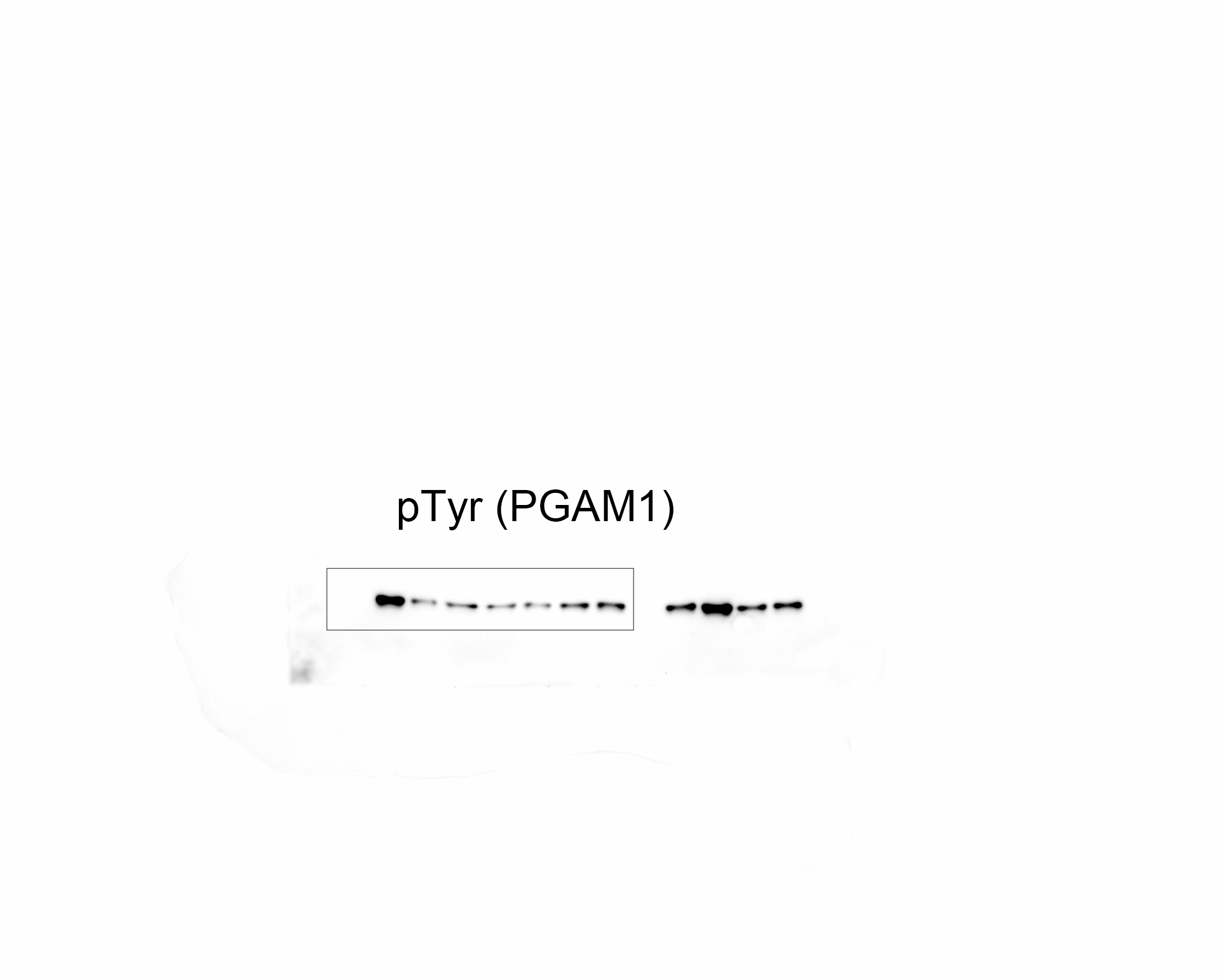

Supplement: Supplementary file 5 — Source data Fig. 3 [file 44318_2024_110_MOESM5_ESM.zip › Figure 3/3C/1-pTyr (PGAM1).tif]

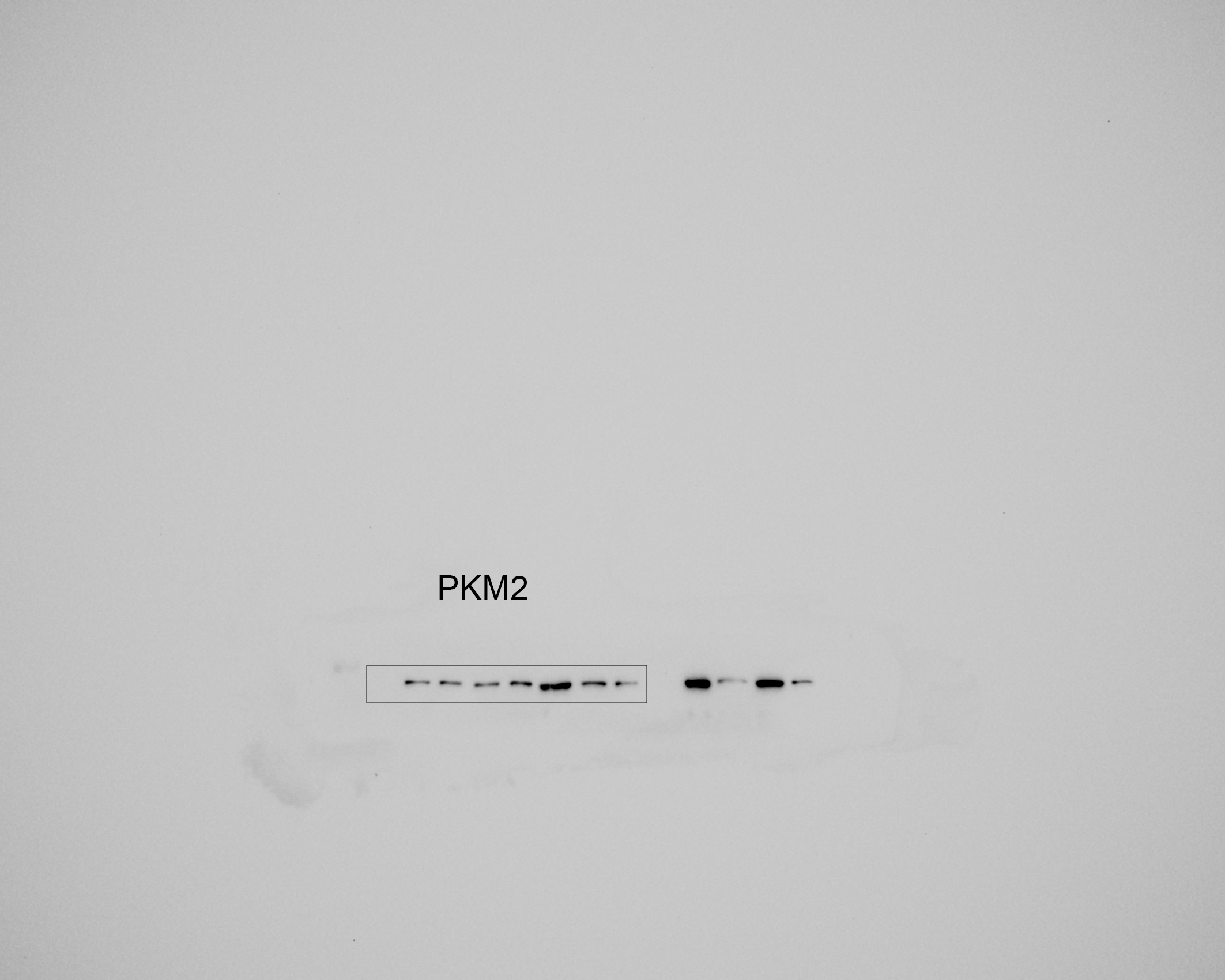

Supplement: Supplementary file 5 — Source data Fig. 3 [file 44318_2024_110_MOESM5_ESM.zip › Figure 3/3D/2-PKM2.tif]

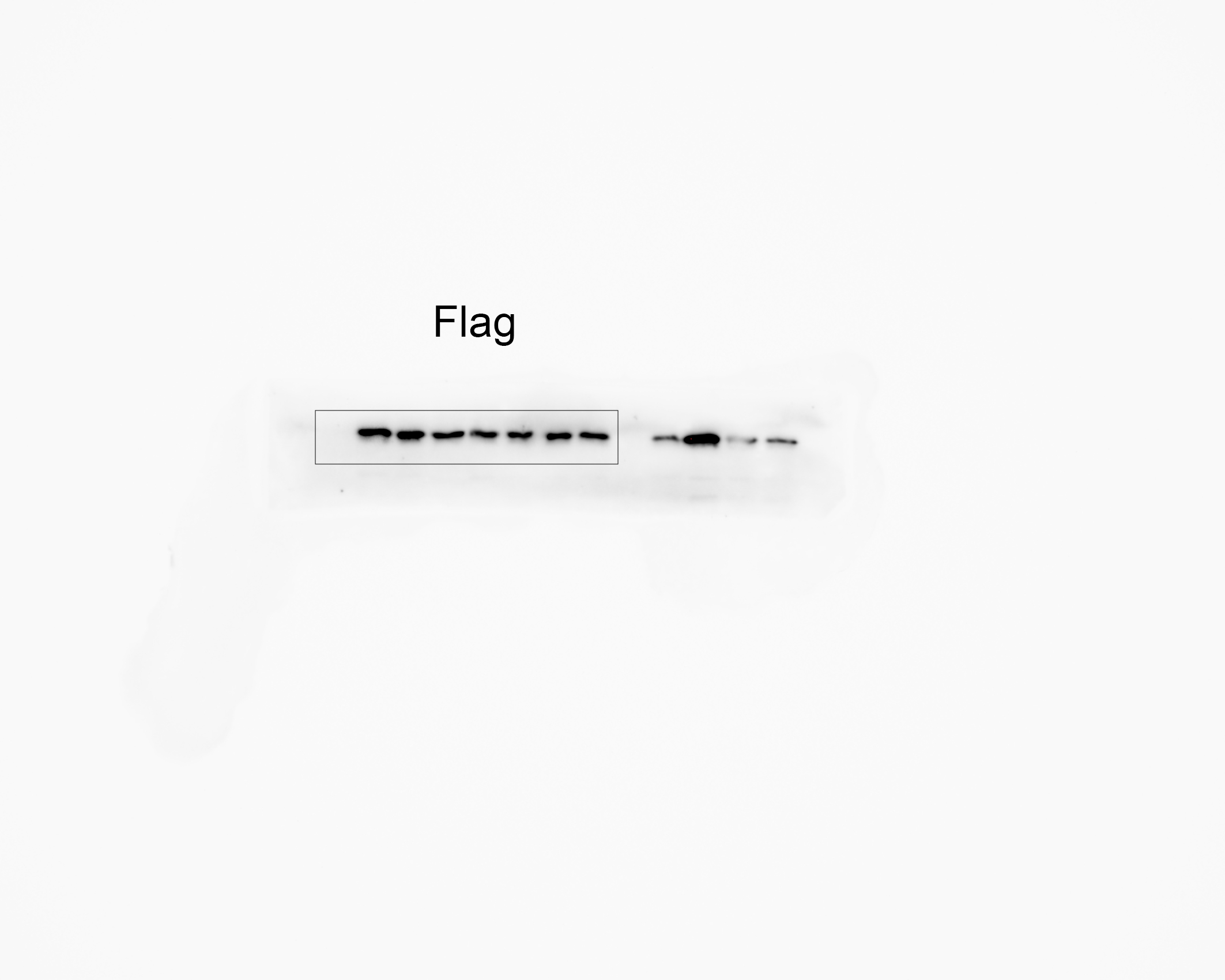

Supplement: Supplementary file 5 — Source data Fig. 3 [file 44318_2024_110_MOESM5_ESM.zip › Figure 3/3D/3-Flag.tif]

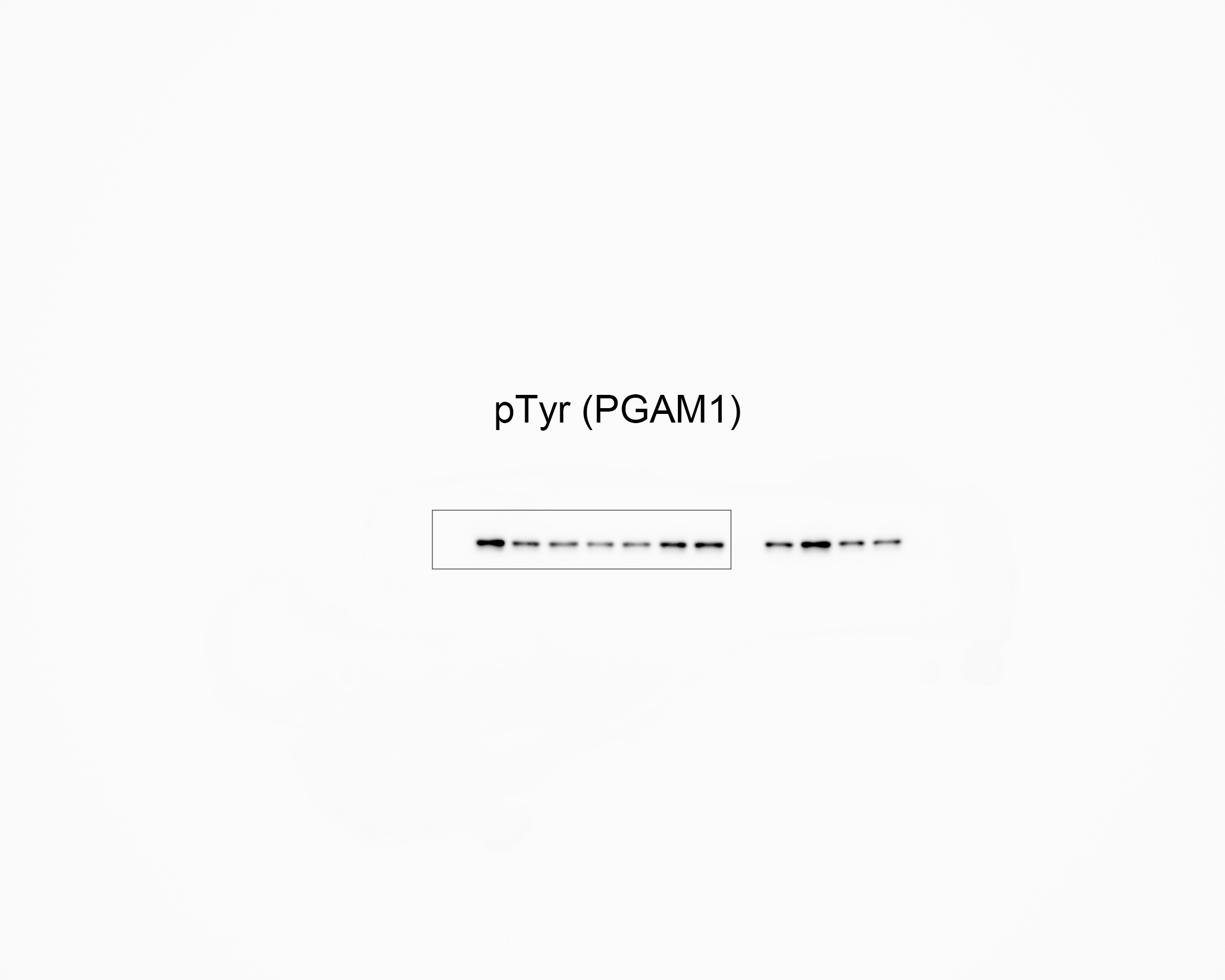

Supplement: Supplementary file 5 — Source data Fig. 3 [file 44318_2024_110_MOESM5_ESM.zip › Figure 3/3D/1-pTyr (PGAM1).tif]

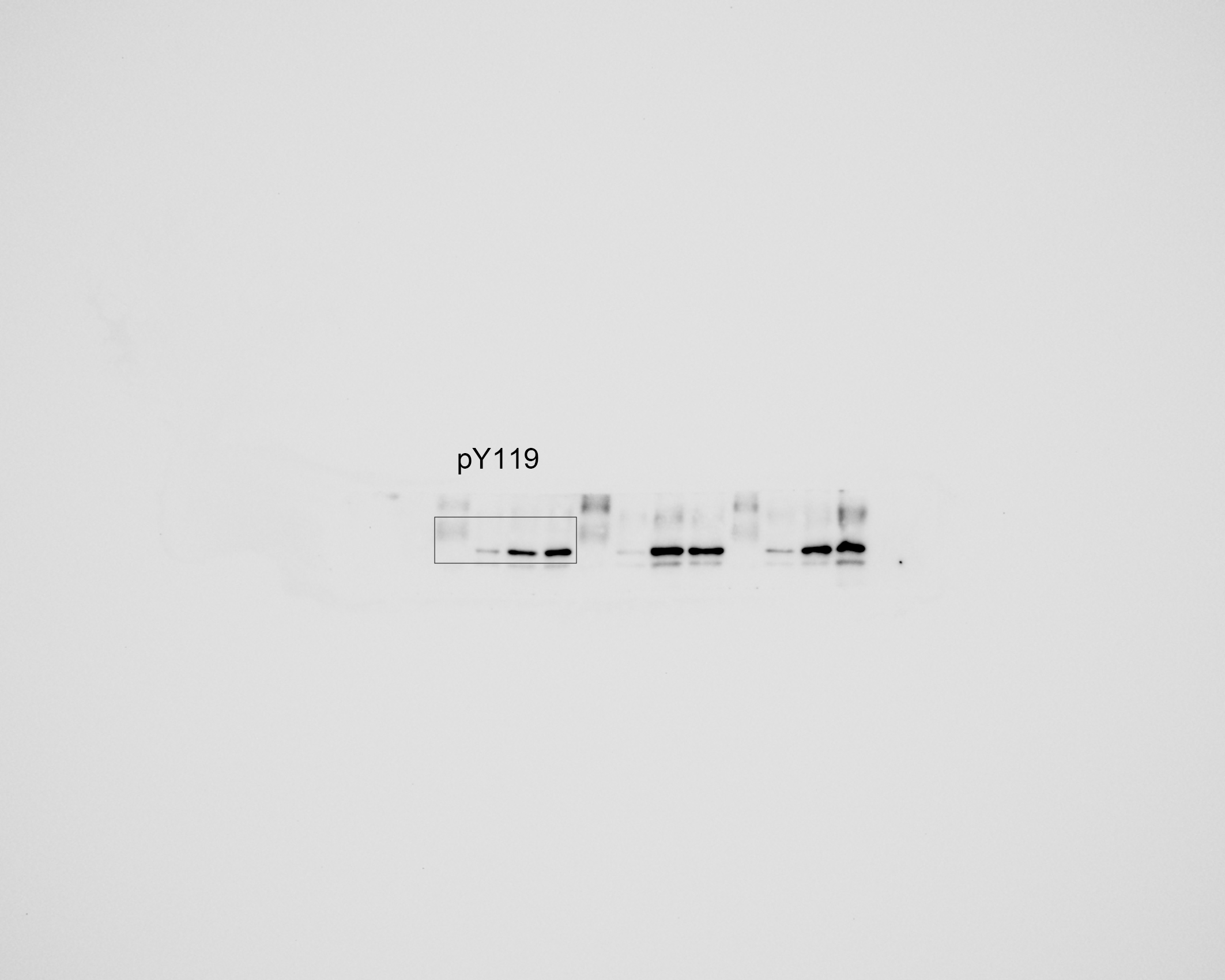

Supplement: Supplementary file 5 — Source data Fig. 3 [file 44318_2024_110_MOESM5_ESM.zip › Figure 3/3H/1-pY119.tif]

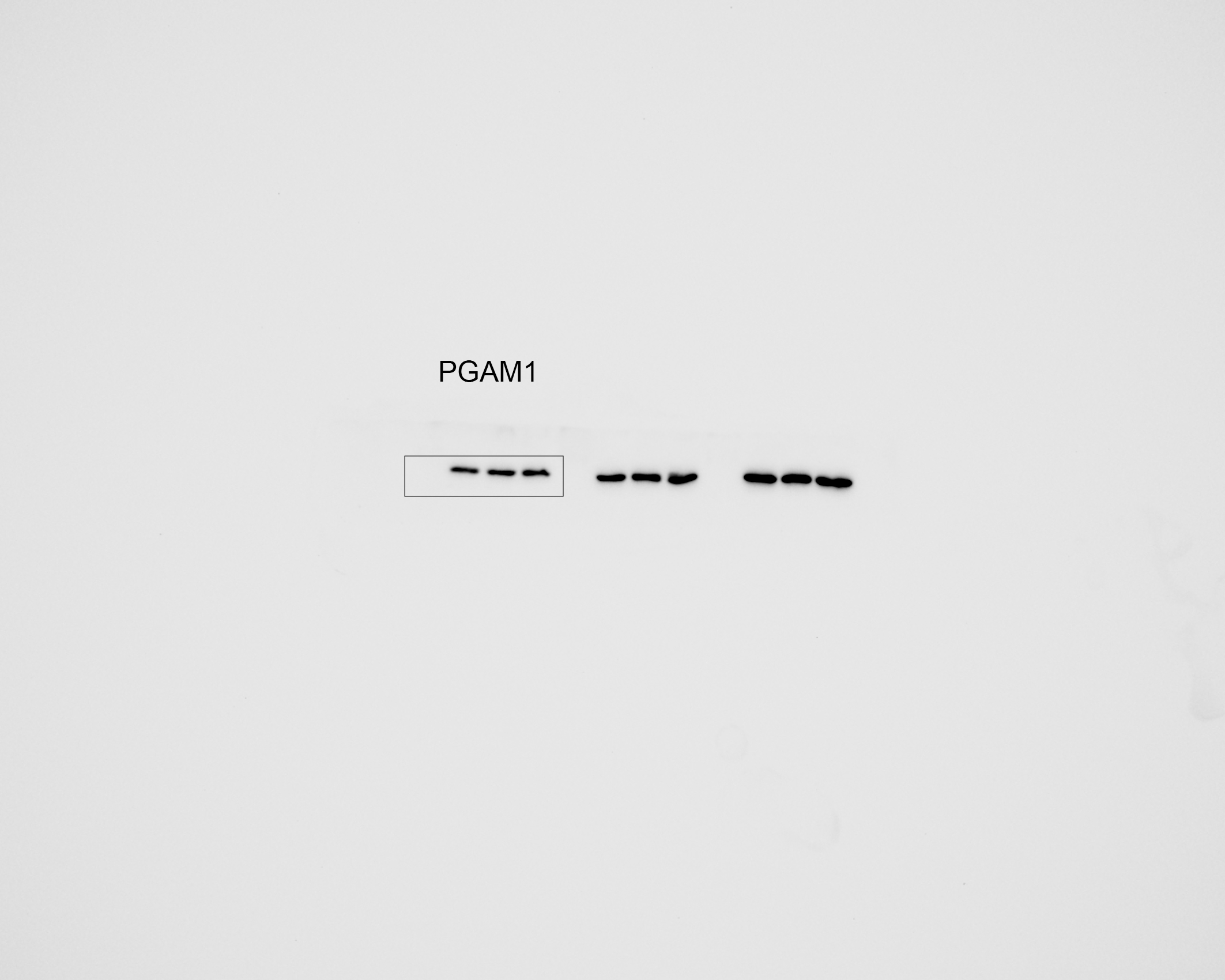

Supplement: Supplementary file 5 — Source data Fig. 3 [file 44318_2024_110_MOESM5_ESM.zip › Figure 3/3H/2-PGAM1.tif]

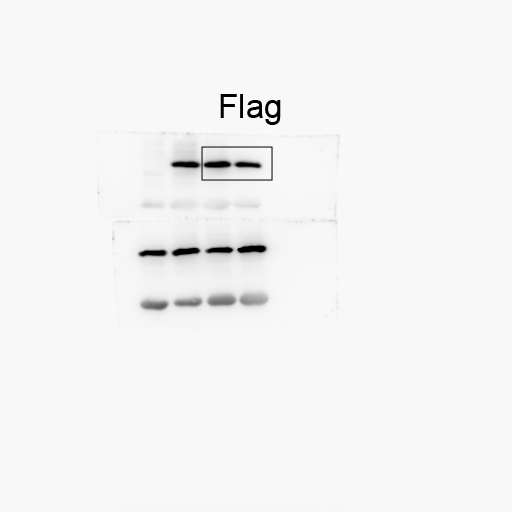

Supplement: Supplementary file 5 — Source data Fig. 3 [file 44318_2024_110_MOESM5_ESM.zip › Figure 3/3A/4-Flag (PGAM1).tif]

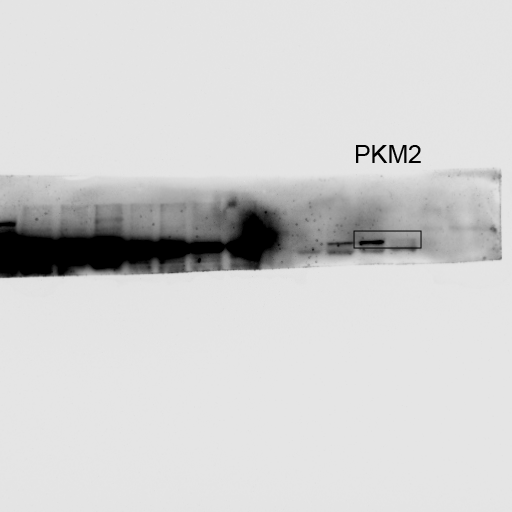

Supplement: Supplementary file 5 — Source data Fig. 3 [file 44318_2024_110_MOESM5_ESM.zip › Figure 3/3A/2-PKM2.tif]

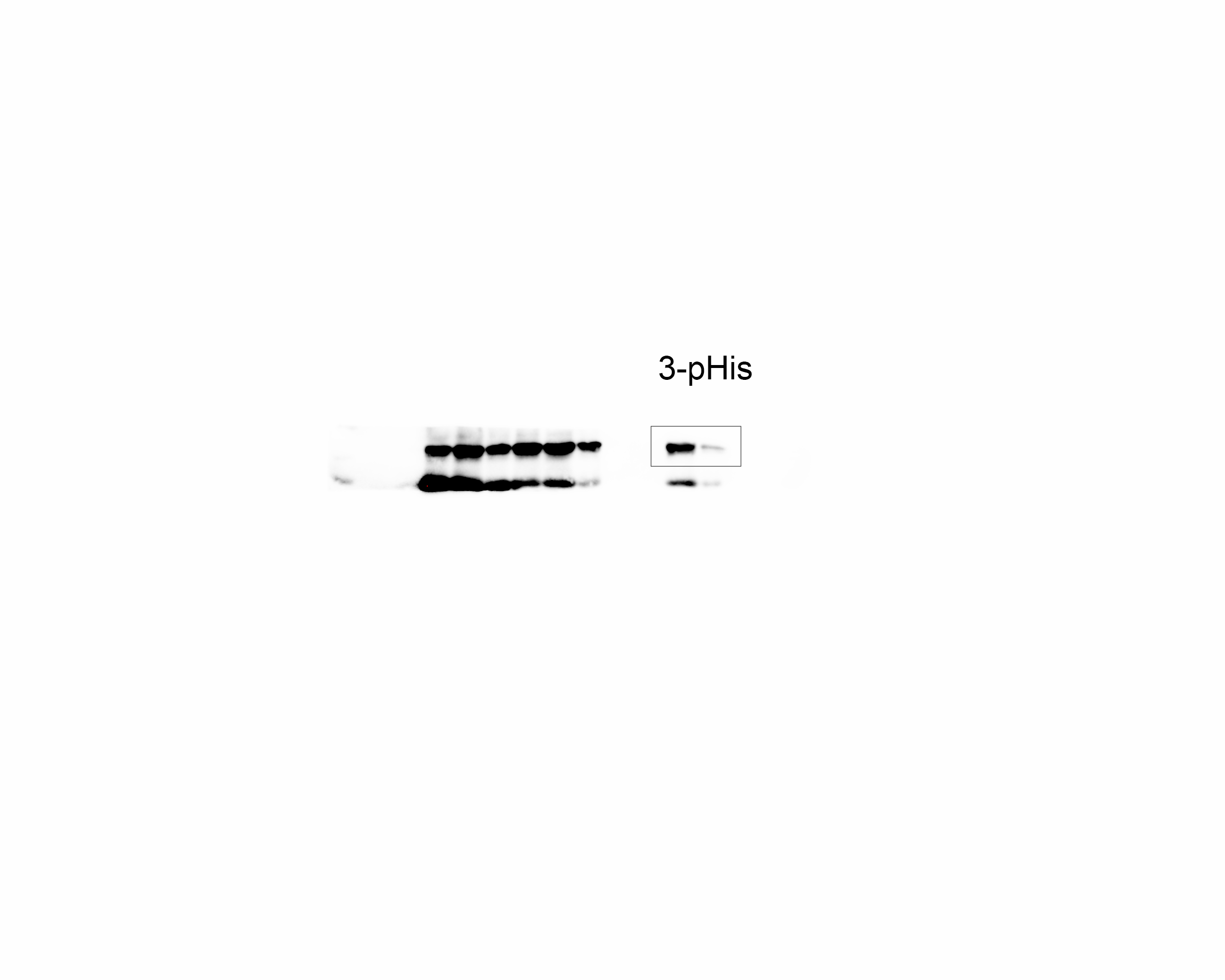

Supplement: Supplementary file 5 — Source data Fig. 3 [file 44318_2024_110_MOESM5_ESM.zip › Figure 3/3A/3-3-pHis.tif]

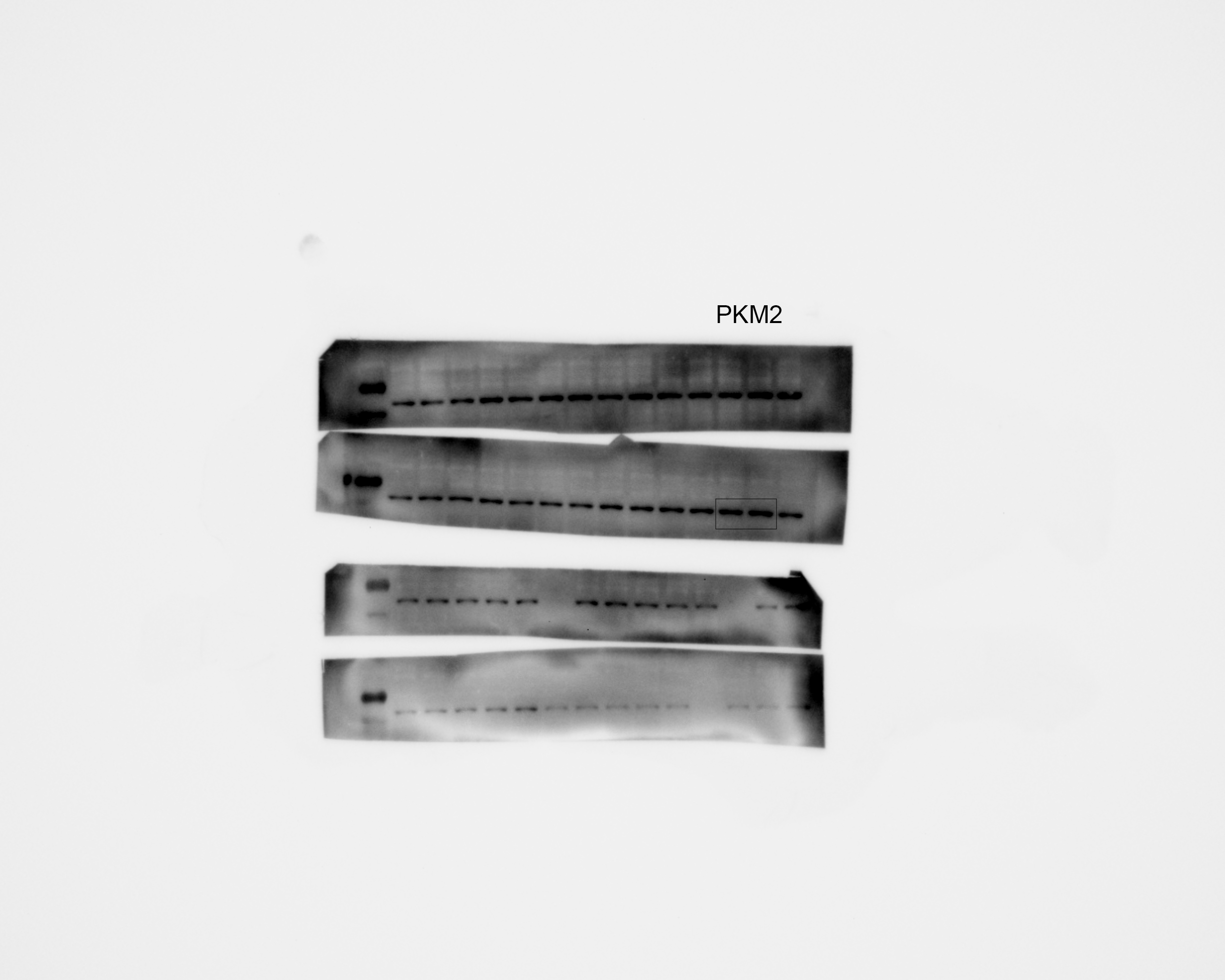

Supplement: Supplementary file 5 — Source data Fig. 3 [file 44318_2024_110_MOESM5_ESM.zip › Figure 3/3A/5-PKM2.tif]

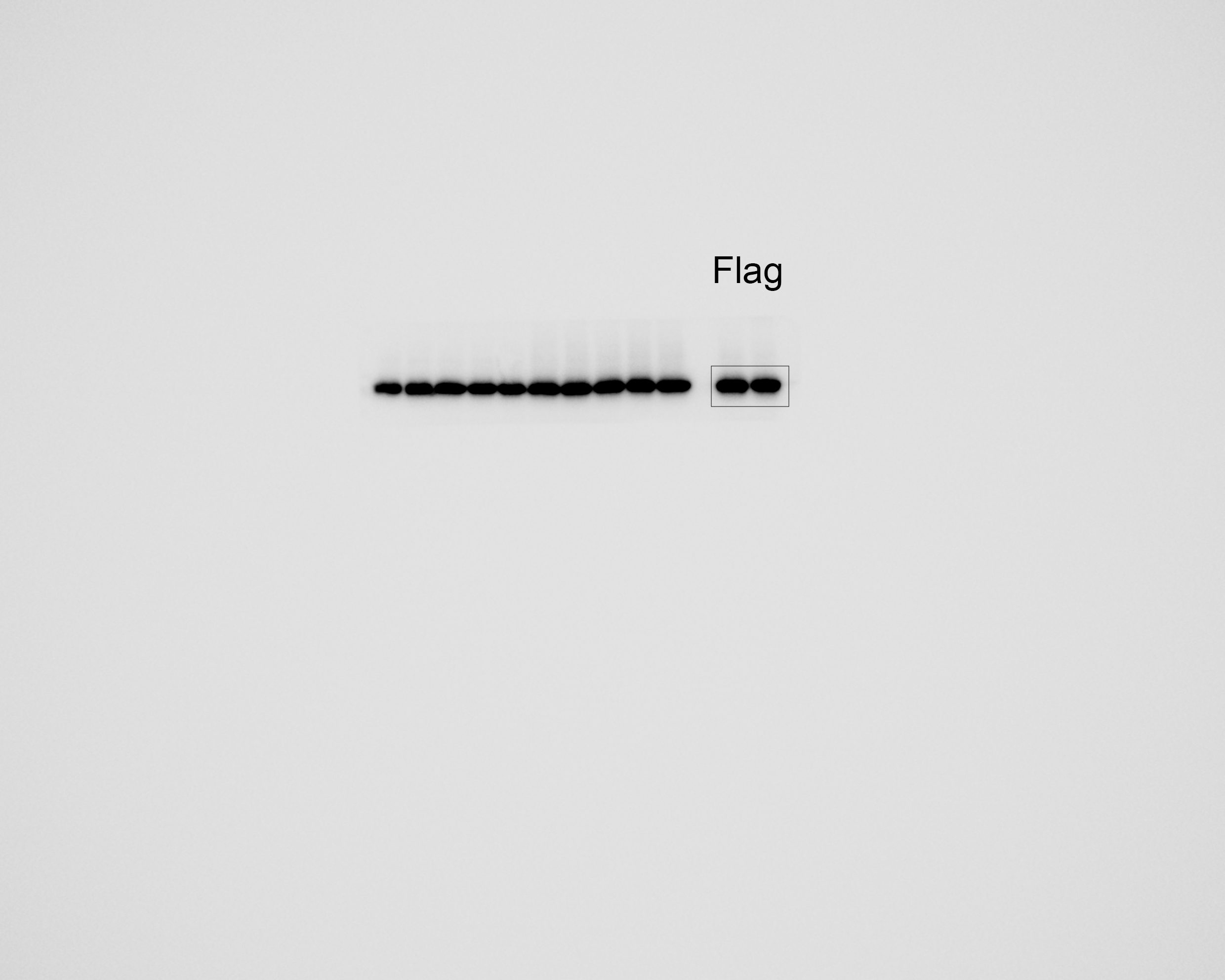

Supplement: Supplementary file 5 — Source data Fig. 3 [file 44318_2024_110_MOESM5_ESM.zip › Figure 3/3A/6-Flag (PGAM1).tif]

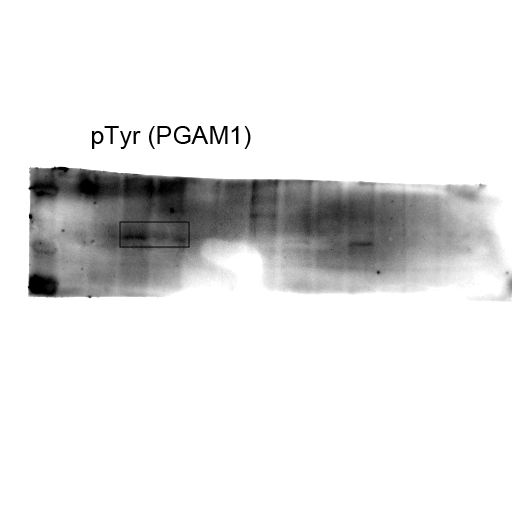

Supplement: Supplementary file 5 — Source data Fig. 3 [file 44318_2024_110_MOESM5_ESM.zip › Figure 3/3A/1-pTyr (PGAM1).tif]

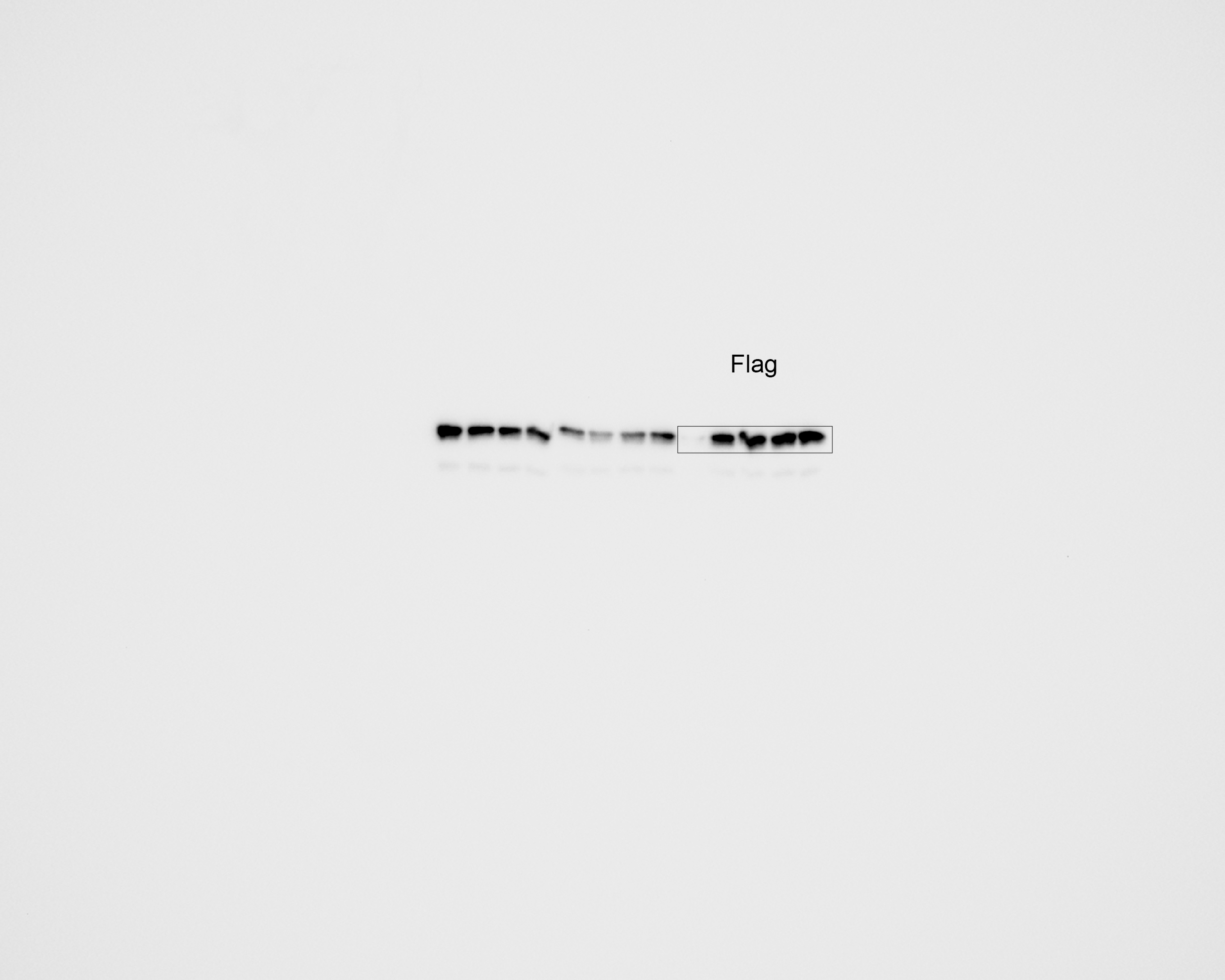

Supplement: Supplementary file 5 — Source data Fig. 3 [file 44318_2024_110_MOESM5_ESM.zip › Figure 3/3I/4-Flag.tif]

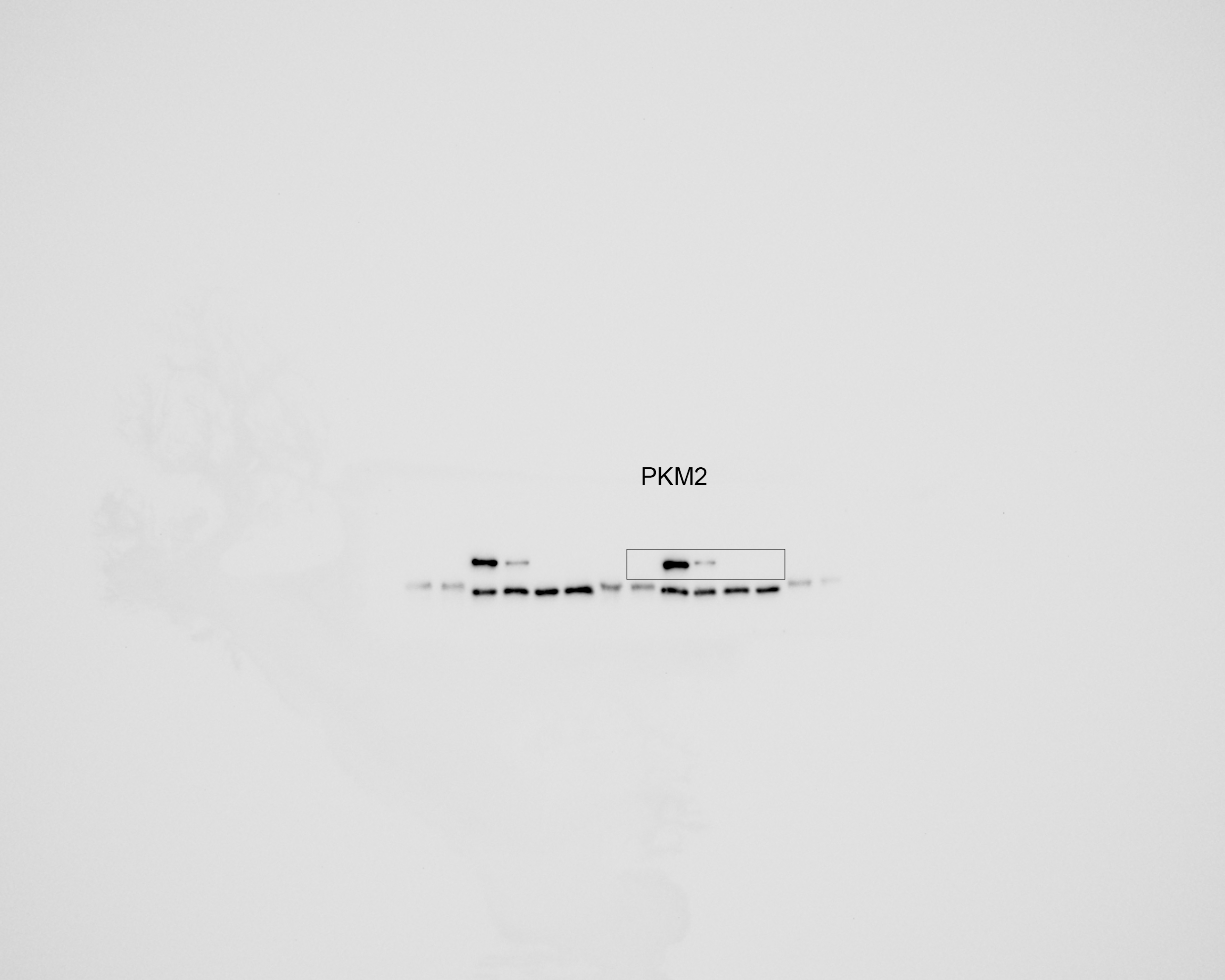

Supplement: Supplementary file 5 — Source data Fig. 3 [file 44318_2024_110_MOESM5_ESM.zip › Figure 3/3I/2-PKM2.tif]

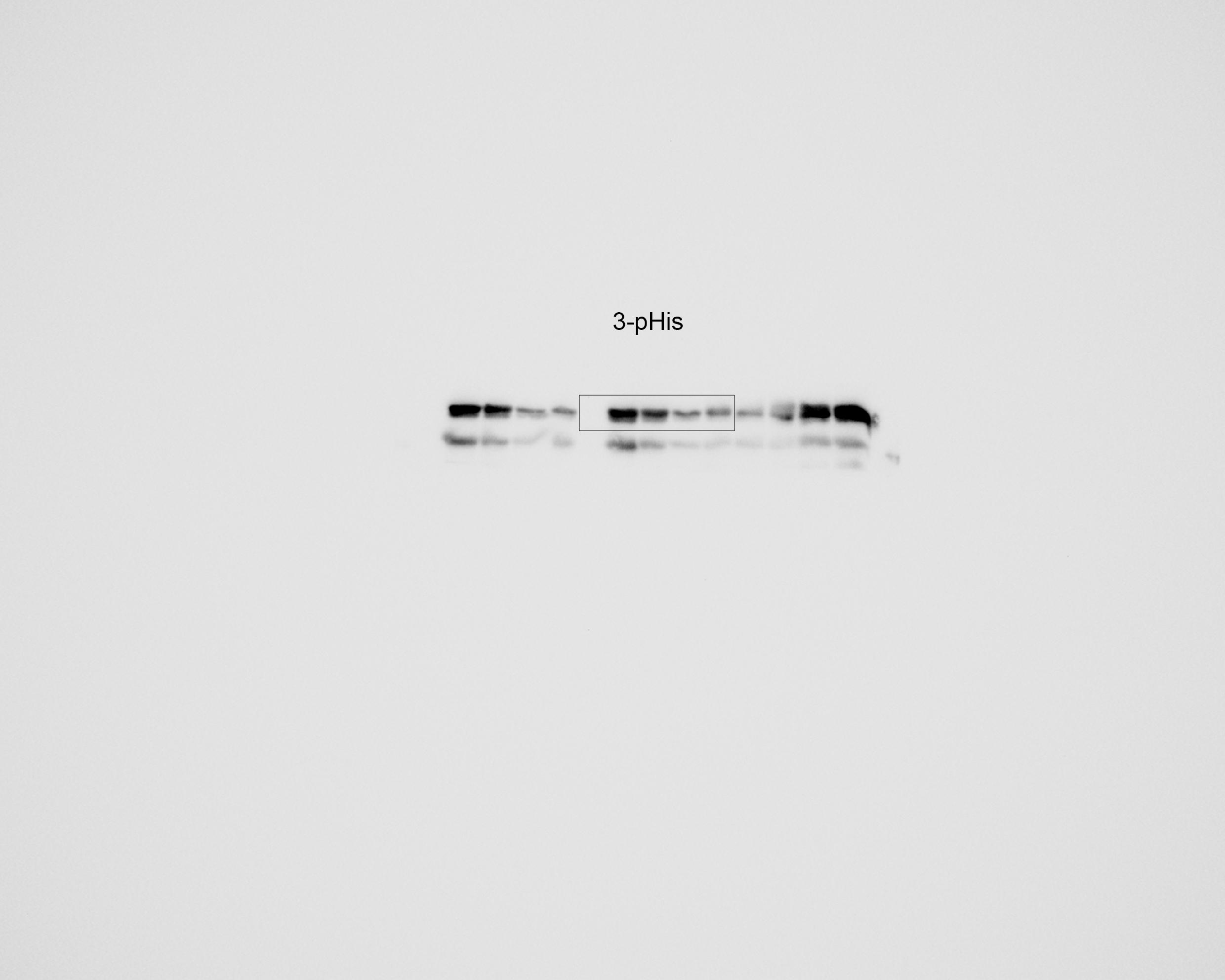

Supplement: Supplementary file 5 — Source data Fig. 3 [file 44318_2024_110_MOESM5_ESM.zip › Figure 3/3I/3-3-pHis.tif]

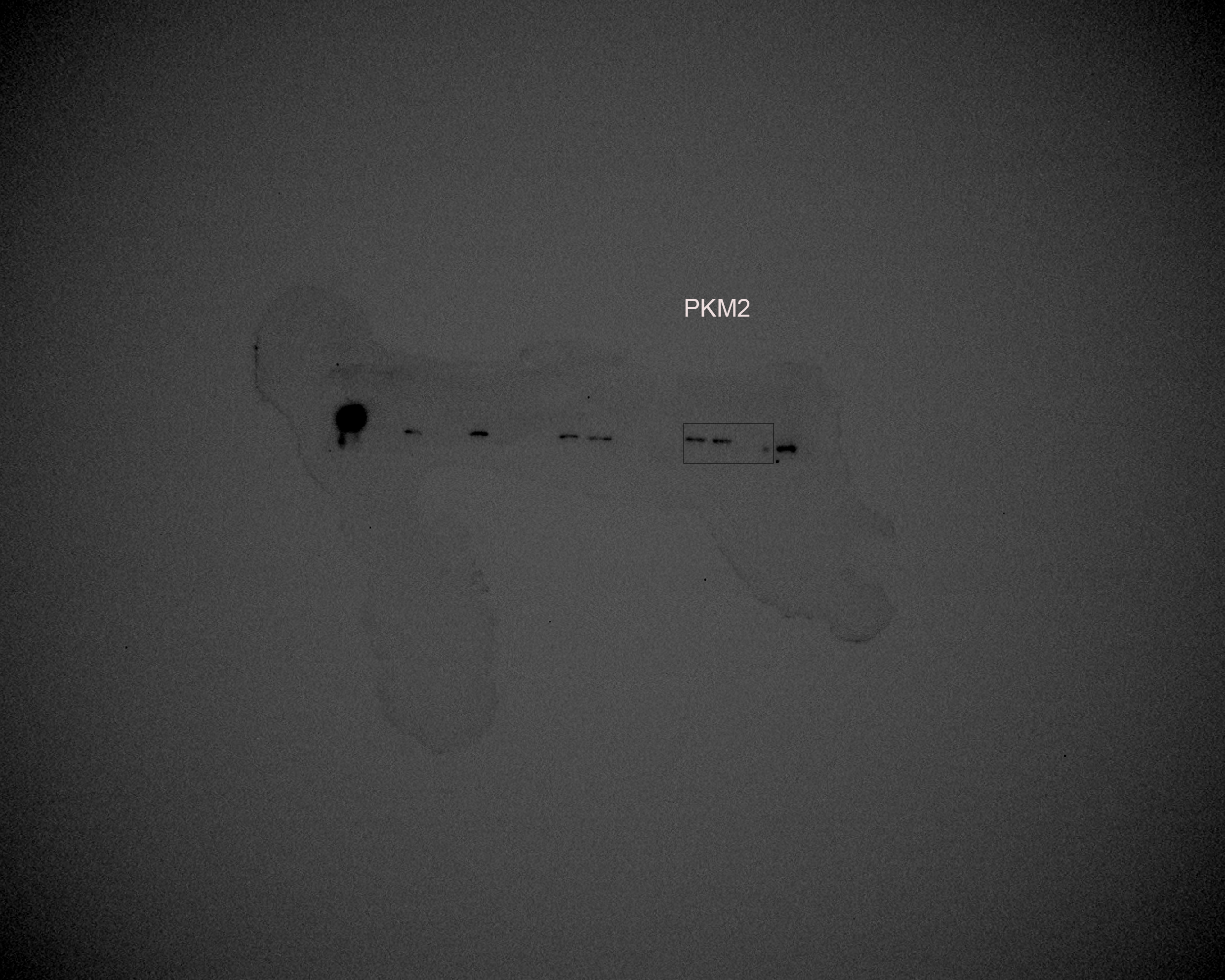

Supplement: Supplementary file 5 — Source data Fig. 3 [file 44318_2024_110_MOESM5_ESM.zip › Figure 3/3I/5-PKM2.tif]

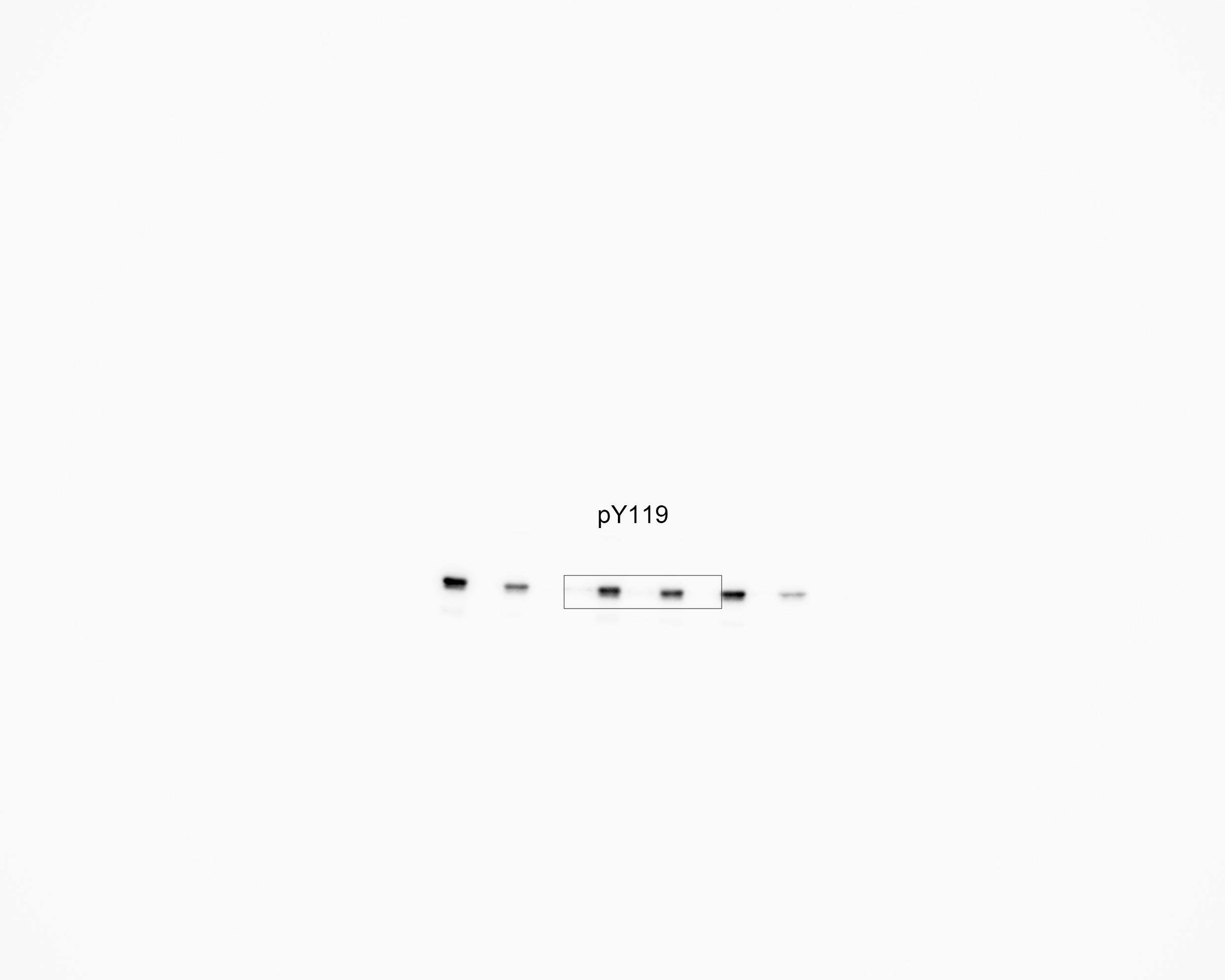

Supplement: Supplementary file 5 — Source data Fig. 3 [file 44318_2024_110_MOESM5_ESM.zip › Figure 3/3I/1-pY119.tif]

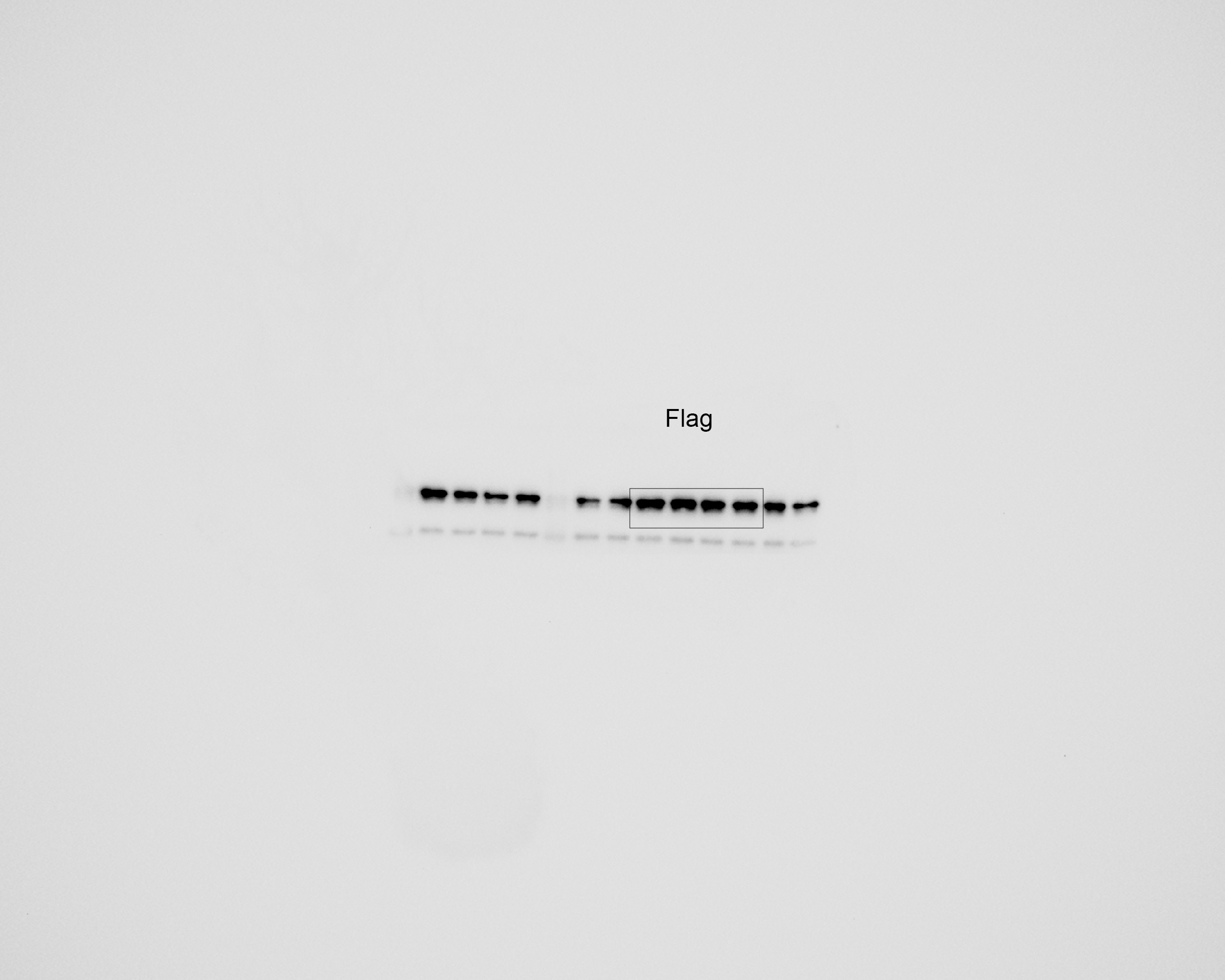

Supplement: Supplementary file 5 — Source data Fig. 3 [file 44318_2024_110_MOESM5_ESM.zip › Figure 3/3I/6-Flag.tif]

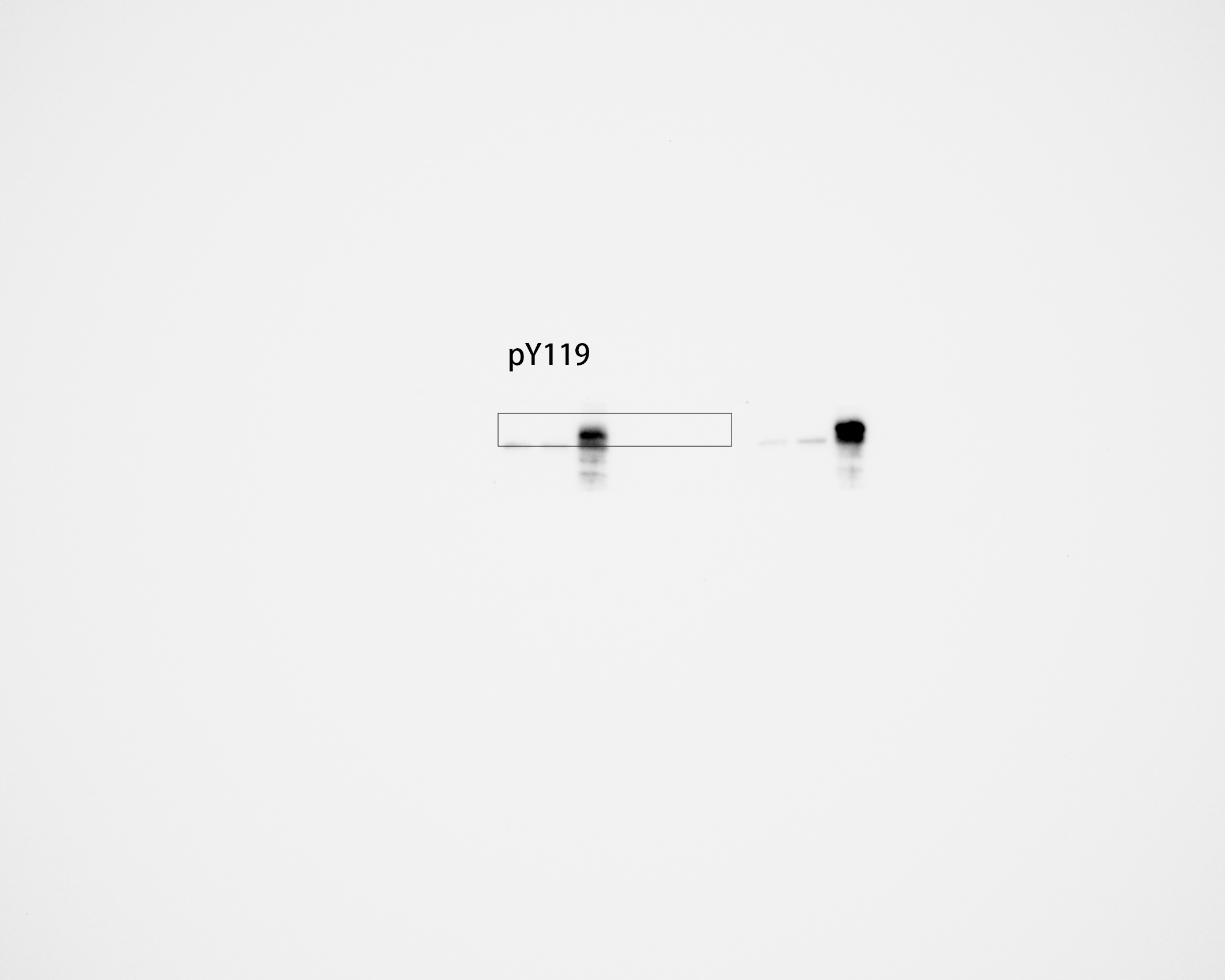

Supplement: Supplementary file 6 — Source data Fig. 4 [file 44318_2024_110_MOESM6_ESM.zip › Figure 4/4E/1-pY119.tif]

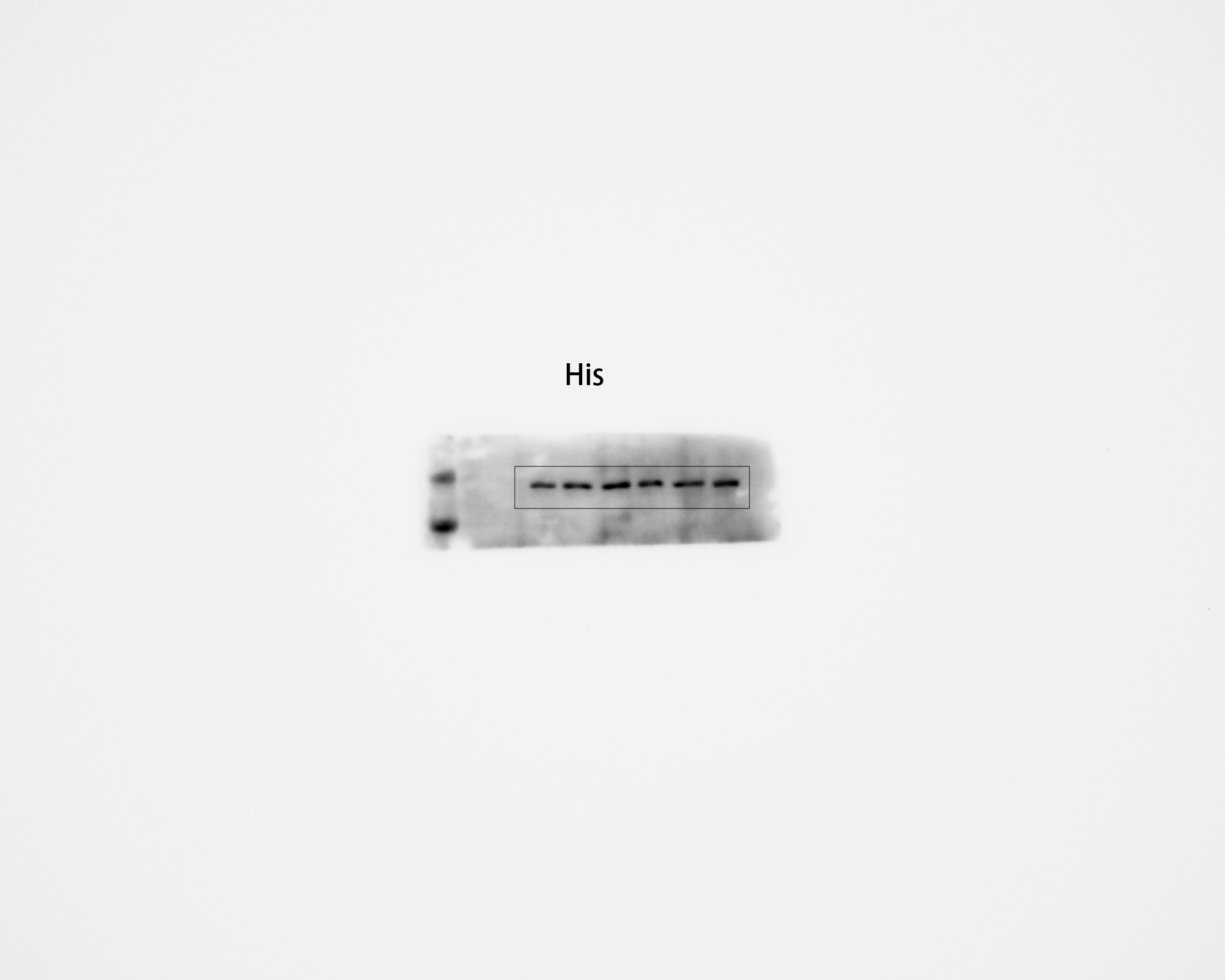

Supplement: Supplementary file 6 — Source data Fig. 4 [file 44318_2024_110_MOESM6_ESM.zip › Figure 4/4E/3-His.tif]

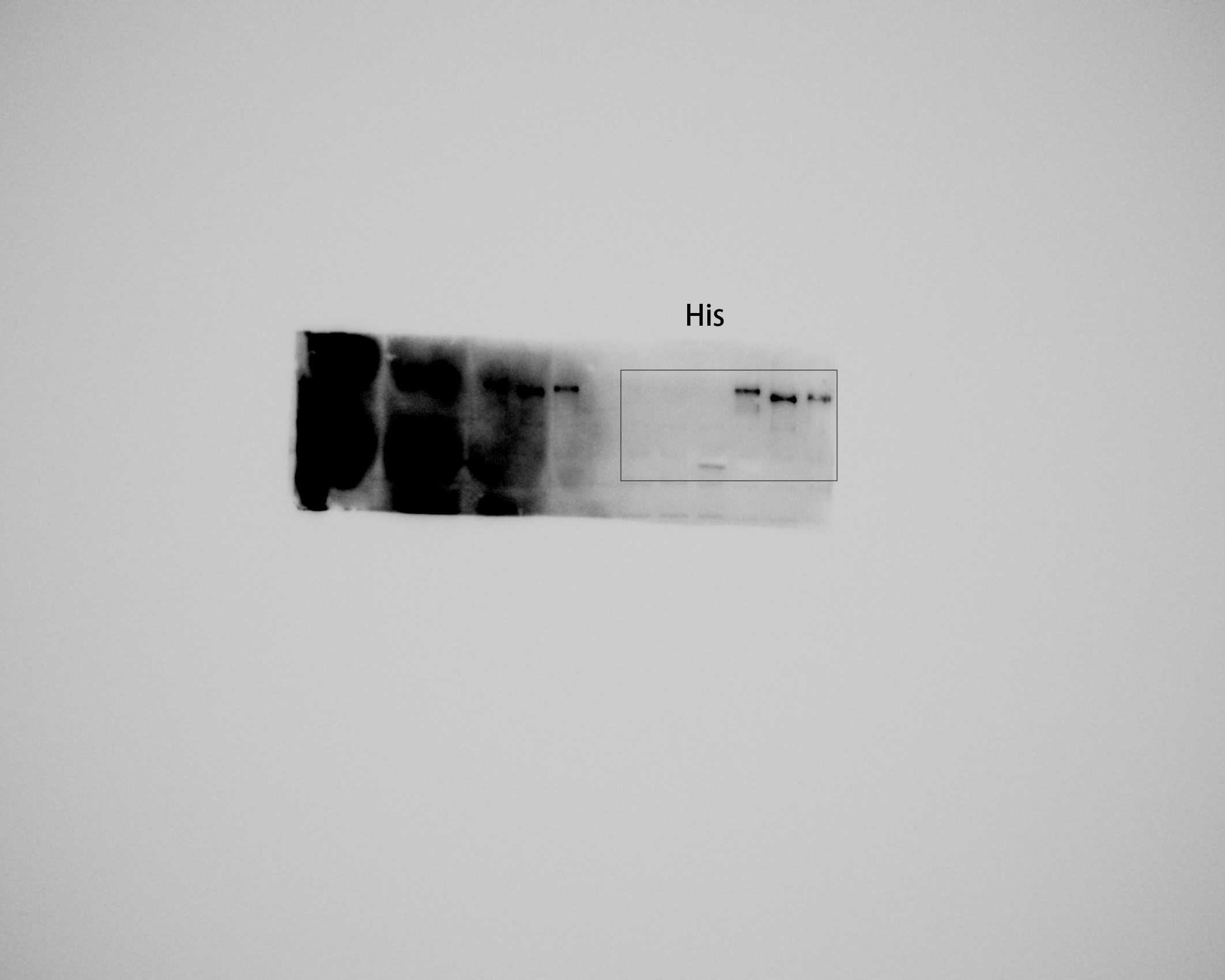

Supplement: Supplementary file 6 — Source data Fig. 4 [file 44318_2024_110_MOESM6_ESM.zip › Figure 4/4E/2-His.tif]

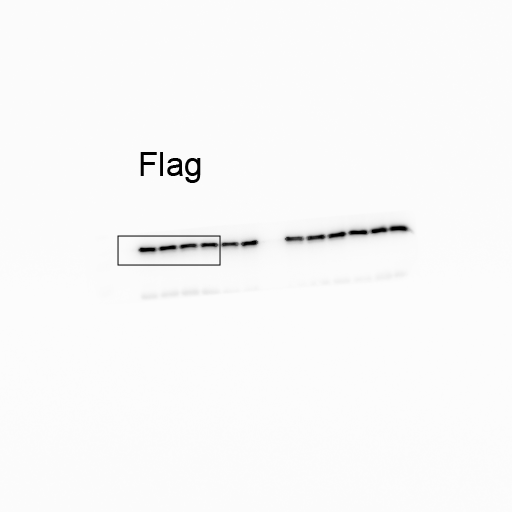

Supplement: Supplementary file 6 — Source data Fig. 4 [file 44318_2024_110_MOESM6_ESM.zip › Figure 4/4B/4-Flag.tif]

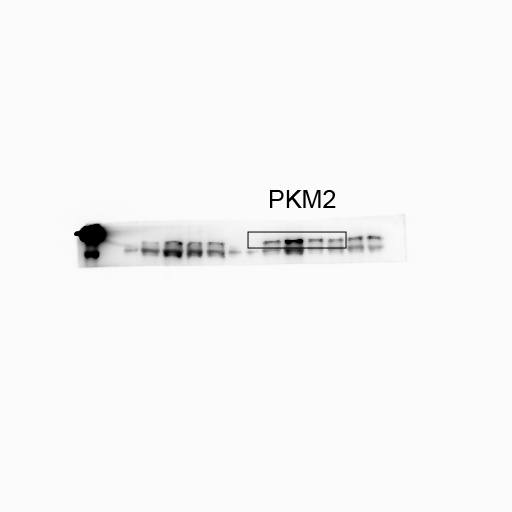

Supplement: Supplementary file 6 — Source data Fig. 4 [file 44318_2024_110_MOESM6_ESM.zip › Figure 4/4B/2-PKM2.tif]

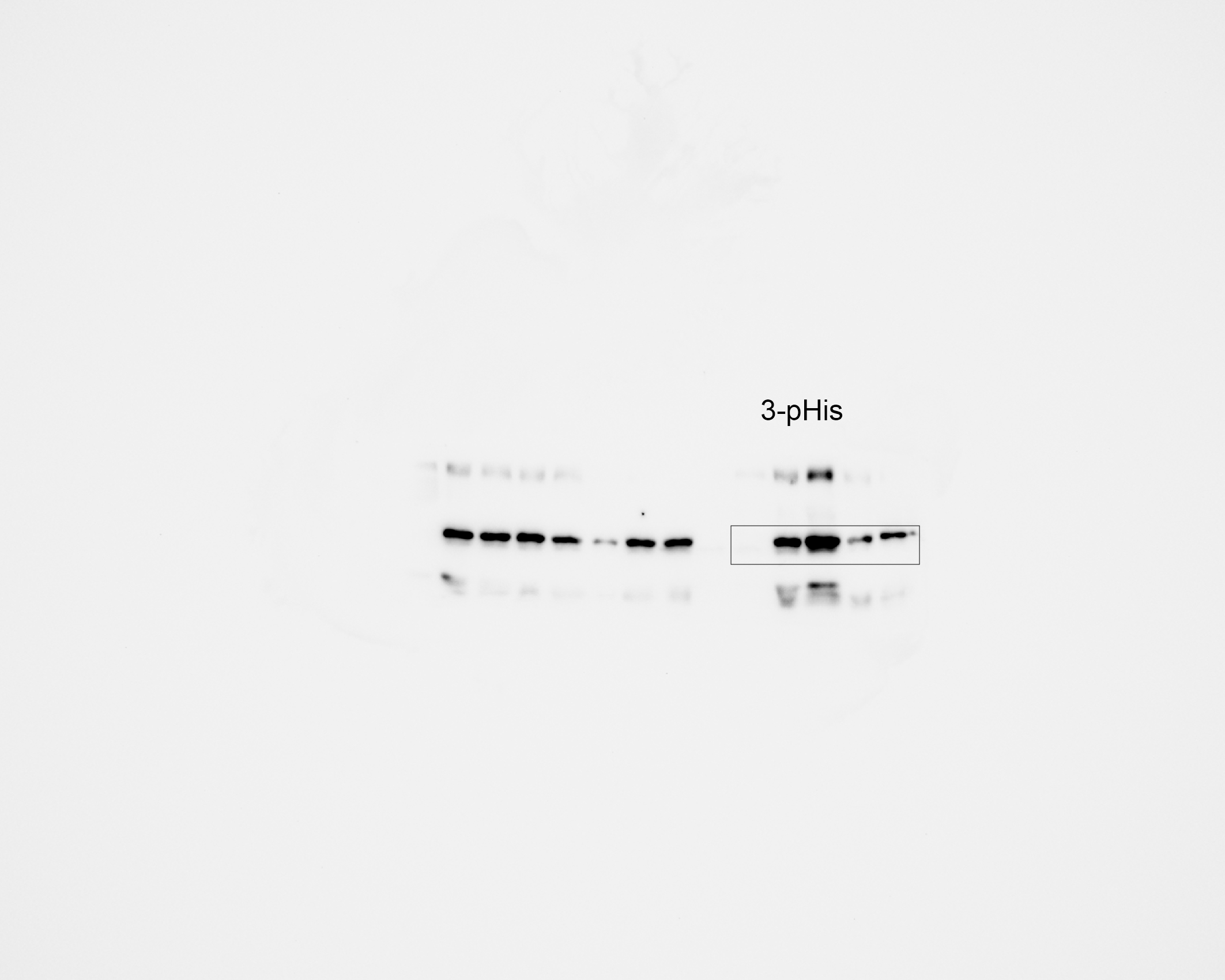

Supplement: Supplementary file 6 — Source data Fig. 4 [file 44318_2024_110_MOESM6_ESM.zip › Figure 4/4B/3-3-pHis.tif]

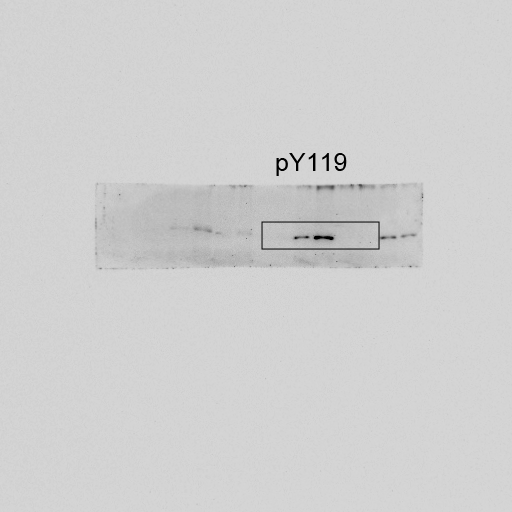

Supplement: Supplementary file 6 — Source data Fig. 4 [file 44318_2024_110_MOESM6_ESM.zip › Figure 4/4B/1-pY119.tif]

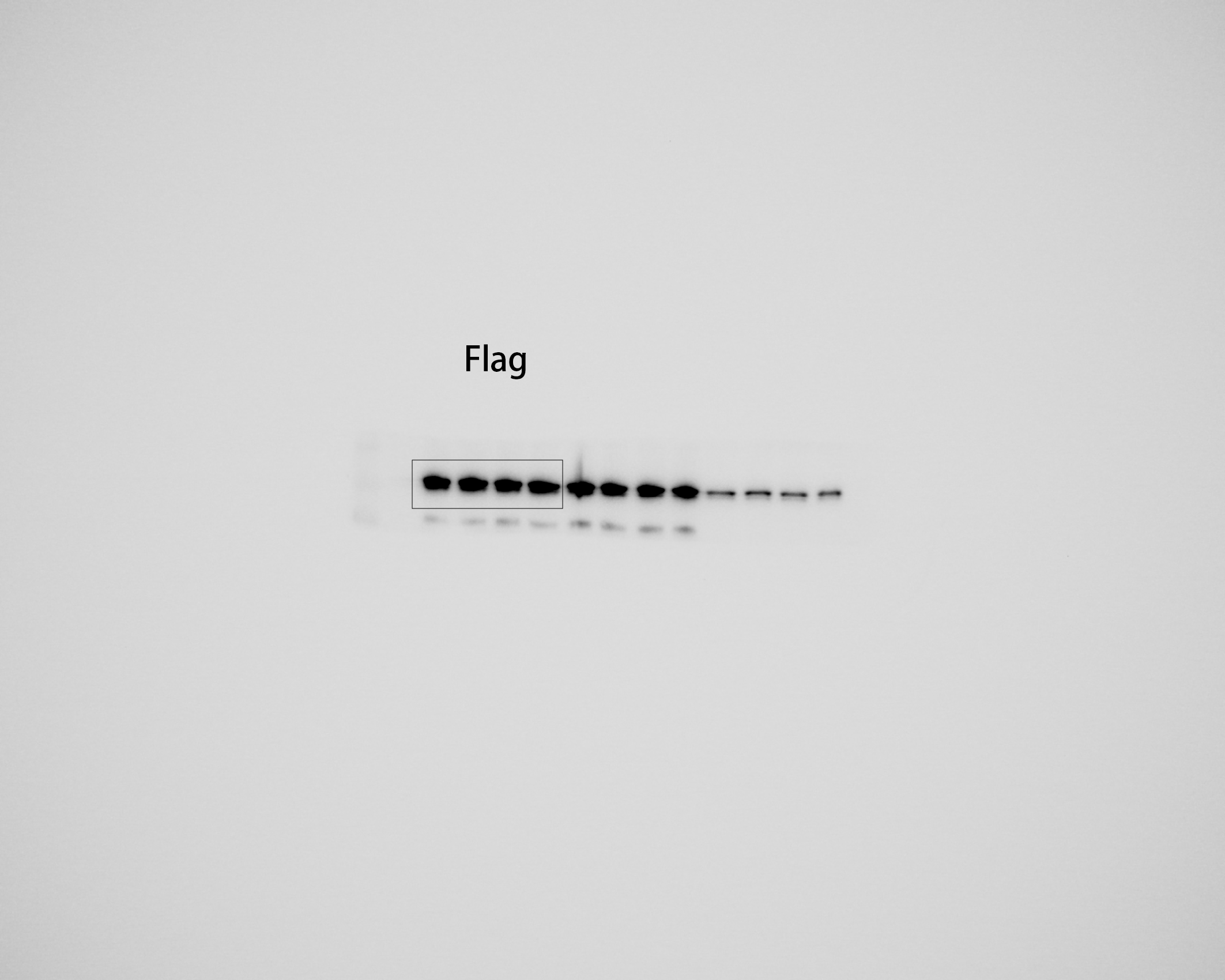

Supplement: Supplementary file 6 — Source data Fig. 4 [file 44318_2024_110_MOESM6_ESM.zip › Figure 4/4B/8-Flag.tif]

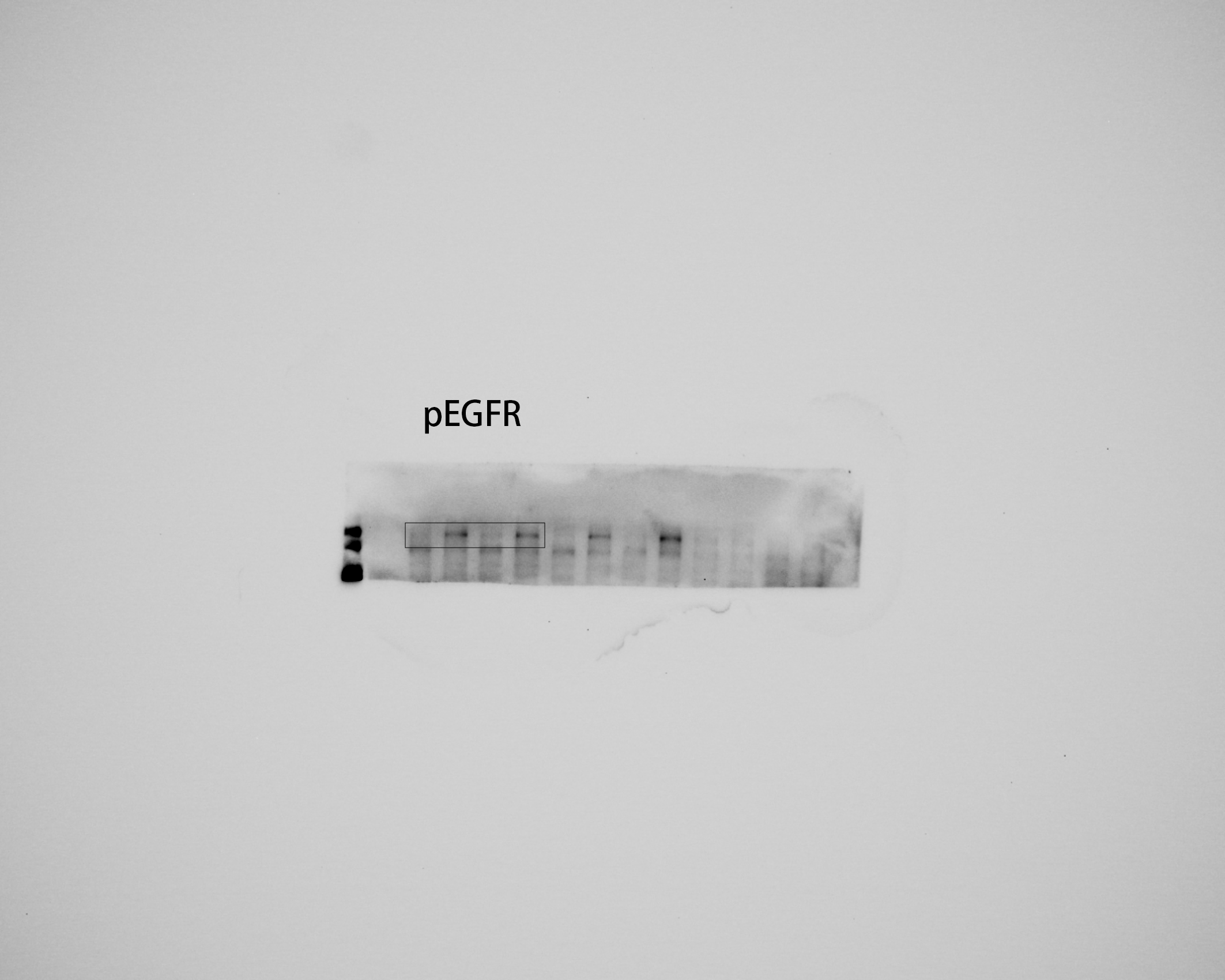

Supplement: Supplementary file 6 — Source data Fig. 4 [file 44318_2024_110_MOESM6_ESM.zip › Figure 4/4B/5-pEGFR.tif]

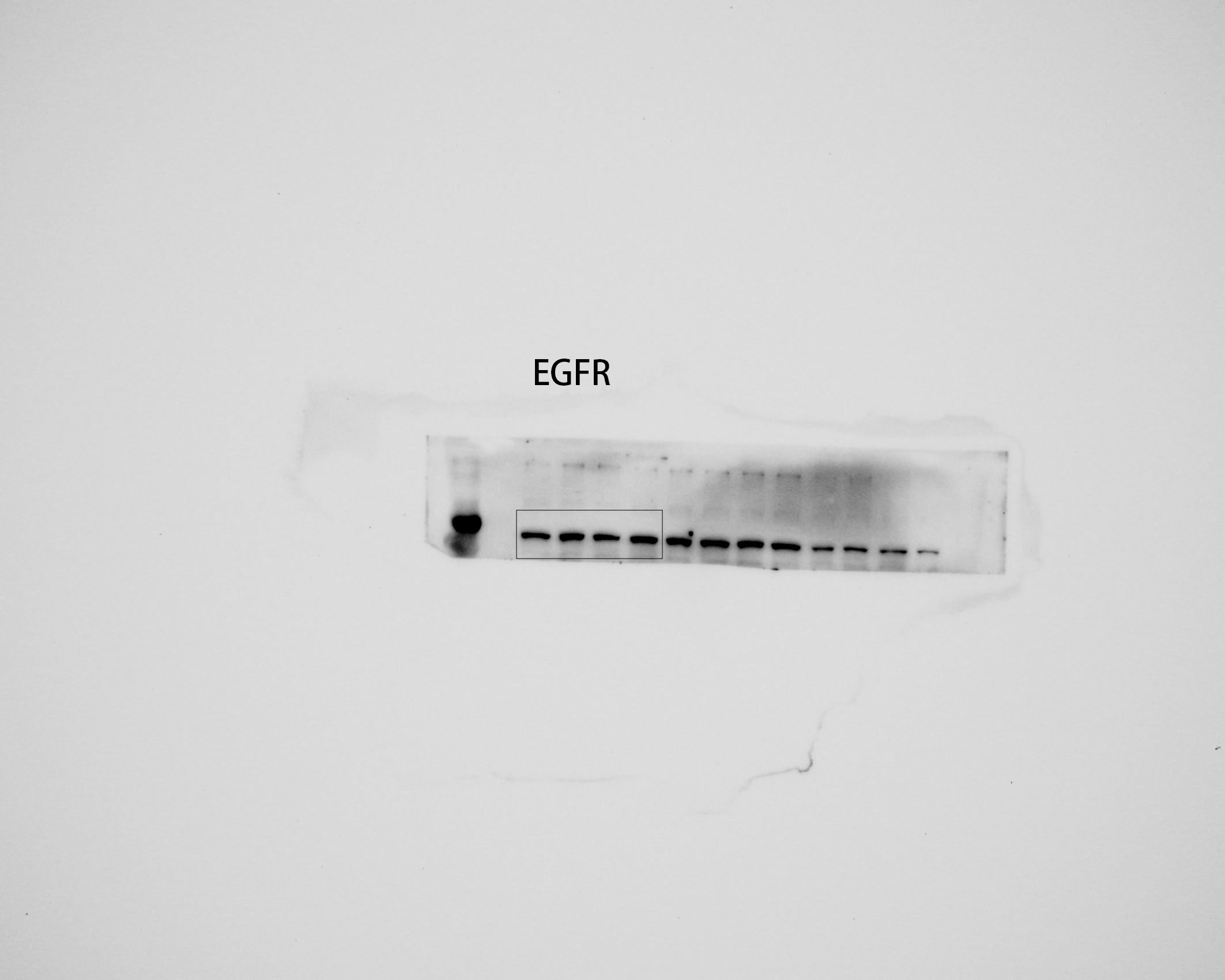

Supplement: Supplementary file 6 — Source data Fig. 4 [file 44318_2024_110_MOESM6_ESM.zip › Figure 4/4B/6-EGFR.tif]

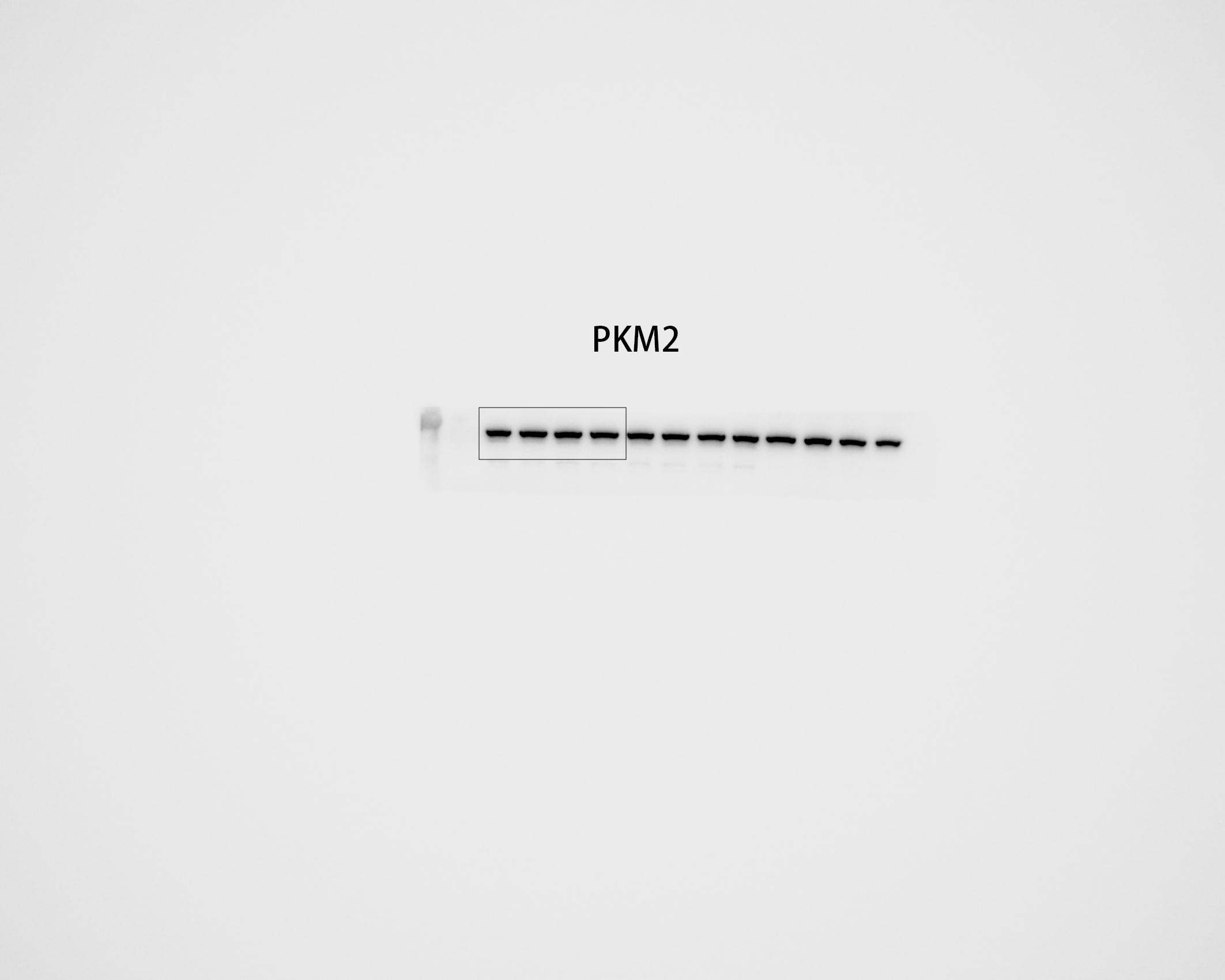

Supplement: Supplementary file 6 — Source data Fig. 4 [file 44318_2024_110_MOESM6_ESM.zip › Figure 4/4B/7-PKM2.tif]

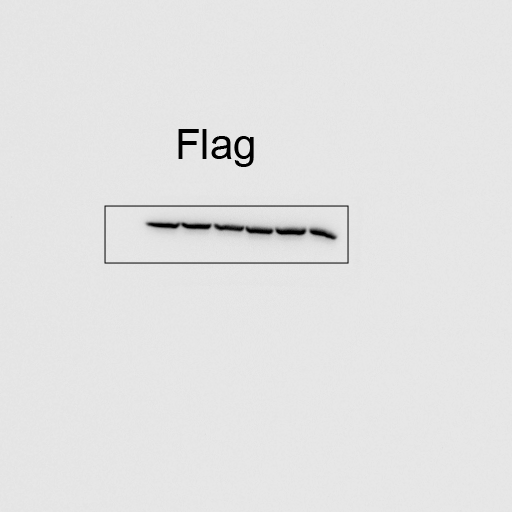

Supplement: Supplementary file 6 — Source data Fig. 4 [file 44318_2024_110_MOESM6_ESM.zip › Figure 4/4C/4-Flag.tif]

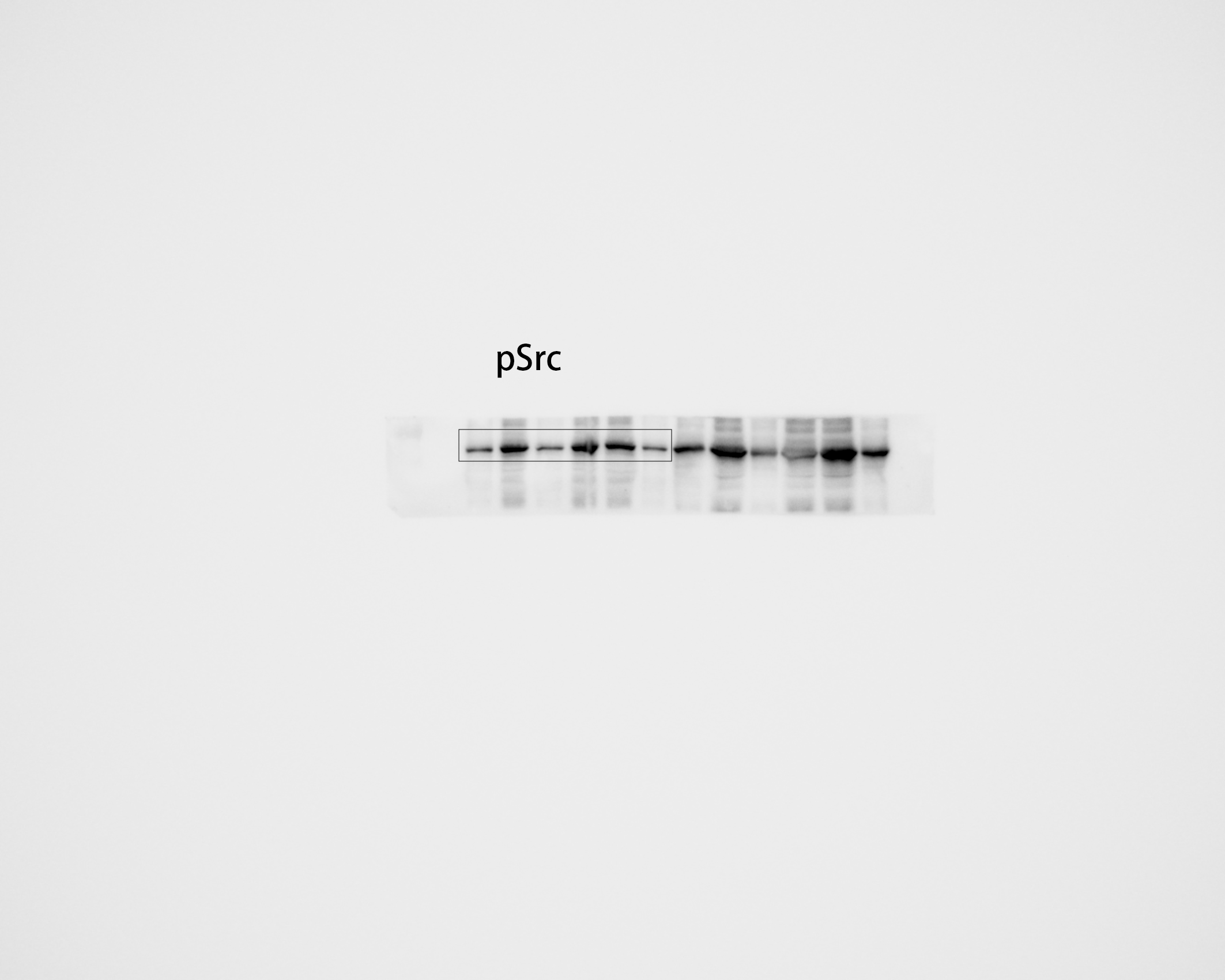

Supplement: Supplementary file 6 — Source data Fig. 4 [file 44318_2024_110_MOESM6_ESM.zip › Figure 4/4C/11-pSrc.tif]

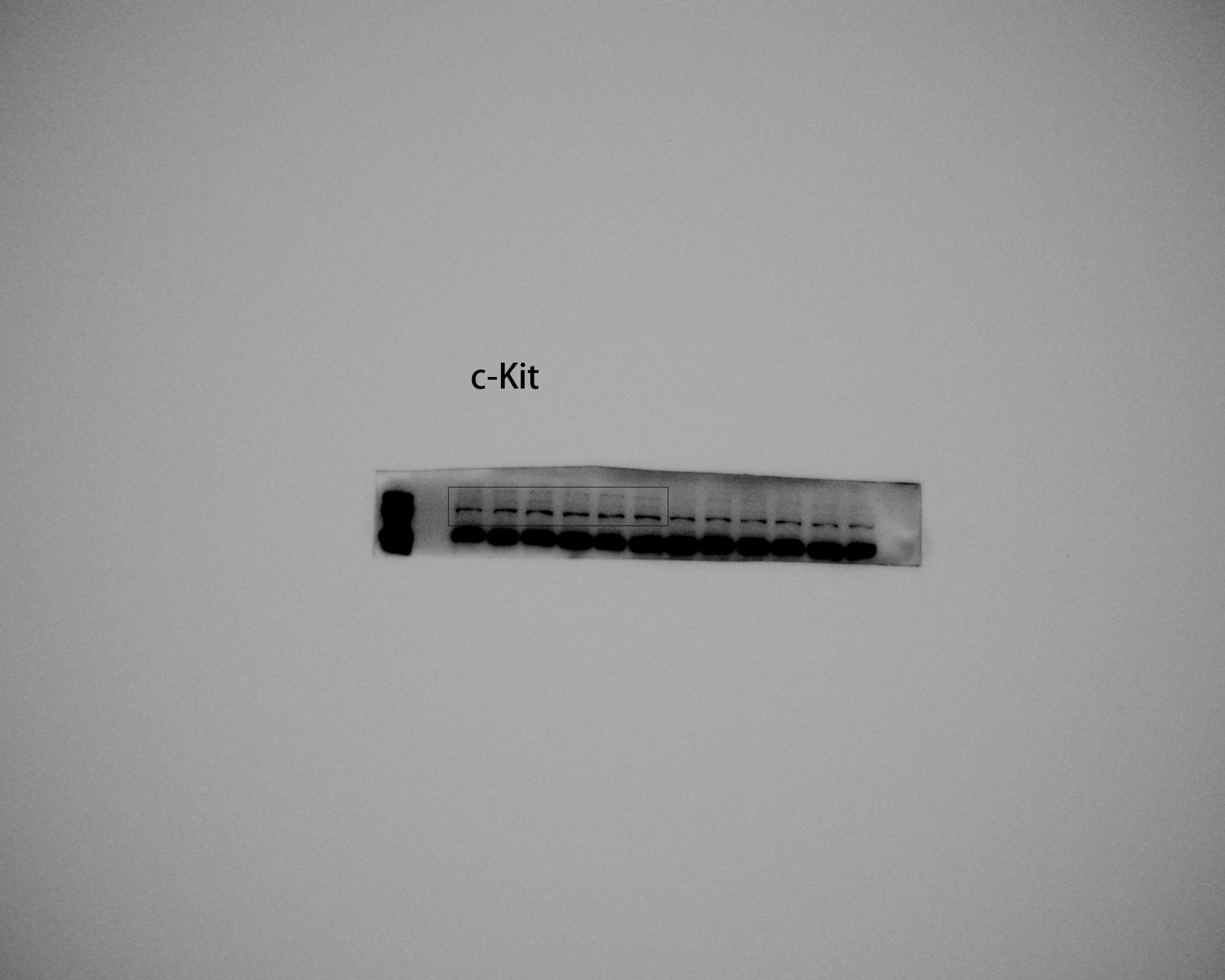

Supplement: Supplementary file 6 — Source data Fig. 4 [file 44318_2024_110_MOESM6_ESM.zip › Figure 4/4C/10-c-Kit.tif]

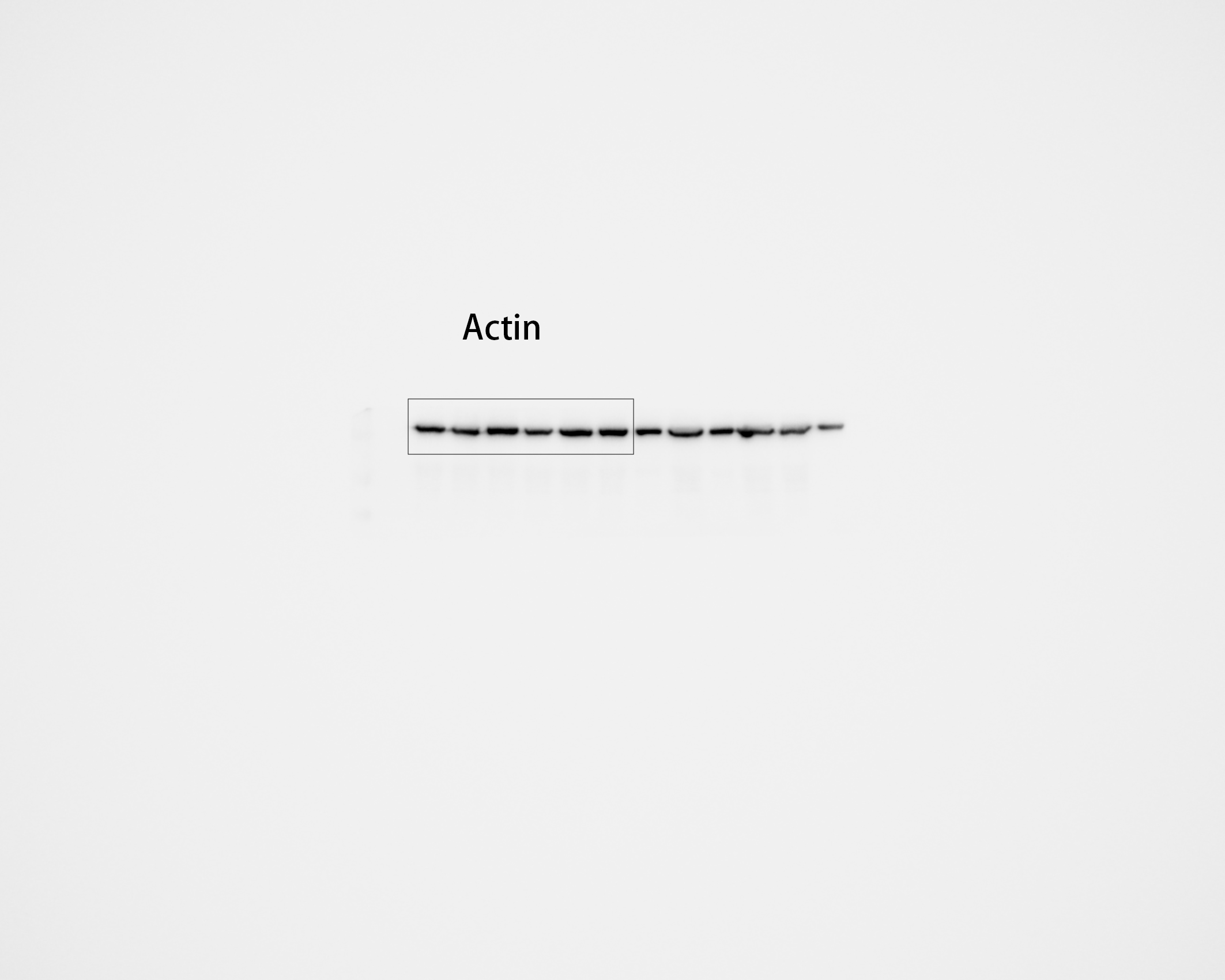

Supplement: Supplementary file 6 — Source data Fig. 4 [file 44318_2024_110_MOESM6_ESM.zip › Figure 4/4C/15-Actin.tif]

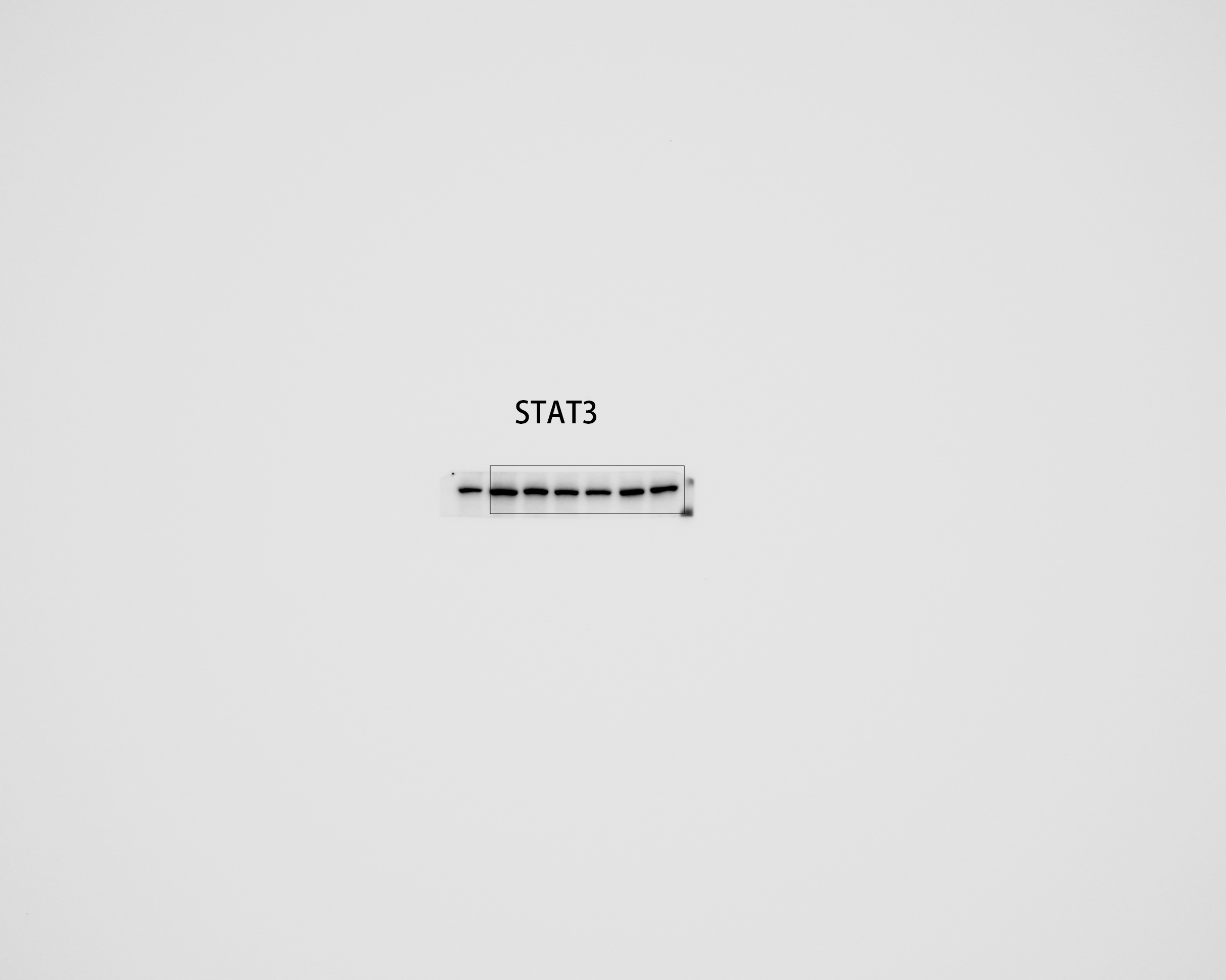

Supplement: Supplementary file 6 — Source data Fig. 4 [file 44318_2024_110_MOESM6_ESM.zip › Figure 4/4C/8-STAT3.tif]

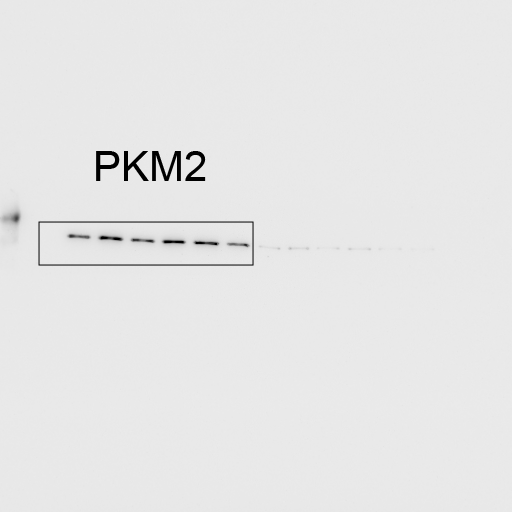

Supplement: Supplementary file 6 — Source data Fig. 4 [file 44318_2024_110_MOESM6_ESM.zip › Figure 4/4C/2-PKM2.tif]

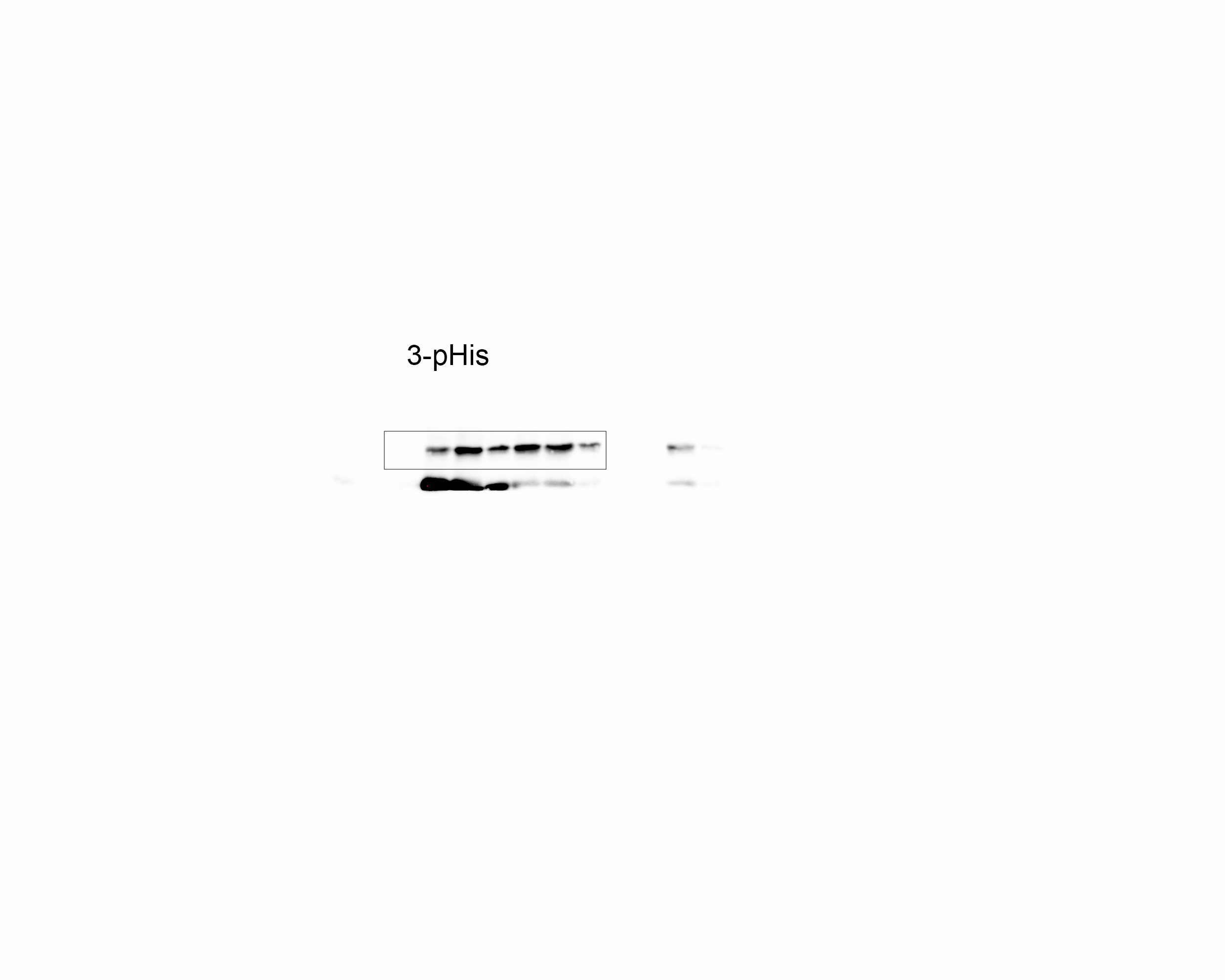

Supplement: Supplementary file 6 — Source data Fig. 4 [file 44318_2024_110_MOESM6_ESM.zip › Figure 4/4C/3-3-pHis.tif]

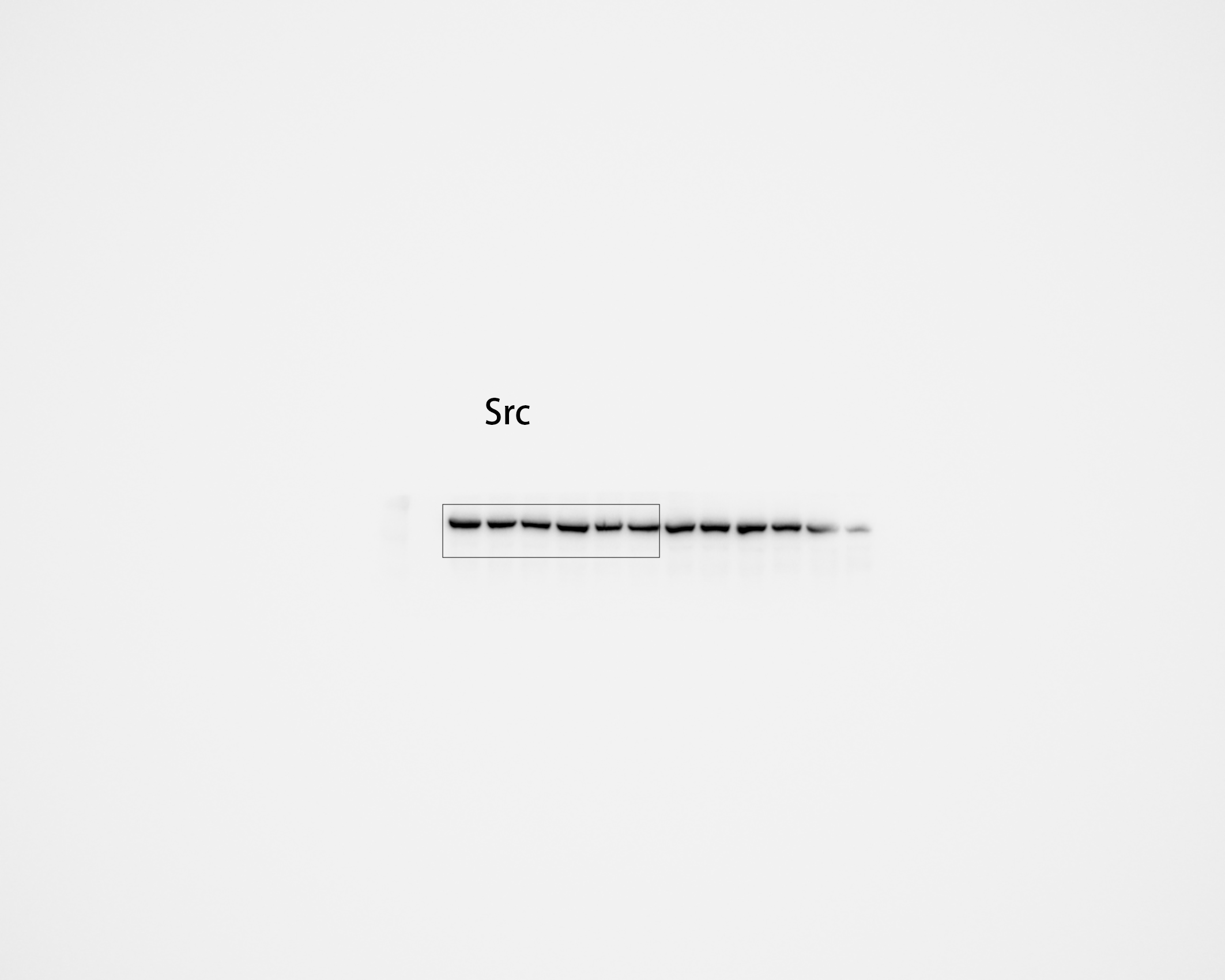

Supplement: Supplementary file 6 — Source data Fig. 4 [file 44318_2024_110_MOESM6_ESM.zip › Figure 4/4C/12-Src.tif]

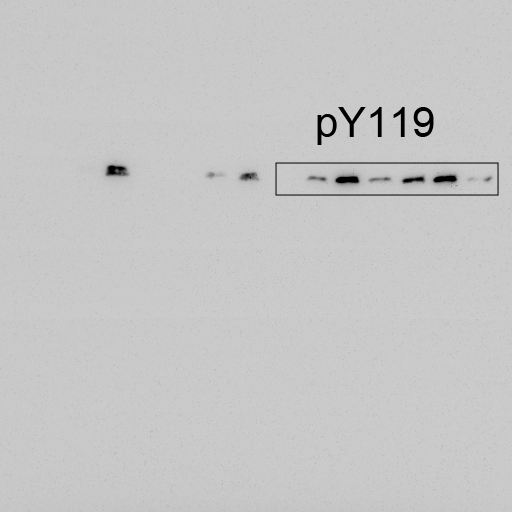

Supplement: Supplementary file 6 — Source data Fig. 4 [file 44318_2024_110_MOESM6_ESM.zip › Figure 4/4C/1-pY119.tif]

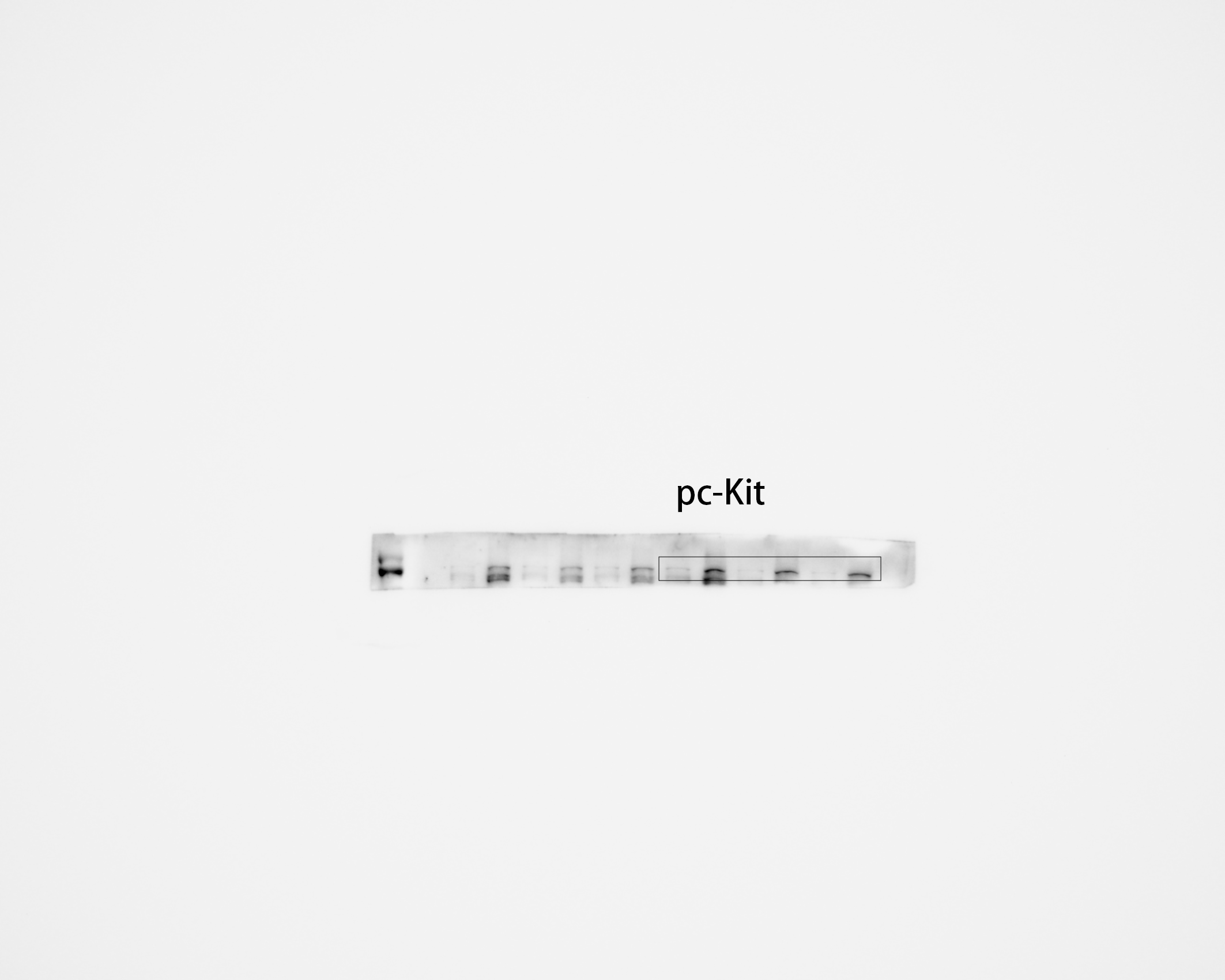

Supplement: Supplementary file 6 — Source data Fig. 4 [file 44318_2024_110_MOESM6_ESM.zip › Figure 4/4C/9-pc-Kit.tif]

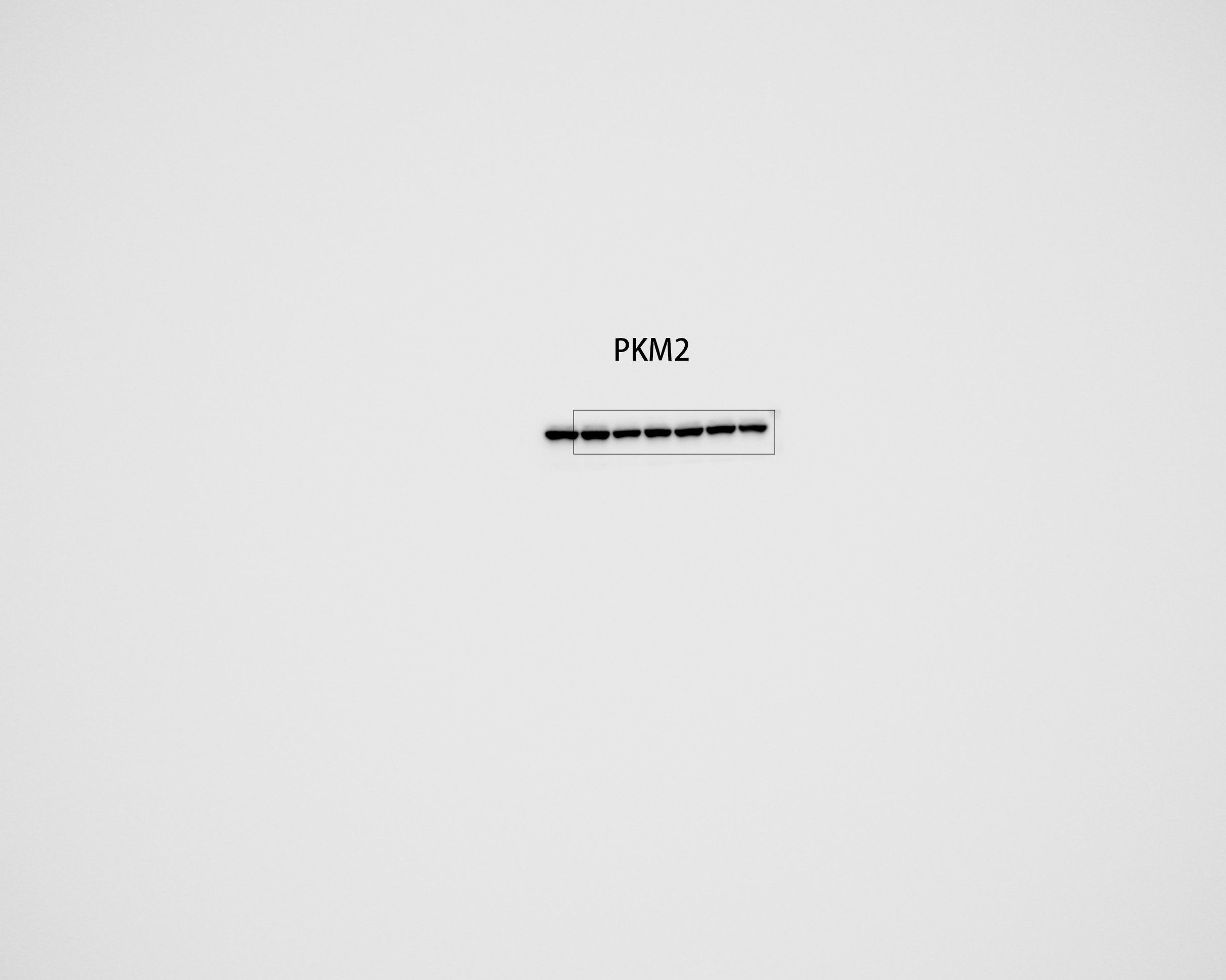

Supplement: Supplementary file 6 — Source data Fig. 4 [file 44318_2024_110_MOESM6_ESM.zip › Figure 4/4C/13-PKM2.tif]

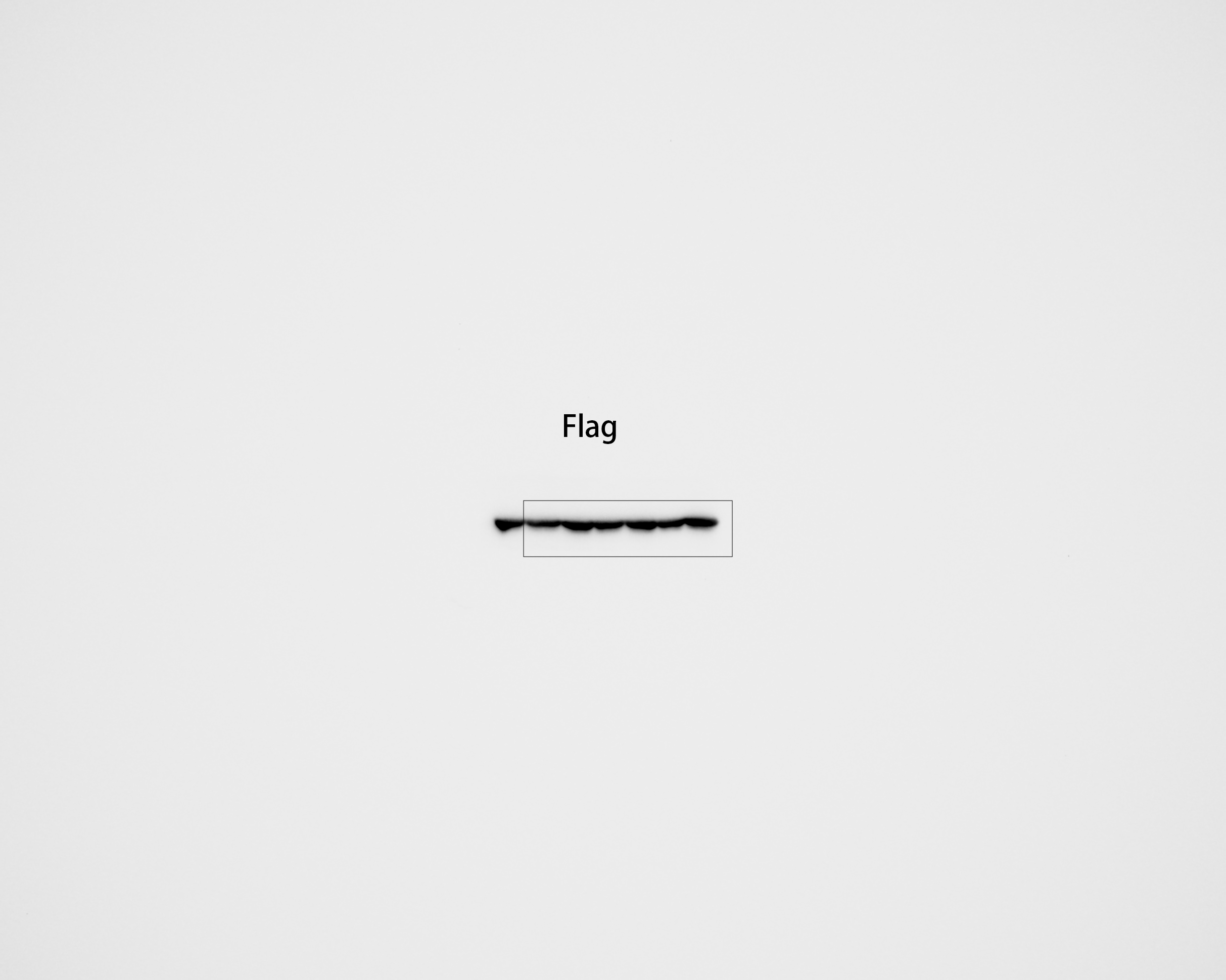

Supplement: Supplementary file 6 — Source data Fig. 4 [file 44318_2024_110_MOESM6_ESM.zip › Figure 4/4C/14-Flag.tif]

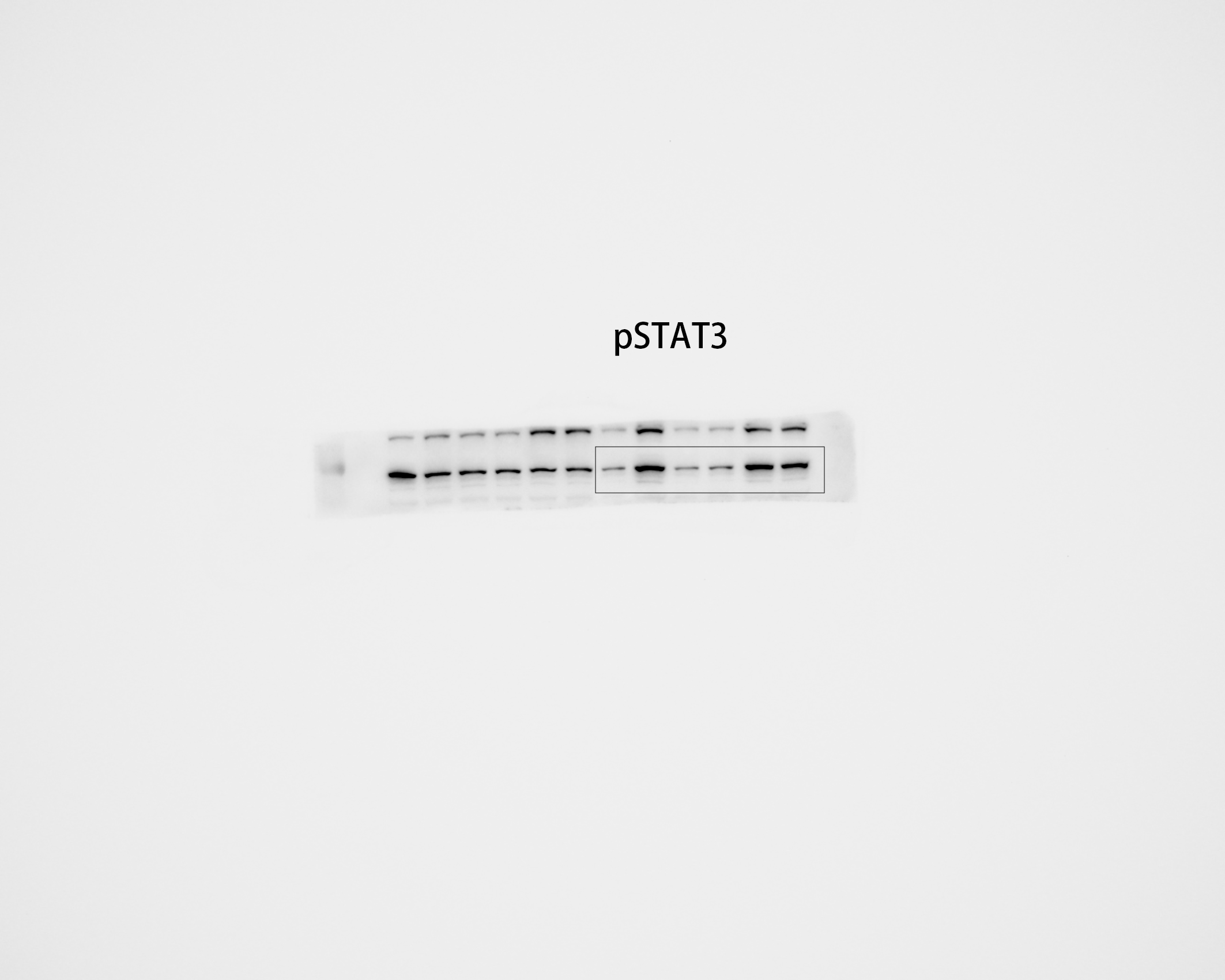

Supplement: Supplementary file 6 — Source data Fig. 4 [file 44318_2024_110_MOESM6_ESM.zip › Figure 4/4C/7-pSTAT3.tif]
