# Supplementary figures and images for: PKM2 functions as a histidine kinase to phosphorylate PGAM1 and increase glycolysis shunts in cancer (part 2 of 3)
Source: EMBO J. 2024 May 15;43(12):5. doi: 10.1038/s44318-024-00110-8 (PMC11183095; doi:10.1038/s44318-024-00110-8)

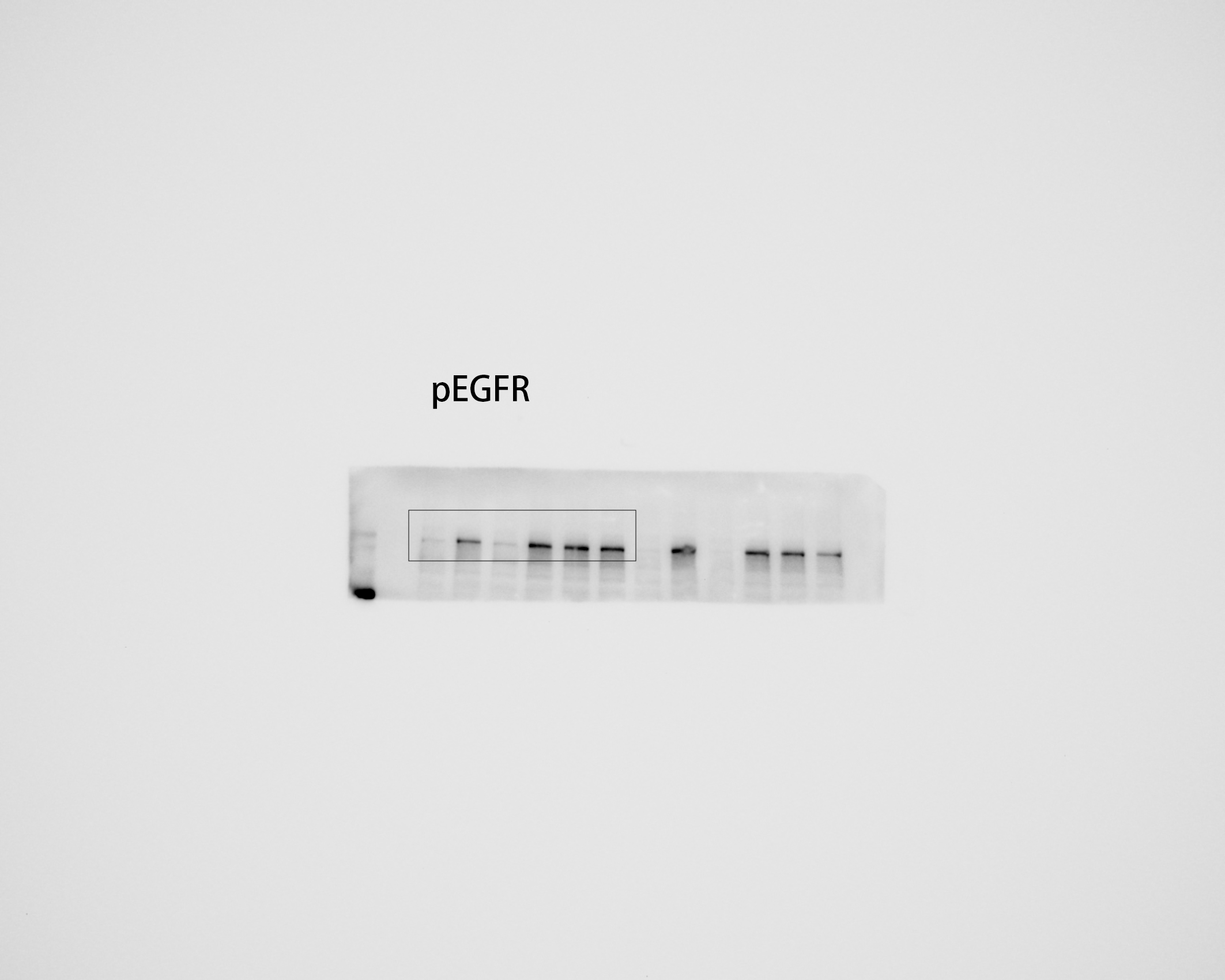

Supplement: Supplementary file 6 — Source data Fig. 4 [file 44318_2024_110_MOESM6_ESM.zip › Figure 4/4C/5-pEGFR.tif]

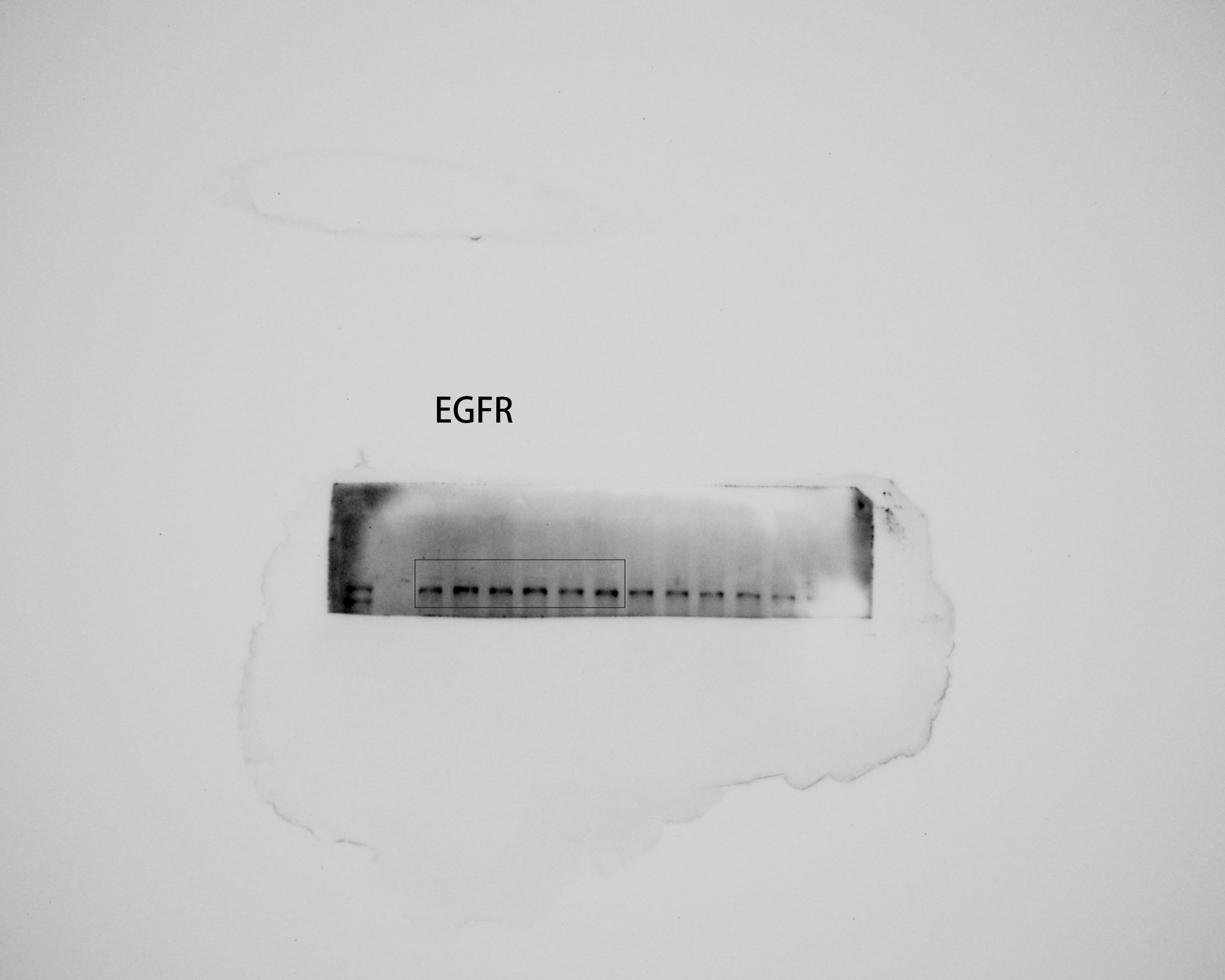

Supplement: Supplementary file 6 — Source data Fig. 4 [file 44318_2024_110_MOESM6_ESM.zip › Figure 4/4C/6-EGFR.tif]

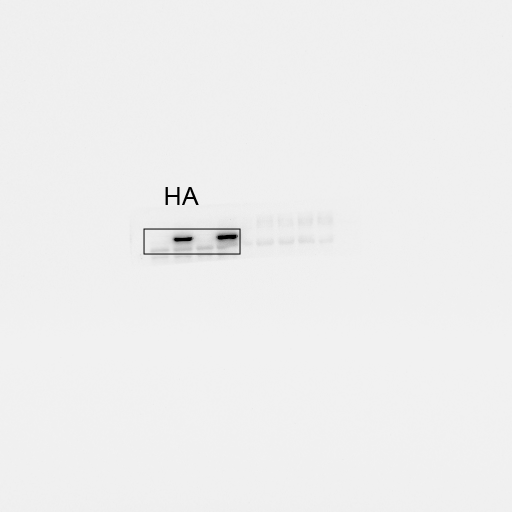

Supplement: Supplementary file 6 — Source data Fig. 4 [file 44318_2024_110_MOESM6_ESM.zip › Figure 4/4D/5-HA.tif]

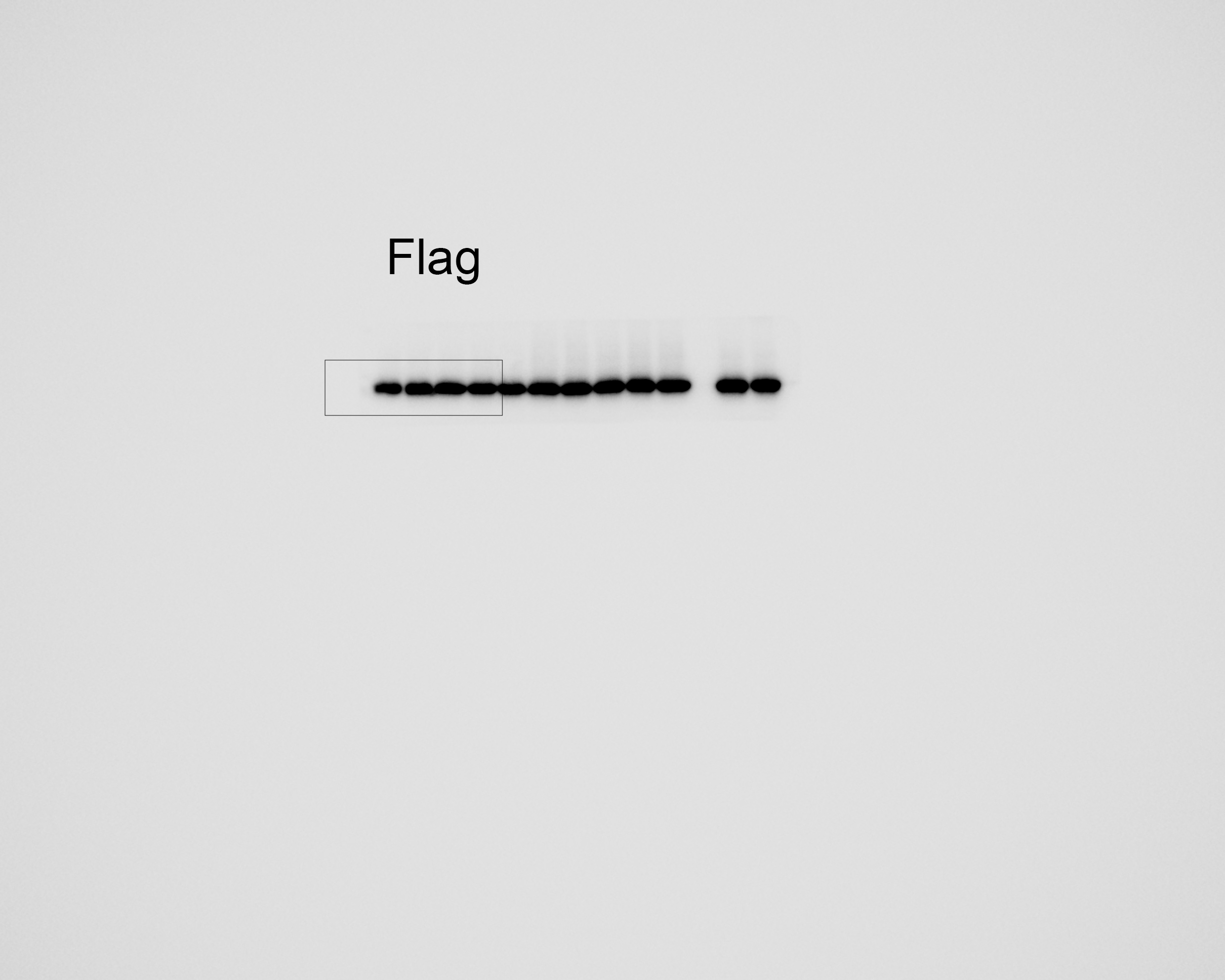

Supplement: Supplementary file 6 — Source data Fig. 4 [file 44318_2024_110_MOESM6_ESM.zip › Figure 4/4D/4-Flag.tif]

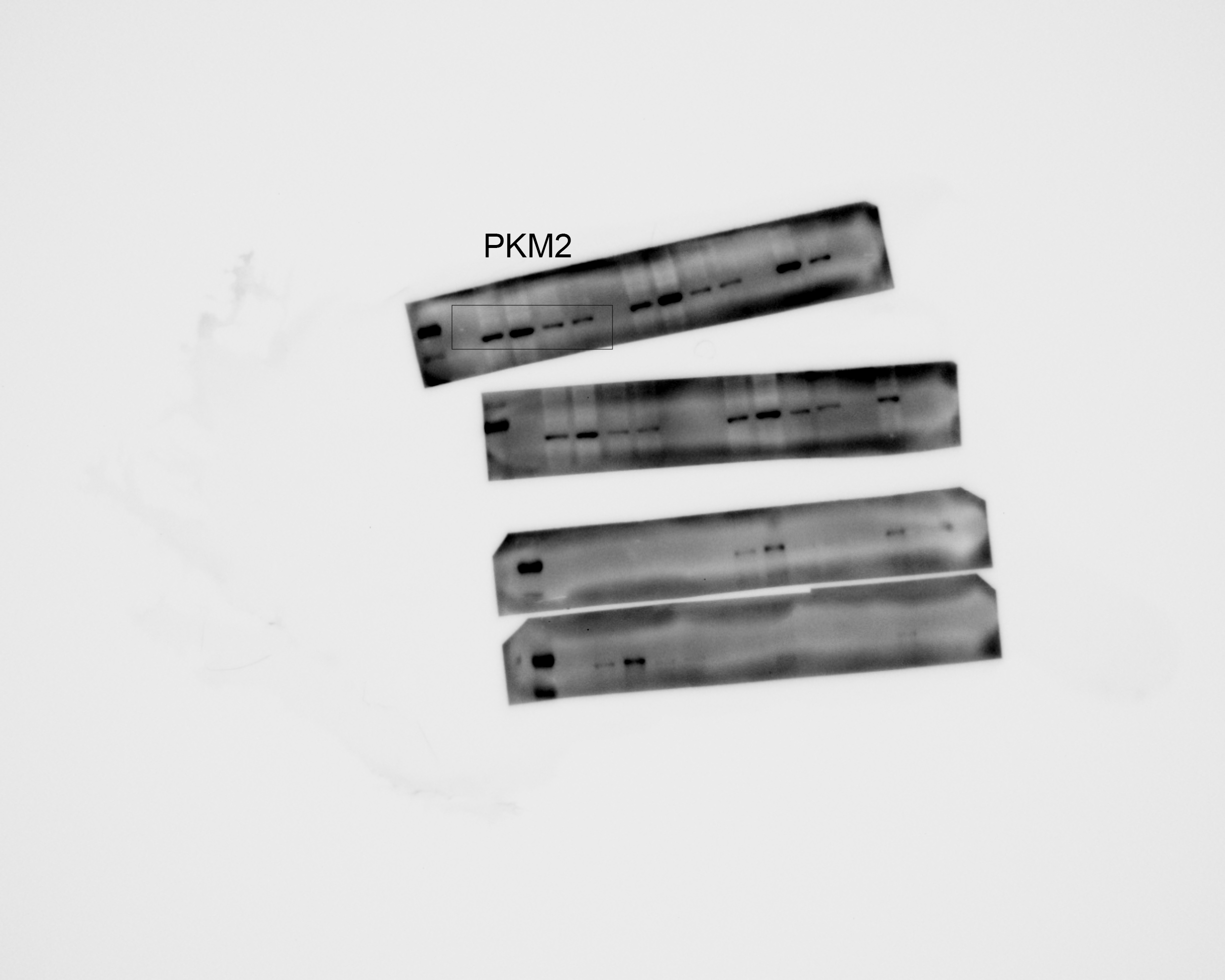

Supplement: Supplementary file 6 — Source data Fig. 4 [file 44318_2024_110_MOESM6_ESM.zip › Figure 4/4D/2-PKM2.tif]

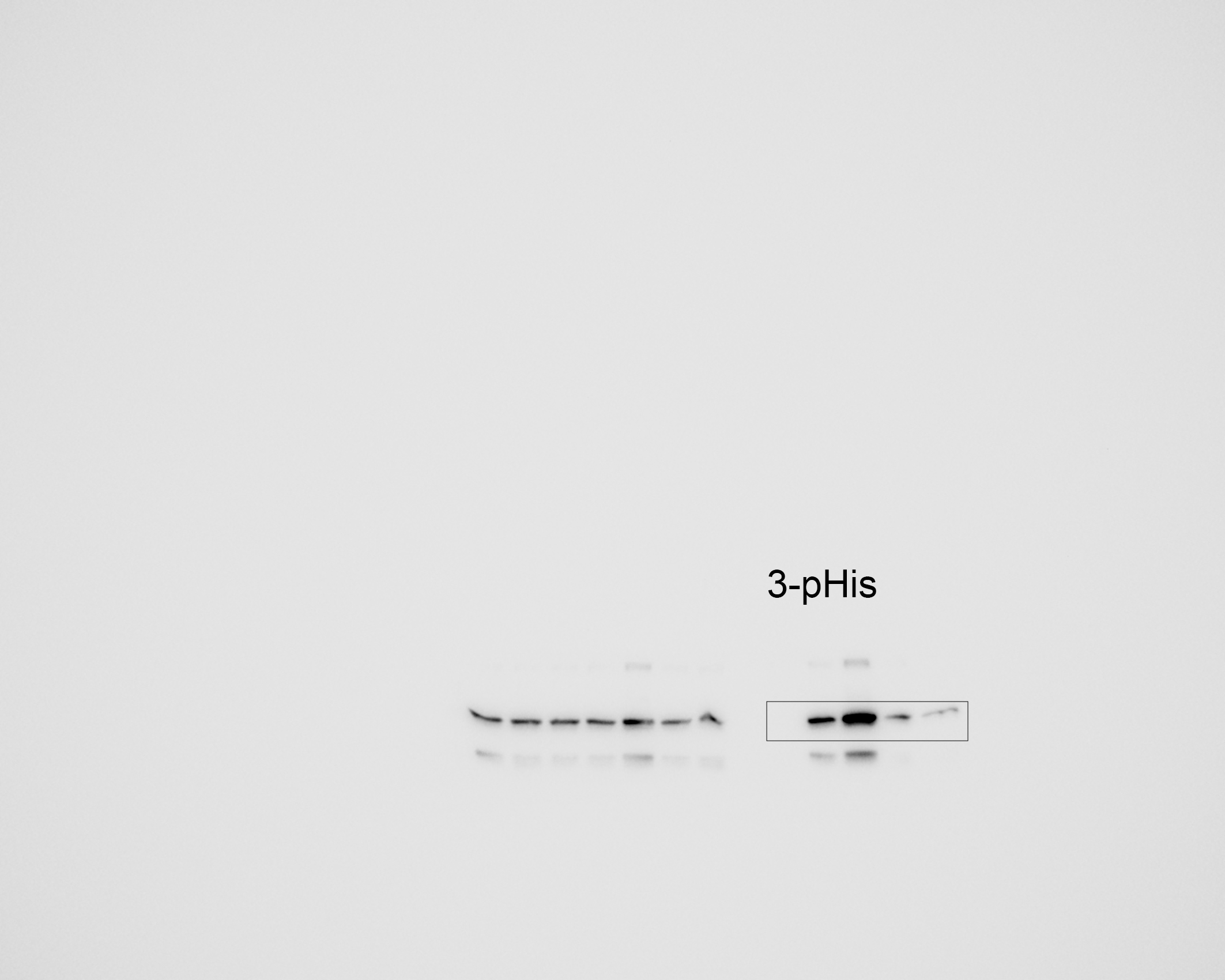

Supplement: Supplementary file 6 — Source data Fig. 4 [file 44318_2024_110_MOESM6_ESM.zip › Figure 4/4D/3-3-pHis.tif]

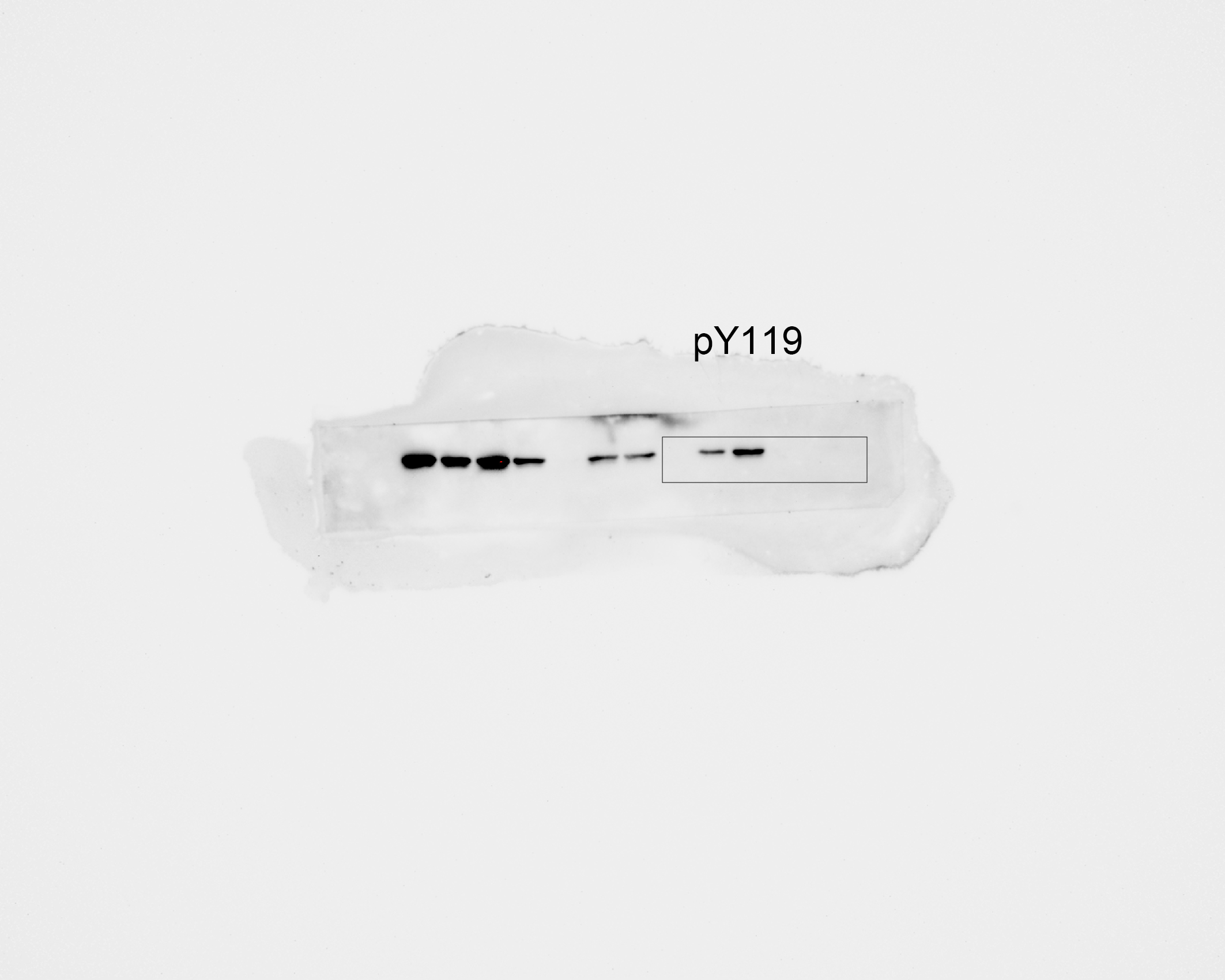

Supplement: Supplementary file 6 — Source data Fig. 4 [file 44318_2024_110_MOESM6_ESM.zip › Figure 4/4D/1-pY119.tif]

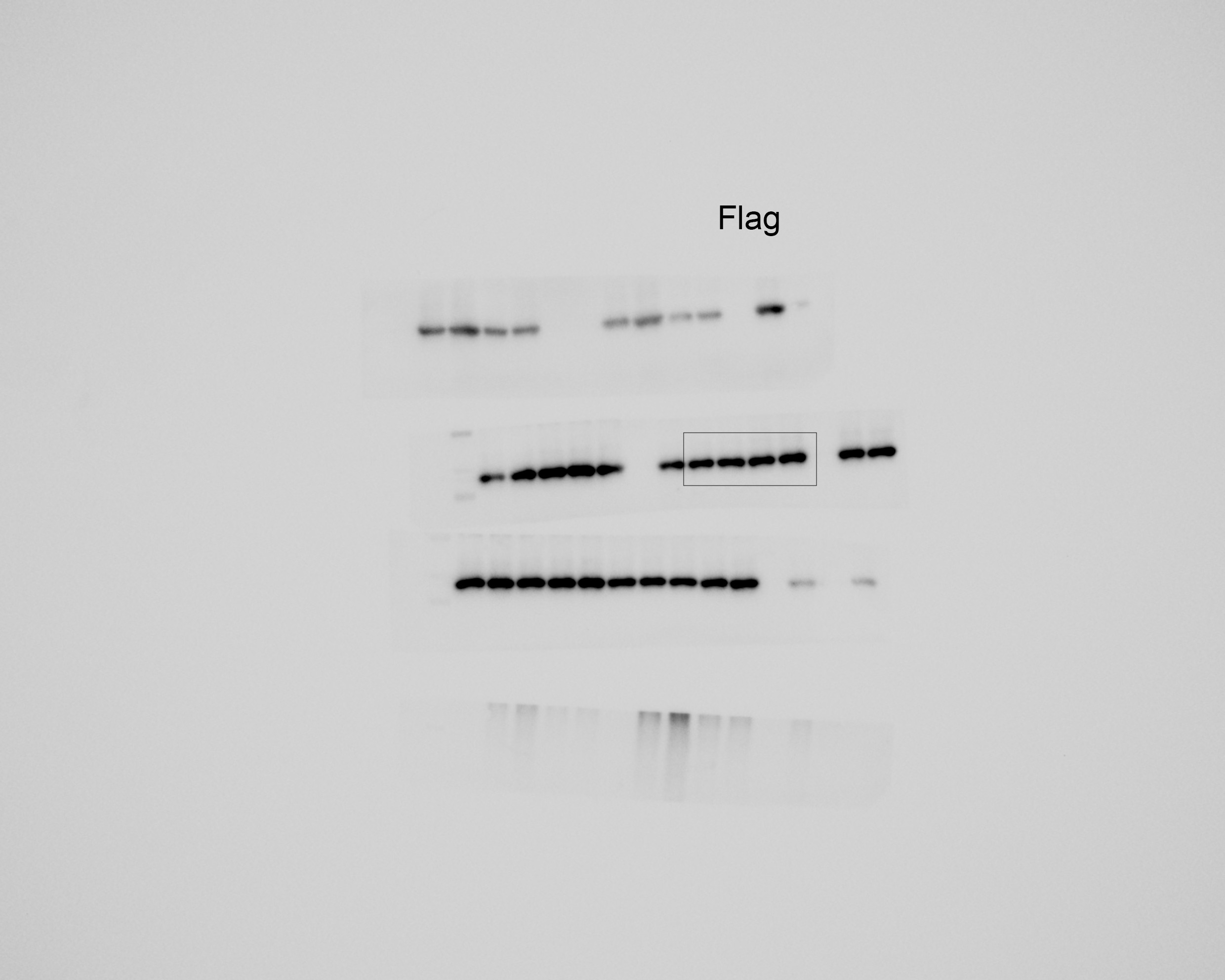

Supplement: Supplementary file 6 — Source data Fig. 4 [file 44318_2024_110_MOESM6_ESM.zip › Figure 4/4D/8-Flag.tif]

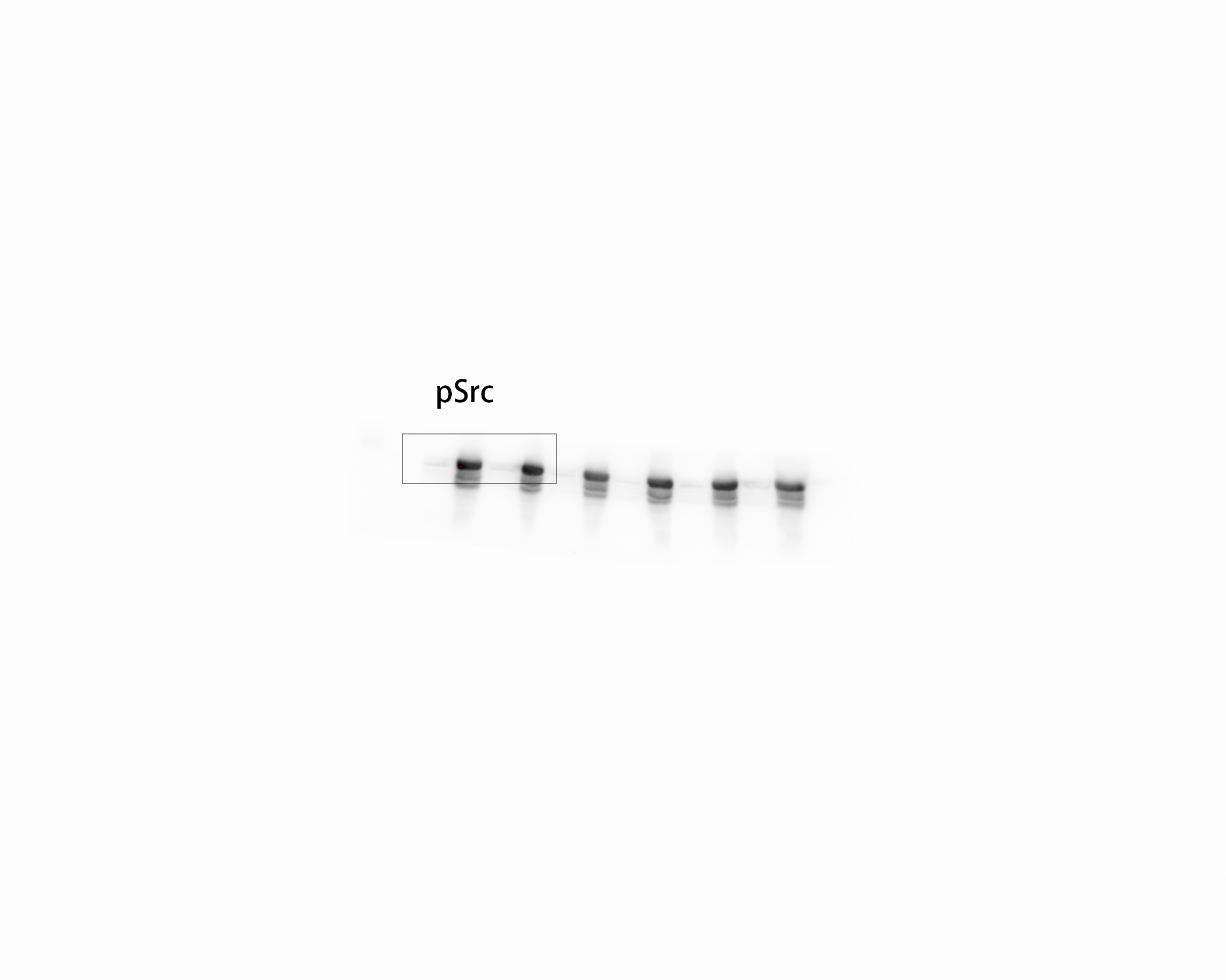

Supplement: Supplementary file 6 — Source data Fig. 4 [file 44318_2024_110_MOESM6_ESM.zip › Figure 4/4D/6-pSrc.tif]

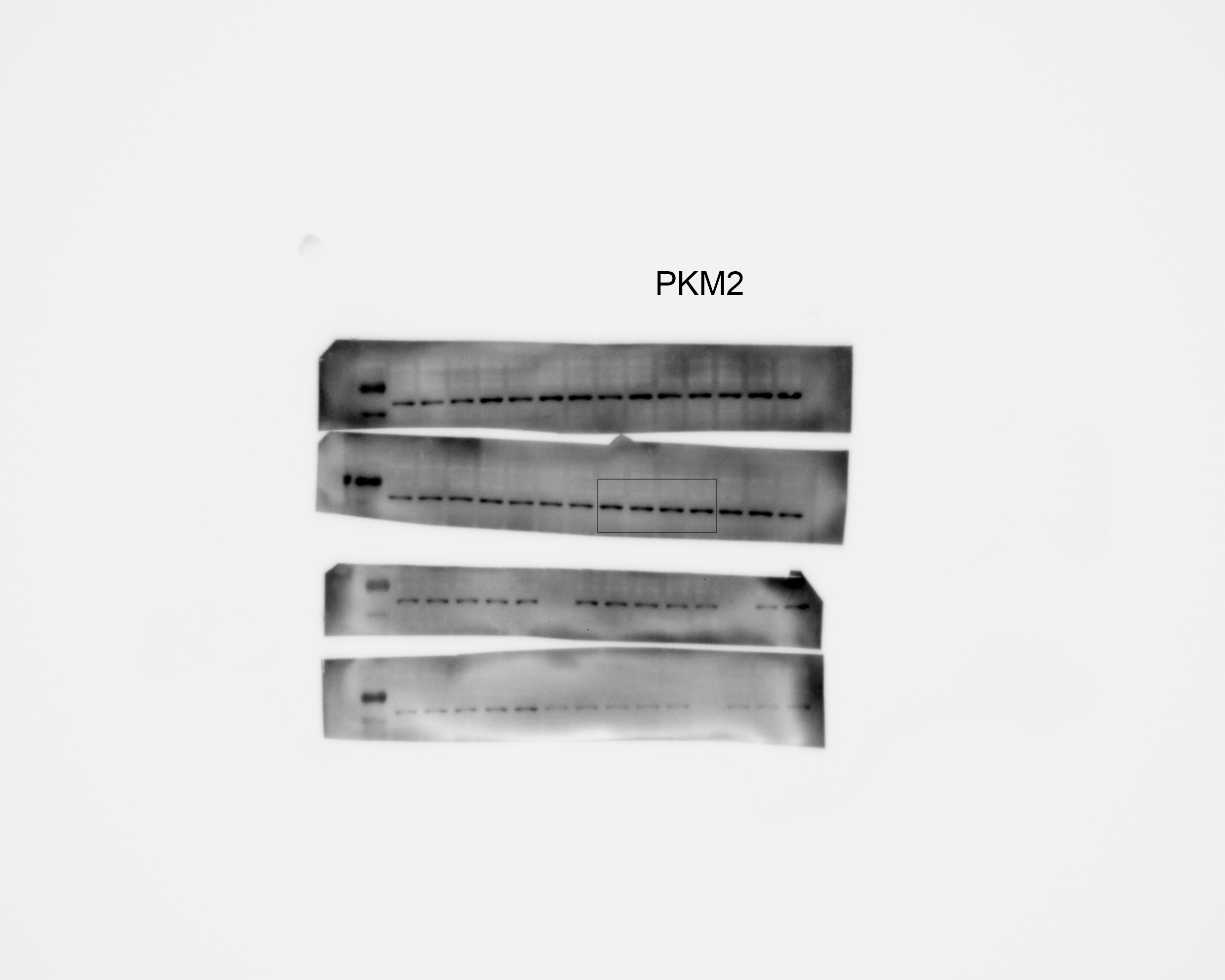

Supplement: Supplementary file 6 — Source data Fig. 4 [file 44318_2024_110_MOESM6_ESM.zip › Figure 4/4D/7-PKM2.tif]

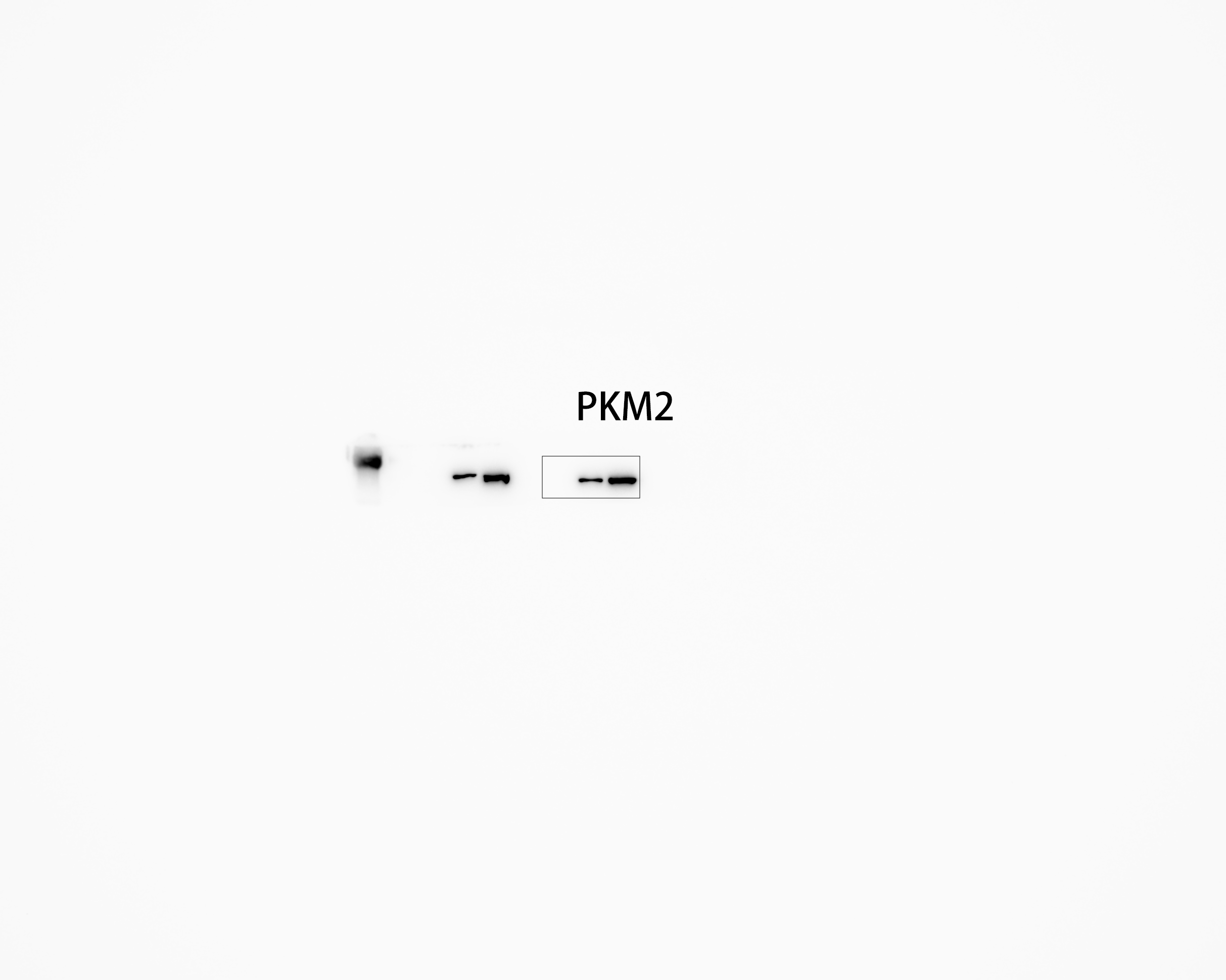

Supplement: Supplementary file 6 — Source data Fig. 4 [file 44318_2024_110_MOESM6_ESM.zip › Figure 4/4A/2-PKM2.tif]

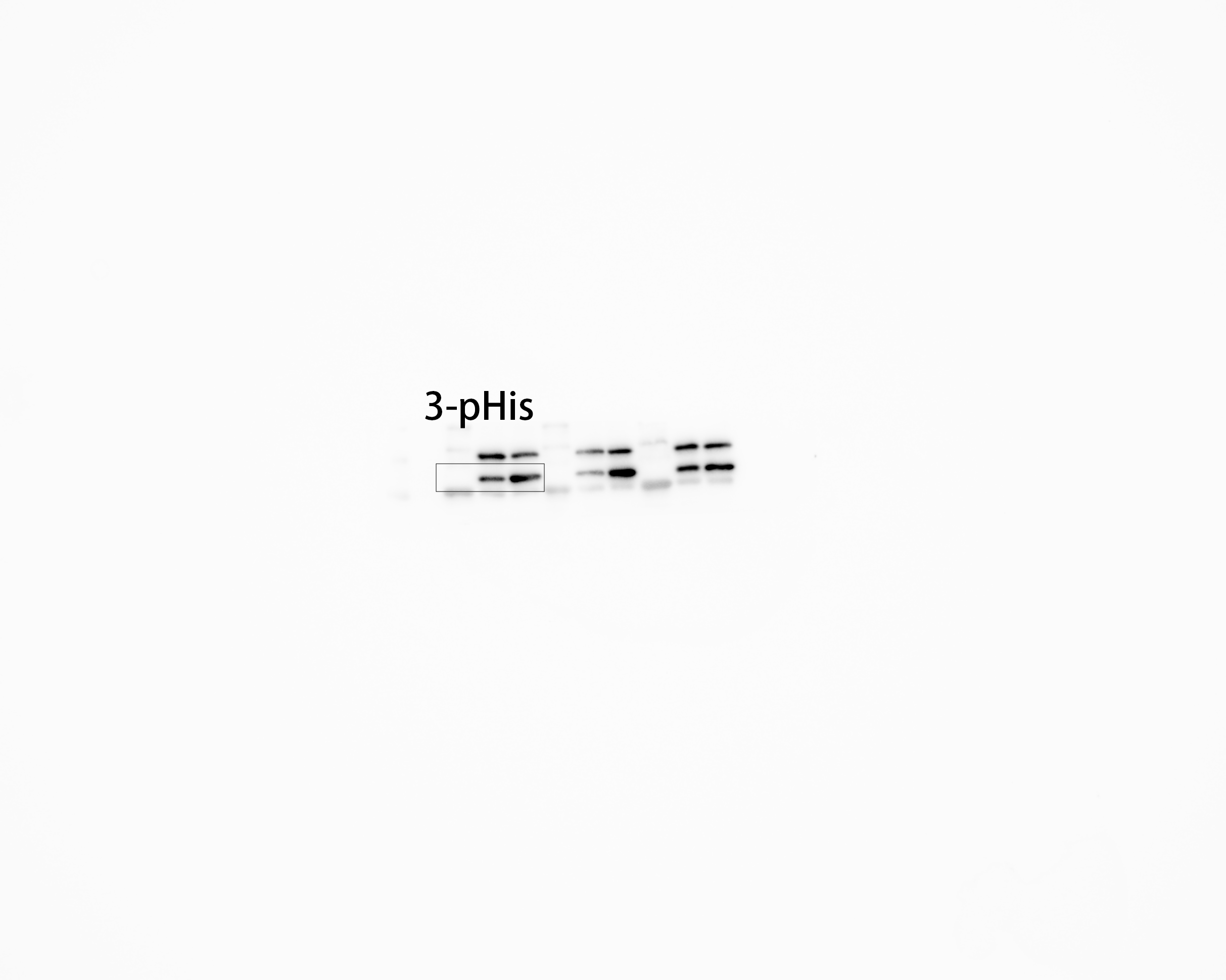

Supplement: Supplementary file 6 — Source data Fig. 4 [file 44318_2024_110_MOESM6_ESM.zip › Figure 4/4A/3-3-pHis.tif]

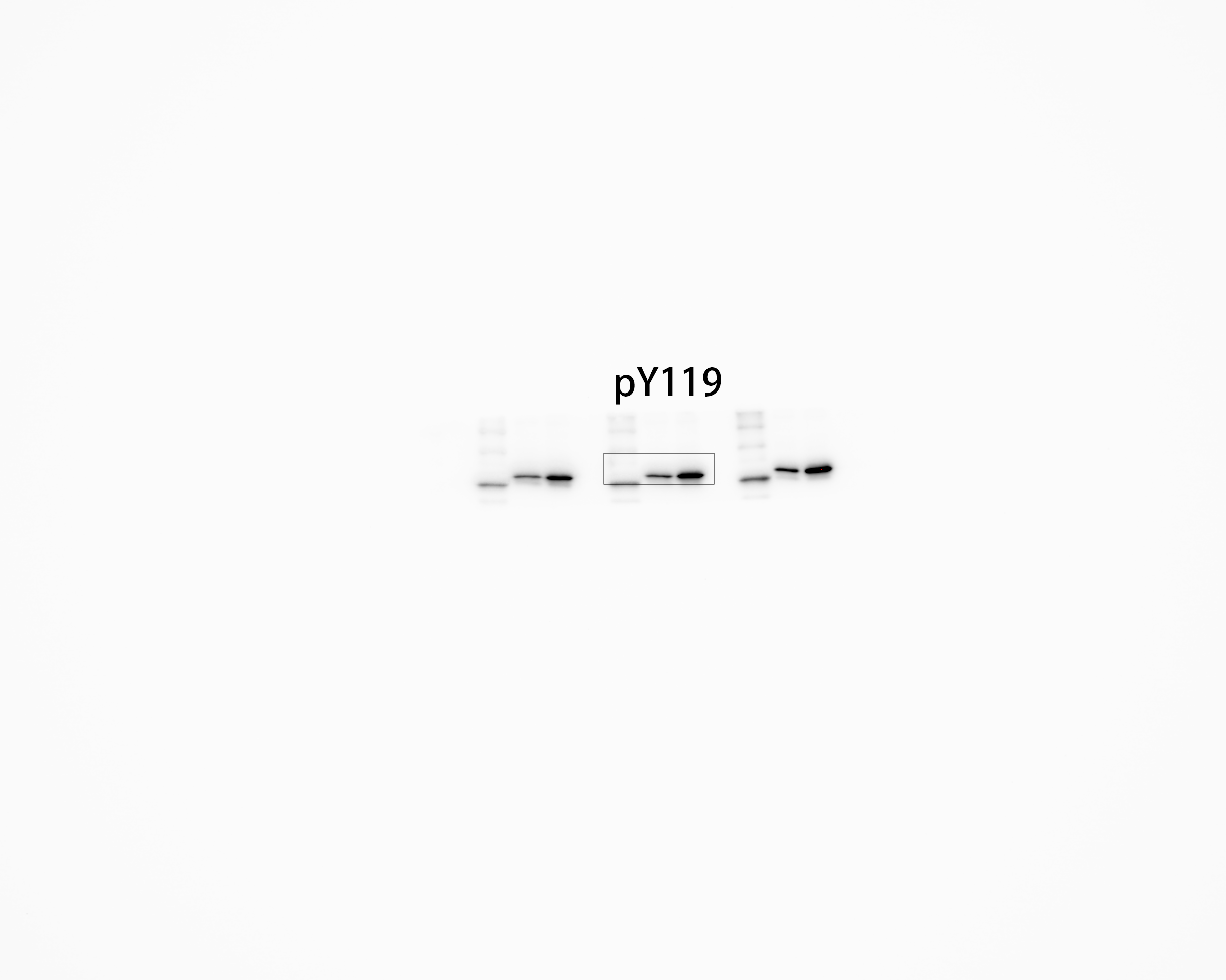

Supplement: Supplementary file 6 — Source data Fig. 4 [file 44318_2024_110_MOESM6_ESM.zip › Figure 4/4A/1-pY119.tif]

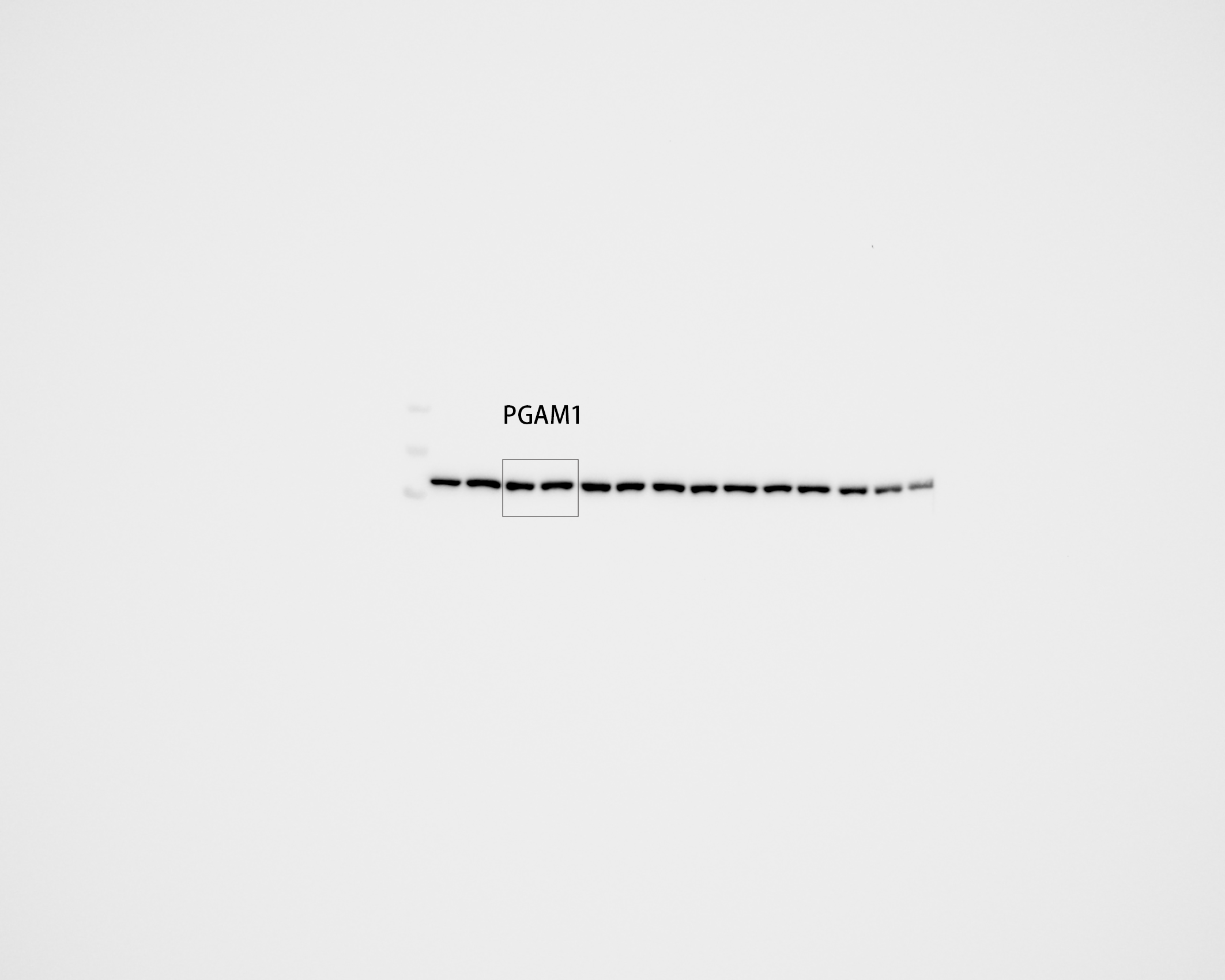

Supplement: Supplementary file 6 — Source data Fig. 4 [file 44318_2024_110_MOESM6_ESM.zip › Figure 4/4A/8-PGAM1.tif]

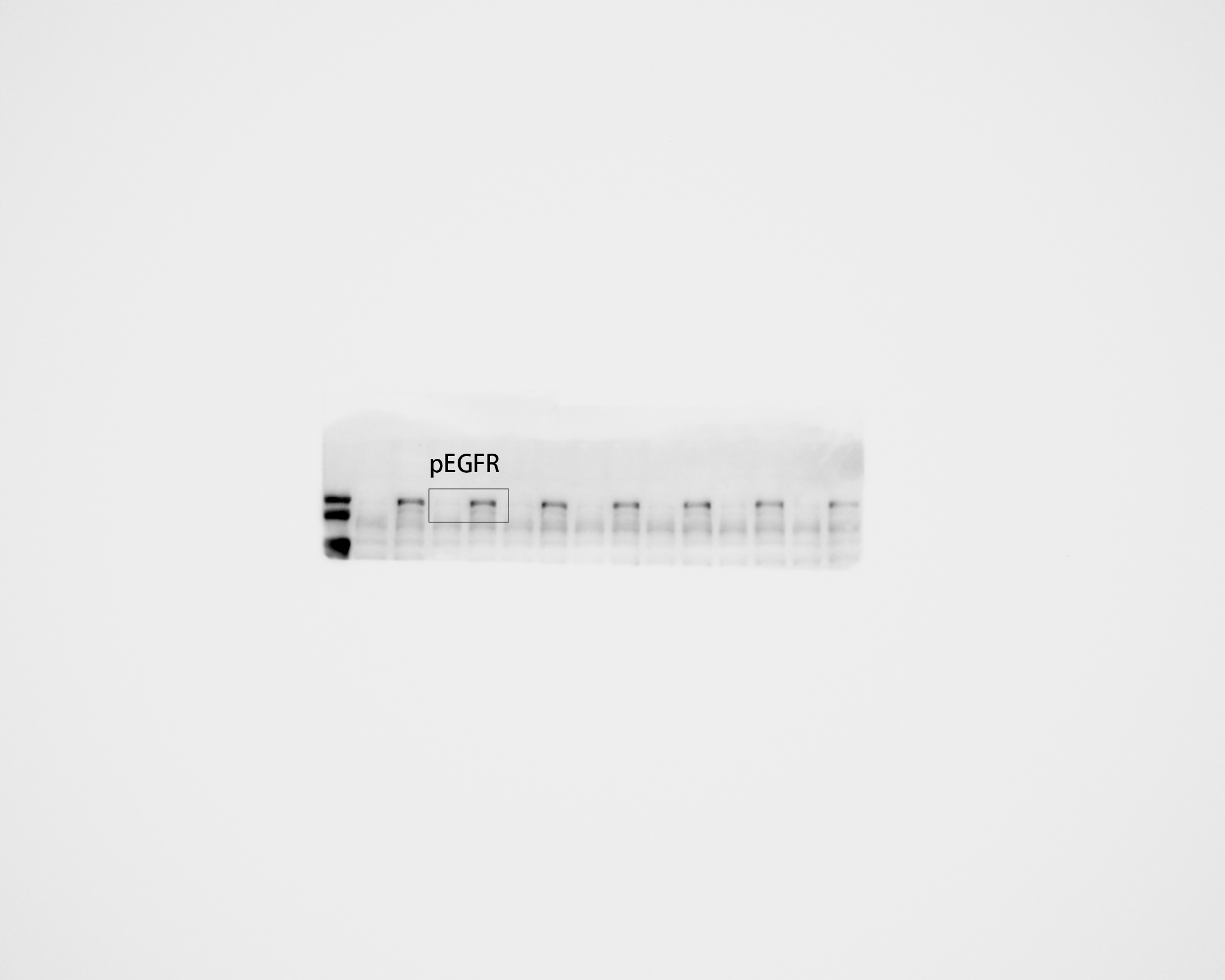

Supplement: Supplementary file 6 — Source data Fig. 4 [file 44318_2024_110_MOESM6_ESM.zip › Figure 4/4A/5-pEGFR.tif]

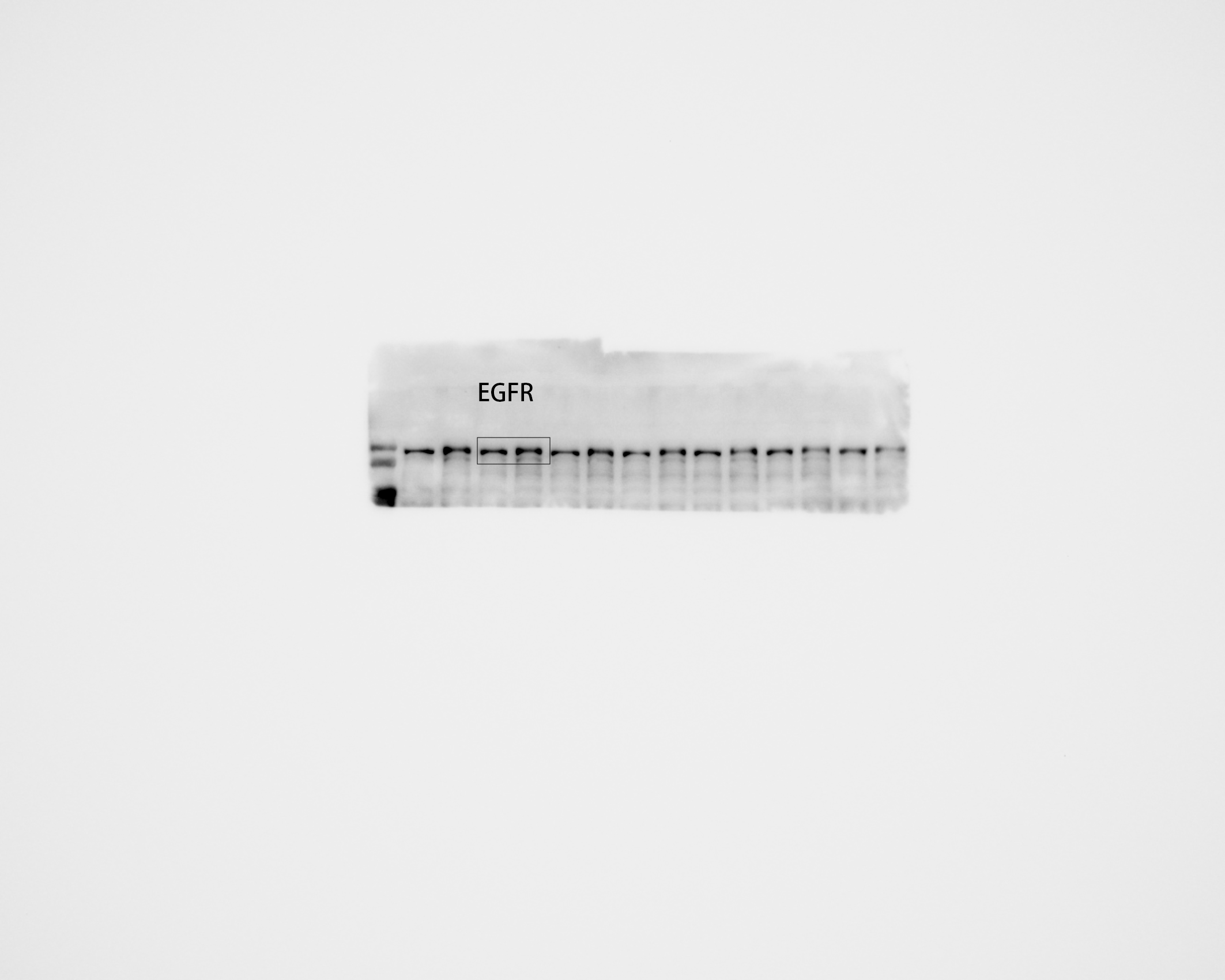

Supplement: Supplementary file 6 — Source data Fig. 4 [file 44318_2024_110_MOESM6_ESM.zip › Figure 4/4A/6-EGFR.tif]

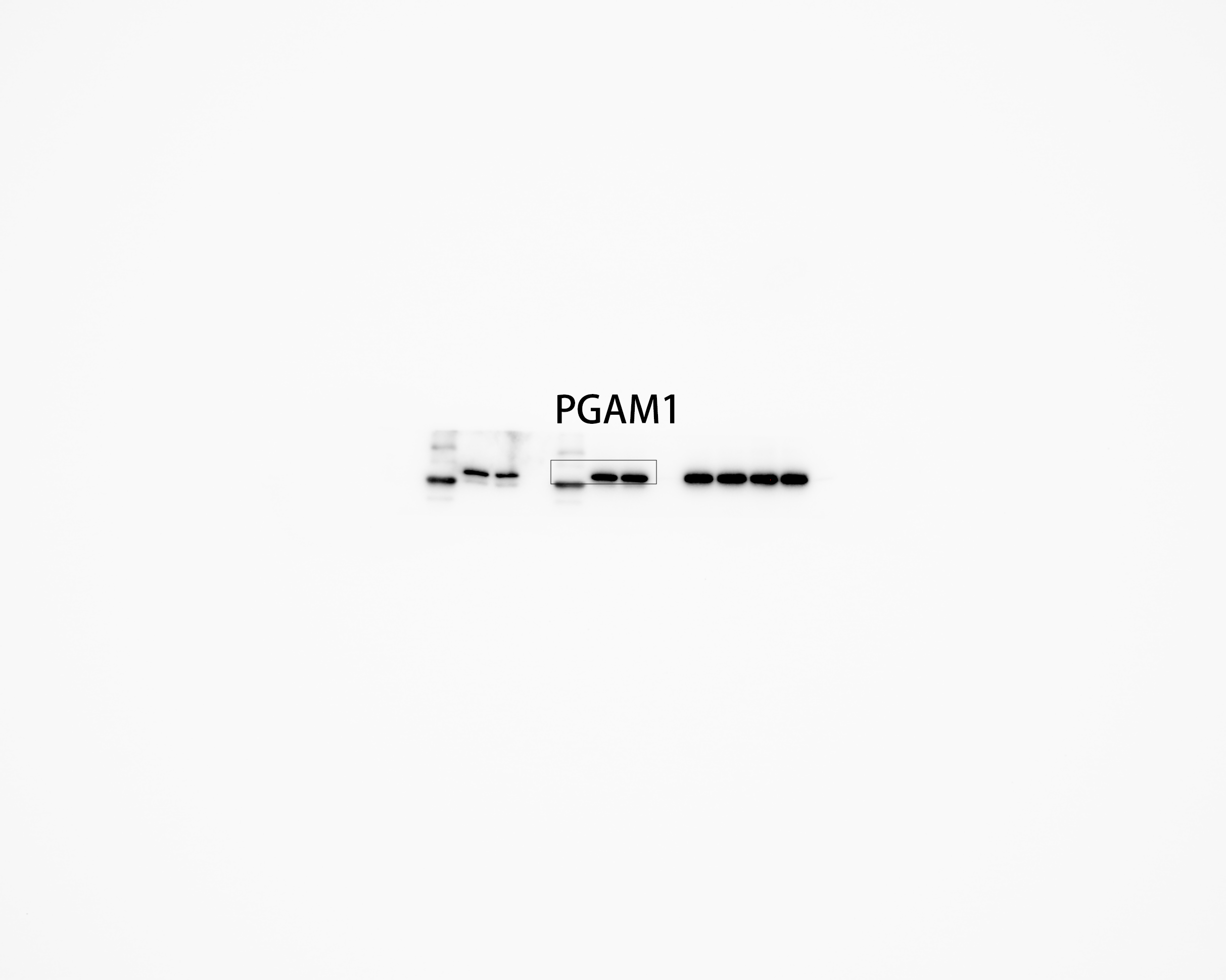

Supplement: Supplementary file 6 — Source data Fig. 4 [file 44318_2024_110_MOESM6_ESM.zip › Figure 4/4A/4-PGAM1.tif]

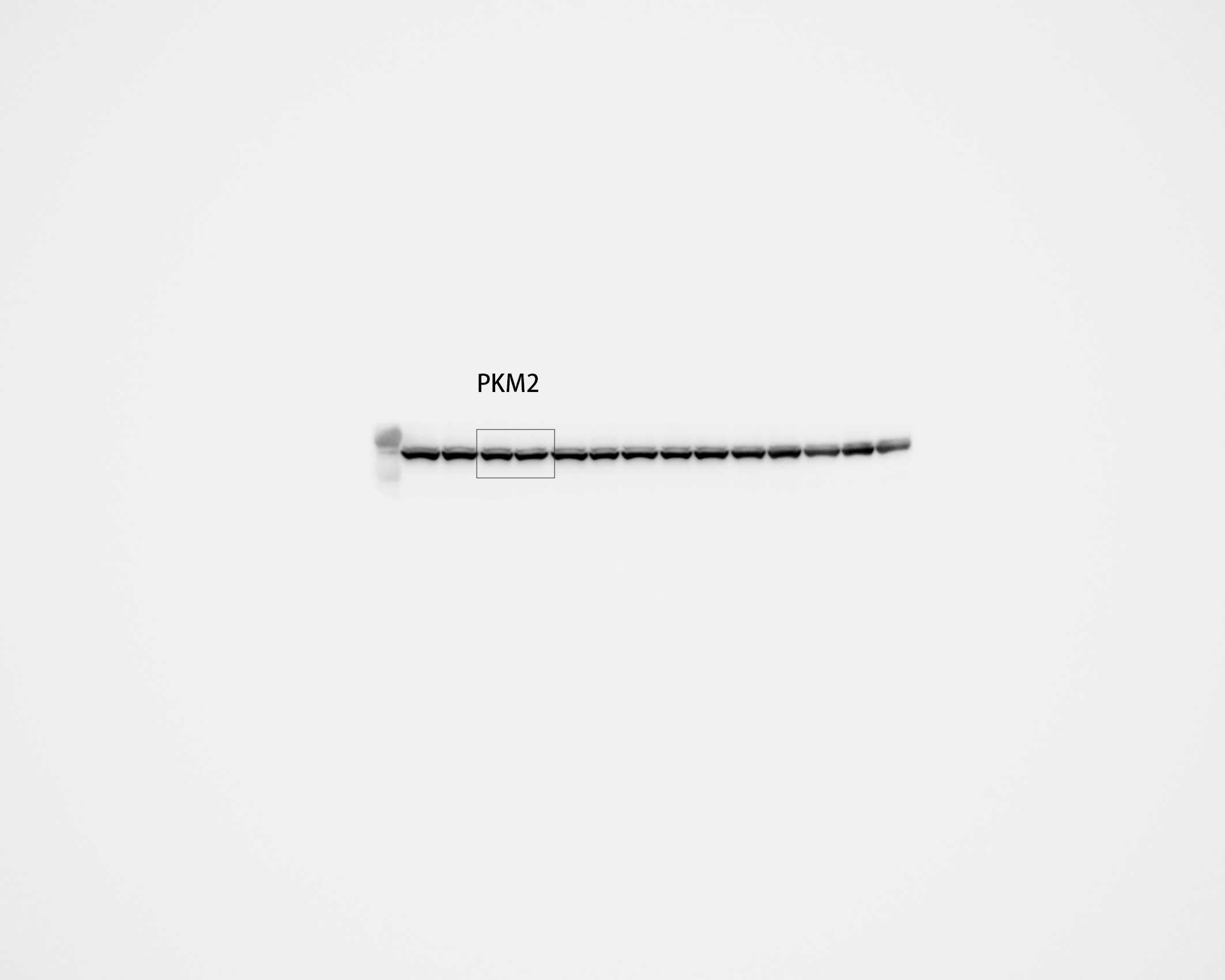

Supplement: Supplementary file 6 — Source data Fig. 4 [file 44318_2024_110_MOESM6_ESM.zip › Figure 4/4A/7-PKM2.tif]

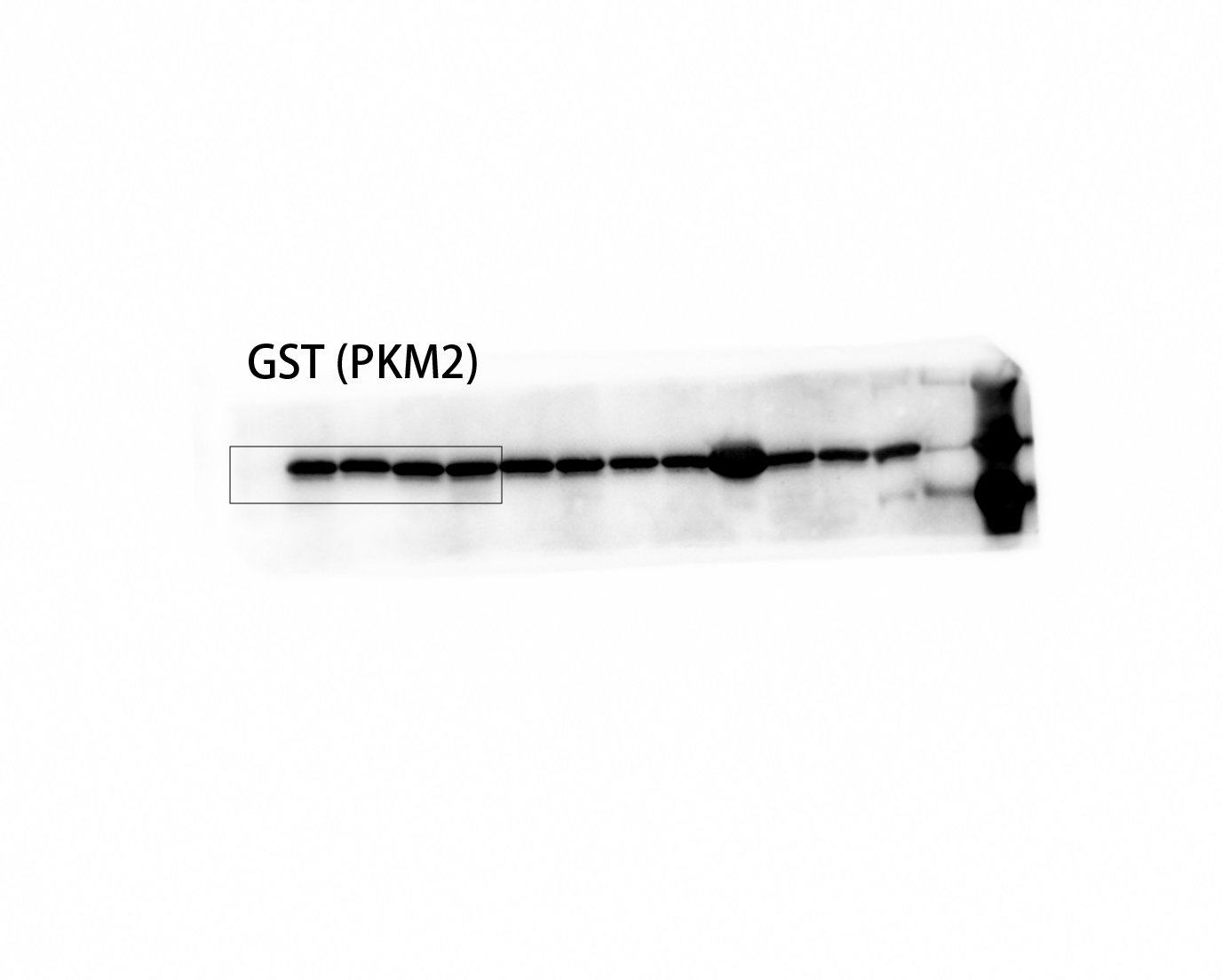

Supplement: Supplementary file 6 — Source data Fig. 4 [file 44318_2024_110_MOESM6_ESM.zip › Figure 4/4F/5-GST (PKM2).Tif]

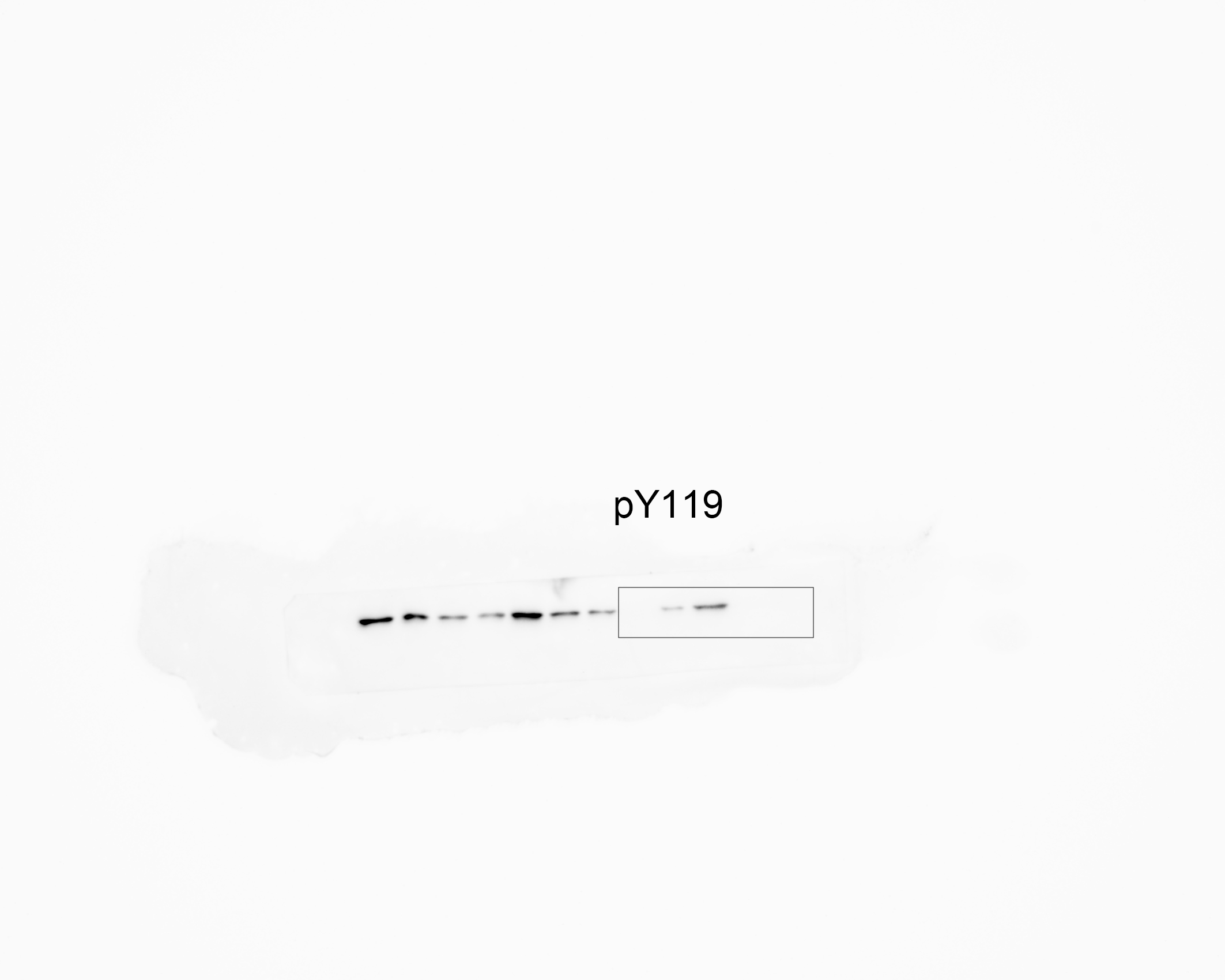

Supplement: Supplementary file 6 — Source data Fig. 4 [file 44318_2024_110_MOESM6_ESM.zip › Figure 4/4F/1-pY119.tif]

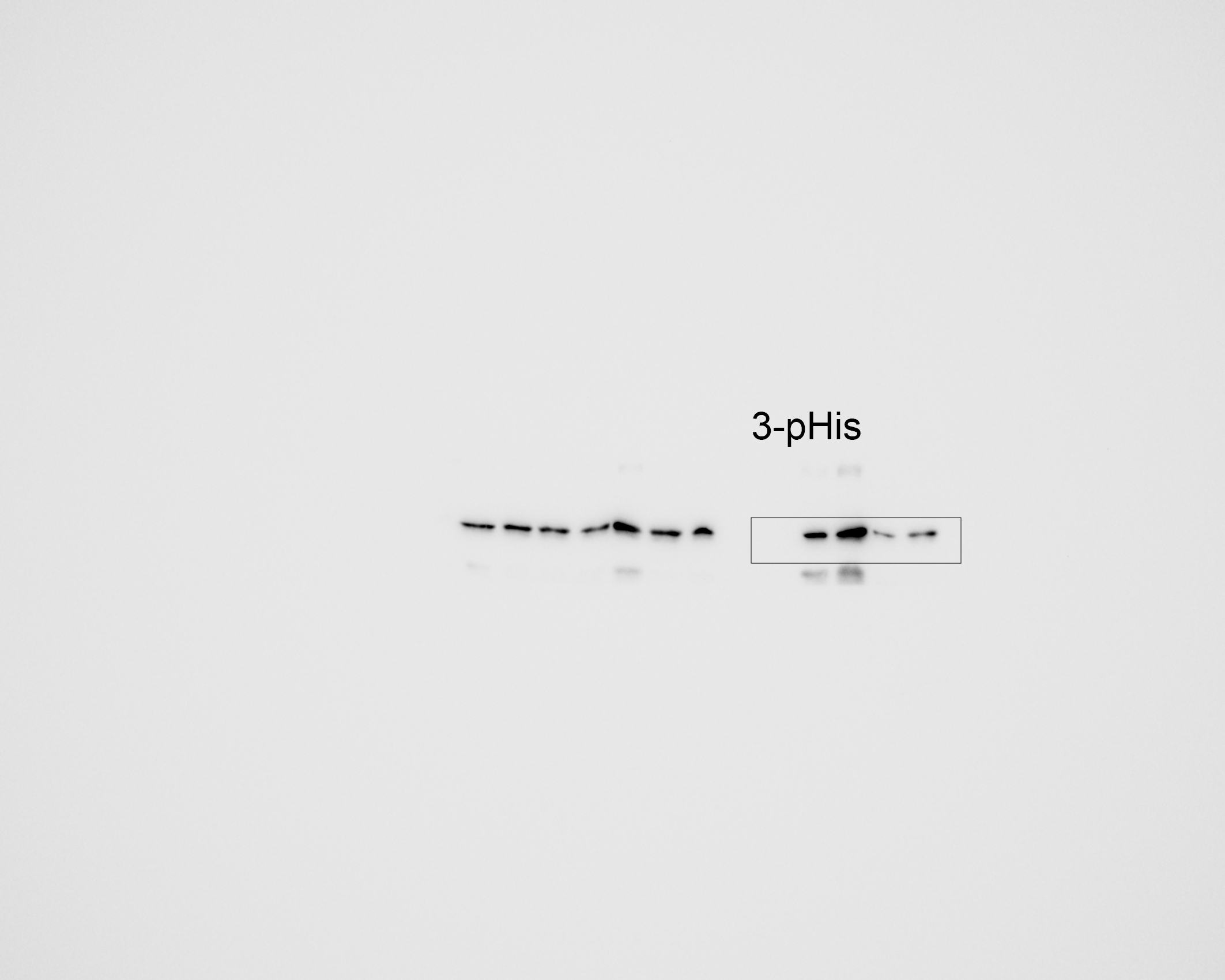

Supplement: Supplementary file 6 — Source data Fig. 4 [file 44318_2024_110_MOESM6_ESM.zip › Figure 4/4F/2-3-pHis.tif]

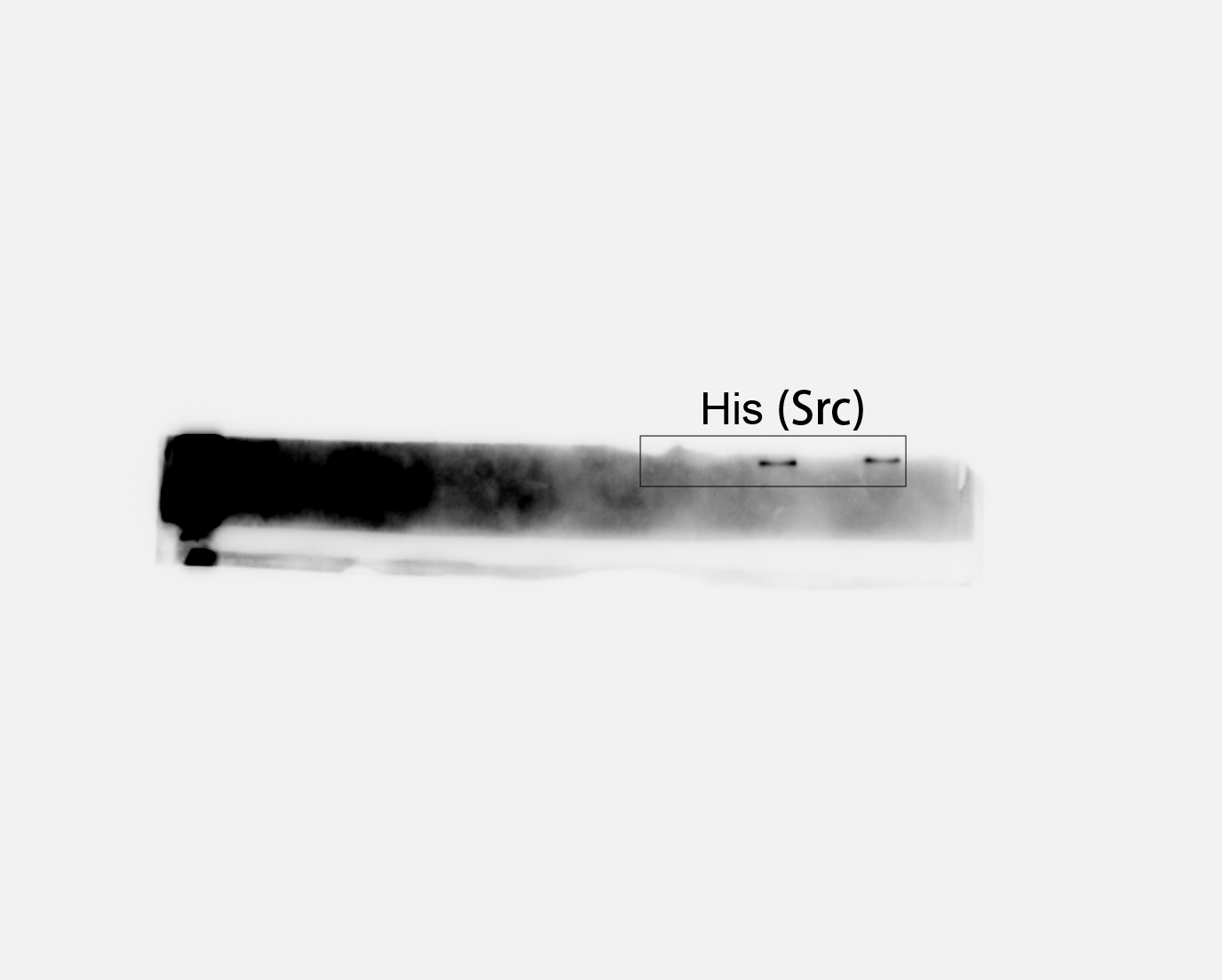

Supplement: Supplementary file 6 — Source data Fig. 4 [file 44318_2024_110_MOESM6_ESM.zip › Figure 4/4F/3-His (Src).Tif]

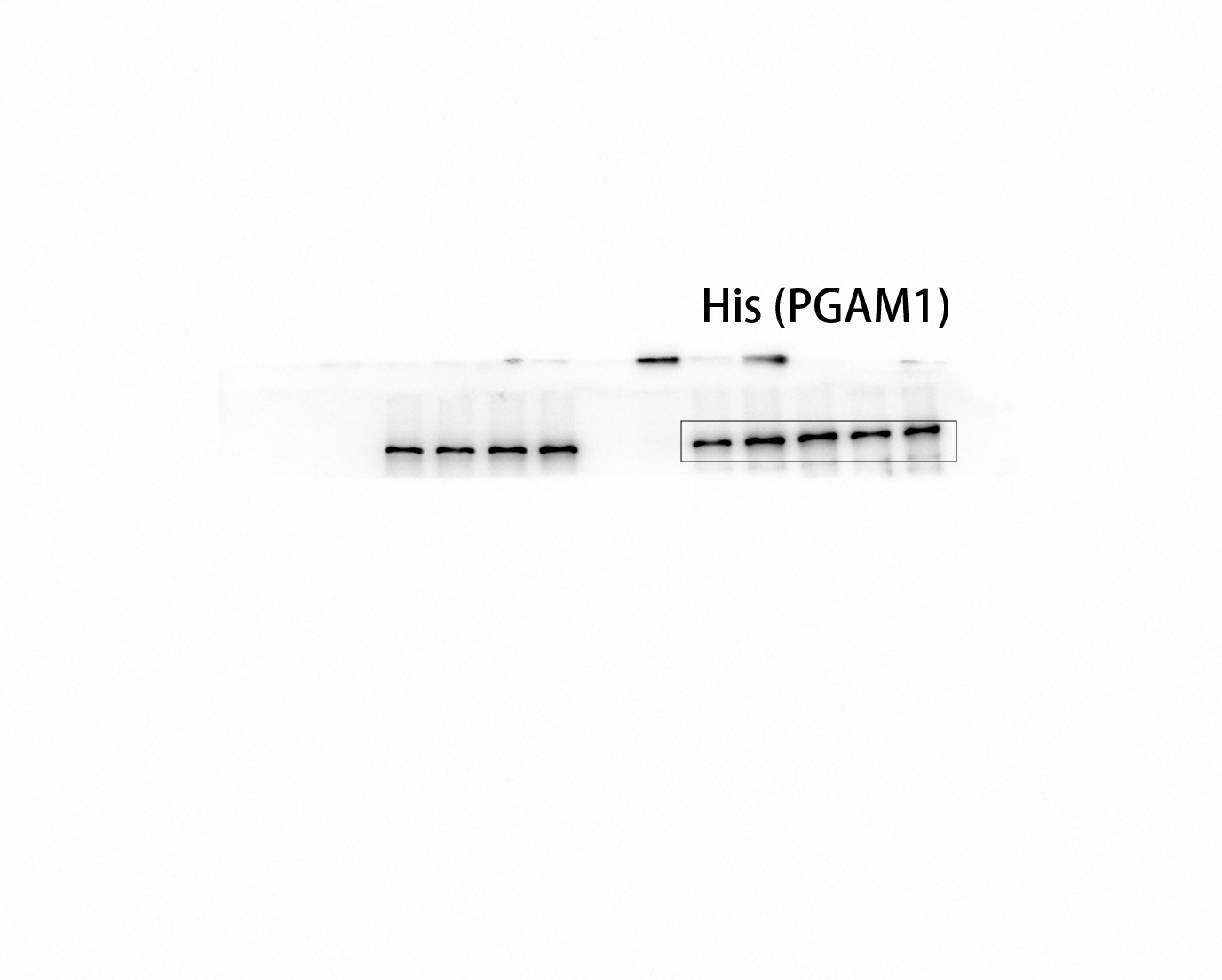

Supplement: Supplementary file 6 — Source data Fig. 4 [file 44318_2024_110_MOESM6_ESM.zip › Figure 4/4F/4-His (PGAM1).Tif]

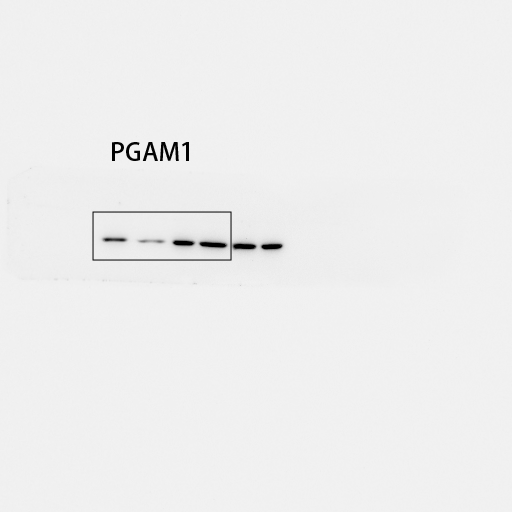

Supplement: Supplementary file 7 — Source data Fig. 5 [file 44318_2024_110_MOESM7_ESM.zip › Figure 5/5A/1-PGAM1.tif]

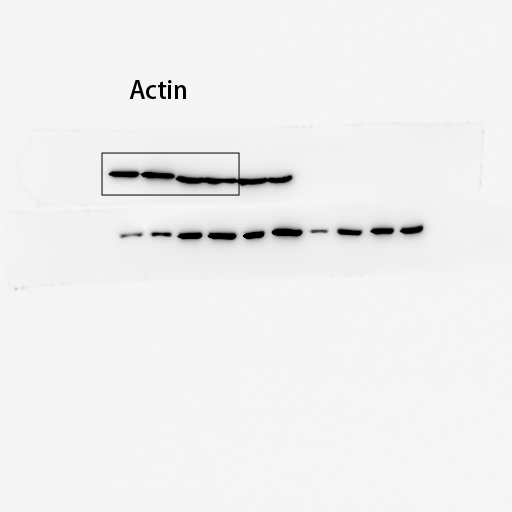

Supplement: Supplementary file 7 — Source data Fig. 5 [file 44318_2024_110_MOESM7_ESM.zip › Figure 5/5A/2-Actin.tif]

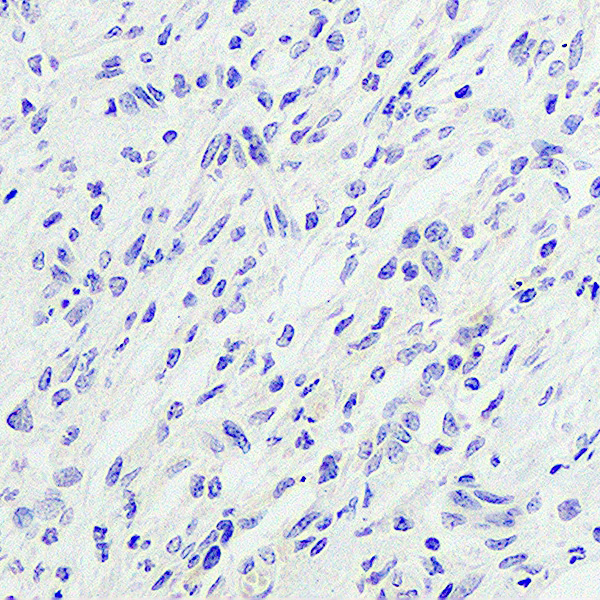

Supplement: Supplementary file 7 — Source data Fig. 5 [file 44318_2024_110_MOESM7_ESM.zip › Figure 5/5H/PGAM1 Y119F- PGAM1 pY119.jpg]

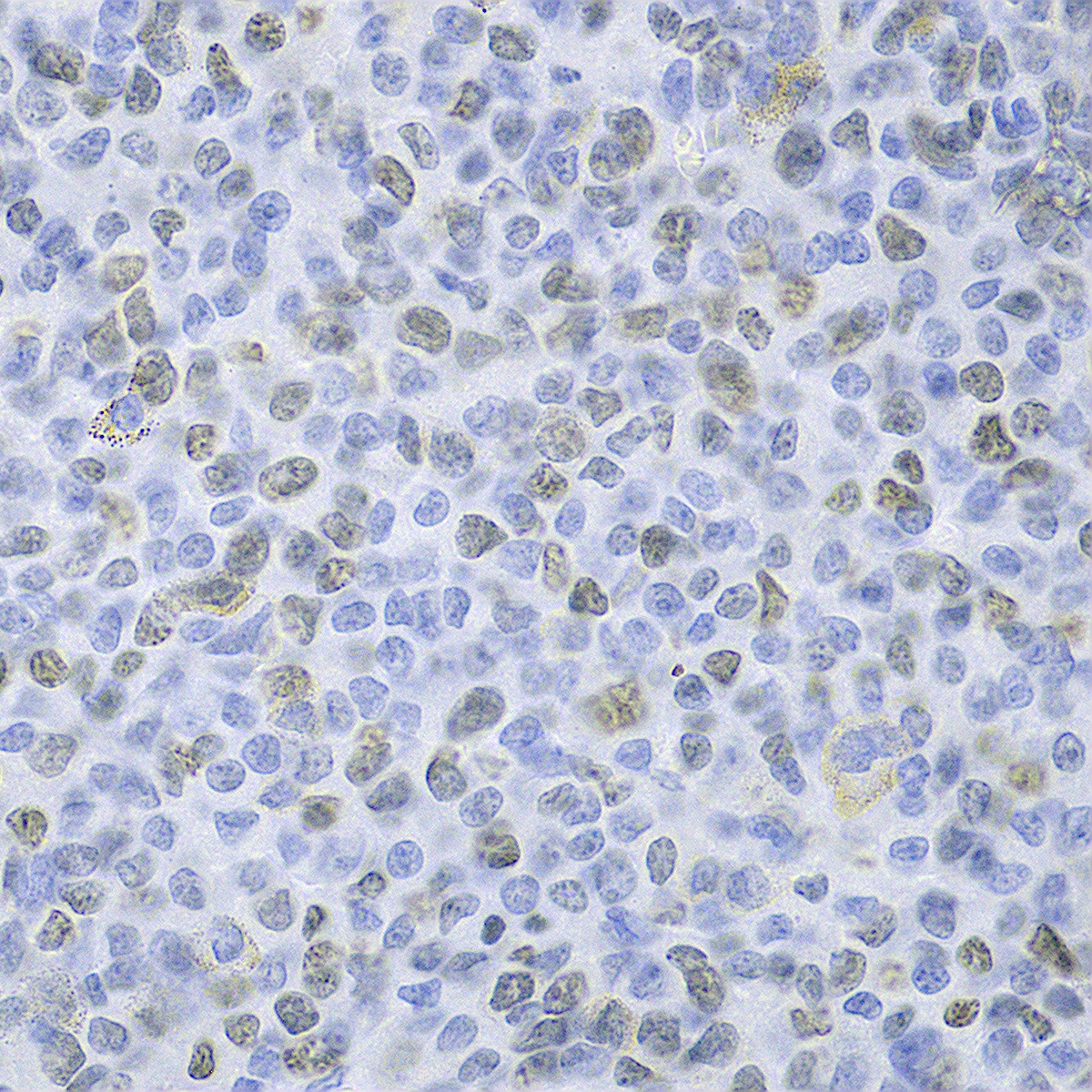

Supplement: Supplementary file 7 — Source data Fig. 5 [file 44318_2024_110_MOESM7_ESM.zip › Figure 5/5H/PGAM1 Y119F - Ki67.jpg]

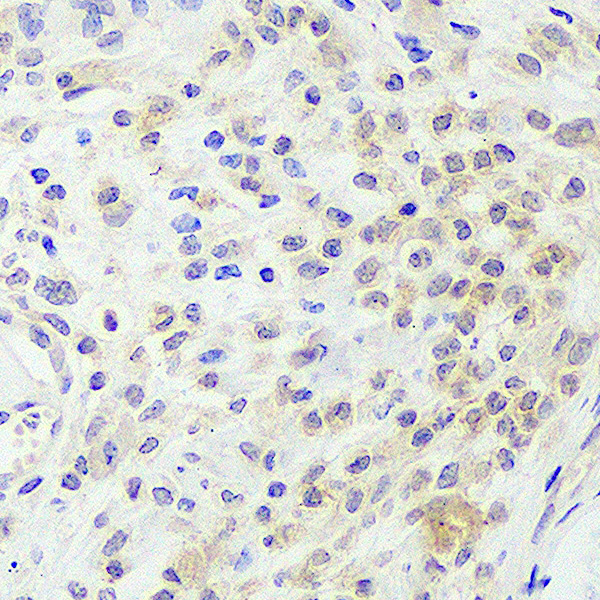

Supplement: Supplementary file 7 — Source data Fig. 5 [file 44318_2024_110_MOESM7_ESM.zip › Figure 5/5H/PGAM1 WT - PGAM1 pY119.jpg]

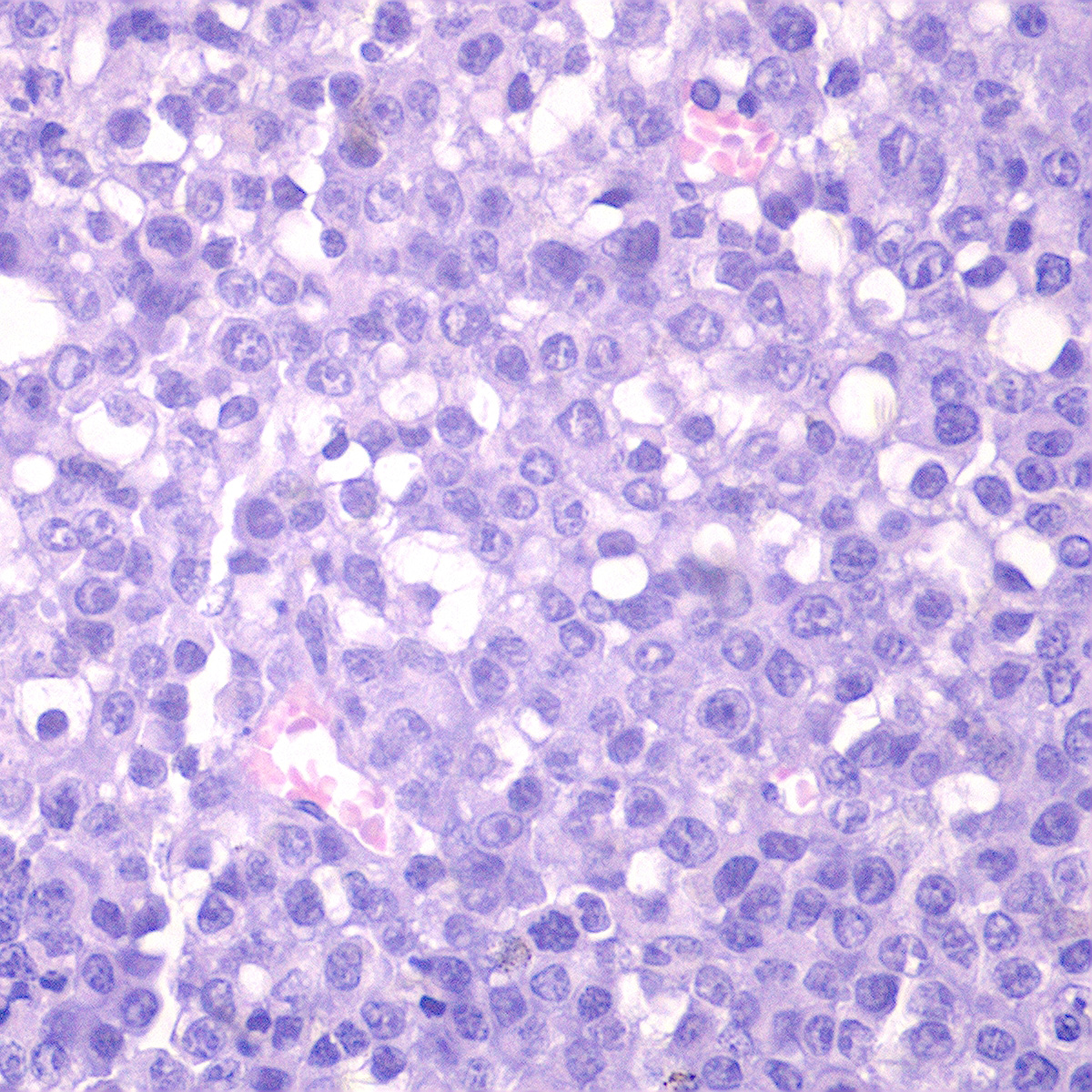

Supplement: Supplementary file 7 — Source data Fig. 5 [file 44318_2024_110_MOESM7_ESM.zip › Figure 5/5H/PGAM1 Y119F - HE.jpg]

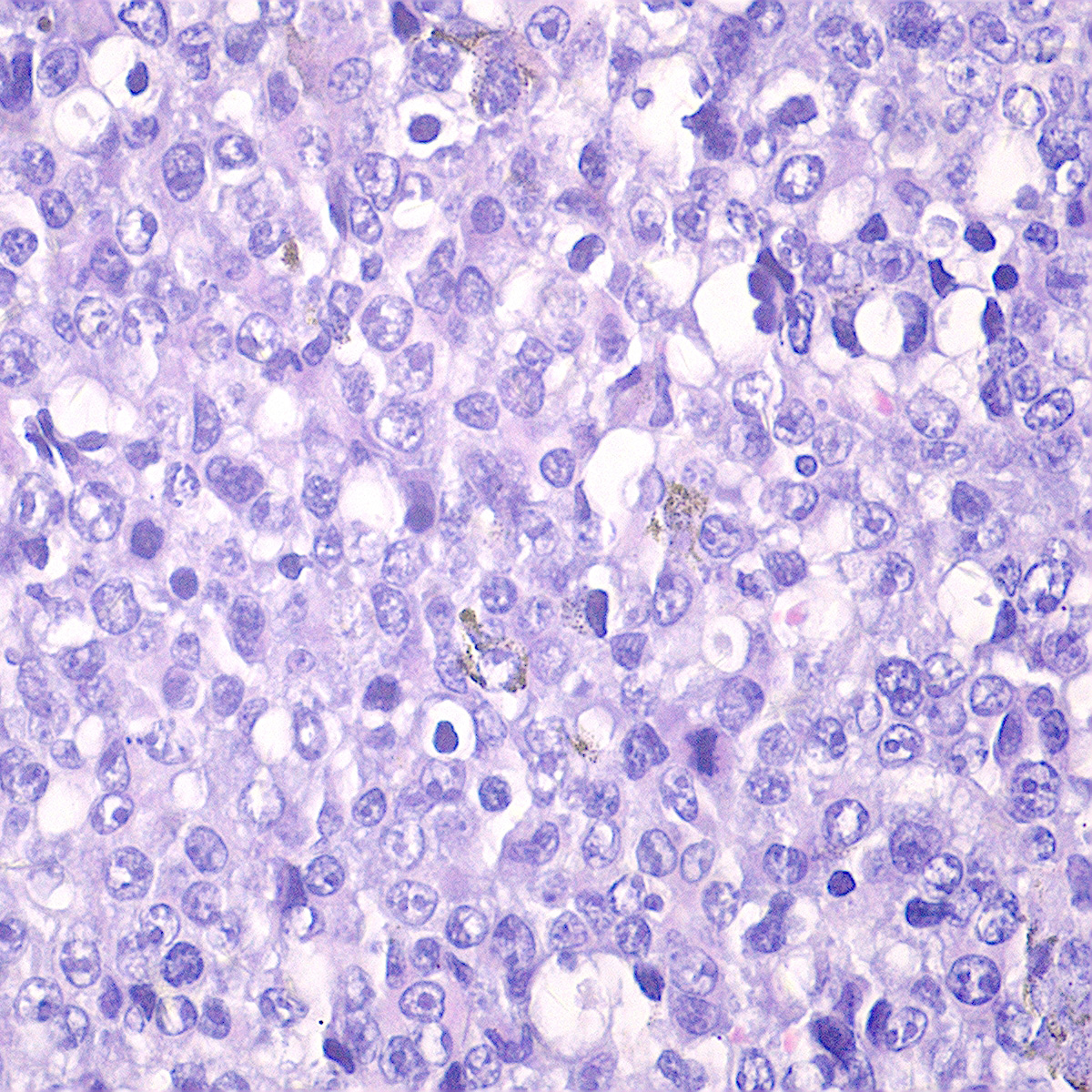

Supplement: Supplementary file 7 — Source data Fig. 5 [file 44318_2024_110_MOESM7_ESM.zip › Figure 5/5H/PGAM1 WT - HE.jpg]

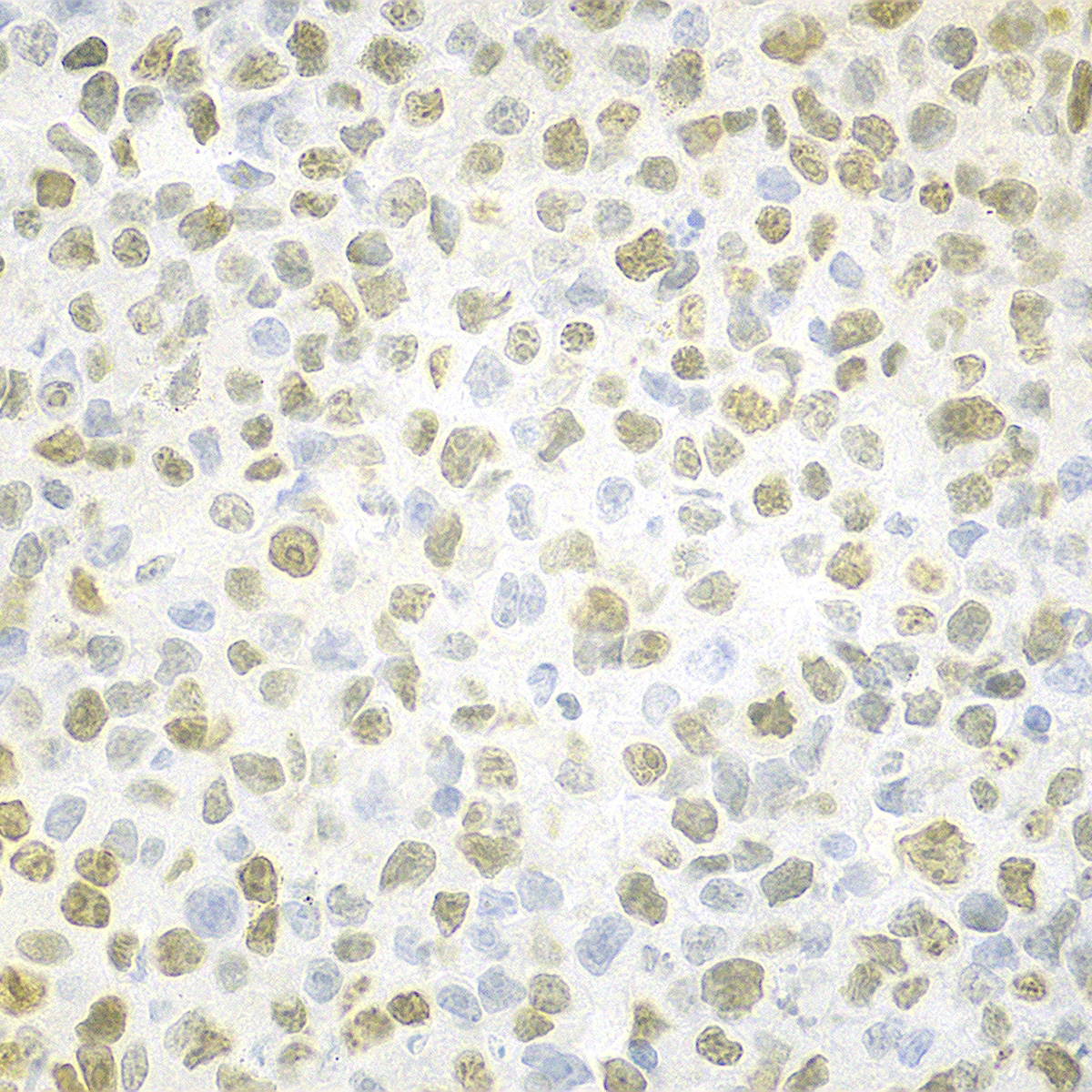

Supplement: Supplementary file 7 — Source data Fig. 5 [file 44318_2024_110_MOESM7_ESM.zip › Figure 5/5H/PGAM1 WT - Ki67.jpg]

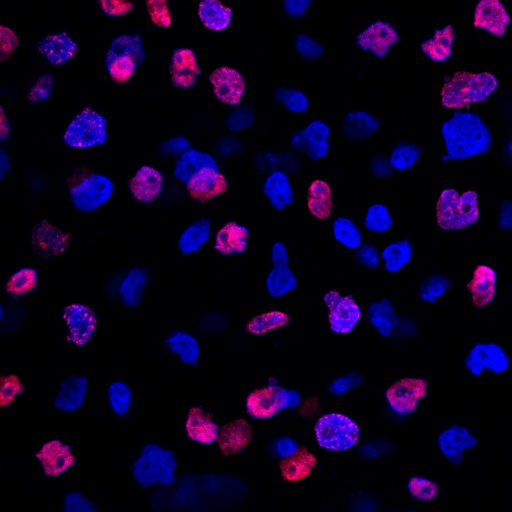

Supplement: Supplementary file 7 — Source data Fig. 5 [file 44318_2024_110_MOESM7_ESM.zip › Figure 5/5C/PGAM1 WT - Merge.jpg]

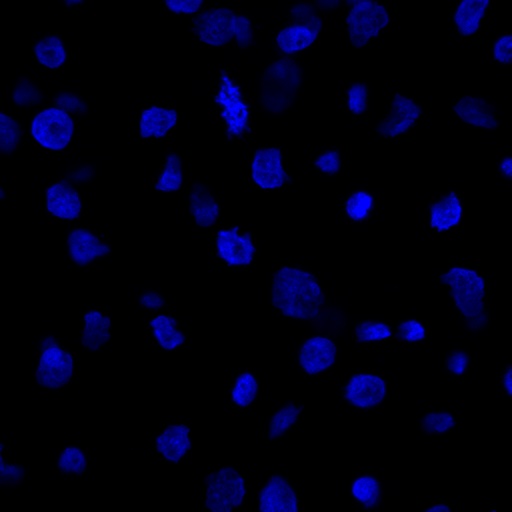

Supplement: Supplementary file 7 — Source data Fig. 5 [file 44318_2024_110_MOESM7_ESM.zip › Figure 5/5C/PGAM1 Y119F - DAPI.jpg]

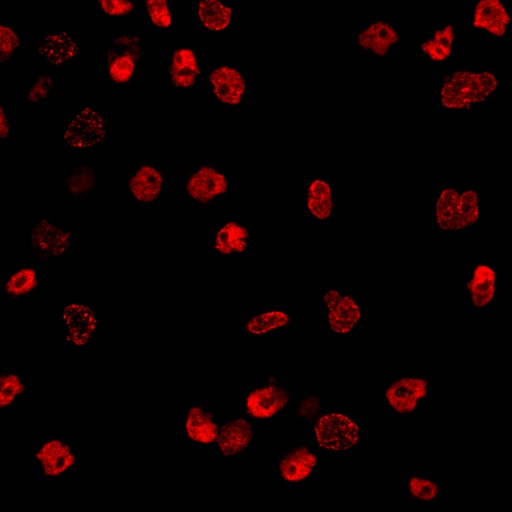

Supplement: Supplementary file 7 — Source data Fig. 5 [file 44318_2024_110_MOESM7_ESM.zip › Figure 5/5C/PGAM1 WT - EdU.jpg]

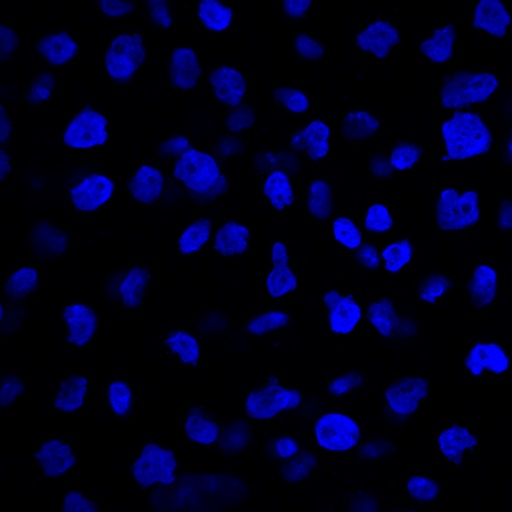

Supplement: Supplementary file 7 — Source data Fig. 5 [file 44318_2024_110_MOESM7_ESM.zip › Figure 5/5C/PGAM1 WT - DAPI.jpg]

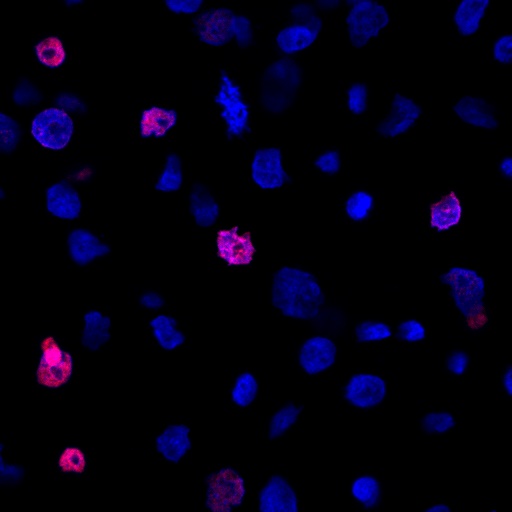

Supplement: Supplementary file 7 — Source data Fig. 5 [file 44318_2024_110_MOESM7_ESM.zip › Figure 5/5C/PGAM1 Y119F - Merge.jpg]

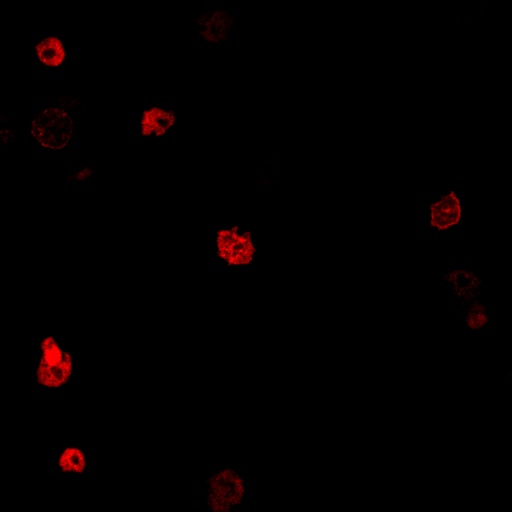

Supplement: Supplementary file 7 — Source data Fig. 5 [file 44318_2024_110_MOESM7_ESM.zip › Figure 5/5C/PGAM1 Y119F - EdU.jpg]

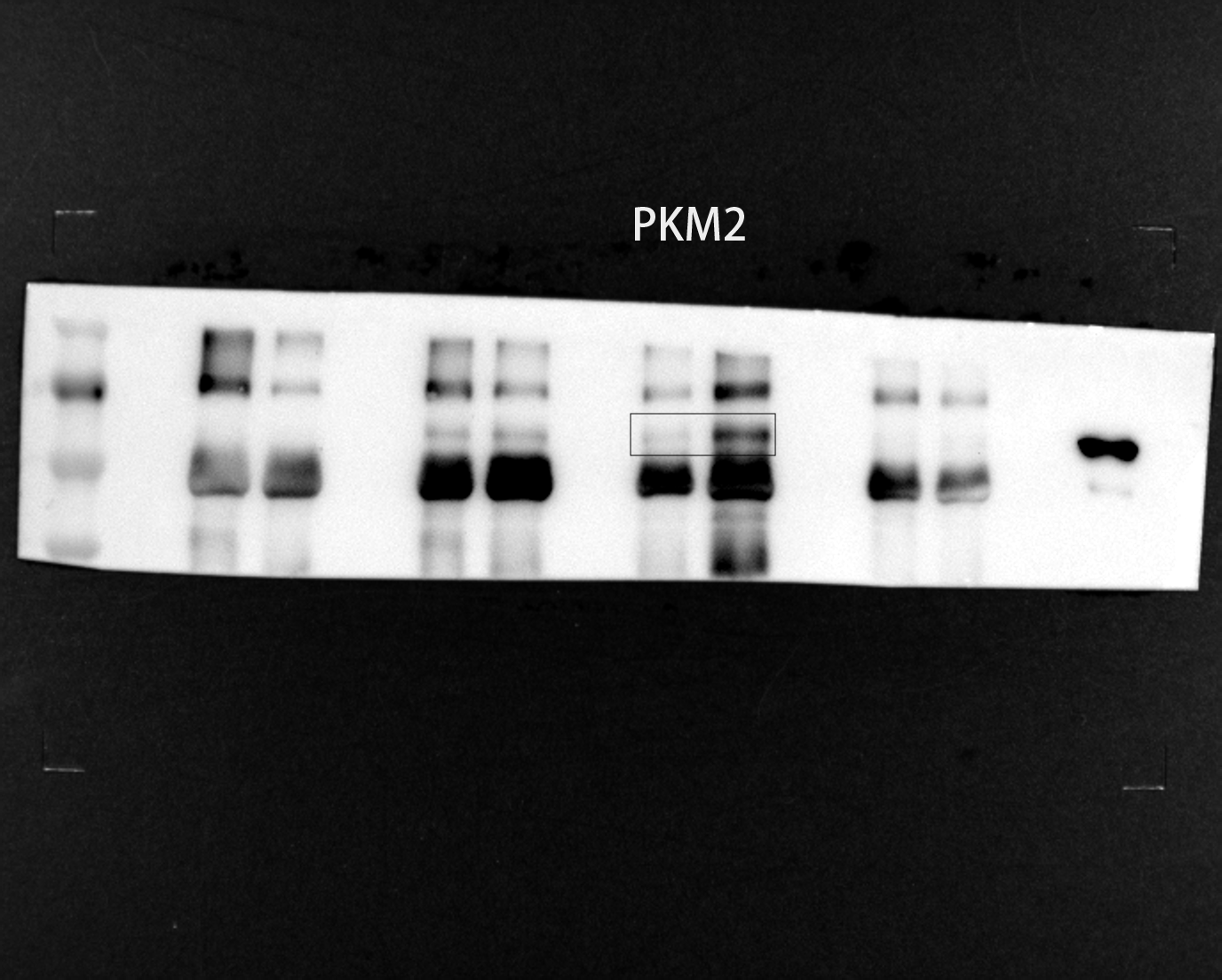

Supplement: Supplementary file 8 — Source data Fig. 6 [file 44318_2024_110_MOESM8_ESM.zip › Figure 6/6C/13.Tif]

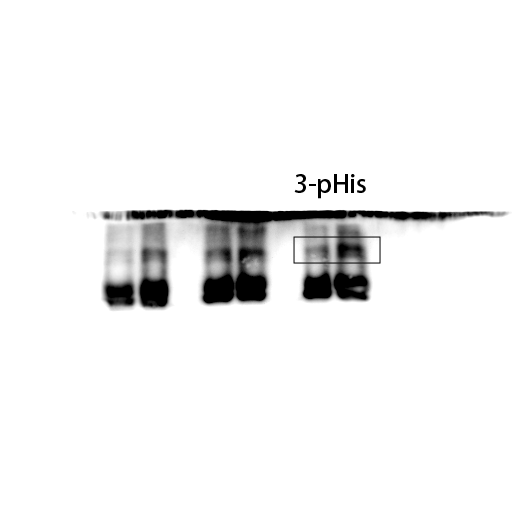

Supplement: Supplementary file 8 — Source data Fig. 6 [file 44318_2024_110_MOESM8_ESM.zip › Figure 6/6C/6-3-pHis.tif]

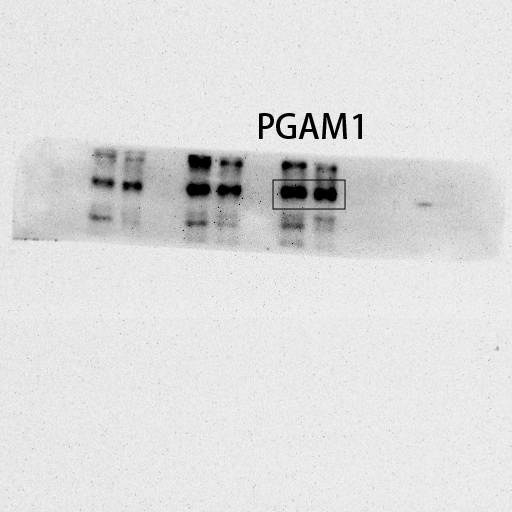

Supplement: Supplementary file 8 — Source data Fig. 6 [file 44318_2024_110_MOESM8_ESM.zip › Figure 6/6C/26-PGAM1.tif]

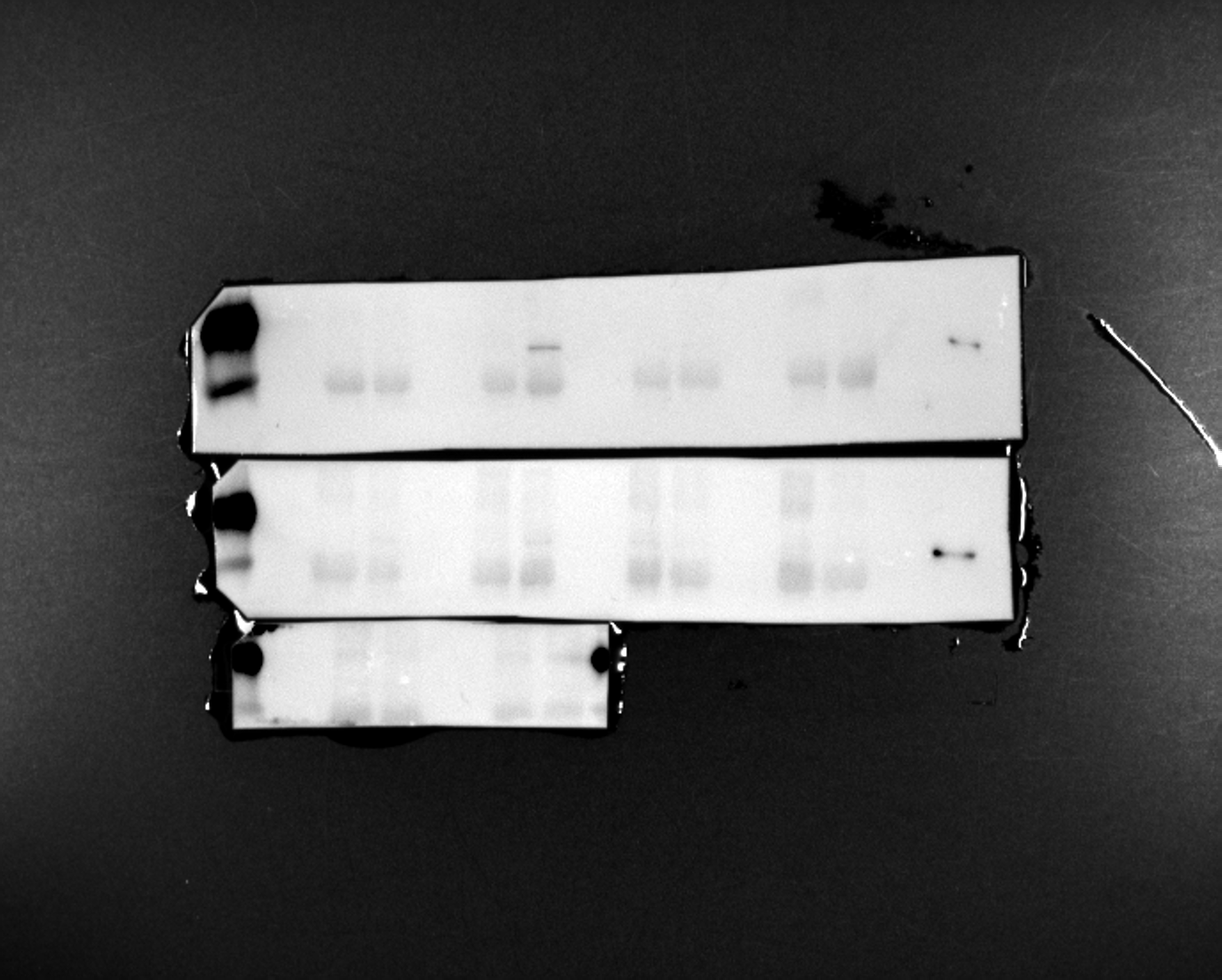

Supplement: Supplementary file 8 — Source data Fig. 6 [file 44318_2024_110_MOESM8_ESM.zip › Figure 6/6C/15.Tif]

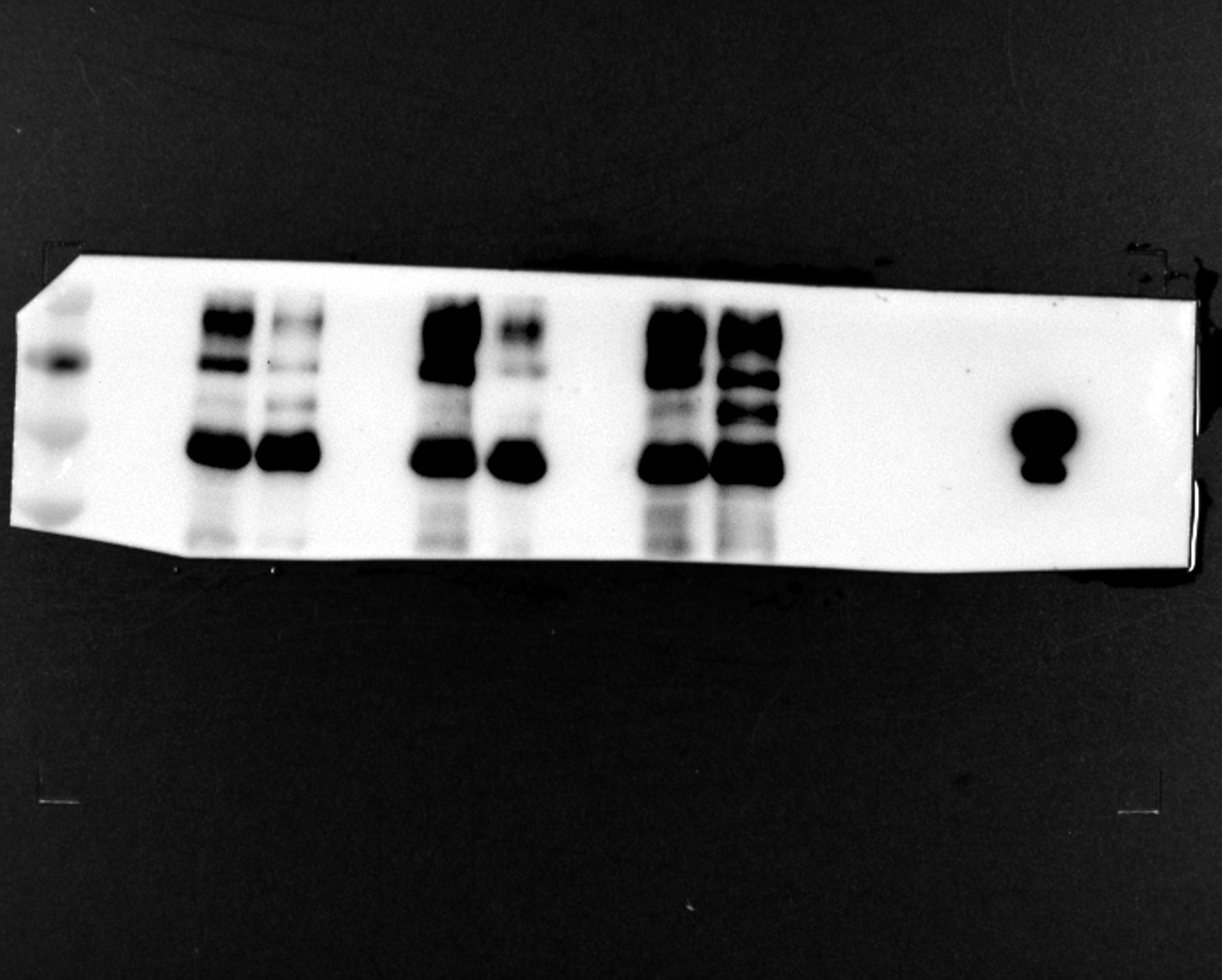

Supplement: Supplementary file 8 — Source data Fig. 6 [file 44318_2024_110_MOESM8_ESM.zip › Figure 6/6C/14.Tif]

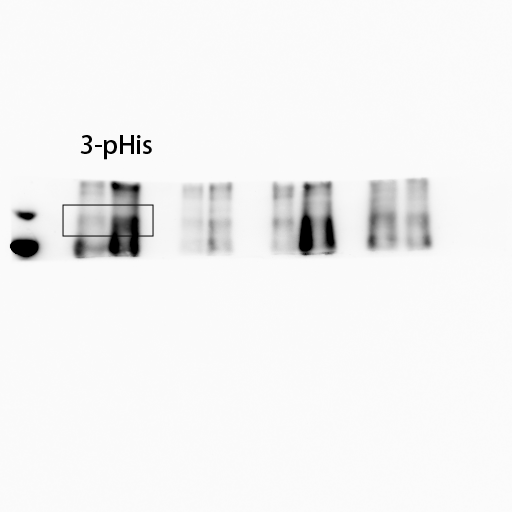

Supplement: Supplementary file 8 — Source data Fig. 6 [file 44318_2024_110_MOESM8_ESM.zip › Figure 6/6C/10-3-pHis.tif]

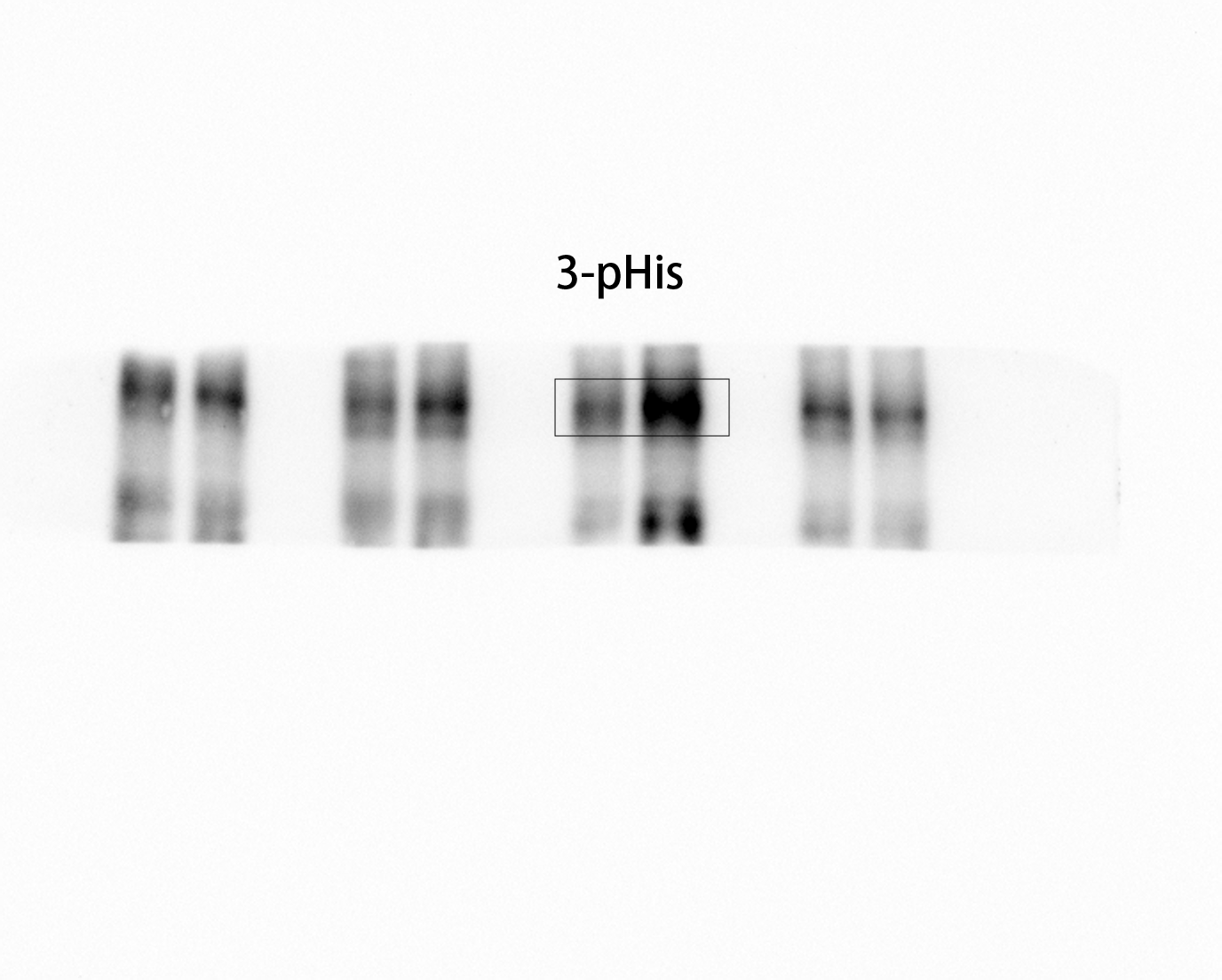

Supplement: Supplementary file 8 — Source data Fig. 6 [file 44318_2024_110_MOESM8_ESM.zip › Figure 6/6C/3-3-pHis.Tif]

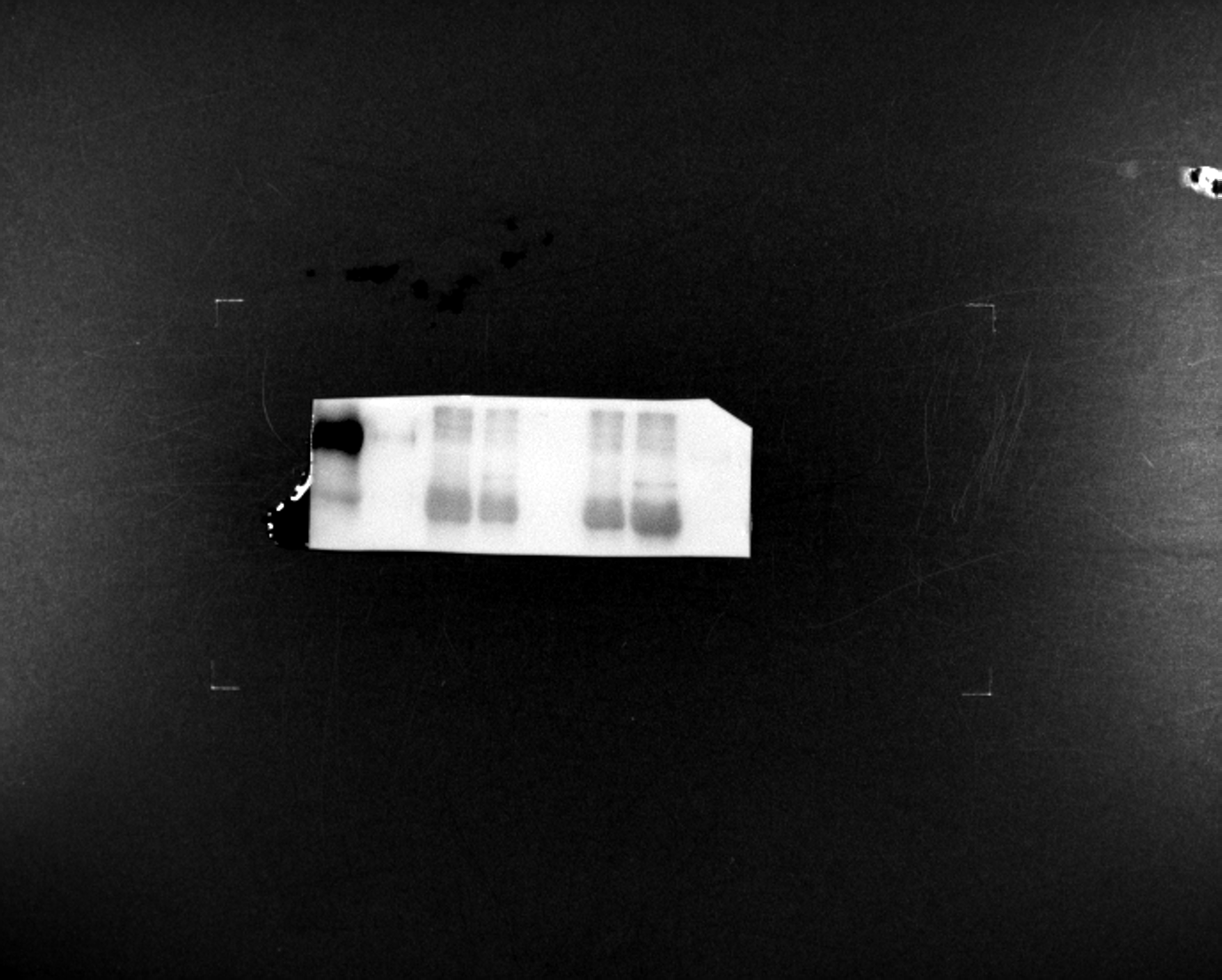

Supplement: Supplementary file 8 — Source data Fig. 6 [file 44318_2024_110_MOESM8_ESM.zip › Figure 6/6C/17.Tif]

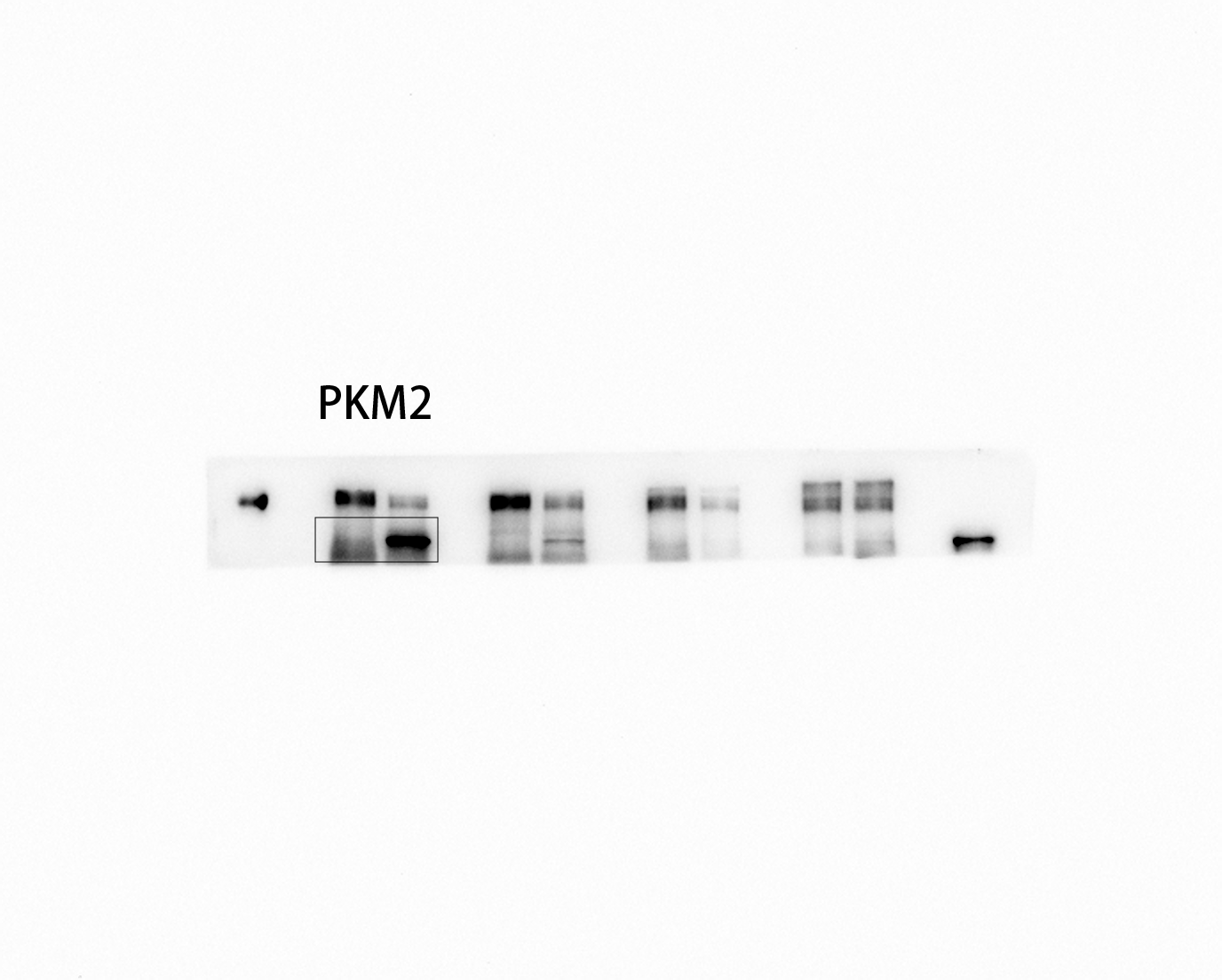

Supplement: Supplementary file 8 — Source data Fig. 6 [file 44318_2024_110_MOESM8_ESM.zip › Figure 6/6C/11-PKM2.Tif]

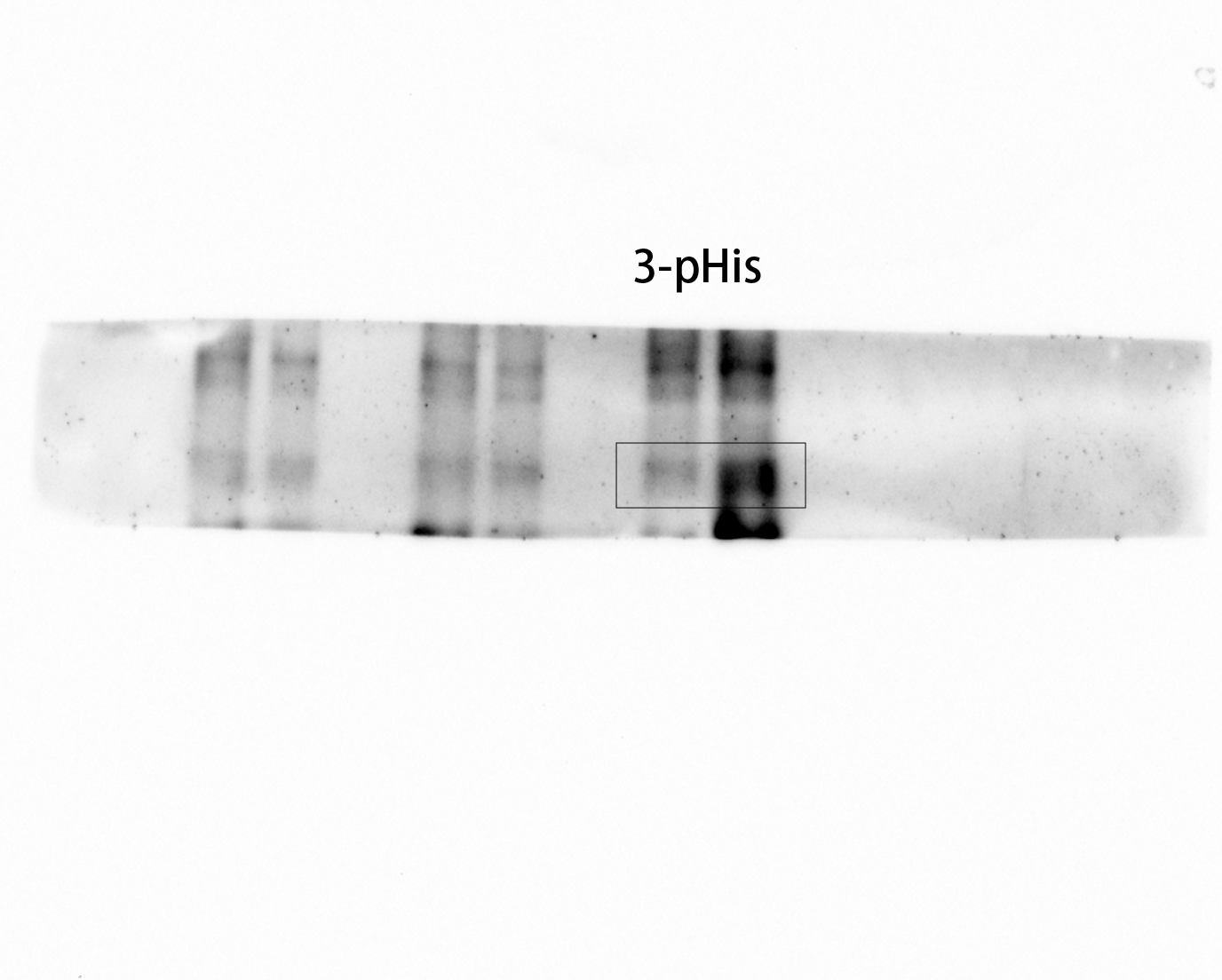

Supplement: Supplementary file 8 — Source data Fig. 6 [file 44318_2024_110_MOESM8_ESM.zip › Figure 6/6C/4-3-pHis.Tif]

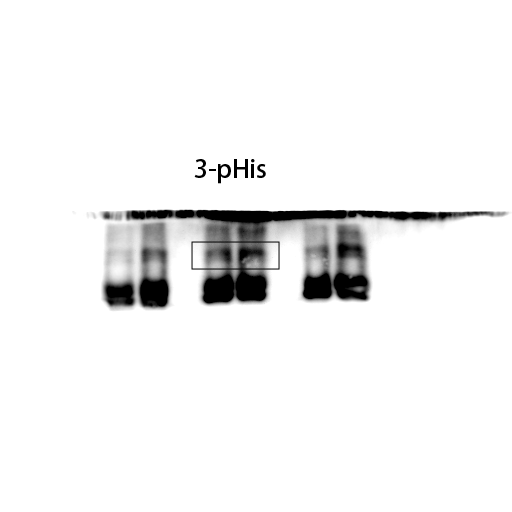

Supplement: Supplementary file 8 — Source data Fig. 6 [file 44318_2024_110_MOESM8_ESM.zip › Figure 6/6C/9-3-pHis.tif]

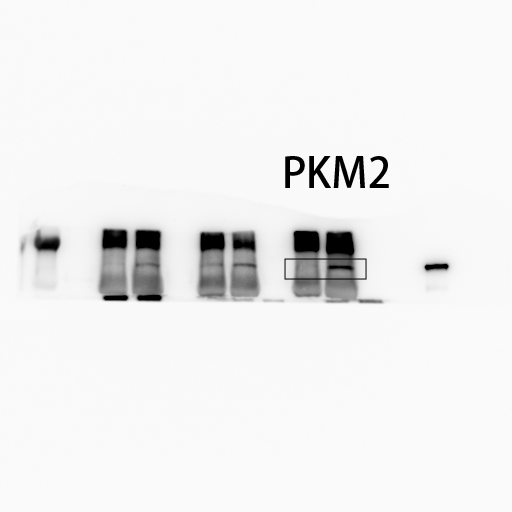

Supplement: Supplementary file 8 — Source data Fig. 6 [file 44318_2024_110_MOESM8_ESM.zip › Figure 6/6C/16-PKM2.tif]

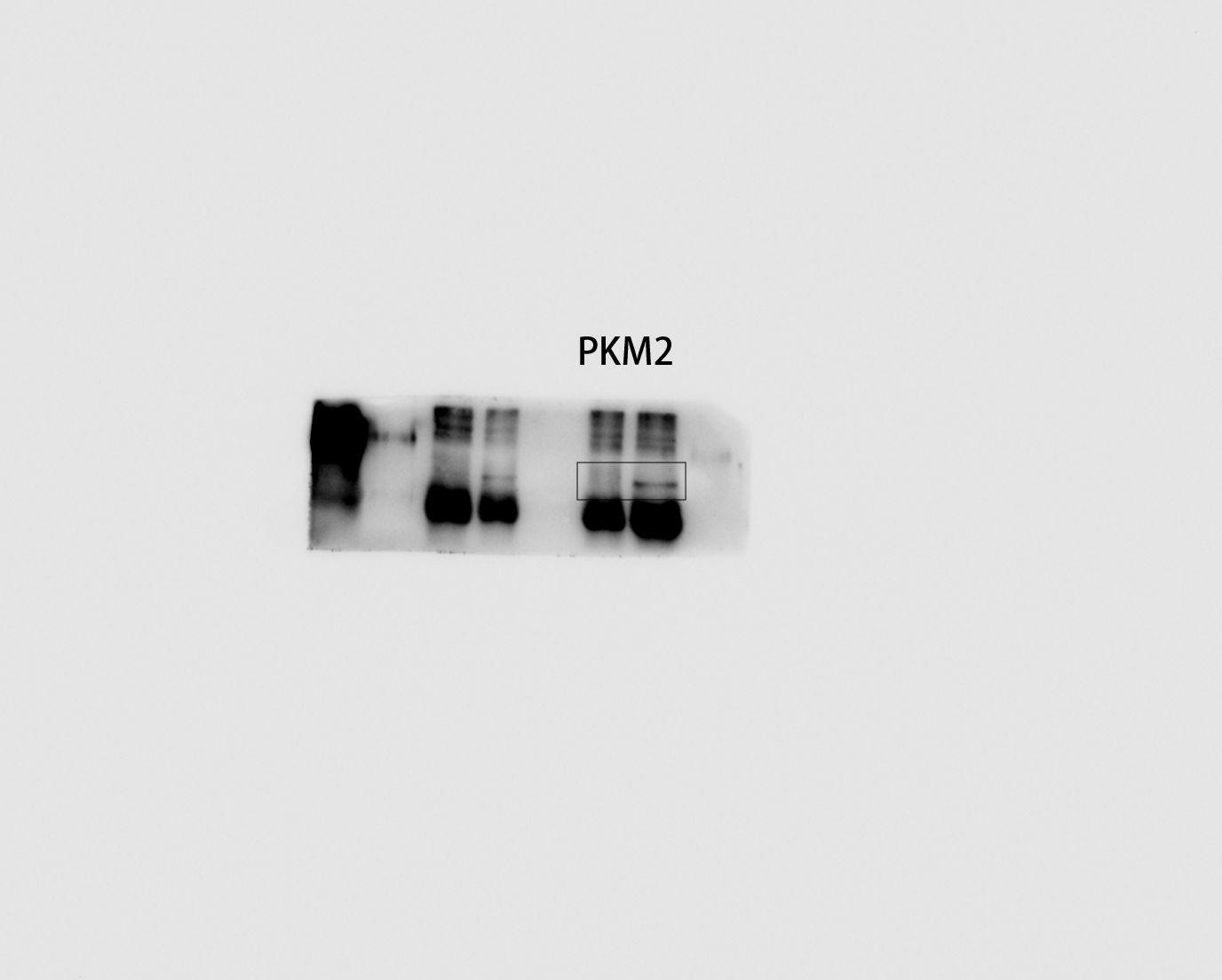

Supplement: Supplementary file 8 — Source data Fig. 6 [file 44318_2024_110_MOESM8_ESM.zip › Figure 6/6C/17-PKM2.Tif]

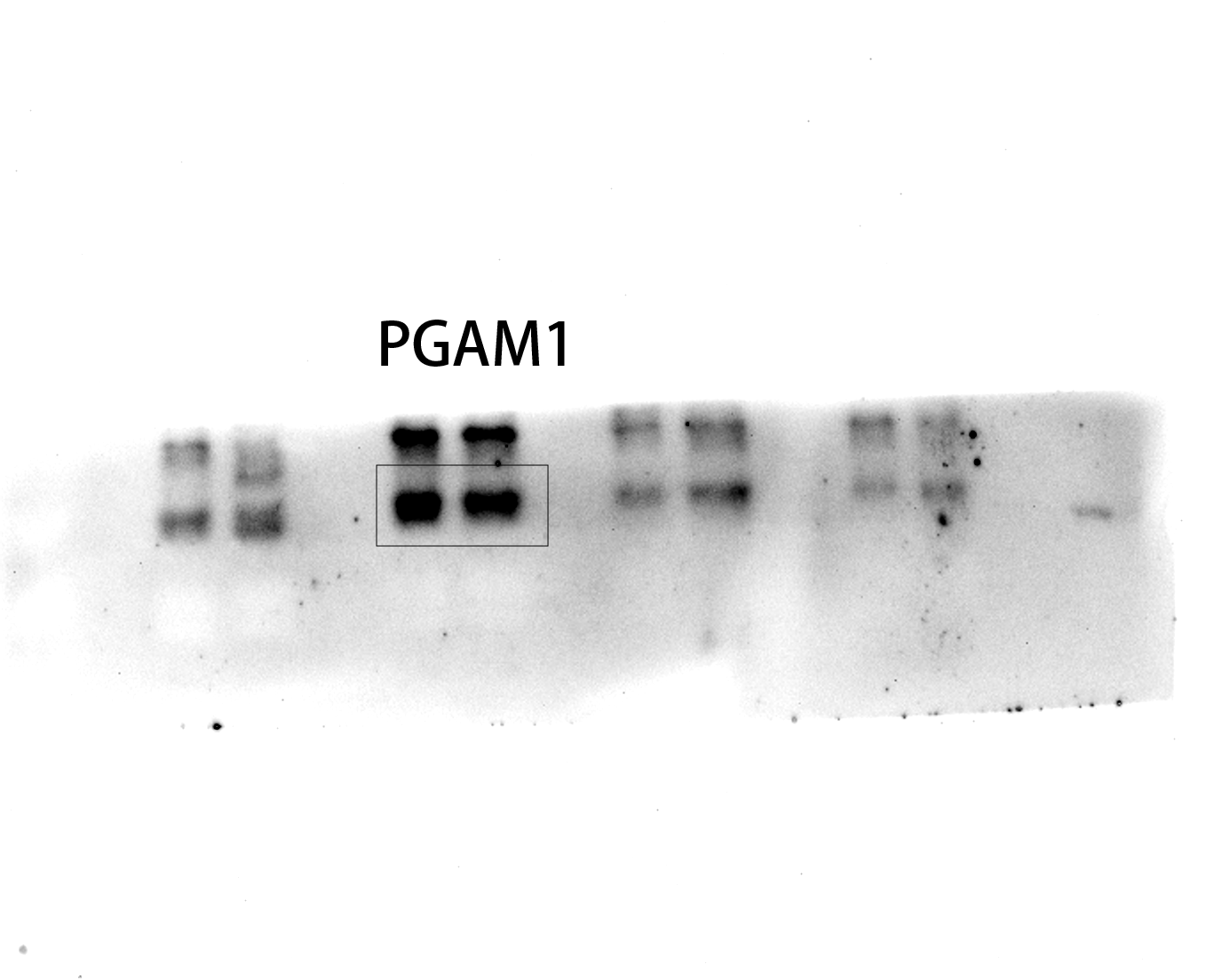

Supplement: Supplementary file 8 — Source data Fig. 6 [file 44318_2024_110_MOESM8_ESM.zip › Figure 6/6C/27-PGAM1.Tif]

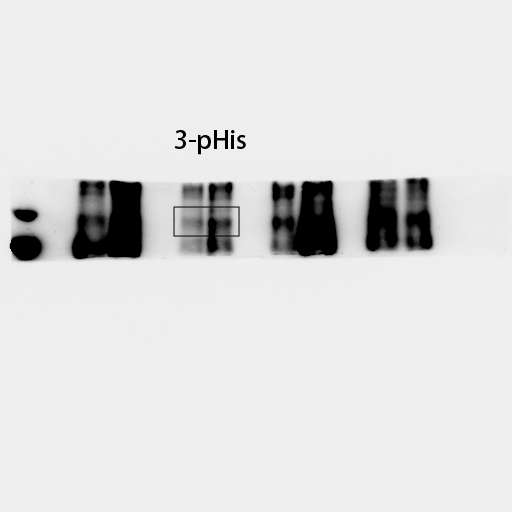

Supplement: Supplementary file 8 — Source data Fig. 6 [file 44318_2024_110_MOESM8_ESM.zip › Figure 6/6C/1-3-pHis.tif]

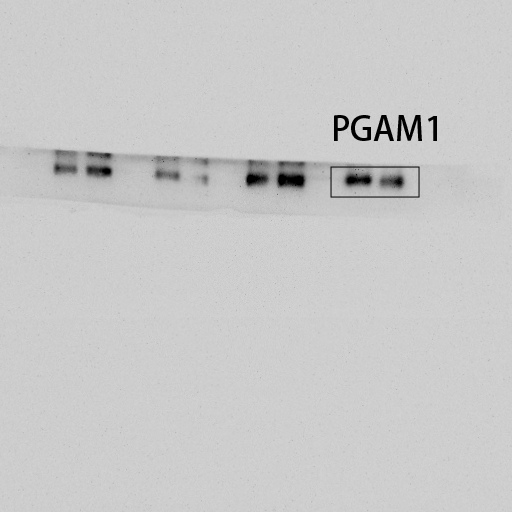

Supplement: Supplementary file 8 — Source data Fig. 6 [file 44318_2024_110_MOESM8_ESM.zip › Figure 6/6C/21-PGAM1.tif]

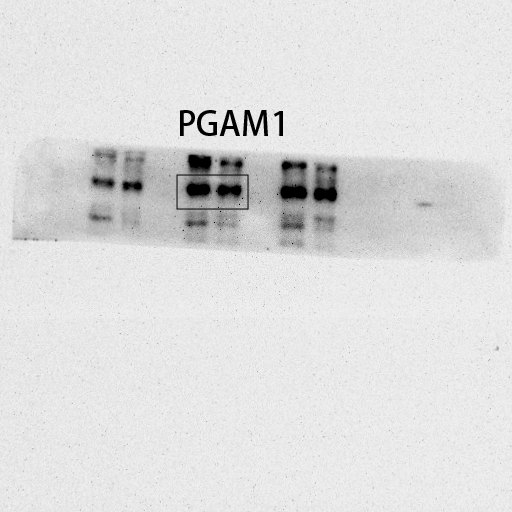

Supplement: Supplementary file 8 — Source data Fig. 6 [file 44318_2024_110_MOESM8_ESM.zip › Figure 6/6C/29-PGAM1.tif]

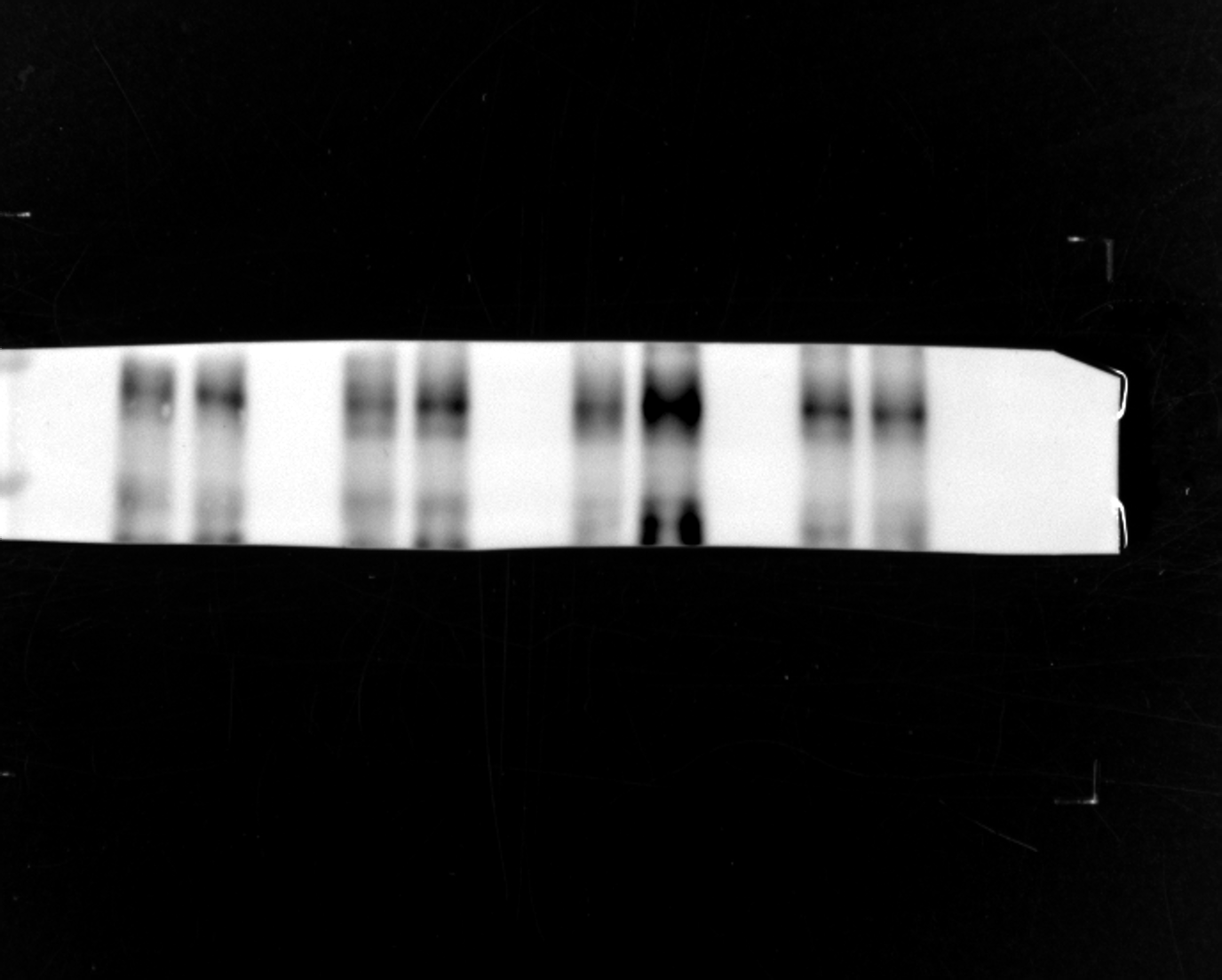

Supplement: Supplementary file 8 — Source data Fig. 6 [file 44318_2024_110_MOESM8_ESM.zip › Figure 6/6C/3.Tif]

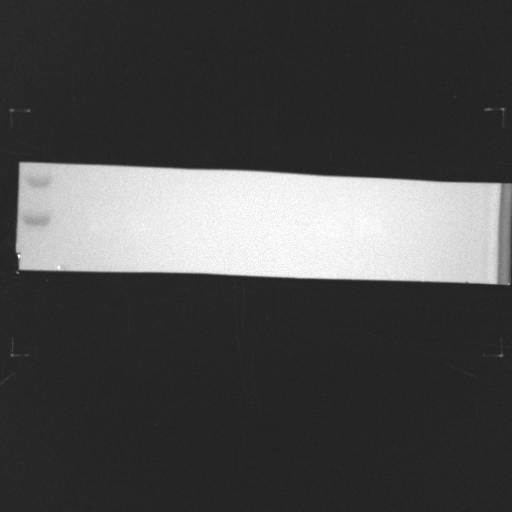

Supplement: Supplementary file 8 — Source data Fig. 6 [file 44318_2024_110_MOESM8_ESM.zip › Figure 6/6C/2.tif]

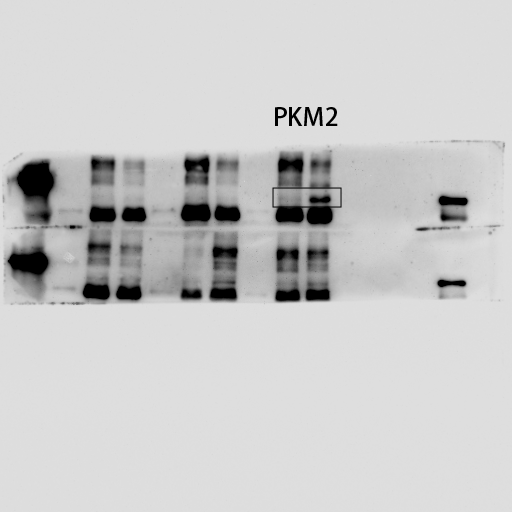

Supplement: Supplementary file 8 — Source data Fig. 6 [file 44318_2024_110_MOESM8_ESM.zip › Figure 6/6C/12-PKM2.tif]

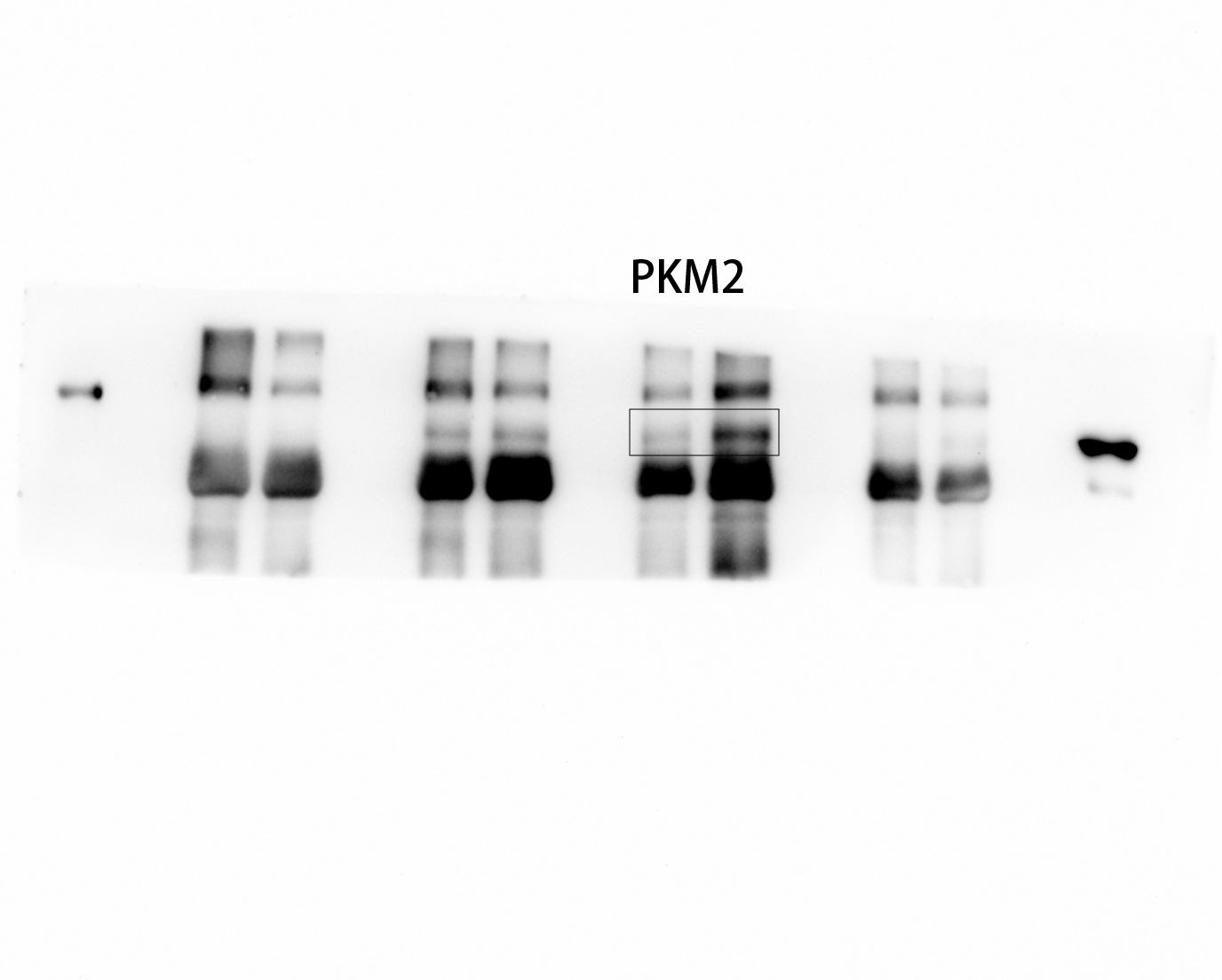

Supplement: Supplementary file 8 — Source data Fig. 6 [file 44318_2024_110_MOESM8_ESM.zip › Figure 6/6C/13-PKM2.Tif]

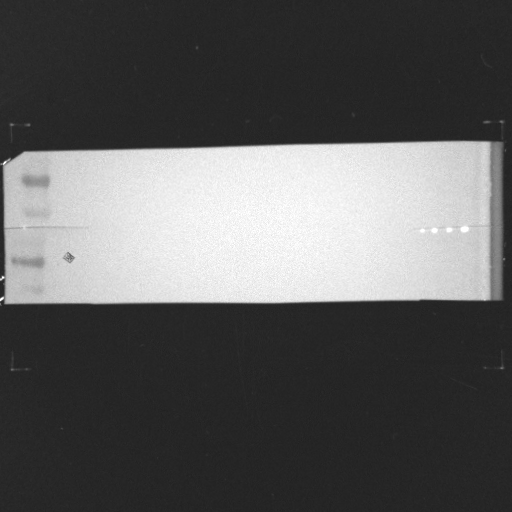

Supplement: Supplementary file 8 — Source data Fig. 6 [file 44318_2024_110_MOESM8_ESM.zip › Figure 6/6C/12 20.tif]

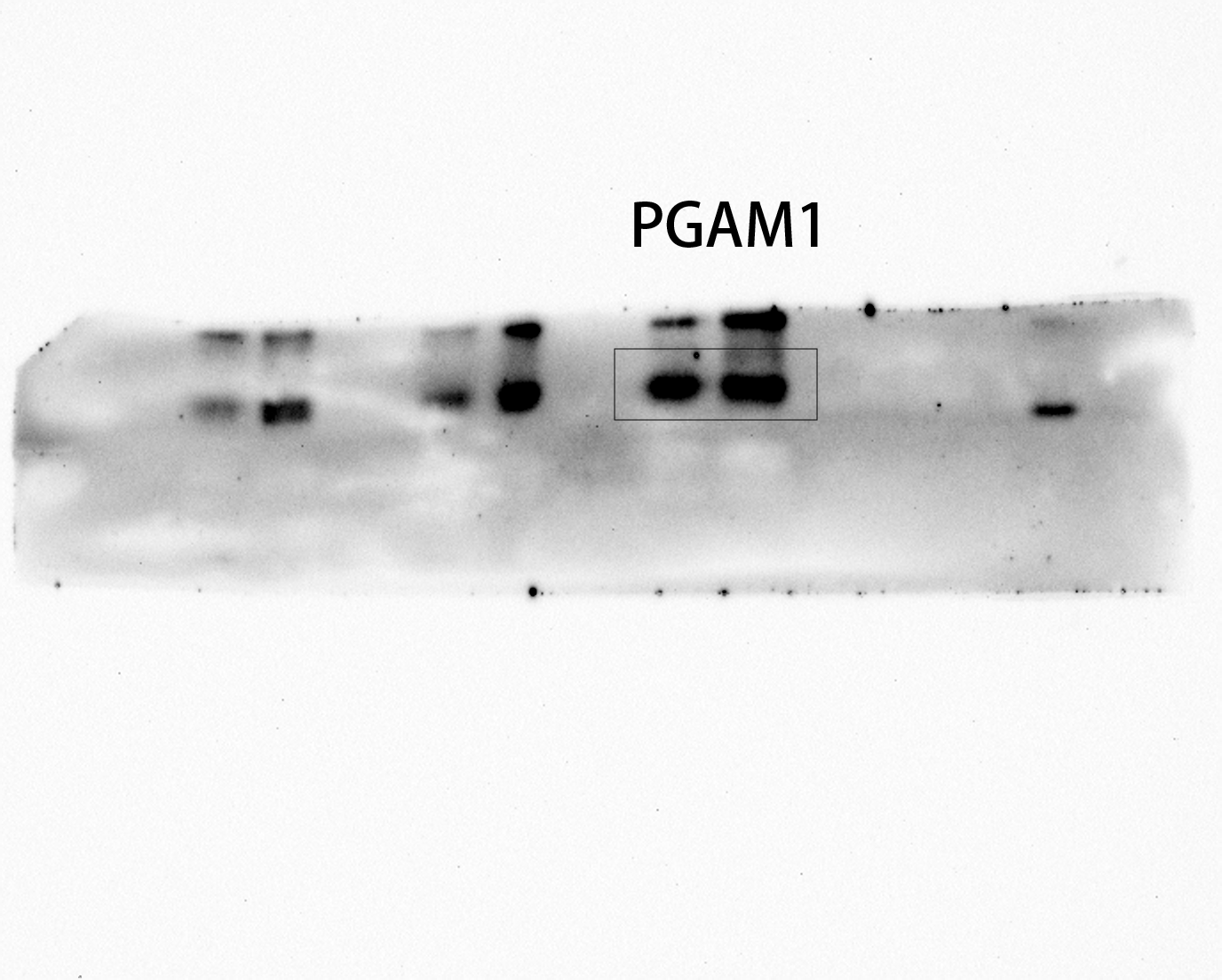

Supplement: Supplementary file 8 — Source data Fig. 6 [file 44318_2024_110_MOESM8_ESM.zip › Figure 6/6C/24-PGAM1.Tif]

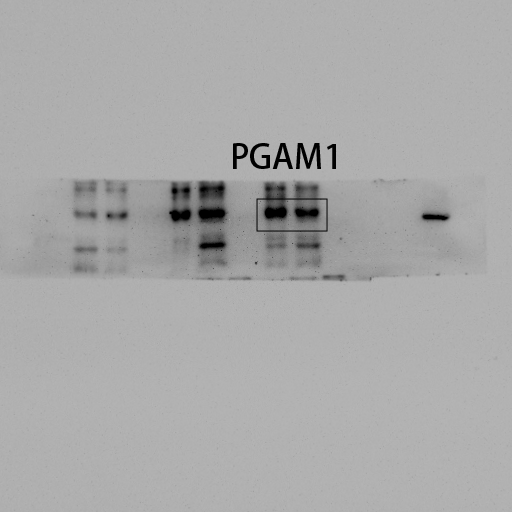

Supplement: Supplementary file 8 — Source data Fig. 6 [file 44318_2024_110_MOESM8_ESM.zip › Figure 6/6C/30-PGAM1.tif]

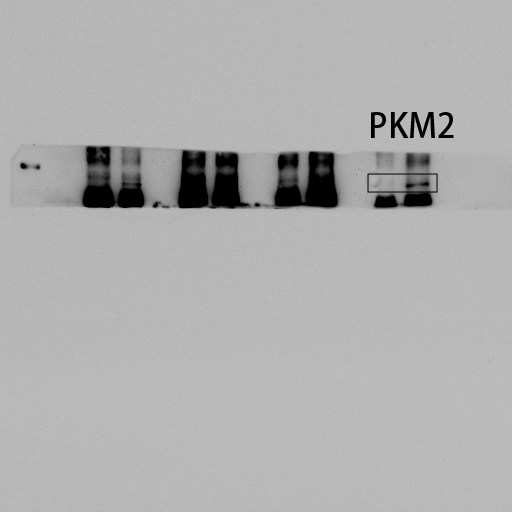

Supplement: Supplementary file 8 — Source data Fig. 6 [file 44318_2024_110_MOESM8_ESM.zip › Figure 6/6C/18-PKM2.tif]

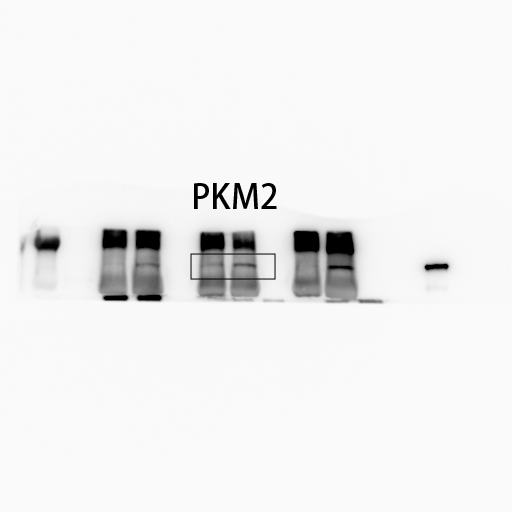

Supplement: Supplementary file 8 — Source data Fig. 6 [file 44318_2024_110_MOESM8_ESM.zip › Figure 6/6C/19-PKM2.tif]

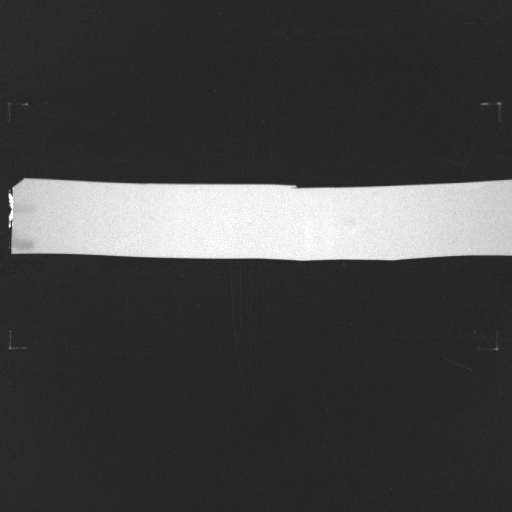

Supplement: Supplementary file 8 — Source data Fig. 6 [file 44318_2024_110_MOESM8_ESM.zip › Figure 6/6C/5 7.tif]

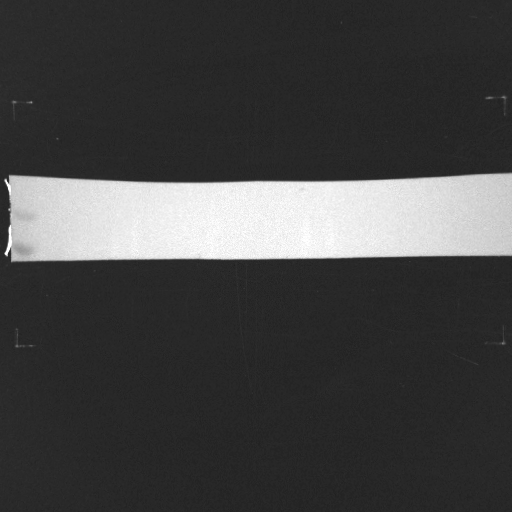

Supplement: Supplementary file 8 — Source data Fig. 6 [file 44318_2024_110_MOESM8_ESM.zip › Figure 6/6C/1.tif]

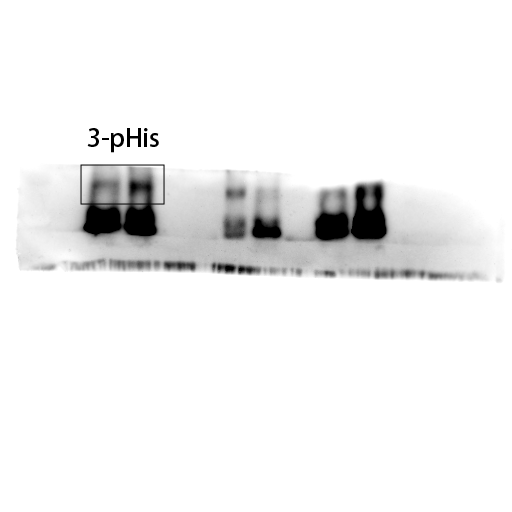

Supplement: Supplementary file 8 — Source data Fig. 6 [file 44318_2024_110_MOESM8_ESM.zip › Figure 6/6C/2-3-pHis.tif]

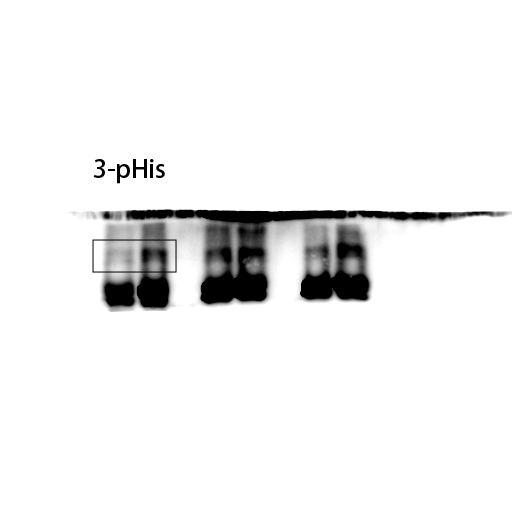

Supplement: Supplementary file 8 — Source data Fig. 6 [file 44318_2024_110_MOESM8_ESM.zip › Figure 6/6C/8-3pHis.tif]

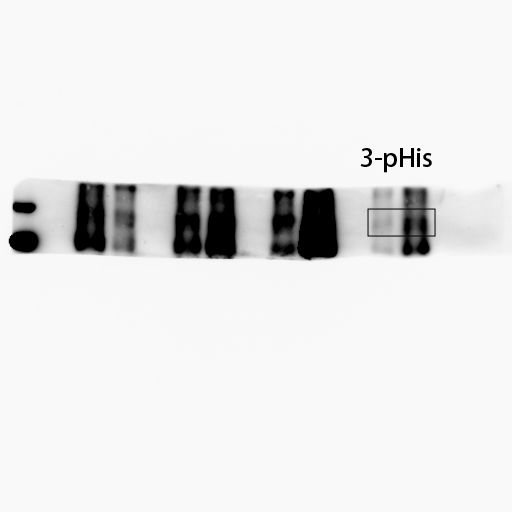

Supplement: Supplementary file 8 — Source data Fig. 6 [file 44318_2024_110_MOESM8_ESM.zip › Figure 6/6C/7-3-pHis.tif]

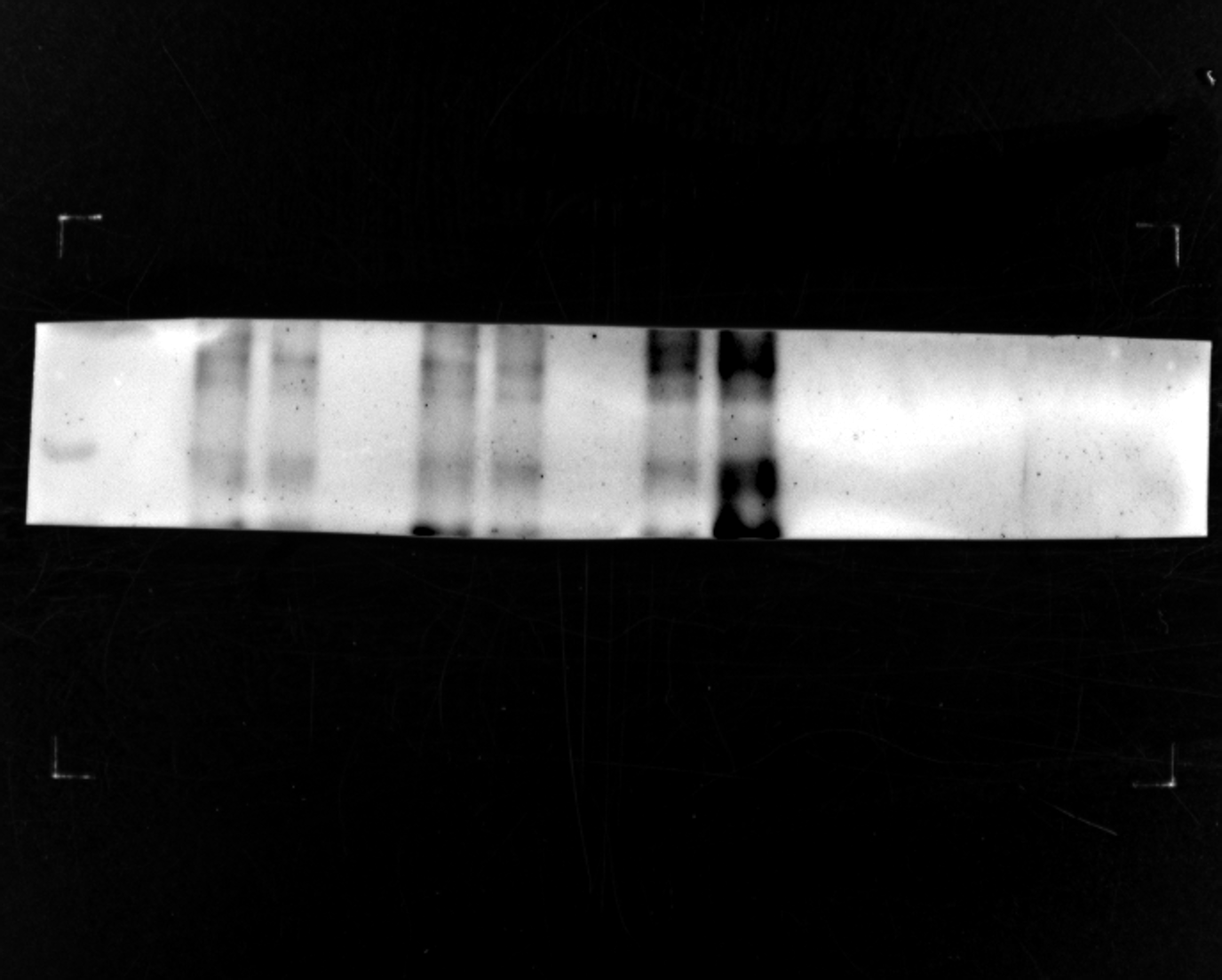

Supplement: Supplementary file 8 — Source data Fig. 6 [file 44318_2024_110_MOESM8_ESM.zip › Figure 6/6C/4.Tif]

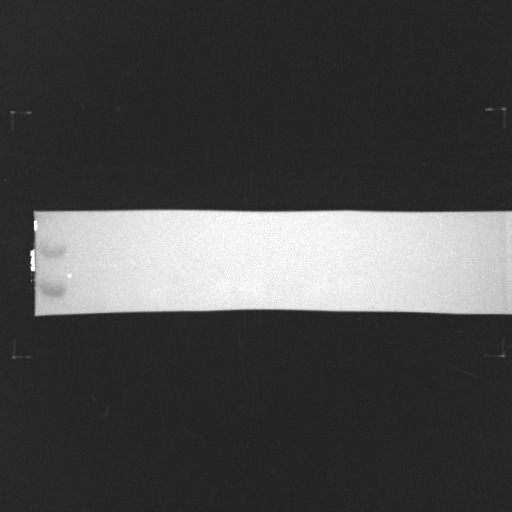

Supplement: Supplementary file 8 — Source data Fig. 6 [file 44318_2024_110_MOESM8_ESM.zip › Figure 6/6C/6 8 9.tif]

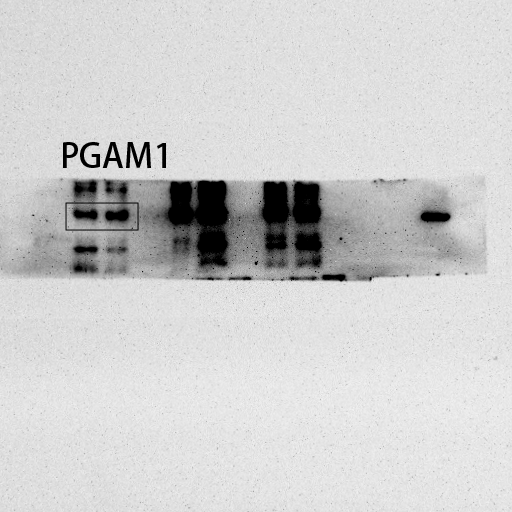

Supplement: Supplementary file 8 — Source data Fig. 6 [file 44318_2024_110_MOESM8_ESM.zip › Figure 6/6C/22-PGAM1.tif]

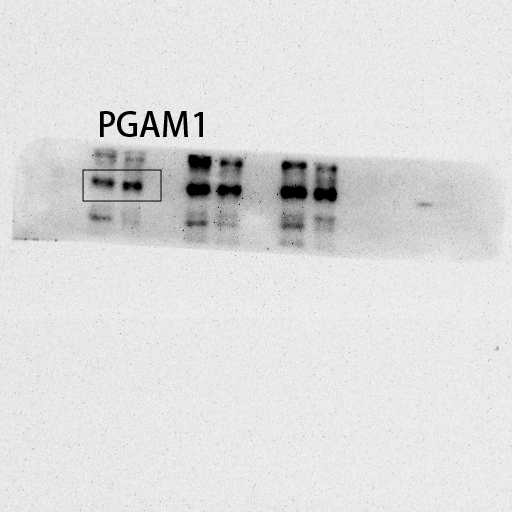

Supplement: Supplementary file 8 — Source data Fig. 6 [file 44318_2024_110_MOESM8_ESM.zip › Figure 6/6C/28-PGAM1.tif]

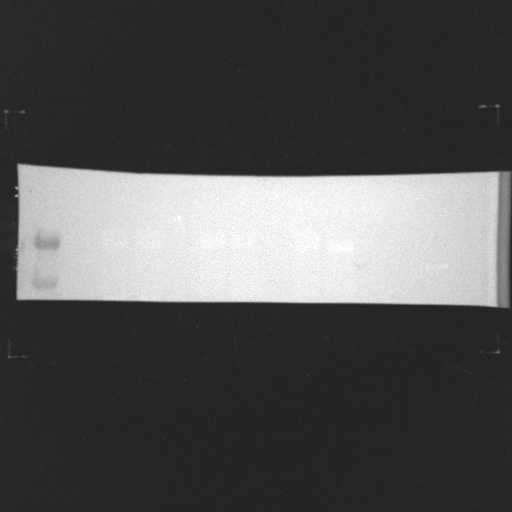

Supplement: Supplementary file 8 — Source data Fig. 6 [file 44318_2024_110_MOESM8_ESM.zip › Figure 6/6C/16 19.tif]

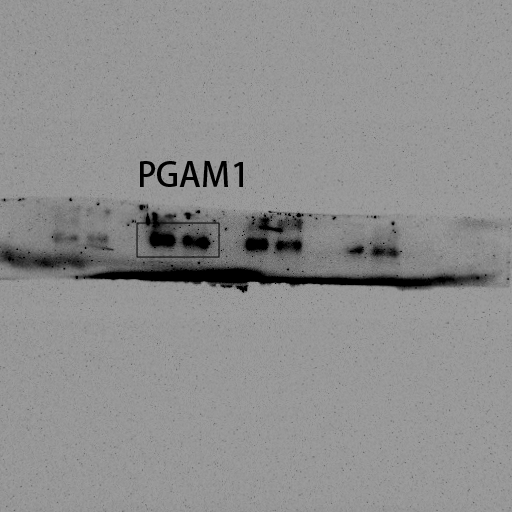

Supplement: Supplementary file 8 — Source data Fig. 6 [file 44318_2024_110_MOESM8_ESM.zip › Figure 6/6C/25-PGAM1.tif]

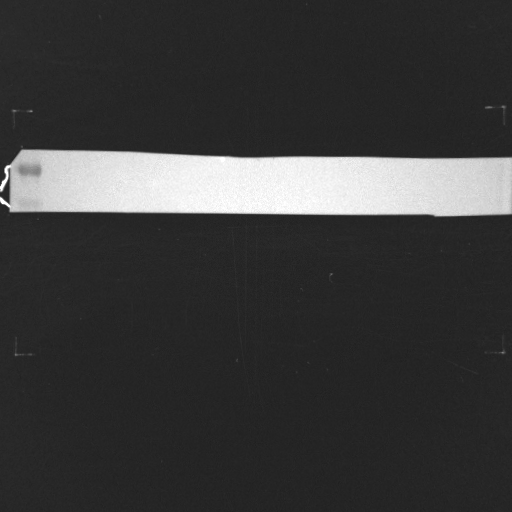

Supplement: Supplementary file 8 — Source data Fig. 6 [file 44318_2024_110_MOESM8_ESM.zip › Figure 6/6C/18.tif]

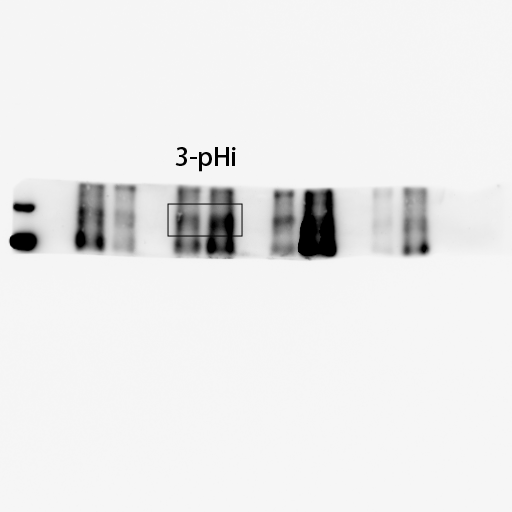

Supplement: Supplementary file 8 — Source data Fig. 6 [file 44318_2024_110_MOESM8_ESM.zip › Figure 6/6C/5-3-pHis.tif]

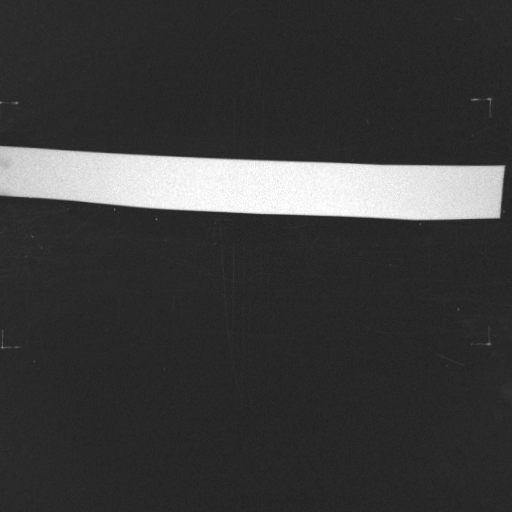

Supplement: Supplementary file 8 — Source data Fig. 6 [file 44318_2024_110_MOESM8_ESM.zip › Figure 6/6C/21.tif]

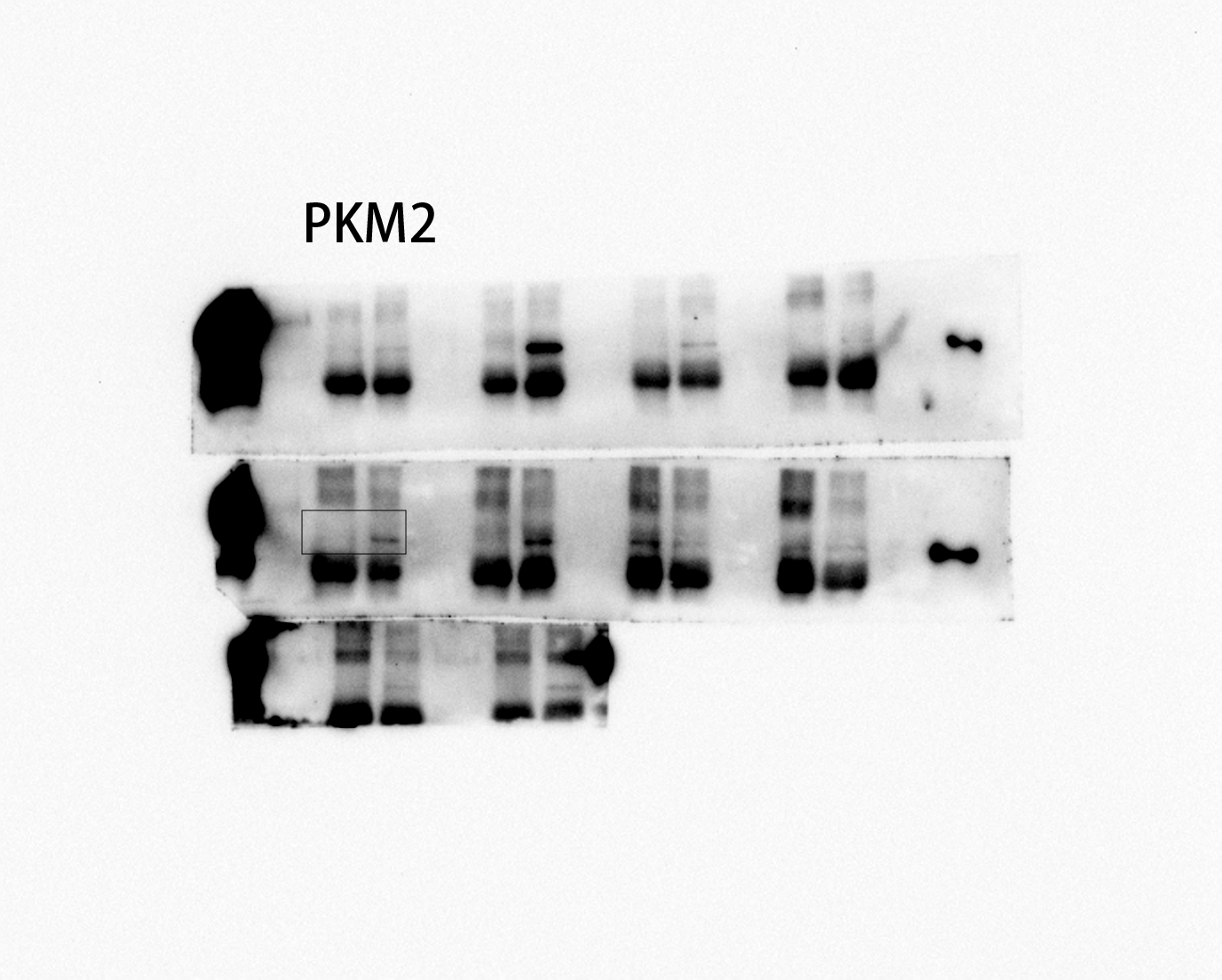

Supplement: Supplementary file 8 — Source data Fig. 6 [file 44318_2024_110_MOESM8_ESM.zip › Figure 6/6C/15-PKM2.Tif]

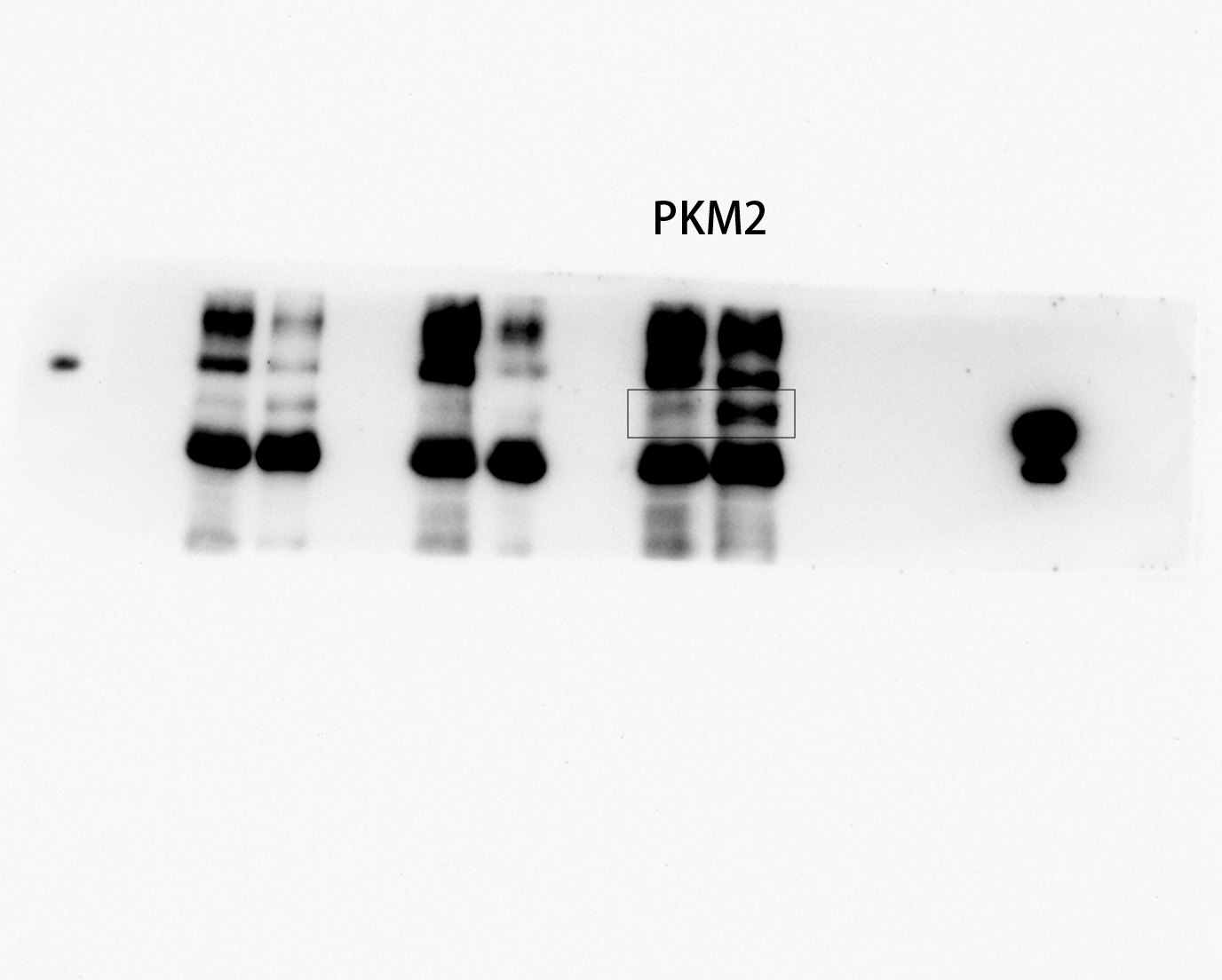

Supplement: Supplementary file 8 — Source data Fig. 6 [file 44318_2024_110_MOESM8_ESM.zip › Figure 6/6C/14-PKM2.Tif]

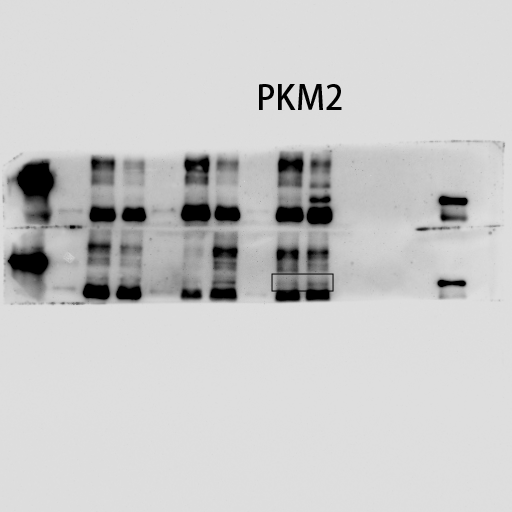

Supplement: Supplementary file 8 — Source data Fig. 6 [file 44318_2024_110_MOESM8_ESM.zip › Figure 6/6C/20-PKM2.tif]

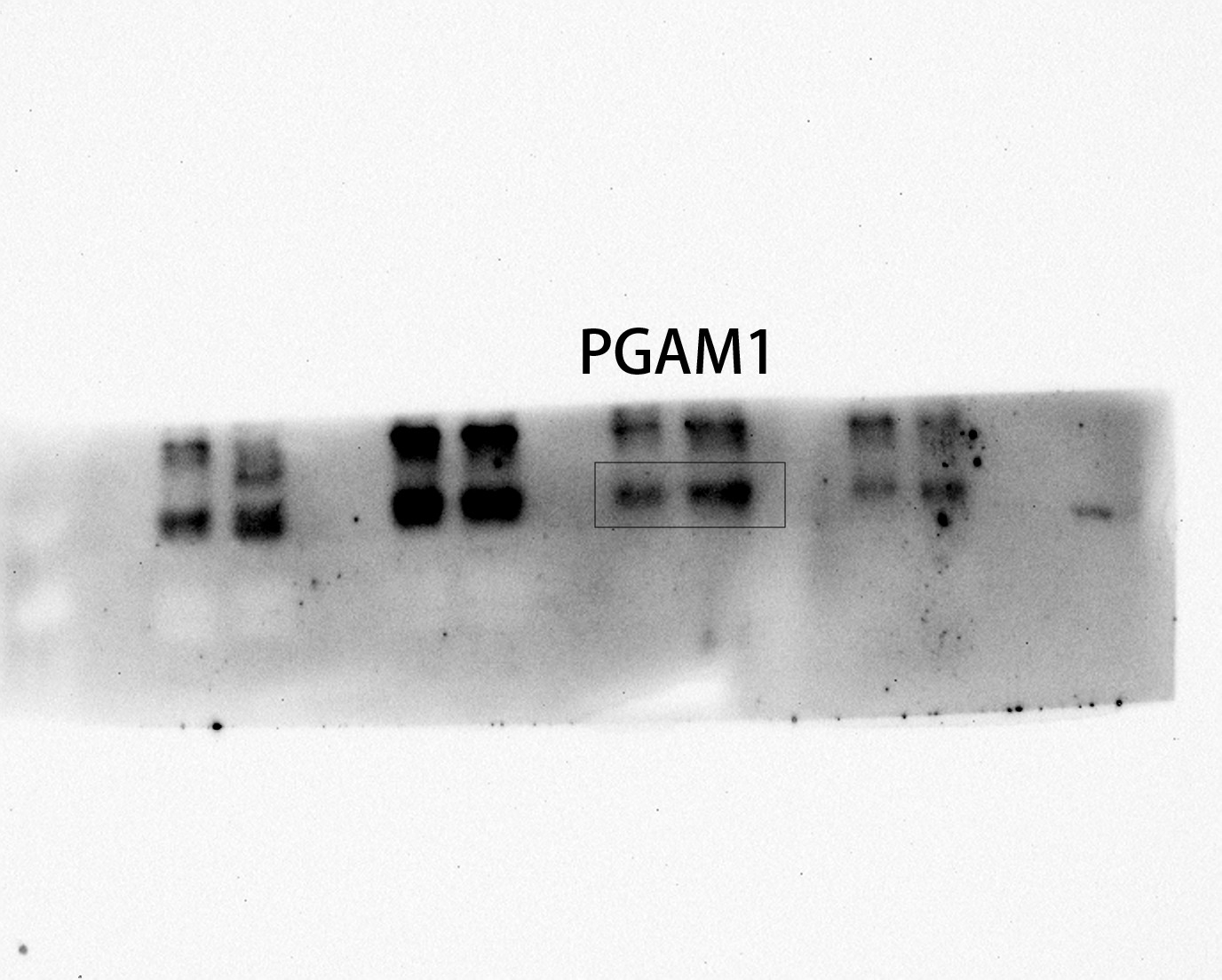

Supplement: Supplementary file 8 — Source data Fig. 6 [file 44318_2024_110_MOESM8_ESM.zip › Figure 6/6C/23-PGAM1.Tif]

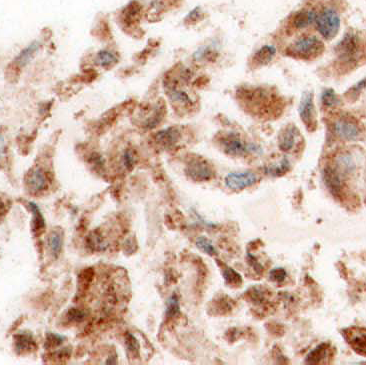

Supplement: Supplementary file 8 — Source data Fig. 6 [file 44318_2024_110_MOESM8_ESM.zip › Figure 6/6A-B/Patient 2 - T - Src.tif]

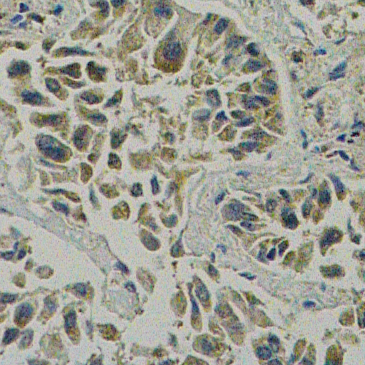

Supplement: Supplementary file 8 — Source data Fig. 6 [file 44318_2024_110_MOESM8_ESM.zip › Figure 6/6A-B/Patient 1 - T - PKM2.tif]

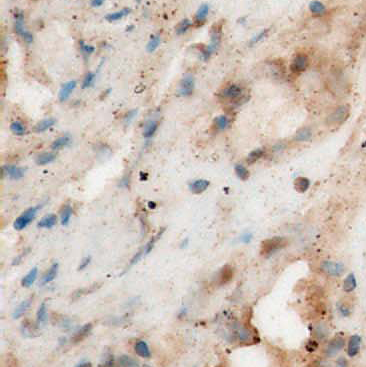

Supplement: Supplementary file 8 — Source data Fig. 6 [file 44318_2024_110_MOESM8_ESM.zip › Figure 6/6A-B/Patient 2 - A - Src.tif]

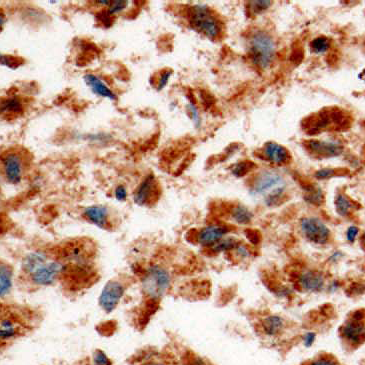

Supplement: Supplementary file 8 — Source data Fig. 6 [file 44318_2024_110_MOESM8_ESM.zip › Figure 6/6A-B/Patient 1 - T - Src.tif]

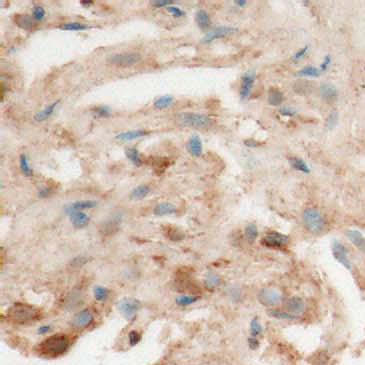

Supplement: Supplementary file 8 — Source data Fig. 6 [file 44318_2024_110_MOESM8_ESM.zip › Figure 6/6A-B/Patient 1 - A - Src.tif]

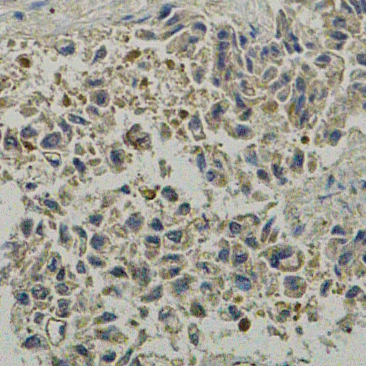

Supplement: Supplementary file 8 — Source data Fig. 6 [file 44318_2024_110_MOESM8_ESM.zip › Figure 6/6A-B/Patient 2 - T - PKM2.tif]

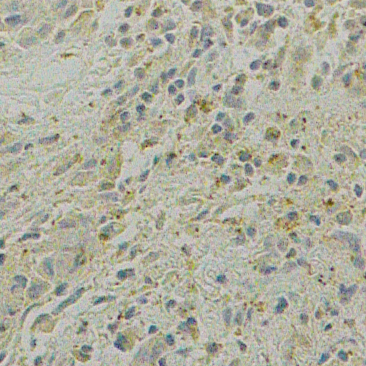

Supplement: Supplementary file 8 — Source data Fig. 6 [file 44318_2024_110_MOESM8_ESM.zip › Figure 6/6A-B/Patient 3 - T - PGAM1 pY119.tif]

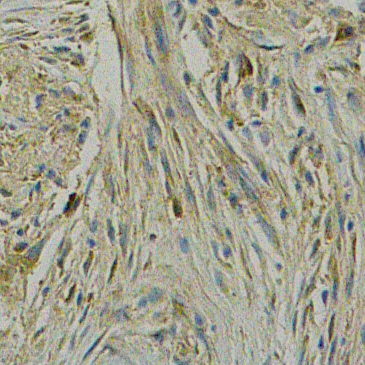

Supplement: Supplementary file 8 — Source data Fig. 6 [file 44318_2024_110_MOESM8_ESM.zip › Figure 6/6A-B/Patient 3 - T - PKM2.tif]

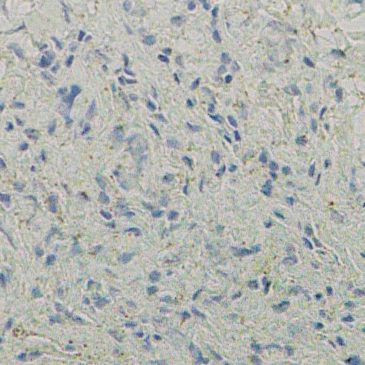

Supplement: Supplementary file 8 — Source data Fig. 6 [file 44318_2024_110_MOESM8_ESM.zip › Figure 6/6A-B/Patient 2 - A - PGAM1 pY119.tif]

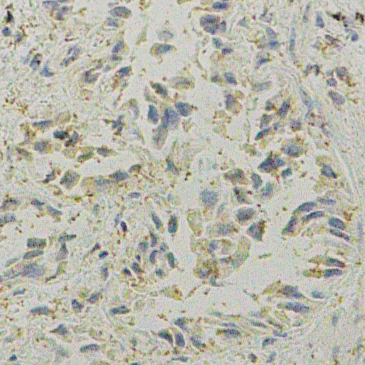

Supplement: Supplementary file 8 — Source data Fig. 6 [file 44318_2024_110_MOESM8_ESM.zip › Figure 6/6A-B/Patient 1 - T - PGAM1 pY119.tif]

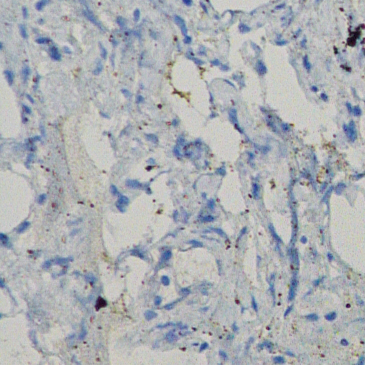

Supplement: Supplementary file 8 — Source data Fig. 6 [file 44318_2024_110_MOESM8_ESM.zip › Figure 6/6A-B/Patient 2 - A - PKM2.tif]

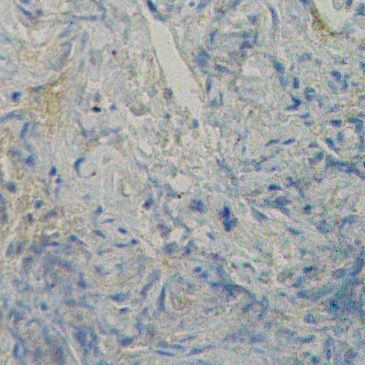

Supplement: Supplementary file 8 — Source data Fig. 6 [file 44318_2024_110_MOESM8_ESM.zip › Figure 6/6A-B/Patient 3 - A - PKM2.tif]

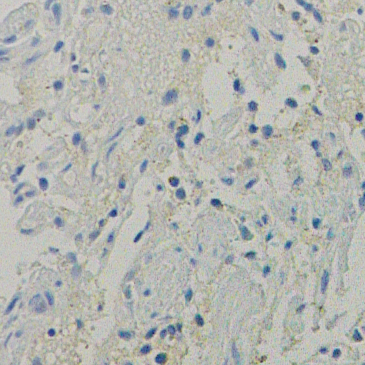

Supplement: Supplementary file 8 — Source data Fig. 6 [file 44318_2024_110_MOESM8_ESM.zip › Figure 6/6A-B/Patient 1 - A - PKM2.tif]

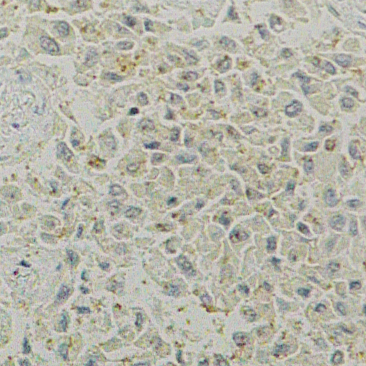

Supplement: Supplementary file 8 — Source data Fig. 6 [file 44318_2024_110_MOESM8_ESM.zip › Figure 6/6A-B/Patient 2 - T - PGAM1 pY119.tif]

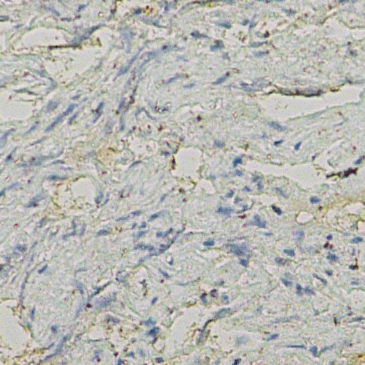

Supplement: Supplementary file 8 — Source data Fig. 6 [file 44318_2024_110_MOESM8_ESM.zip › Figure 6/6A-B/Patient 1 - A - PGAM1 pY119.tif]

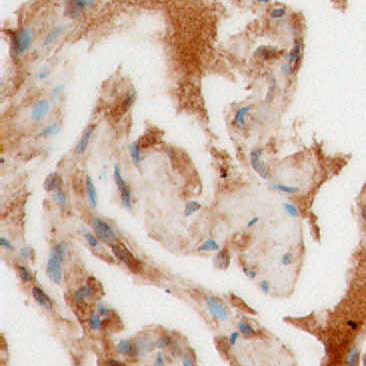

Supplement: Supplementary file 8 — Source data Fig. 6 [file 44318_2024_110_MOESM8_ESM.zip › Figure 6/6A-B/Patient 3 - A - Src.tif]

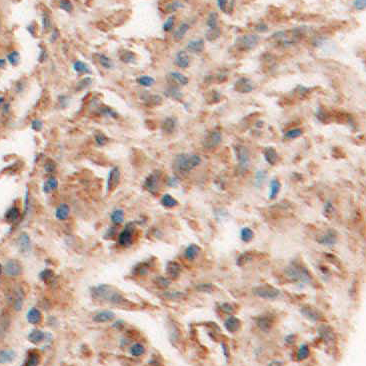

Supplement: Supplementary file 8 — Source data Fig. 6 [file 44318_2024_110_MOESM8_ESM.zip › Figure 6/6A-B/Patient 3 - T - Src.tif]

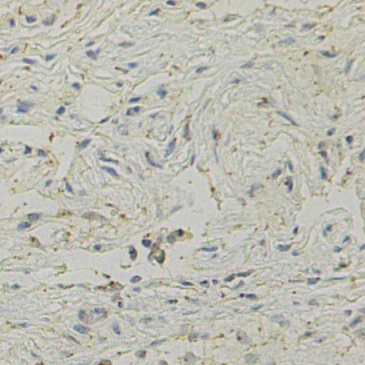

Supplement: Supplementary file 8 — Source data Fig. 6 [file 44318_2024_110_MOESM8_ESM.zip › Figure 6/6A-B/Patient 3 - A - PGAM1 pY119.tif]

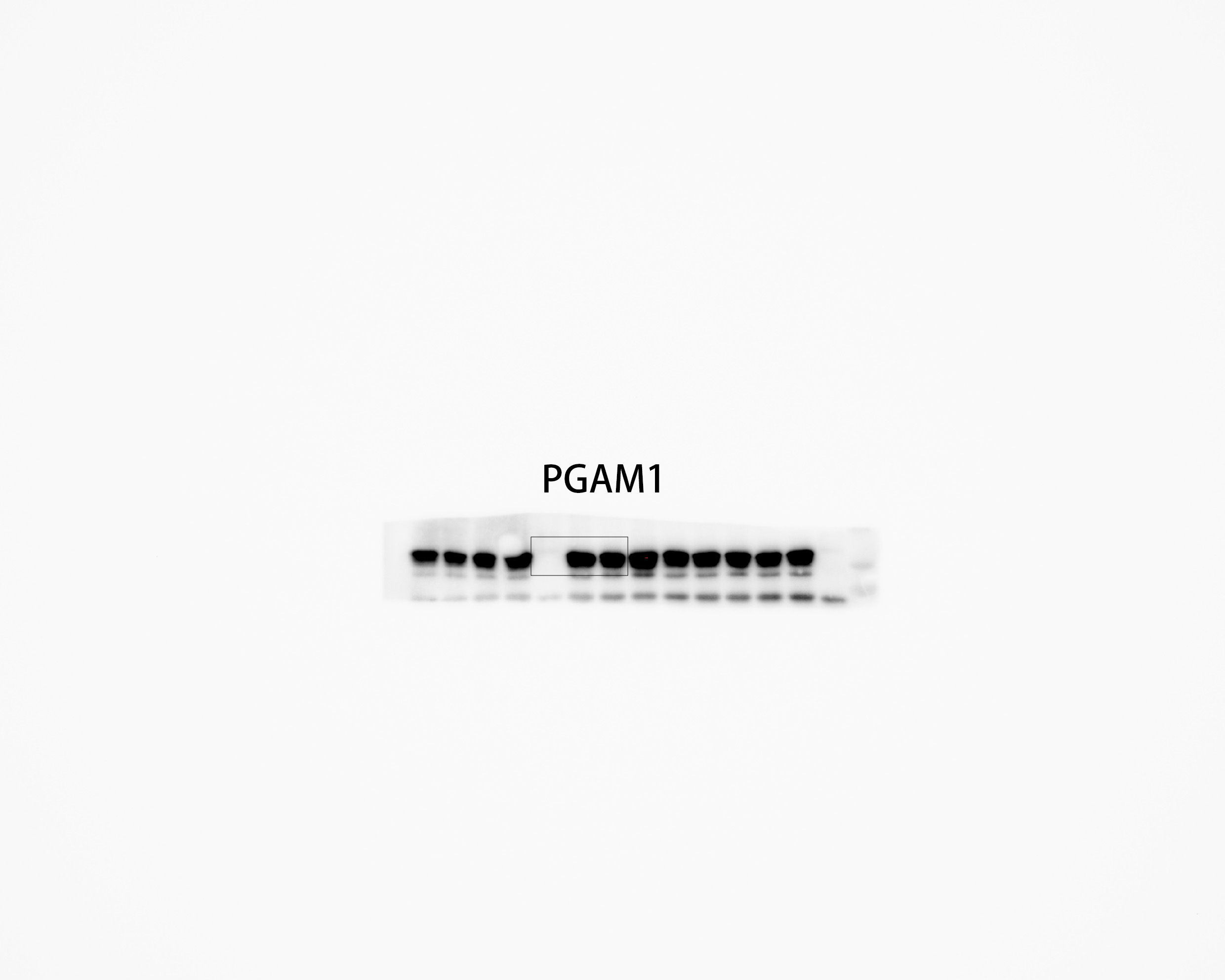

Supplement: Supplementary file 9 — Source data Fig. 7 [file 44318_2024_110_MOESM9_ESM.zip › Figure 7/7B/4-Flag.tif]
