# Supplementary figures and images for: PKM2 functions as a histidine kinase to phosphorylate PGAM1 and increase glycolysis shunts in cancer (part 3 of 3)
Source: EMBO J. 2024 May 15;43(12):5. doi: 10.1038/s44318-024-00110-8 (PMC11183095; doi:10.1038/s44318-024-00110-8)

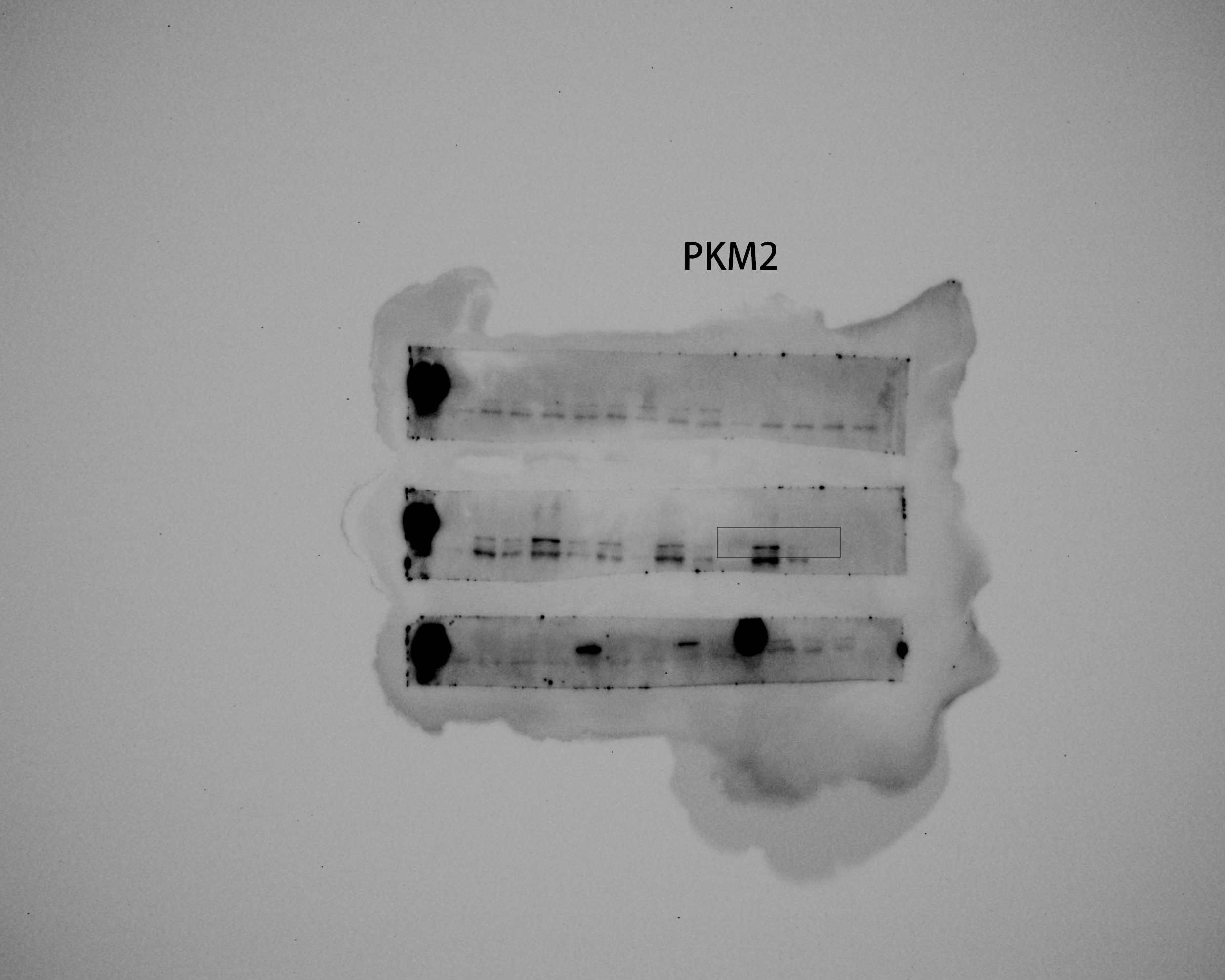

Supplement: Supplementary file 9 — Source data Fig. 7 [file 44318_2024_110_MOESM9_ESM.zip › Figure 7/7B/2-PKM2.tif]

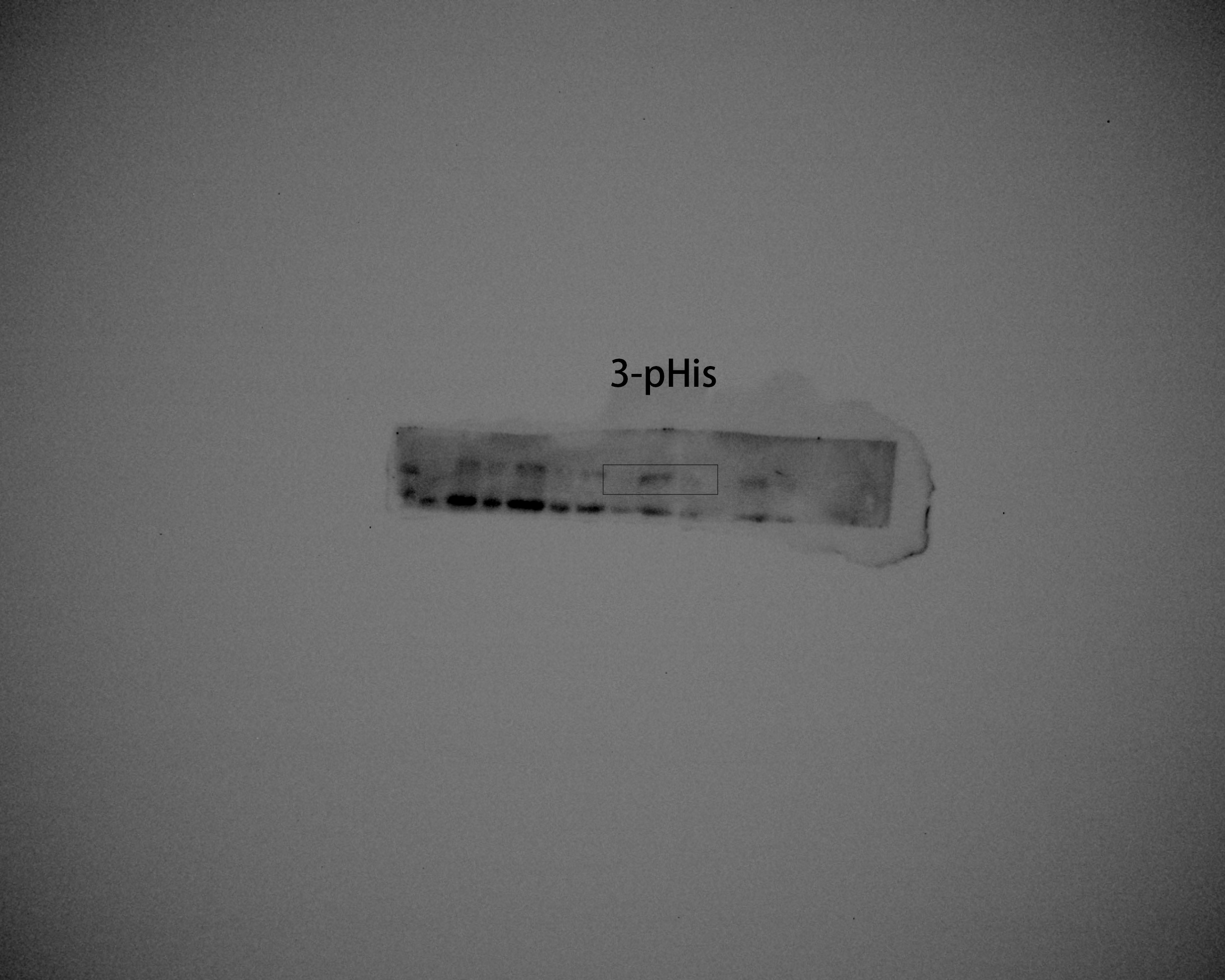

Supplement: Supplementary file 9 — Source data Fig. 7 [file 44318_2024_110_MOESM9_ESM.zip › Figure 7/7B/3-3-pHis.tif]

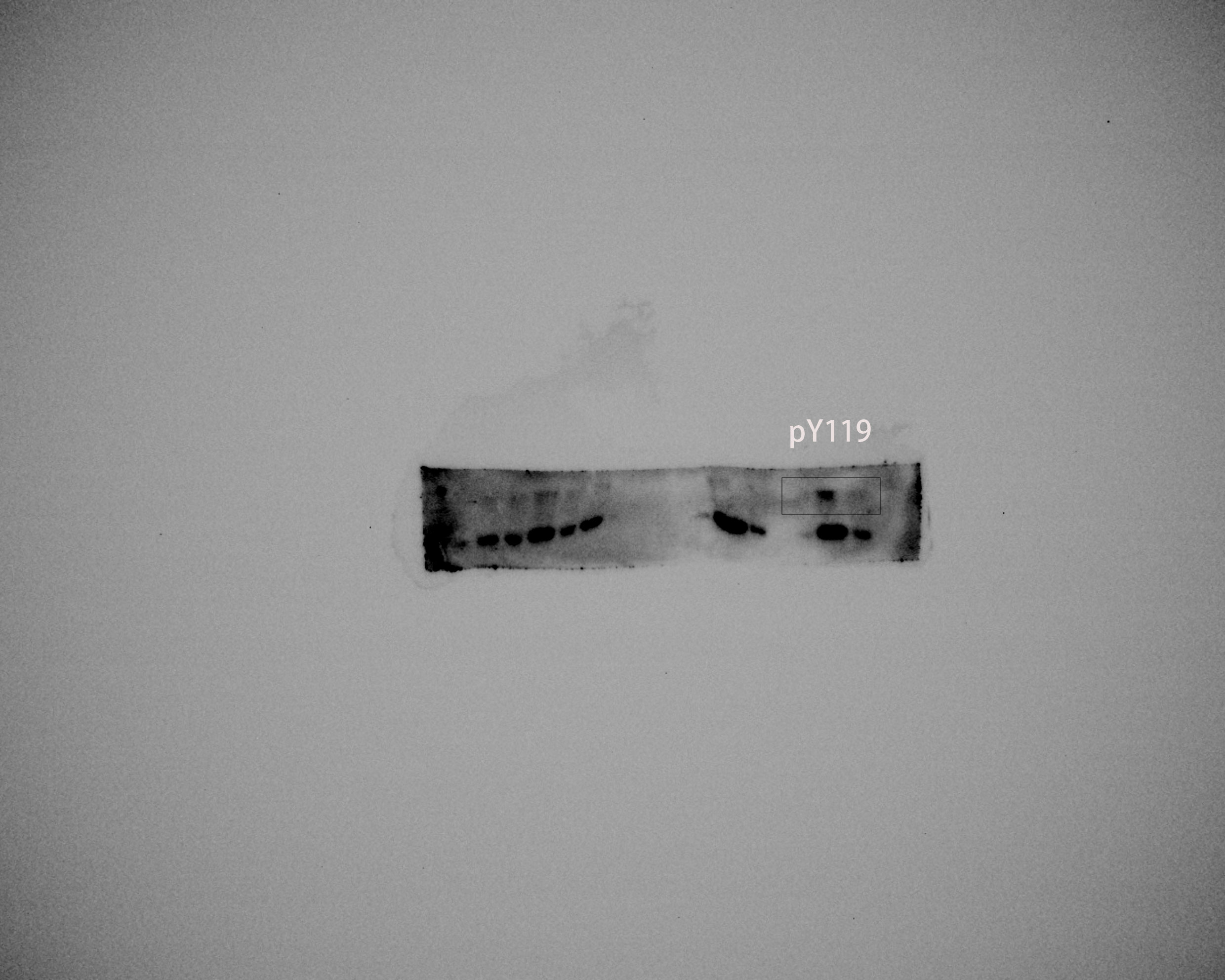

Supplement: Supplementary file 9 — Source data Fig. 7 [file 44318_2024_110_MOESM9_ESM.zip › Figure 7/7B/1-pY119.tif]

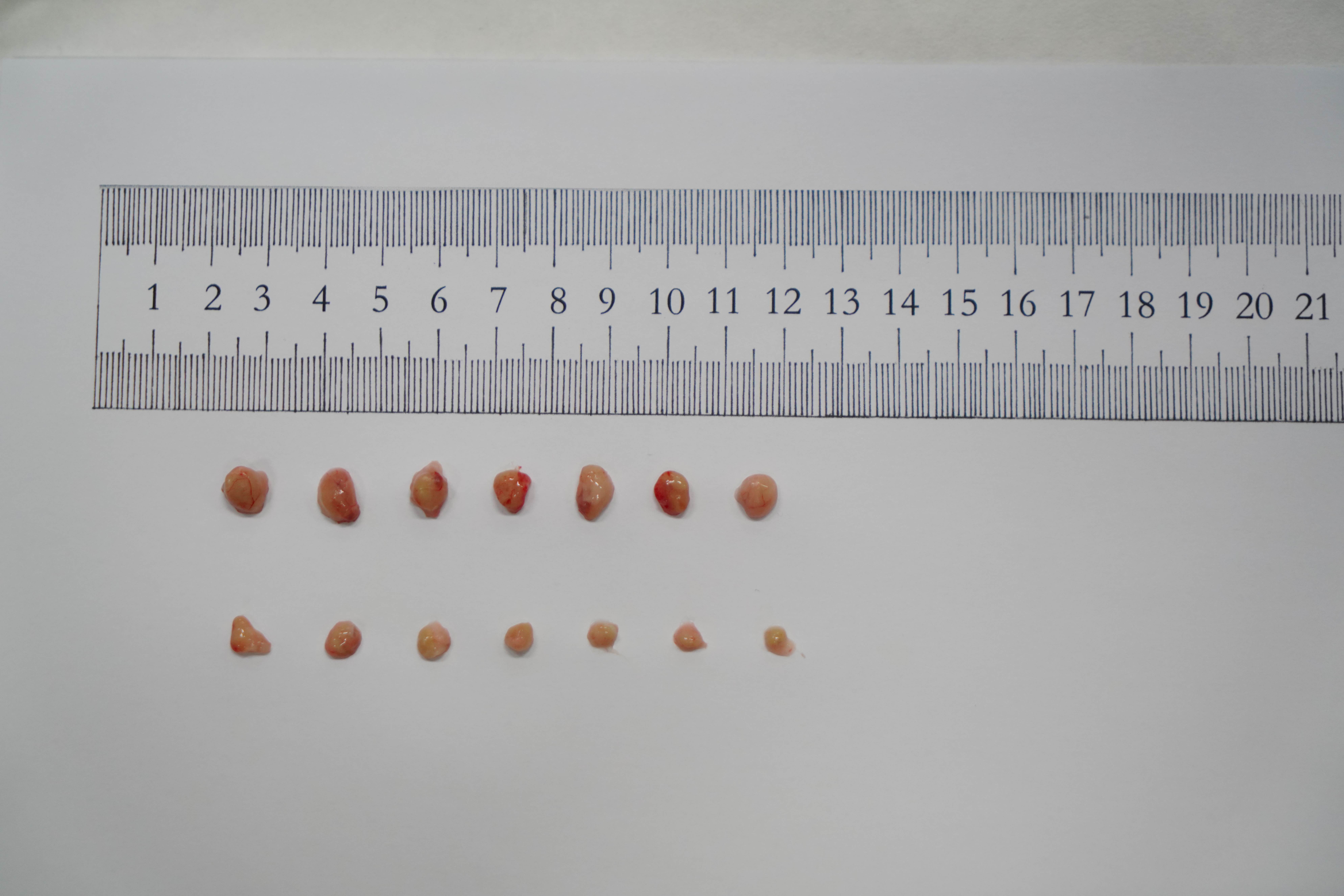

Supplement: Supplementary file 9 — Source data Fig. 7 [file 44318_2024_110_MOESM9_ESM.zip › Figure 7/7D-G/2-Tumors.JPG]

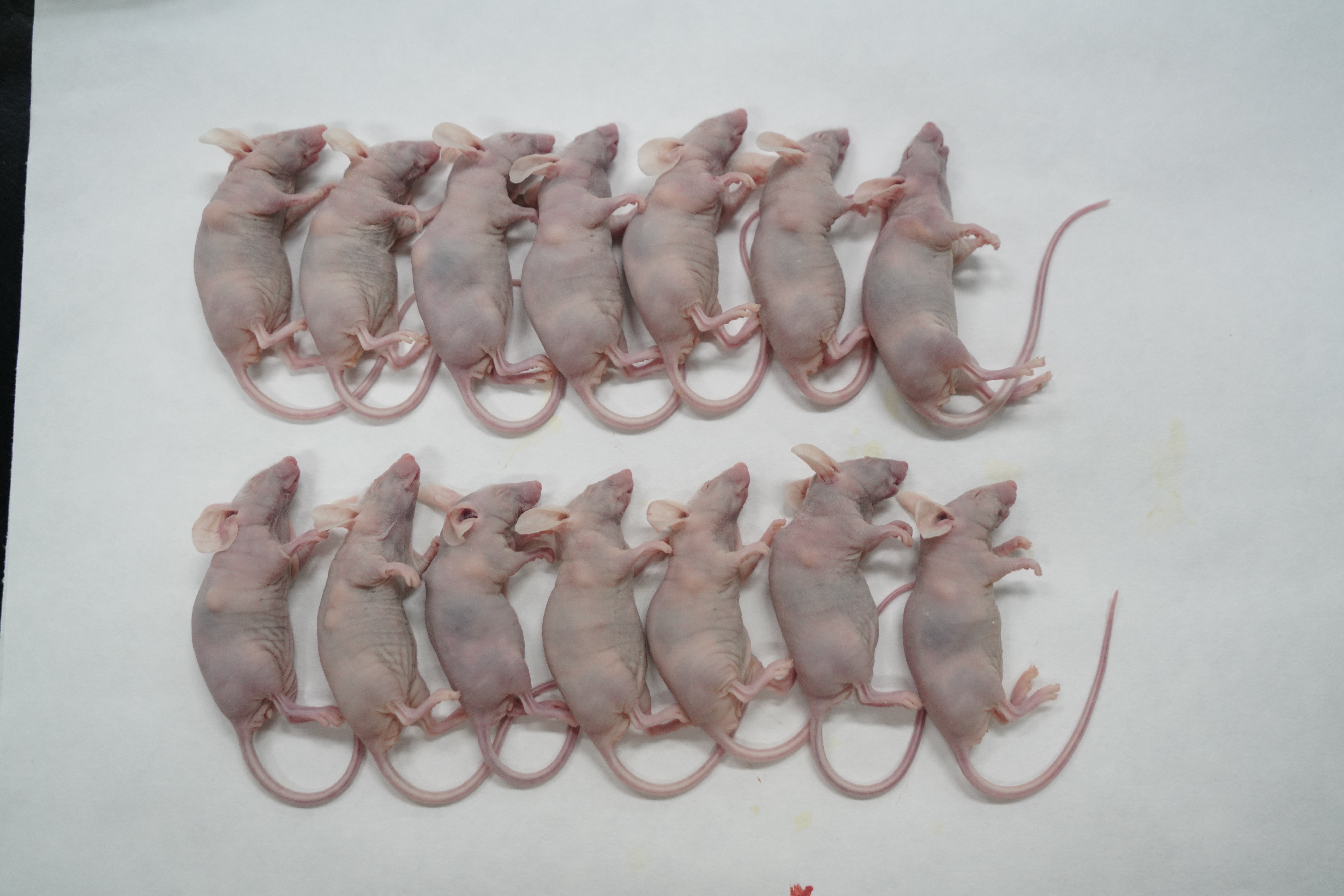

Supplement: Supplementary file 9 — Source data Fig. 7 [file 44318_2024_110_MOESM9_ESM.zip › Figure 7/7D-G/1-Mice xenograft tumors.JPG]

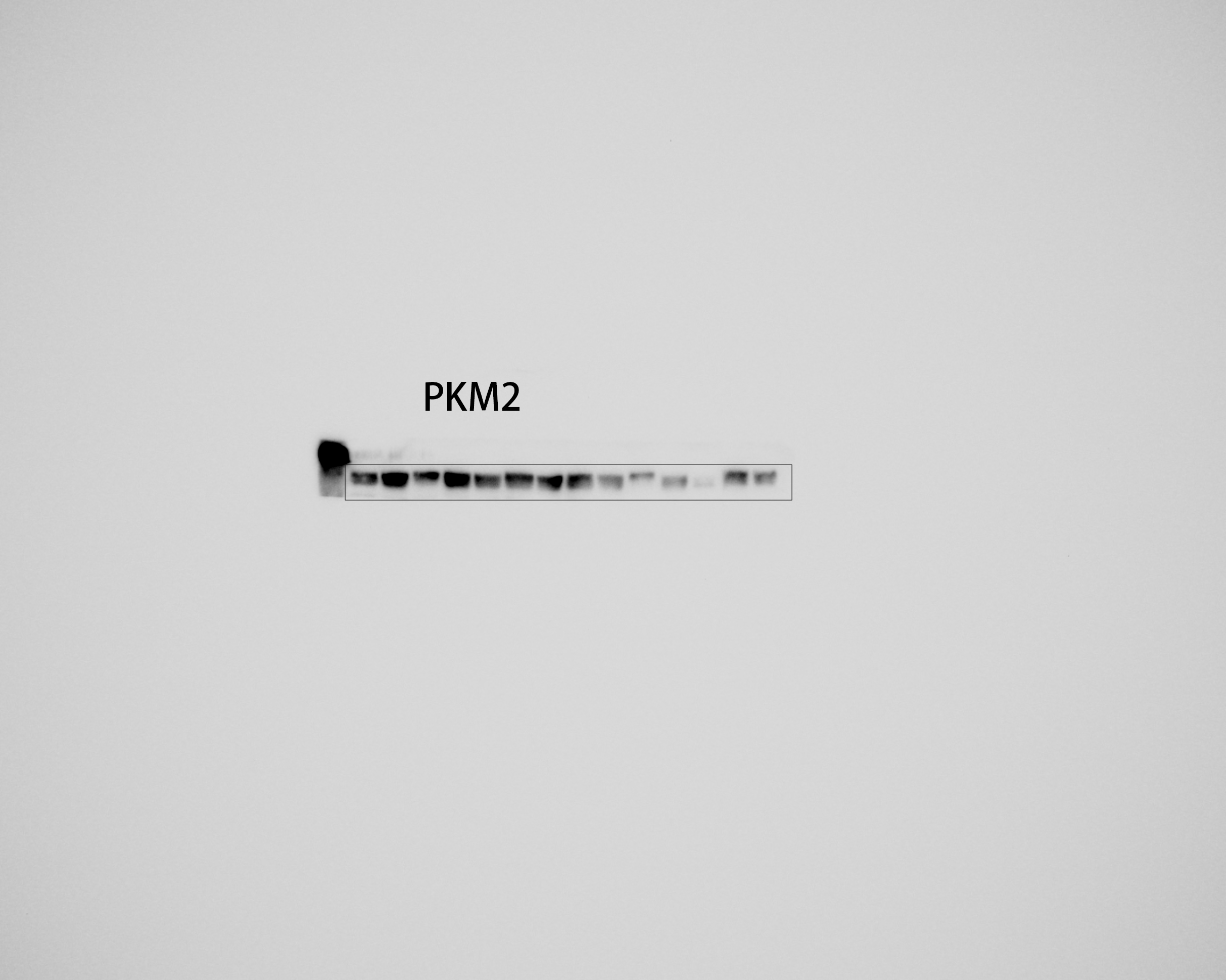

Supplement: Supplementary file 9 — Source data Fig. 7 [file 44318_2024_110_MOESM9_ESM.zip › Figure 7/7H/2-PKM2.tif]

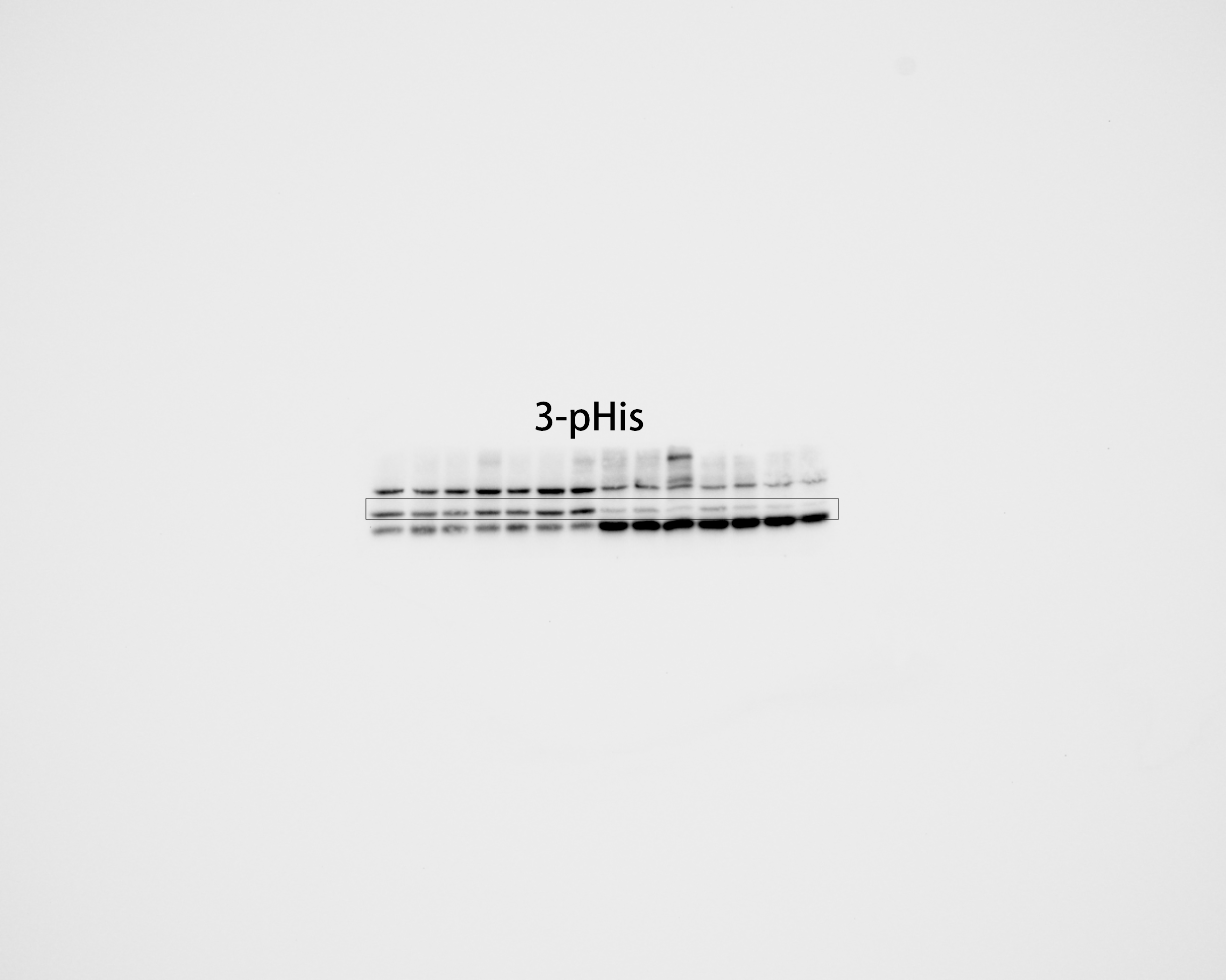

Supplement: Supplementary file 9 — Source data Fig. 7 [file 44318_2024_110_MOESM9_ESM.zip › Figure 7/7H/3-3-pHis.tif]

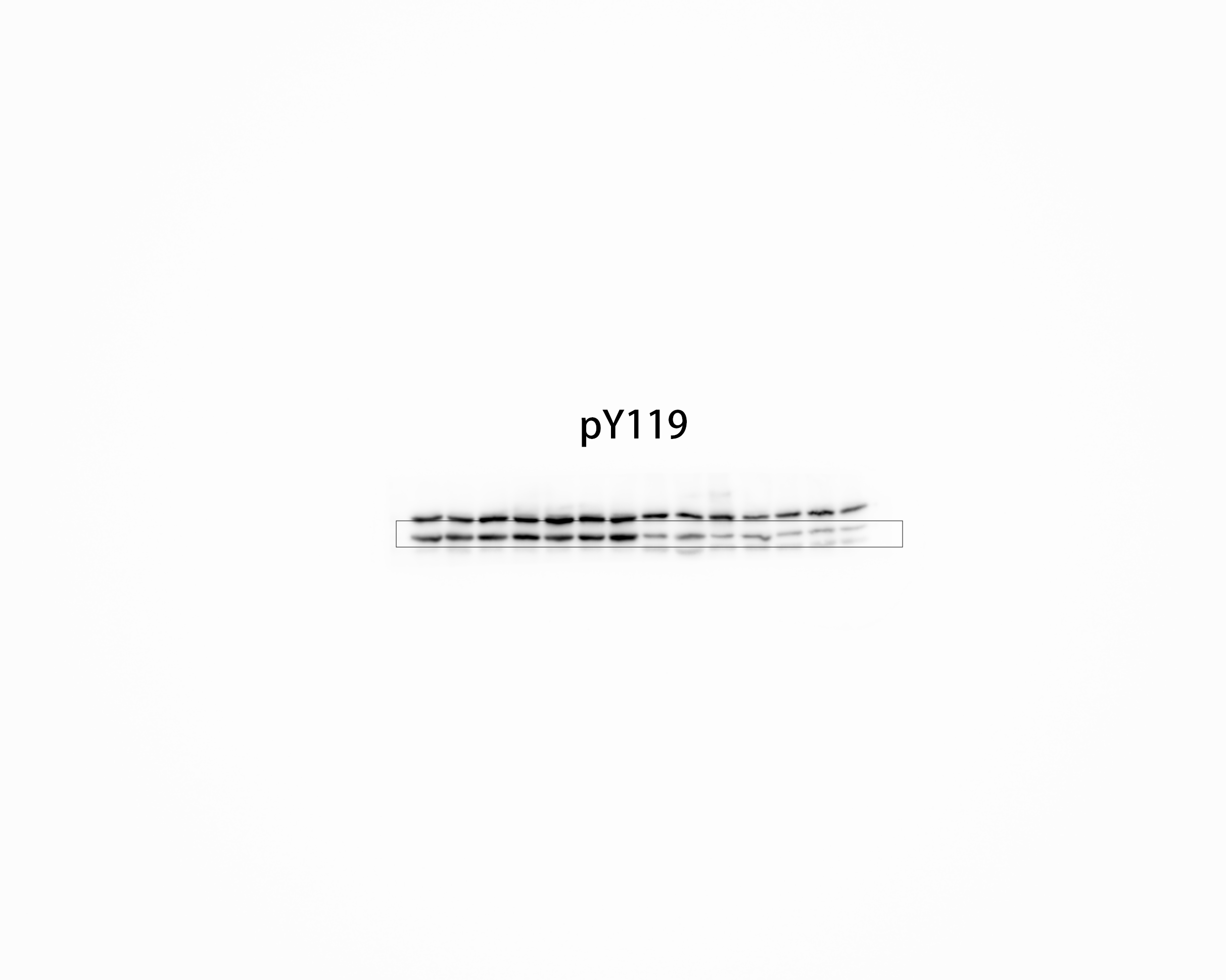

Supplement: Supplementary file 9 — Source data Fig. 7 [file 44318_2024_110_MOESM9_ESM.zip › Figure 7/7H/1-pY119.tif]

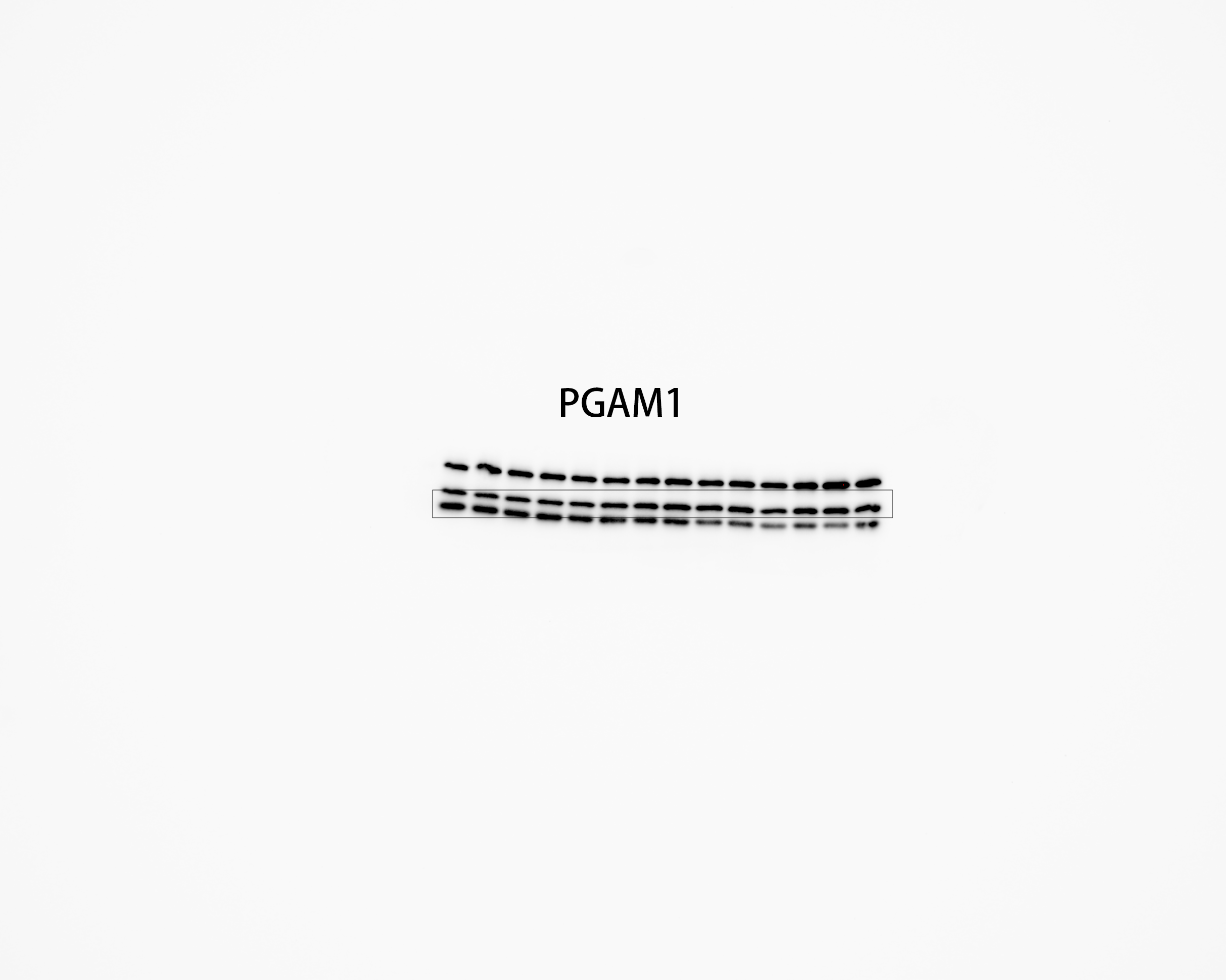

Supplement: Supplementary file 9 — Source data Fig. 7 [file 44318_2024_110_MOESM9_ESM.zip › Figure 7/7H/4-PGAM1.tif]
