# Supplementary material for: PKM2 functions as a histidine kinase to phosphorylate PGAM1 and increase glycolysis shunts in cancer
Source: EMBO J. 2024 May 15;43(12):5. doi: 10.1038/s44318-024-00110-8 (PMC11183095; doi:10.1038/s44318-024-00110-8)
Supplement: Supplementary file 11 — Expanded View Figures [file 44318_2024_110_MOESM11_ESM.pdf]

Expanded View Figures

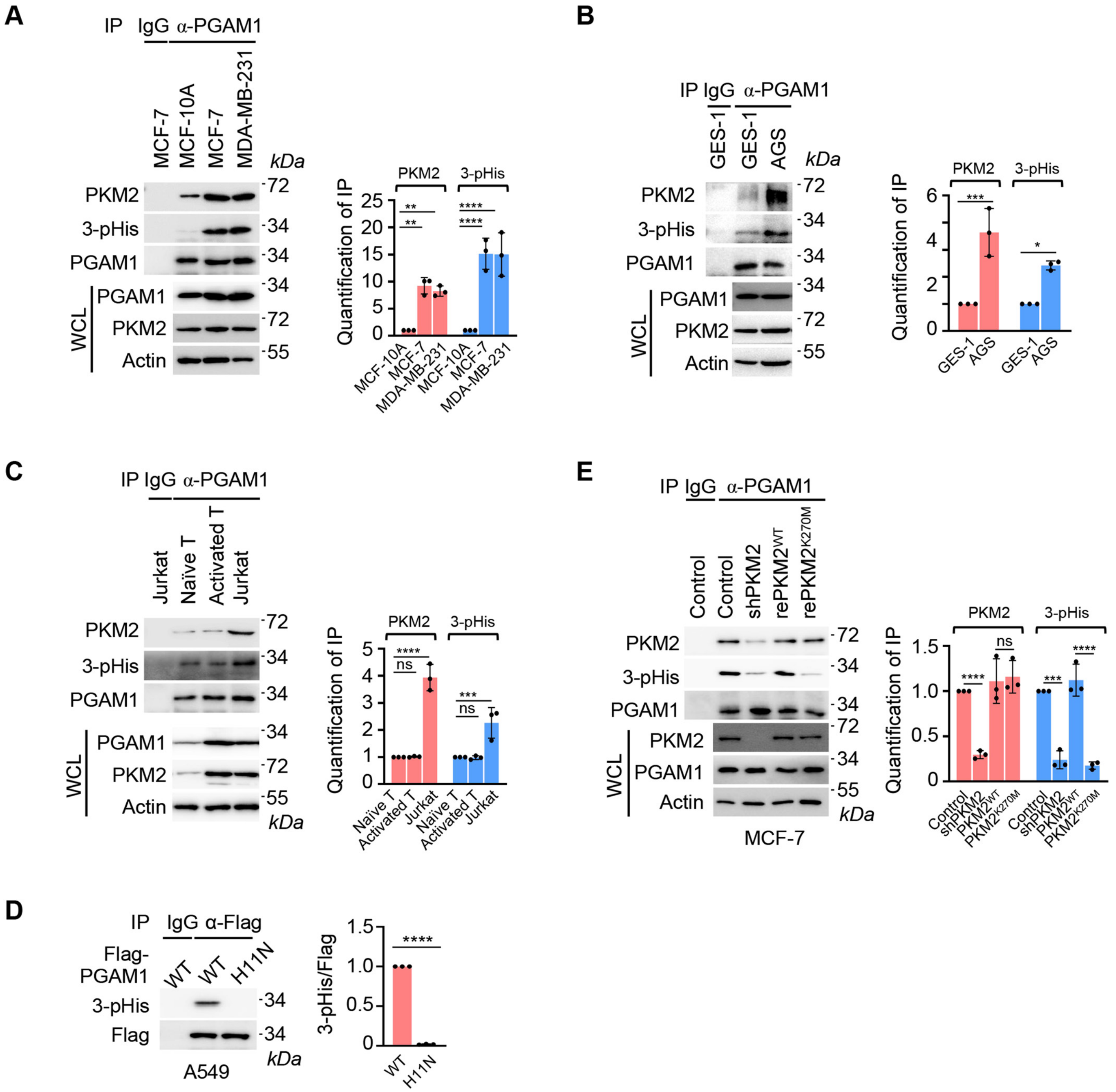

Figure EV1. PKM2 upregulates PGAM H11 phosphorylation.

(**A–C**) PGAM1–PKM2 interaction and PGAM1 phosphorylation are more evident in tumor cells. Left: PGAM1-associated proteins were immunoprecipitated from the whole-cell lysates of different tumor cells and analyzed by WB. Right: Quantification of IP. Relative levels of PKM2 or 3-pHis were normalized to that of PGAM1 for each group. (**D**) PGAM1 H11N mutation blocks 3-pHis modification. A549 cells were transfected with Flag- PGAM1 WT or H11N mutation for 48 h. Left: Flag (PGAM1)-associated proteins were immunoprecipitated and analyzed by WB. Right: Quantification of IP. Relative levels of 3-pHis were normalized to that of Flag (PGAM1) for each group. (**E**) PKM2 K270M mutation reduces PGAM1 H11 phosphorylation. PKM2-depleted MCF-7 cells were re-expressed PKM2 WT or PKM2 K270M mutation. Left: PGAM1-associated proteins were immunoprecipitated and analyzed by WB. Right: Quantification of IP. Relative levels of PKM2 or 3-pHis were normalized to that of PGAM1 for each group. Data information: for WB in (**A–E**), one representative experiment out of three was shown. IgG served as a negative control. Data represent mean ± SD of three (**A–E**) independent experiments, with significance determined by one-way ANOVA test (**A–C**, **E**) or Student's *t* test (**D**); ****$P < 0.0001$, ***$P < 0.001$, **$P < 0.01$, *$P < 0.05$, ns, nonsignificant. Source data are available online for this figure.

 © The Author(s)

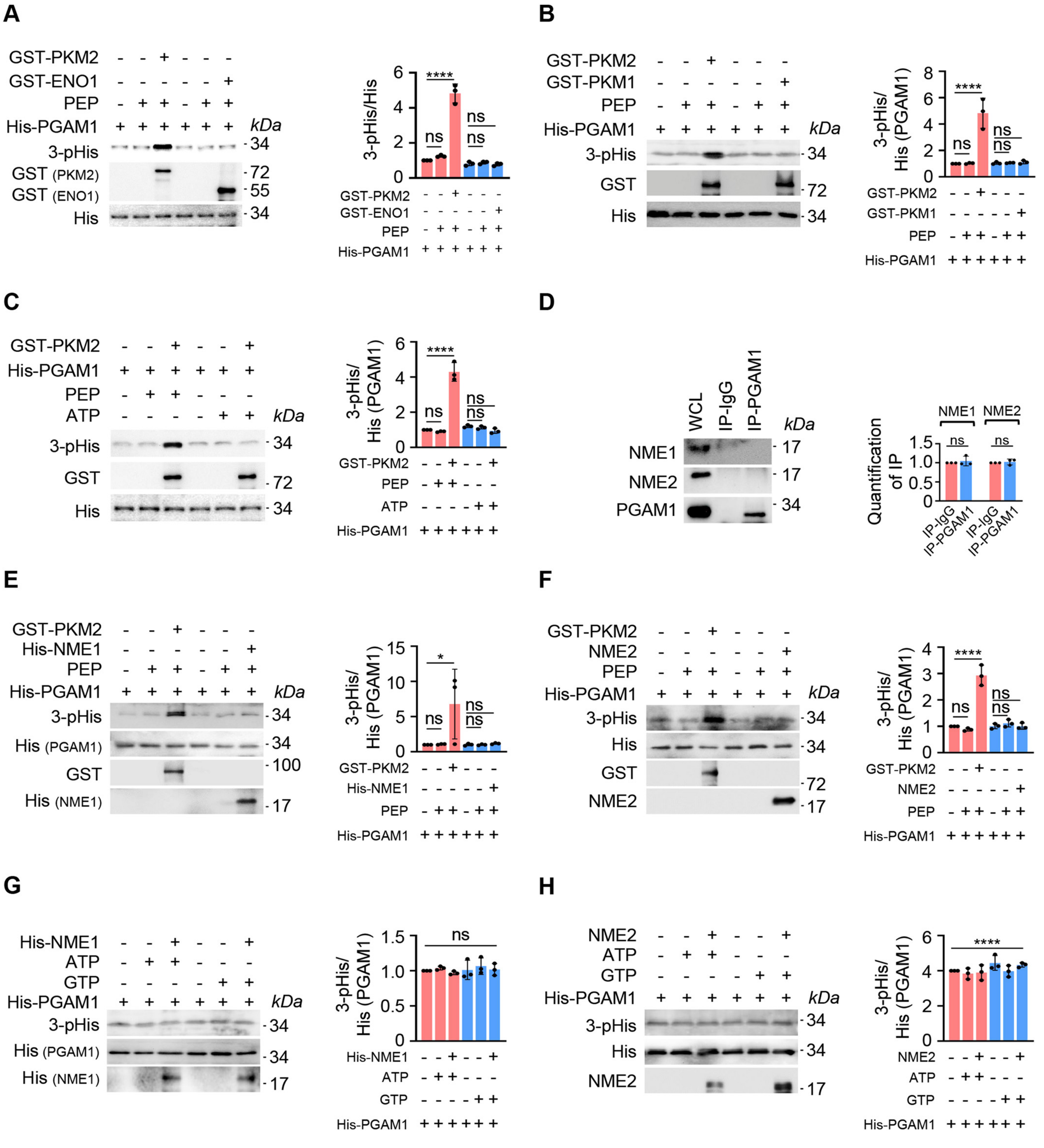
A
GST-PKM2 - - + - - -
GST-ENO1 - - - - - +
PEP - + + - + +
His-PGAM1 + + + + + +
kDa
3-pHis
GST (PKM2)
GST (ENO1)
His
34
72
55
34
3-pHis/His
****
ns
ns
ns
B
GST-PKM2 - - + - - -
GST-PKM1 - - - - - +
PEP - + + - + +
His-PGAM1 + + + + + +
3-pHis
GST
His
3-pHis/ His (PGAM1)
C
GST-PKM2 - - + - - +
His-PGAM1 + + + + + +
PEP - + + - - -
ATP - - - - + +
3-pHis
GST
His
D
WCL
IP-IgG
IP-PGAM1
NME1
NME2
PGAM1
17
17
34
NME1
NME2
Quantification of IP
IP-IgG
IP-PGAM1
E
GST-PKM2 - - + - - -
His-NME1 - - - - - +
PEP - + + - + +
His-PGAM1 + + + + + +
3-pHis
His (PGAM1)
GST
His (NME1)
100
17
*
F
GST-PKM2 - - + - - -
NME2 - - - - - +
PEP - + + - + +
His-PGAM1 + + + + + +
3-pHis
His
GST
NME2
G
His-NME1 - - + - - +
ATP - + + - - -
GTP - - - - + +
His-PGAM1 + + + + + +
3-pHis
His (PGAM1)
His (NME1)
H
NME2 - - + - - +
ATP - + + - - -
GTP - - - - + +
His-PGAM1 + + + + + +
3-pHis
His
NME2

Figure EV2. ENO1, PKM1, ATP, NME1 or NME2 does not impact PGAM1 H11 phosphorylation.

(**A–C**) ENO1, PKM1 or ATP does not affect PGAM1 H11 phosphorylation. Left: In vitro kinase assay was carried out with recombinant GST-ENO1, GST-PKM2, GST-PKM1 and His-PGAM1 in the presence of PEP or ATP. PGAM1 H11 phosphorylation was detected by WB. Right: Quantification of WB. Relative levels of 3-pHis were normalized to that of His (PGAM1) for each group. (**D**) PGAM1 does not interact with NME1 or NME2. Left: PGAM1-associated proteins in A549 cells were immunoprecipitated and analyzed by WB. IgG served as a negative control. Right: Quantification of IP. Relative levels of NME1 or NME2 in IP were normalized to that in WCL for each group. (**E–H**) NME1 or NME2 does not affect PGAM1 H11 phosphorylation. Left: In vitro kinase assay was carried out with recombinant GST-PKM2, His-NME1, NME2 and His-PGAM1 in the presence of PEP, ATP or GTP. PGAM1 H11 phosphorylation was detected by WB. Right: Quantification of WB. Relative levels of 3-pHis were normalized to that of His (PGAM1) for each group. Data information: for WB in (**A–H**), one representative experiment out of three was shown. Data were represented as mean ± SD of three independent experiments (**A–H**) with significance determined by one-way ANOVA test (**A–C**, **E–H**) and Student's *t* test (**D**); ****$P < 0.0001$, *$P < 0.05$, ns nonsignificant. Source data are available online for this figure.

© The Author(s)

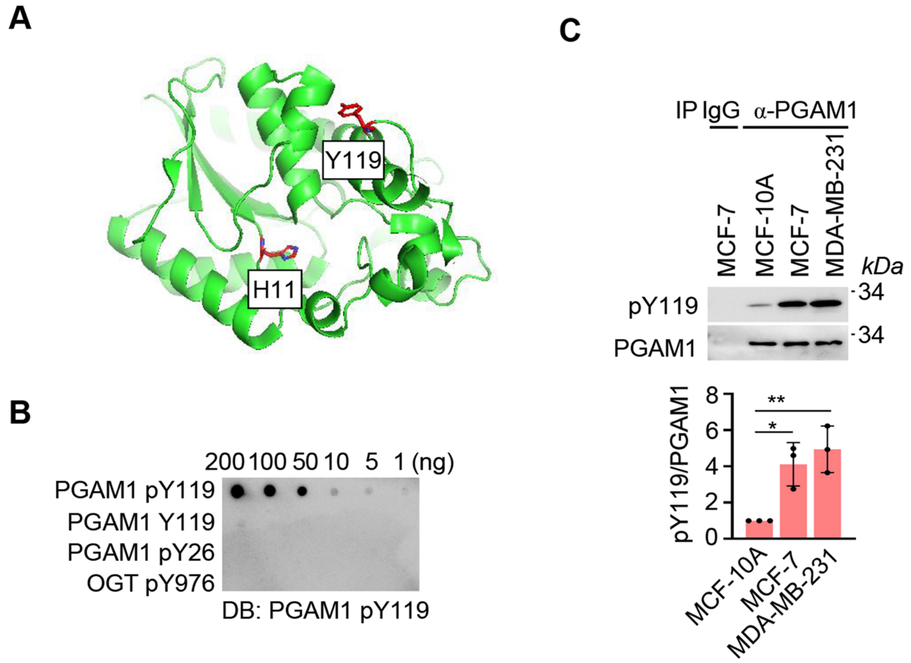

Figure EV3. PGAM1 Y119 molecular location and Y119 phosphorylation analysis in different cells.

(**A**) Molecular location of Y119 and H11 in PGAM1. A monomer of PGAM1 from protein data bank (PDB, ID: 4GPI) was shown in green cartoon. Y119 and H11 is shown in red sticks. (**B**) Confirmation of PGAM1 pY119 antibody by dot blot (DB). Titrated amounts of peptides, including phosphorylated PGAM1 Y119, unphosphorylated PGAM1 Y119, phosphorylated PGAM1 Y26 and phosphorylated OGT Y976, were spotted on nitrocellulose membrane and then probed with PGAM1 pY119 antibody. (**C**) PGAM1 Y119 phosphorylation is more evident in tumor cells. Top: In MCF-10A, MCF-7 and MDA-MB-231 cell lines, PGAM1-associated proteins were immunoprecipitated and analyzed by WB. One representative experiment out of three was shown. IgG served as a negative control. Bottom: Quantification of IP. Relative levels of pY119 were normalized to that of PGAM1 for each group. Data represents mean ± SD ($n = 3$) with significance determined by one-way ANOVA test; $^{**}P < 0.01$, $^{*}P < 0.05$. Source data are available online for this figure.

© The Author(s)

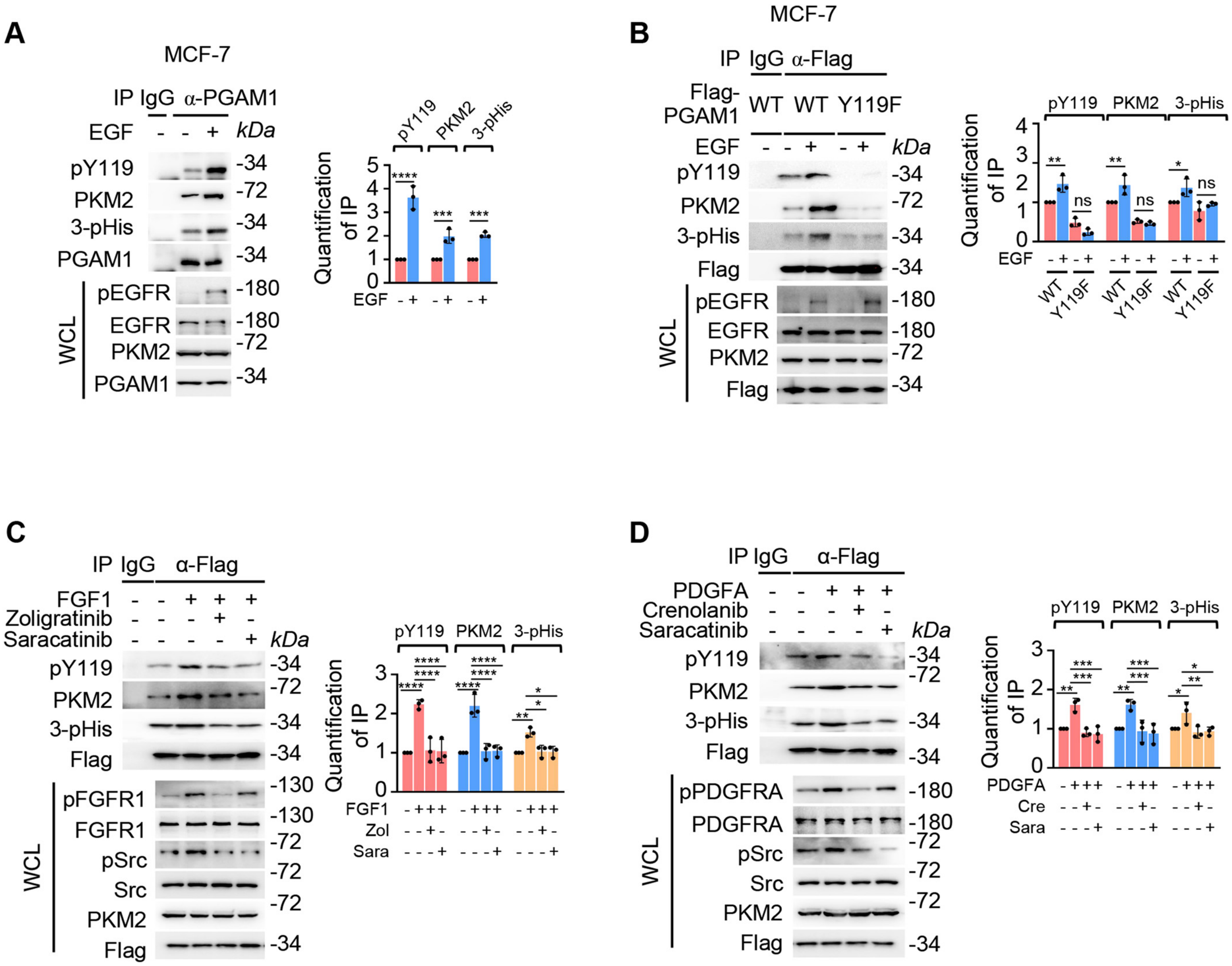

Figure EV4. Growth factors' signaling regulates PGAM1 Y119 phosphorylation.

(**A**, **B**) EGF triggers PGAM1 Y119 phosphorylation. Left: MCF-7 cells, or MCF-7 cells transfected with Flag-PGAM1 plasmids were cultured in the serum-free medium for 48 h, and then treated with or without EGF (100 ng/ml) for 30 min. Flag (PGAM1)-associated proteins were immunoprecipitated and analyzed by WB. Right: Quantification of IP. Relative levels of pY119, PKM2 or 3-pHis were normalized to that of endogenous or exogenous PGAM1 for each group. (**C**, **D**) FGF and PDGF trigger PGAM1 Y119 phosphorylation. Left: A549 cells were transfected with Flag-PGAM1 for 6 h, then cultured in serum-free medium and simultaneously treated with Zoligratinib (10 μM), Crenolanib (20 pM), Saracatinib (1 μM) for 48 h. Subsequently, the cells were incubated with FGF1 and PDGFA (50 ng/ml) for 6 h. Flag (PGAM1)-associated proteins were immunoprecipitated and analyzed by WB. Right: Quantification of IP. Relative levels of pY119, PKM2 or 3-pHis were normalized to that of Flag (PGAM1) for each group. Data information: for WB in (**A**–**D**), one representative experiment out of three was shown. IgG served as a negative control. Data represent mean ± SD of three (**A**–**D**) independent experiments with significance determined by two-way ANOVA test; ****$P < 0.0001$, ***$P < 0.001$, **$P < 0.01$, *$P < 0.05$. ns nonsignificant. Source data are available online for this figure.

© The Author(s)

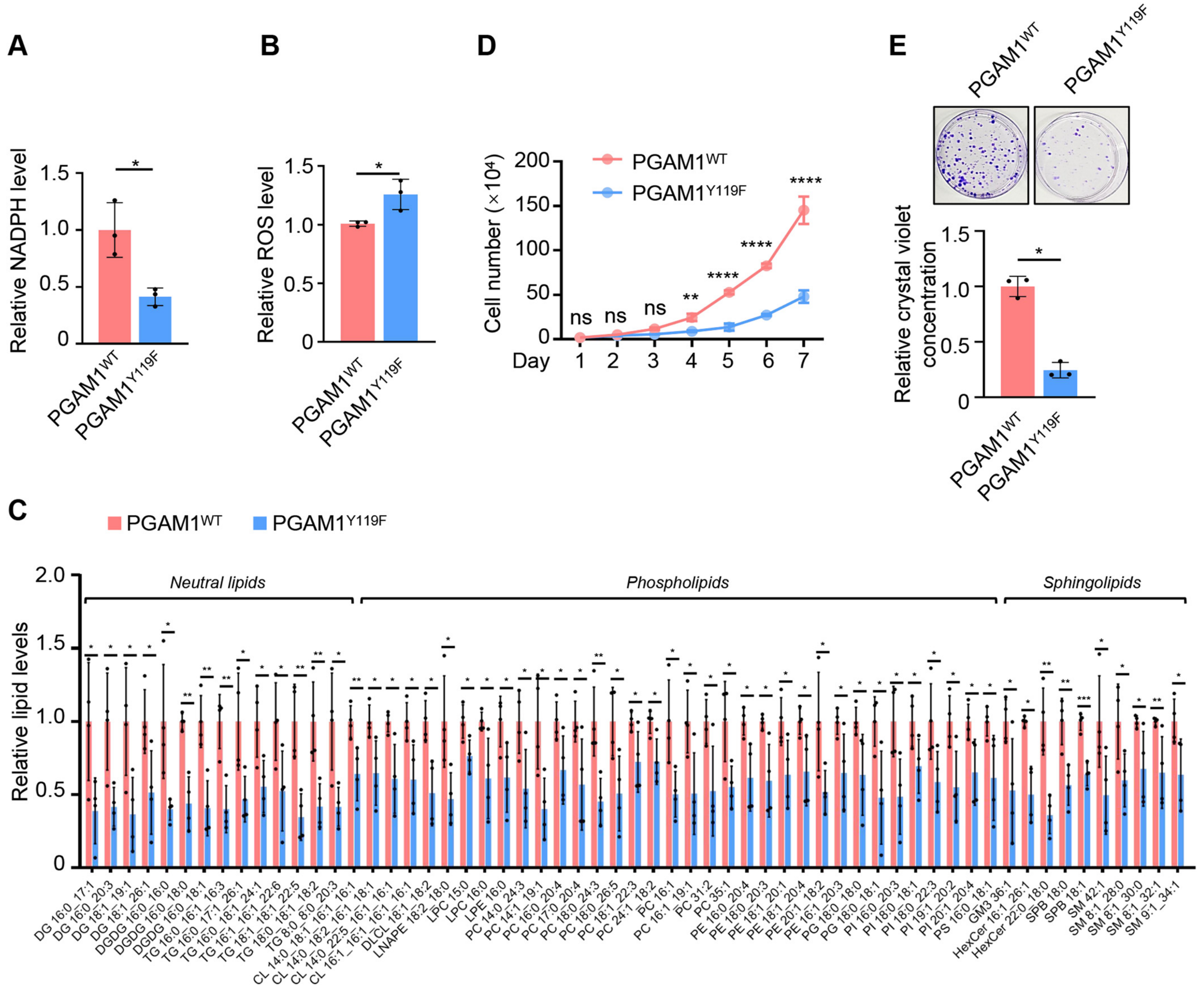

Figure EV5. PGAM1 Y119 mutation compromises cell proliferation.

(**A**, **B**) PGAM1 Y119 mutation impairs redox balance. Intracellular NADPH (**A**) and ROS (**B**) levels in PGAM1 WT- and Y119F mutation- rescued cells were examined with commercially available kits. $n = 3$ independent experiments. (**C**) Impact of PGAM1 Y119 mutation on lipid levels. Lipid levels in PGAM1 WT- and Y119F mutant-restored cells were analyzed by UHPLC-HRMS, which is related to Fig. 5D. $n = 4$ biological replicas (**D**) PGAM1 Y119 mutation hampers cell proliferation. PGAM1 WT- and Y119F mutant-restored cells were seeded with the same number and then counted every 24 h. The proliferation curves were drawn. $n = 3$ independent experiments. (**E**) PGAM1 Y119 mutation inhibits cell colony formation. PGAM1 WT- and Y119F mutation-rescued cells were seeded at the same number and cultured for 20 days. Cell colonies were stained by crystal violet. Top: Representative images and (bottom) quantifications were shown. $n = 3$ independent experiments. Data information: data represents mean ± SD ($n \geq 3$) with significance determined by Student's t test (**A–E**); ****$P < 0.0001$, ***$P < 0.001$, **$P < 0.01$, *$P < 0.05$. ns nonsignificant. Source data are available online for this figure.
